# Supplementary material for: Protocell arrays for simultaneous detection of diverse analytes
Source: Nat Commun. 2021 Sep 29;12:5724. doi: 10.1038/s41467-021-25989-3 (PMC8481512; doi:10.1038/s41467-021-25989-3)
Supplement: Supplementary file 1 — Supplementary Information [file 41467_2021_25989_MOESM1_ESM.docx]

Supplementary Information for

Protocell arrays for simultaneous detection of diverse analytes

Yan Zhang^1^, Taisuke Kojima^2^, Ge-Ah Kim^3^, Monica P. McNerney^1,4^, Shuichi Takayama^2, *^, Mark P. Styczynski^1, *^

^1^ School of Chemical & Biomolecular Engineering, Georgia Institute of Technology

^2^ Department of Biomedical Engineering, Georgia Institute of Technology

^3^ School of Materials Science and Engineering, Georgia Institute of Technology

^4^ Current address: Department of Systems Biology, Harvard Medical School

^*^ Corresponding Authors. Correspondance and requests for materials should be addressed to ST (email: [takayama@gatech.edu](mailto:takayama@gatech.edu)) or MPS (email: [mark.styczynski@chbe.gatech.edu](mailto:mark.styczynski@chbe.gatech.edu))

Table of Contents

[Supplementary Fig. 1 Injection-molded polystyrene plates used in protocell array reactions. 3](#_Toc81295572)

[Supplementary Fig. 2 Characterization of CFE reaction compartmentalization and protein production in PEG-Ficoll and PEG-dextran ATPS. 4](#_Toc81295573)

[Supplementary Fig. 3 Individual biological replicates of simultaneous detection of multiple model small molecules in membrane-less protocell arrays (Figure 3). 6](#_Toc81295574)

[Supplementary Fig. 4 Individual biological replicates for simultaneous detection of multiple model RNA sequences in membrane-less protocell arrays (Fig. 4c-d). 8](#_Toc81295575)

[Supplementary Fig. 5 Individual biological replicates for simultaneous detection of multiple model linear DNA sequences in membrane-less protocell arrays (Fig. 4e-f). 10](#_Toc81295576)

[Supplementary Fig. 6 Comparison of toehold switch sensitivity to RNA triggers in single-phase CFE and protocell arrays. 12](#_Toc81295577)

[Supplementary Fig. 7 Comparison of toehold switch sensitivity to trigger-encoding linear DNA in single-phase CFE and protocell arrays. 13](#_Toc81295578)

[Supplementary Fig. 8 Co-expression of triggers B and H in single-phase CFE reactions mutually represses their output. 14](#_Toc81295579)

[Supplementary Fig. 9 Protocell array setup for simultaneous detection of zinc and vitamin B_12_ in protocell arrays. 15](#_Toc81295580)

[Supplementary Fig. 10 Protocell array setup for simultaneous detection of *B. theta* and STEC bacteria. 16](#_Toc81295581)

[Supplementary Fig. 11 Individual biological replicates for simultaneous detection of multiple clinically relevant biomarkers across multiple molecular classes in a water matrix (Fig. 5b). 17](#_Toc81295582)

[Supplementary Fig. 12. Characterization of multi-modal analyte detection in 10% human serum matrix with individual biological replicates. 20](#_Toc81295583)

[Supplementary Fig. 13. Individual biological replicates for simultaneous detection of multiple clinically relevant biomarkers across multiple molecular classes in a 20% serum matrix (Fig. 5c). 21](#_Toc81295584)

[Supplementary Fig. 14 ICP-MS measurement of zinc concentrations in untreated and Chelex-100 treated human serum. Data presented are from one measurement. 23](#_Toc81295585)

[Supplementary Fig. 15 Protocell array output can be interpreted without equipment. 24](#_Toc81295586)

[Supplementary Fig. 16 Individual biological replicates for sensor activation and pigment production in freshly assembled colorimetric sensor reactions. 26](#_Toc81295587)

[Supplementary Fig. 17 Individual biological replicates for sensor activation and pigment production in freeze-dried and rehydrated reactions. 27](#_Toc81295588)

[Supplementary Fig. 18 Protocell arrays are compatible with the isothermal nucleic acid amplification technique. 29](#_Toc81295589)

[Supplementary Fig. 19 Validation of target-specific trigger amplification. 31](#_Toc81295590)

[Supplementary Fig. 20 Comparison of 𝜒DNA and GamS protein for their linear DNA protection capabilities. 32](#_Toc81295591)

[Supplementary Fig. 21 Initial validation of *B. theta*, Stx1, and Stx2 switch activation in response to their cognate triggers in CFE reactions. 33](#_Toc81295592)

[Supplementary Table 1 Description of lysates, plasmid concentrations, and reaction additives present in protocell arrays or CFE reactions in each figure. 34](#_Toc81295593)

[Supplementary Table 2. Primers used for trigger DNA amplification. Lowercase, unlabeled sequences are protective regions to decrease endonuclease degradation. 37](#_Toc81295594)

*
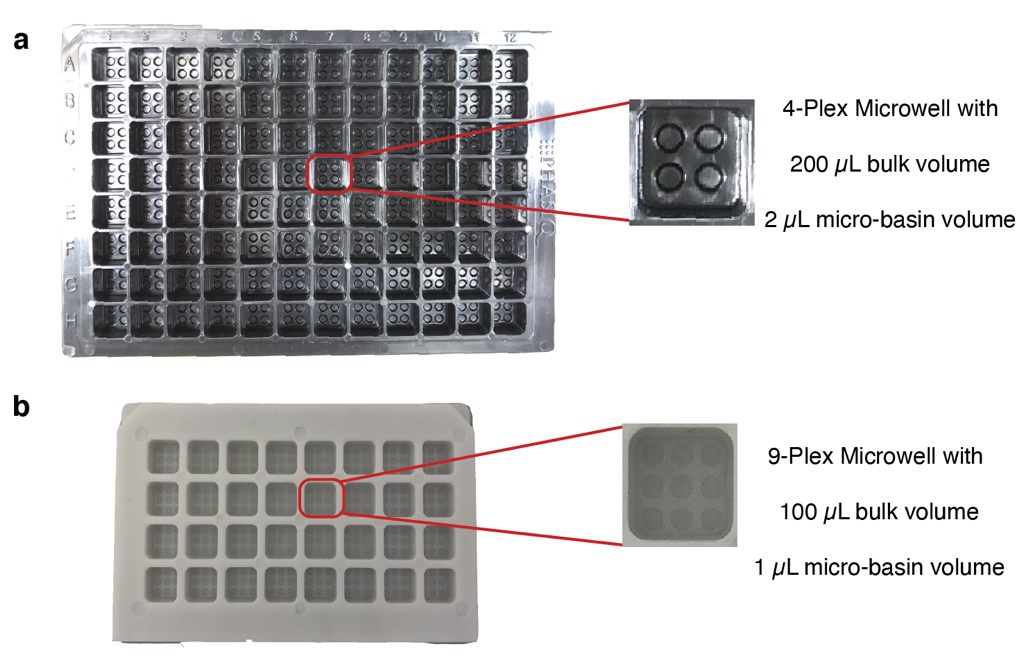
*

**Supplementary Fig. 1** Injection-molded polystyrene plates used in protocell array reactions. Microwell layout and spacing are based on 96-well microplate standards from the Society for Laboratory Automation and Screening. **a** Black microwell plate with 2x2 arrays of 2 µL volume micro-basins for protocell placement. **b** White microwell plate with 3x3 arrays of 1 µL volume micro-basins for protocell placement. All microwell plates used in this study were manufactured by PHASIQ, Inc (Ann Arbor, Michigan) and engineering drawings for both plates are included with this manuscript. Alternative methods to create homemade microwell plates of similar designs are described in previous reports^1,2^.


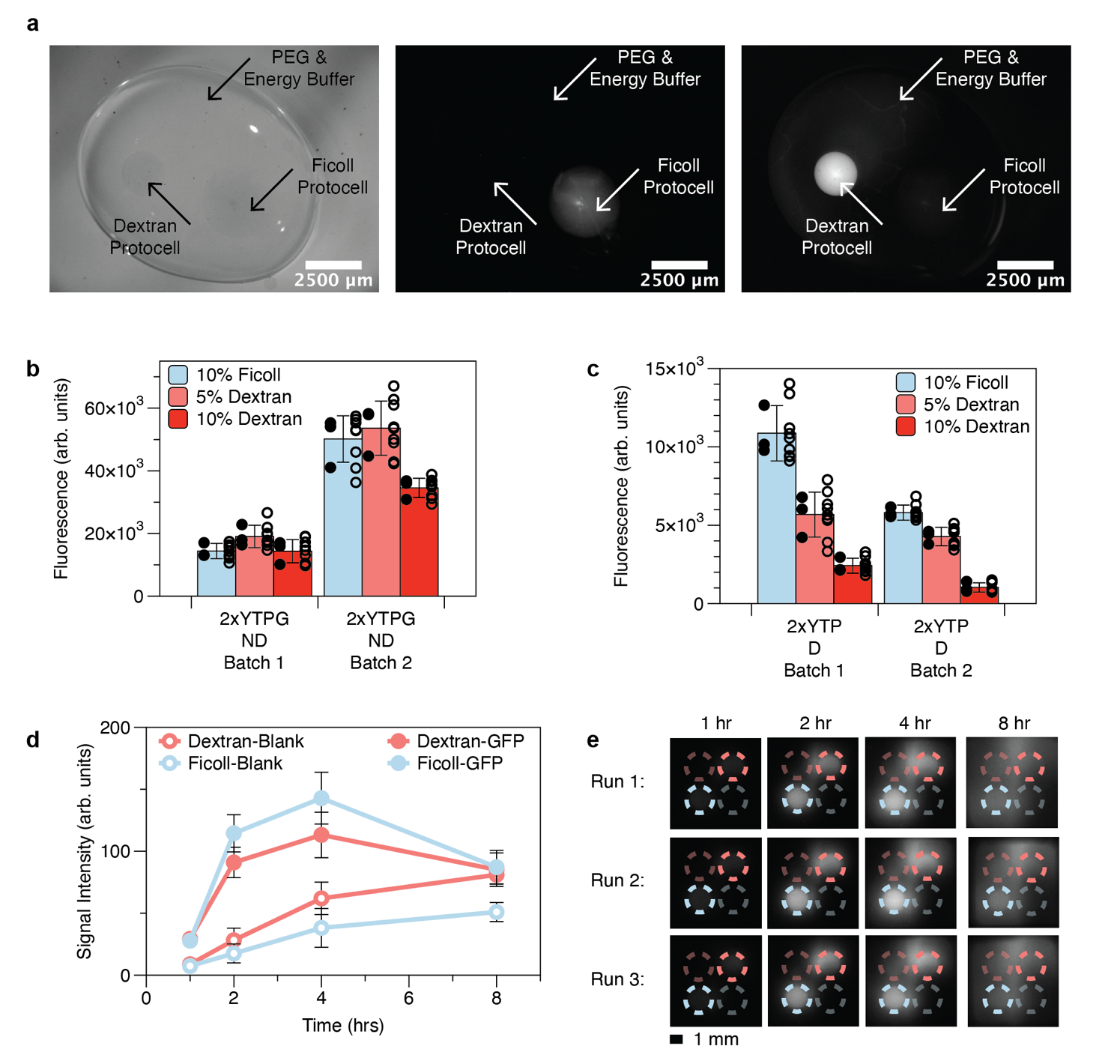


**Supplementary Fig. 2** Characterization of CFE reaction compartmentalization and protein production in PEG-Ficoll and PEG-dextran ATPS. **a** Bright field and fluorescent microscopy images of CFE lysate compartmentalized in 10% Ficoll and 10% dextran protocells, with the bulk phase containing 1x energy mix and 5% PEG. Ficoll and dextran protocells were mixed with 0.2 v/v% TRITC-labeled Ficoll and 0.03 v/v% FITC-labeled dextran, respectively, to aid in polymer visualization on a fluorescent stereomicroscope (Leica M205 FCA, DSR filter set (Ex: 545/30 nm and Em: 620/60nm) for fluorescence imaging of TRITC-Ficoll (middle) and GFP filter set (Ex: 470/40nm and Em: 525/50 nm) for FITC-Dex (right)). Scale bar is 2.5 mm. Data represented are from one experiment. **b-c** PEG-Ficoll ATPS shows better compatibility across cell-free lysates prepared with different growth media and lysate processing steps. Different CFE lysates were tested for GFP production in protocells formed by 10% Ficoll, 5% dextran, and 10% dextran with energy mix supplemented in a 5% PEG bulk phase. Compared to Ficoll, dextran encapsulation showed lower GFP production in CFE lysates prepared using 2xYTP growth media and that have undergone run-off reaction and dialysis. This behavior was consistent across two batches of CFE lysate made on different days. Reactions were incubated at 37˚C for 3 hours. Details on plasmid concentration are provided in Supplementary Table 1. Data are presented as mean values ± SD of 9 replicates (3 biological replicates x 3 technical replicates). Each biological replicate represents an independently assembled reaction on a different day. Solid-filled circles represent the average of each biological replicate, and hollow circles represent all data points. **d** Time course quantification of 10% Ficoll and 5% dextran CFE protocells with and without plasmid encoding for GFP expression. Disruption of the biphasic partitioning of GFP was observed beyond 4 hours. This could be caused by GFP diffusion from protocell to the bulk phase^3,4^. Typical cell-free sensing reactions are on the order of 1 to 3 hours, making issues at longer time scales less relevant. Data are presented as mean values ± SD of 9 replicates (3 biological replicates x 3 technical replicates). Each biological replicate represents an independently assembled reaction on a different day. Details on plasmid concentration can be found in Supplementary Table 1. **e** Representative time-course fluorescence image of compartmentalized cell-free reactions in protocell arrays for each biological replicate. Blue circles indicate Ficoll-formed protocell with a plasmid coding for GFP expression (bright blue) and without plasmid (faded blue). Red circles indicate dextran-formed protocell with a plasmid coding for GFP expression (bright red) and without plasmid (faded red). Scale bar is 1 mm.


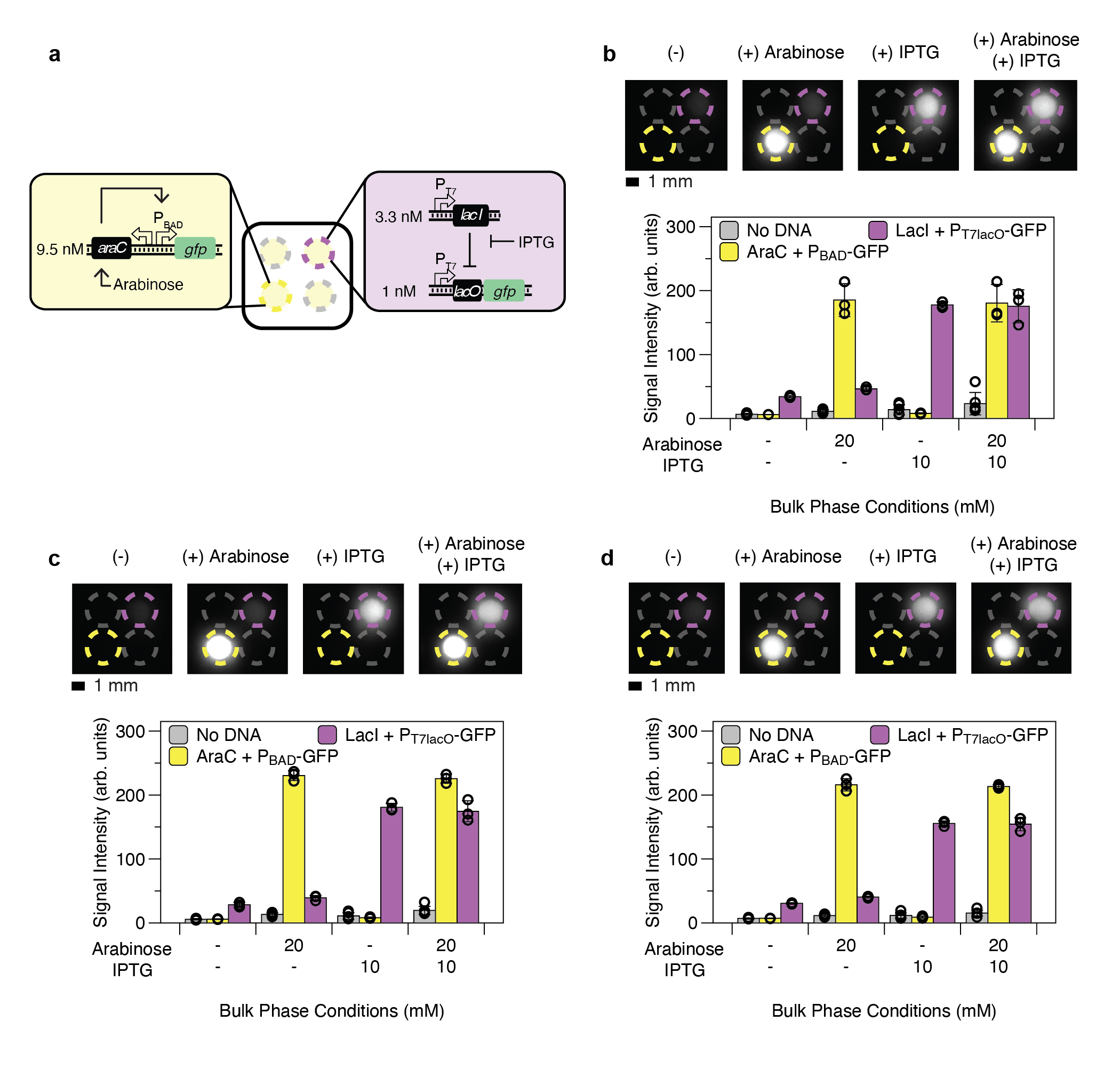


**Supplementary Fig. 3** Individual biological replicates of simultaneous detection of multiple model small molecules in membrane-less protocell arrays (Figure 3). Each subpanel shows the results of an independently assembled experiment on a different day. Each protocell sensor is only activated when its cognate small molecule is present in the bulk phase. Reactions were incubated at 37˚C for 3 hours. Details on CFE lysate and plasmid concentration are provided in Supplementary Table 1. **a** Schematic of protocell array reaction setup for simultaneous analysis of two small molecules, as in Fig. 3a. Yellow and purple circles indicate micro-basins containing arabinose and IPTG sensors, respectively, while gray circles are micro-basins containing CFE protocells without plasmid. **b-d** Representative fluorescence images and pixel quantification of small molecule protocell array sensors under different bulk phase conditions for each biological replicate. Small molecules added to the bulk phase for each condition are indicated above each image. Scale bar is 1 mm. Data are presented as mean values ± SD of 3 technical replicates, and hollow circles represent all data points. Controls with no DNA have 6 technical replicates.


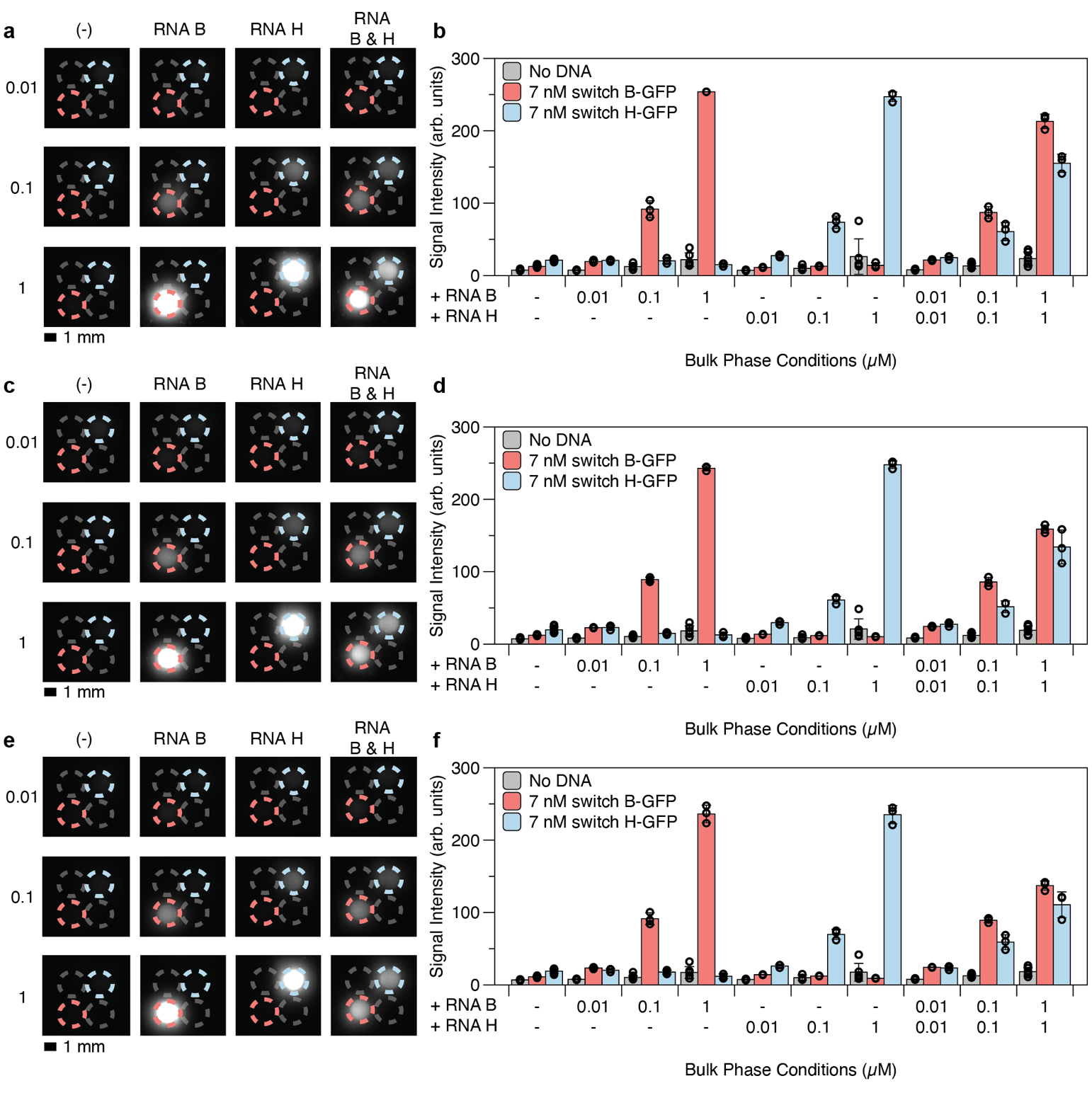


**Supplementary Fig. 4** Individual biological replicates for simultaneous detection of multiple model RNA sequences in membrane-less protocell arrays (Fig. 4c-d). Reactions were incubated at 37˚C for 3 hours. Details on CFE lysate, plasmid concentrations, and reaction additives are provided in Supplementary Table 1. **a, c, e** Representative fluorescence images of simultaneous RNA detection for each biological replicate, independently assembled on a different day. Each protocell sensor is only activated when its cognate RNA trigger is present in the bulk phase. Images in the same column have the same RNA trigger(s) added. Images in the same row have the same concentration of trigger(s) added. Red and blue circles indicate micro-basins containing toehold switches B and H, respectively. Gray circles indicate CFE reactions without plasmid DNA. Scale bar is 1 mm. **b, d, f** Quantification of fluorescence images for each biological replicate. Data are presented as mean values ± SD of 3 technical replicates, and hollow circles represent individual data points. Protocells with no DNA have 6 technical replicates. For bulk phase without RNA triggers (- RNA B & H), data are presented as mean values ± SD of for protocell sensors (switchB-GFP and switchH-GFP) of 9 replicates (3 biological replicates x 3 technical replicates, with one biological replicate from 0.01 µM detection, one from 0.1 µM detection, and one from 1 µM detection). Data for protocells with no DNA in the no trigger condition (- RNA B & H) represent mean values ± SD of 18 replicates (3 biological replicates x 6 technical replicates, with one biological replicate from 0.01 µM detection, one from 0.1 µM detection, and one from 1 µM detection). Experiments at different RNA trigger concentrations were run separately to reduce the time difference between loading the first and last protocells. As a result, no trigger, 0.01 µM trigger B, 0.01 µM trigger H, and 0.01 µM triggers B & H were run as one set. Similar runs were done for RNA triggers at higher concentrations, and the first runs across different trigger concentrations were grouped into one subpanel, resulting in 3 biological replicates for the no trigger condition and single biological replicates for different trigger concentrations in subpanels **a-b**. A similar procedure was used for the second and third runs to create subpanels **c-d** and **e-f**.


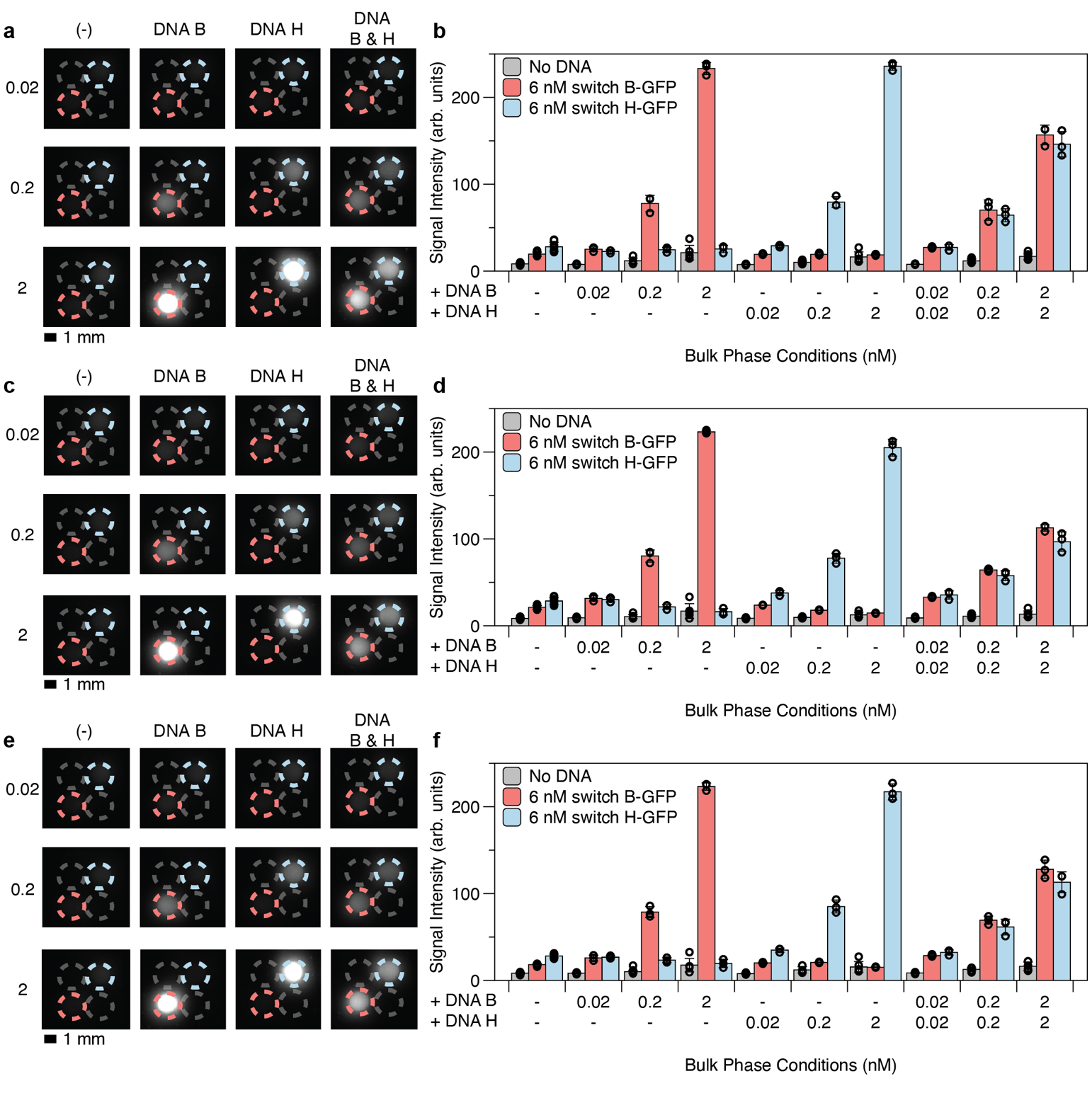


**Supplementary Fig. 5** Individual biological replicates for simultaneous detection of multiple model linear DNA sequences in membrane-less protocell arrays (Fig. 4e-f). Reactions were incubated at 37˚C for 3 hours. Details on CFE lysate, plasmid concentrations, and reaction additives can be found in Supplementary Table 1. **a, c, e** Representative fluorescence image of simultaneous linear DNA detection for each biological replicate independently assembled on a different day. Each protocell sensor is only activated when its cognate linear DNA is present in the bulk phase. Images in the same column have the same linear DNA trigger(s) added. Images in the same row have the same concentration of trigger(s) added. Red and blue circles indicate micro-basins containing toehold switches B and H, respectively. Gray circles indicate CFE reaction without plasmid DNA. Scale bar is 1 mm. **b, d, f** Quantification of fluorescence images for each biological replicate. Data are presented as mean values ± SD of 3 technical replicates, and hollow circles represent individual data points. Protocells with no DNA have 6 technical replicates. Data in sensing reactions for the no trigger condition (- DNA B & H) in each subpanel represent mean values ± SD of 9 replicates (3 biological replicates x 3 technical replicates, with one biological replicate from 0.02 nM detection, one from 0.2 nM detection, and one from 2 nM detection). Data for protocells with no DNA in the no trigger condition (- DNA B & H) represent mean values ± SD of 18 replicates (3 biological replicates x 6 technical replicates, with one biological replicate from 0.02 nM detection, one from 0.2 nM detection, and one from 2 nM detection). Bulk phase conditions at different DNA trigger concentrations were run independently to reduce the time difference between loading the first and last protocells. As a result, no trigger, 0.02 nM trigger B, 0.02 nM trigger H, and 0.02 nM triggers B & H were run as one set. Similar runs were done for DNA triggers at higher concentrations, and all the first runs across different trigger concentrations were grouped into one subpanel, resulting in 3 biological replicates for the no trigger condition and single biological replicates for different trigger concentrations in subpanels **a-b**. A similar procedure was used for the second and third runs to create subpanels **c-d** and **e-f**.


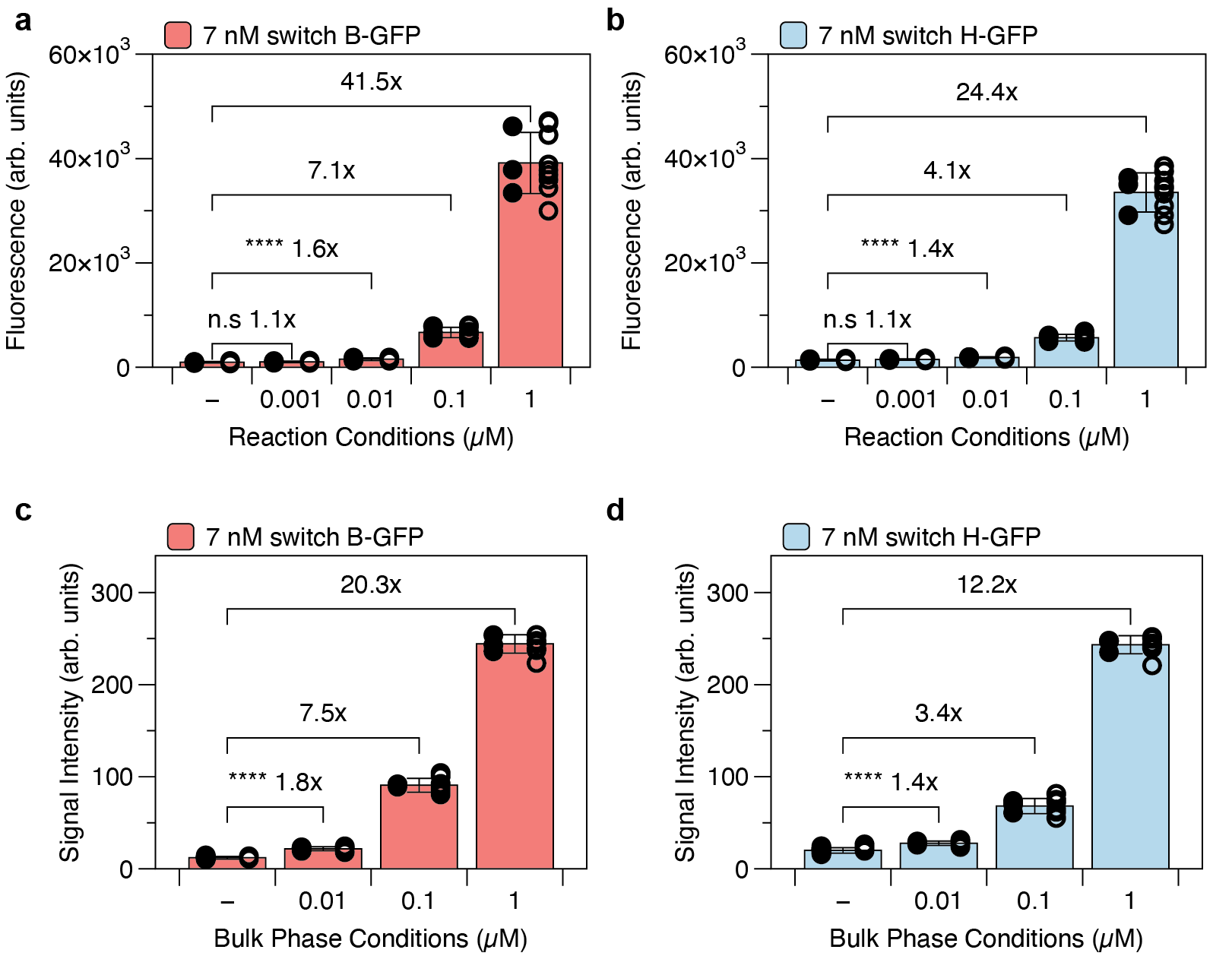


**Supplementary Fig. 6** Comparison of toehold switch sensitivity to RNA triggers in single-phase CFE and protocell arrays. Reactions were incubated at 37 ˚C for 3 hours before fluorescence measurement on a plate reader (for single-phase CFE reactions) or a fluorescence imager (for protocell arrays). Protocell arrays show similar fold activation and limits of detection (approximately 10 nM) for RNA triggers compared to single-phase CFE reactions. **a-b** Toehold switch B and H (respectively) activation at various RNA trigger concentrations in single-phase CFE reactions. Data are presented as mean values ± SD of 9 replicates (3 biological replicates x 3 technical replicates). Each biological replicate represents an independently assembled reaction on a different day. **c-d** Toehold switch B and H (respectively) activation at various RNA trigger concentrations in protocell arrays. Data presented are the same set of data presented in Fig. 4c-d and Supplementary Fig. 4. Data are presented as mean values ± SD of 9 replicates (3 biological replicates x 3 technical replicates), except for baseline conditions of switchB-GFP and switchH-GFP in protocell array reactions, where each has 27 replicates (9 biological replicates x 3 technical replicates). Solid-filled circles represent the average of each biological replicate, and hollow circles represent all data points. Statistical significance relative to basal expression is calculated using a two-tailed Student’s t-test (n=9 for all samples, except for baseline conditions of switchB-GFP and switchH-GFP in protocell array where each has n=27). Asterisk (****) indicates p-value of 1.69E-05 for **a**, 5.84E-06 for **b**, 3.74E-17 for **c**, and 4.88E-08 for **d**. n.s indicates no statistical significance. Details on CFE lysate, plasmid concentrations, and reaction additives are provided in Supplementary Table 1.


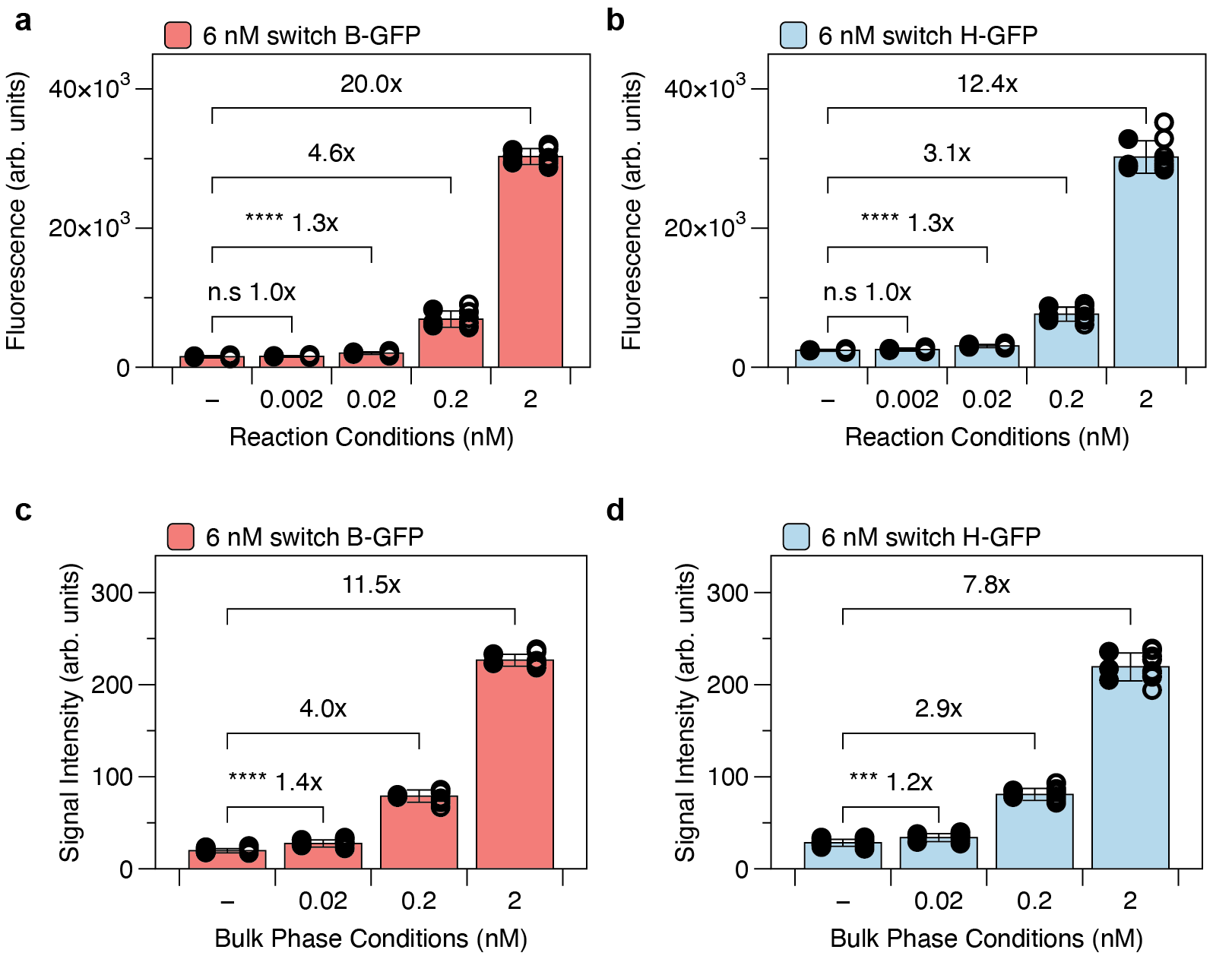


**Supplementary Fig. 7** Comparison of toehold switch sensitivity to trigger-encoding linear DNA in single-phase CFE and protocell arrays. Reactions were incubated at 37 ˚C for 3 hours before fluorescent measurement on a plate reader (for CFE reactions) or a fluorescence imager (for protocell arrays). Protocell arrays show similar limits of detection (approximately 0.02 nM) for linear DNA encoding RNA triggers compared to single-phase CFE reactions. **a-b** Toehold switch B and H (respectively) activation at various DNA trigger concentrations in single-phase CFE reactions. Data are presented as mean values ± SD of 9 replicates (3 biological replicates x 3 technical replicates). Each biological replicate represents an independently assembled reaction on a different day. **c-d** Toehold switch B and H (respectively) activation at various DNA trigger concentrations in protocell arrays. Data presented are the same set of data presented in Fig. 4e-f and Supplementary Fig. 5. Data are presented as mean values ± SD of 9 replicates (3 biological replicates x 3 technical replicates), except for baseline conditions of switchB-GFP and switchH-GFP in protocell array reactions, where each has 27 replicates (9 biological replicates x 3 technical replicates). Statistical significance relative to basal expression is calculated using a two-tailed Student’s t-test (n=9 for all samples except for baseline conditions (-) of switchB-GFP and switchH-GFP in protocell array format, which both have n=27). Asterisk (****) indicates p-value of 1.01E-05 for **a**, 2.43E-06 for **b**, 9.54 E-17 for **c**. Asterisk (***) indicates p-value of 6.19E-04 for **d**, and n.s indicates no statistical significance. Solid-filled circles represent the average of each biological replicate, and hollow circles represent all data points. Details on CFE lysate, plasmid concentrations, and reaction additives can be found in Supplementary Table 1.


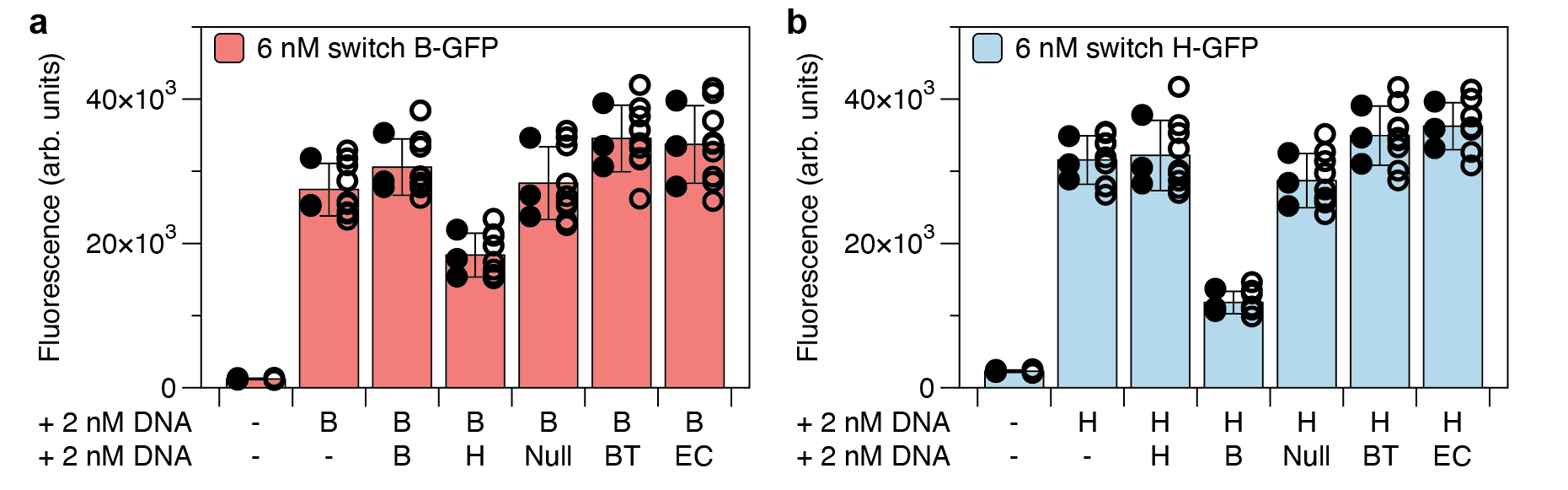


**Supplementary Fig. 8** Co-expression of triggers B and H in single-phase CFE reactions mutually represses their output. This inhibition effect is specific to the combination of triggers B and H, as evidenced by the lack of repression when random (Null) and previously characterized trigger sequences (*Bacteroides thetaiotaomicron* and *Escherichia coli*, B. t and E. c respectively)^6^ are also added to the CFE reactions. This effect occurs for any CFE sensing reaction, not just in protocell arrays. **a** Trigger B was co-expressed with different linear DNA transcribing RNA triggers. Only the addition of trigger H significantly reduced GFP production from the switchB-GFP plasmid. **b** Trigger H was co-expressed with different linear DNA transcribing RNA triggers, and only the addition of trigger B reduced GFP production from the switchH-GFP plasmid. Reactions were incubated at 37 ˚C for 3 hours before fluorescent measurement on a plate reader. Data are presented as mean values ± SD of 9 replicates (3 biological replicates x 3 technical replicates). Each biological replicate is an independently assembled reaction on a different day. Solid-filled circles represent the average of each biological replicate, and hollow circles represent all data points. Details on CFE lysate, plasmid concentrations, and reaction additives can be found in Supplementary Table 1.


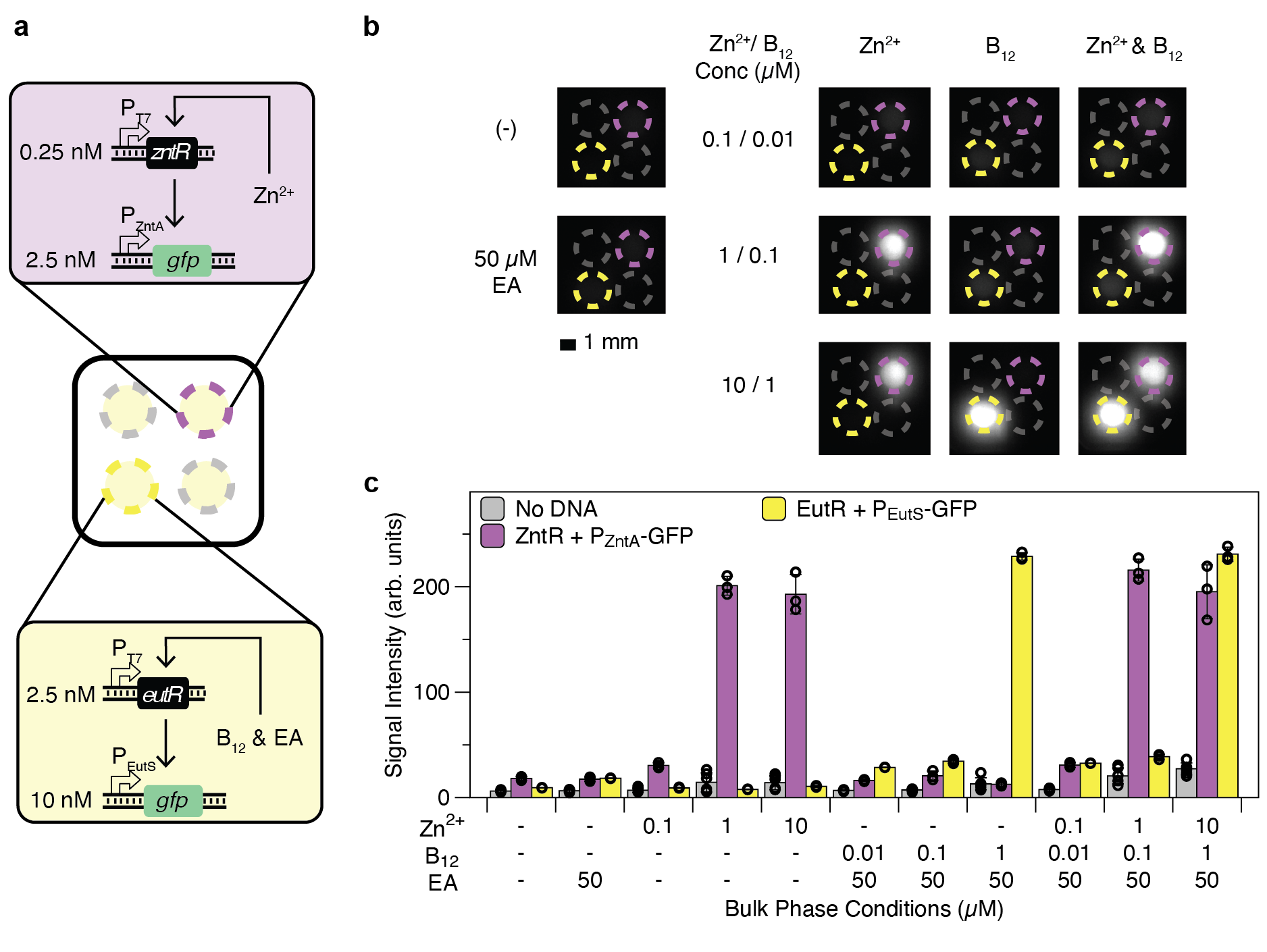


**Supplementary Fig. 9** Protocell array setup for simultaneous detection of zinc and vitamin B_12_ in protocell arrays. Reactions were incubated at 37 ˚C for 3 hours. **a** Schematics of zinc and vitamin B_12_ sensors and their concentrations. Zinc modulates GFP expression by binding to the transcriptional regulator ZntR, which in turn activates transcription from its cognate promoter P_zntA_ (purple schematic). Vitamin B_12_ modulates GFP expression by binding to the transcriptional regulator EutR with ethanolamine (EA) as a cofactor. EutR then activates transcription from its cognate promoter P_eutS_ (yellow schematic). Gray circles represent protocells with CFE reactions without plasmid DNA. Details on CFE lysate used and plasmid concentrations can be found in Supplementary Table 1. **b** Representative fluorescence images of simultaneous detection of small molecules at different input and concentration conditions. (-) indicates neither zinc nor vitamin B_12_ was added in the bulk phase, and 50 µM EA indicates a bulk phase condition with only the cofactor for vitamin B_12_ sensor. Zinc was tested at 0.1, 1, and 10 µM. Vitamin B_12_ was tested at 0.01, 0.1, and 1 µM. All micro-wells containing vitamin B_12_ in the bulk phase also have 50 µM EA as a cofactor for sensor activation. Scale bar is 1 mm. **c** Quantification of fluorescence images in **b** and their replicates. Data are presented as mean values ± SD of 3 technical replicates for sensing reactions and 6 technical replicates for protocells with no DNA. Hollow circles represent all data points.


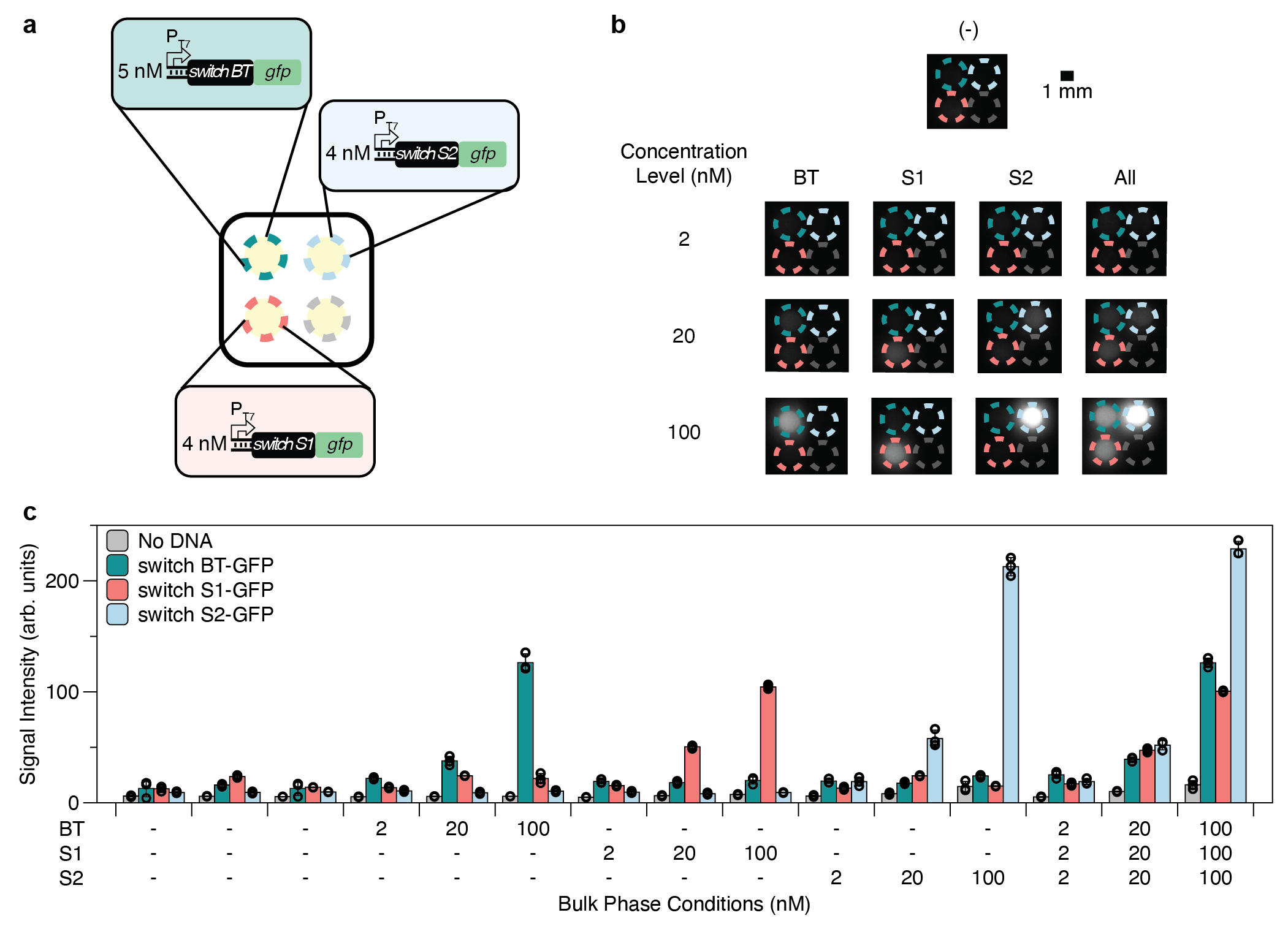


**Supplementary Fig. 10** Protocell array setup for simultaneous detection of *B. theta* and STEC bacteria. Reactions were incubated at 37 ˚C for 3 hours. **a** Teal, red, and blue circles represent protocells containing toehold switch sensors to detect *B. theta* (BT), Stx1 (S1), and Stx2 (S2) triggers, respectively. The gray circle represents a CFE protocell without plasmid DNA. **b** Representative fluorescence image of simultaneous nucleic acid detection at different input and concentration conditions. Location and color of each bacterial sensor as shown in **a**. Linear DNA for expression of triggers was amplified from genomic DNA of each bacterium and added to the bulk phase at concentrations of 2, 20, and 100 nM. Scale bar is 1 mm. Details on CFE lysate used and plasmid concentrations can be found in Supplementary Table 1. **c** Quantification of fluorescence images from **b** and their replicates. Data are presented as mean values ± SD of 3 technical replicates. Hollow circles represent all data points.


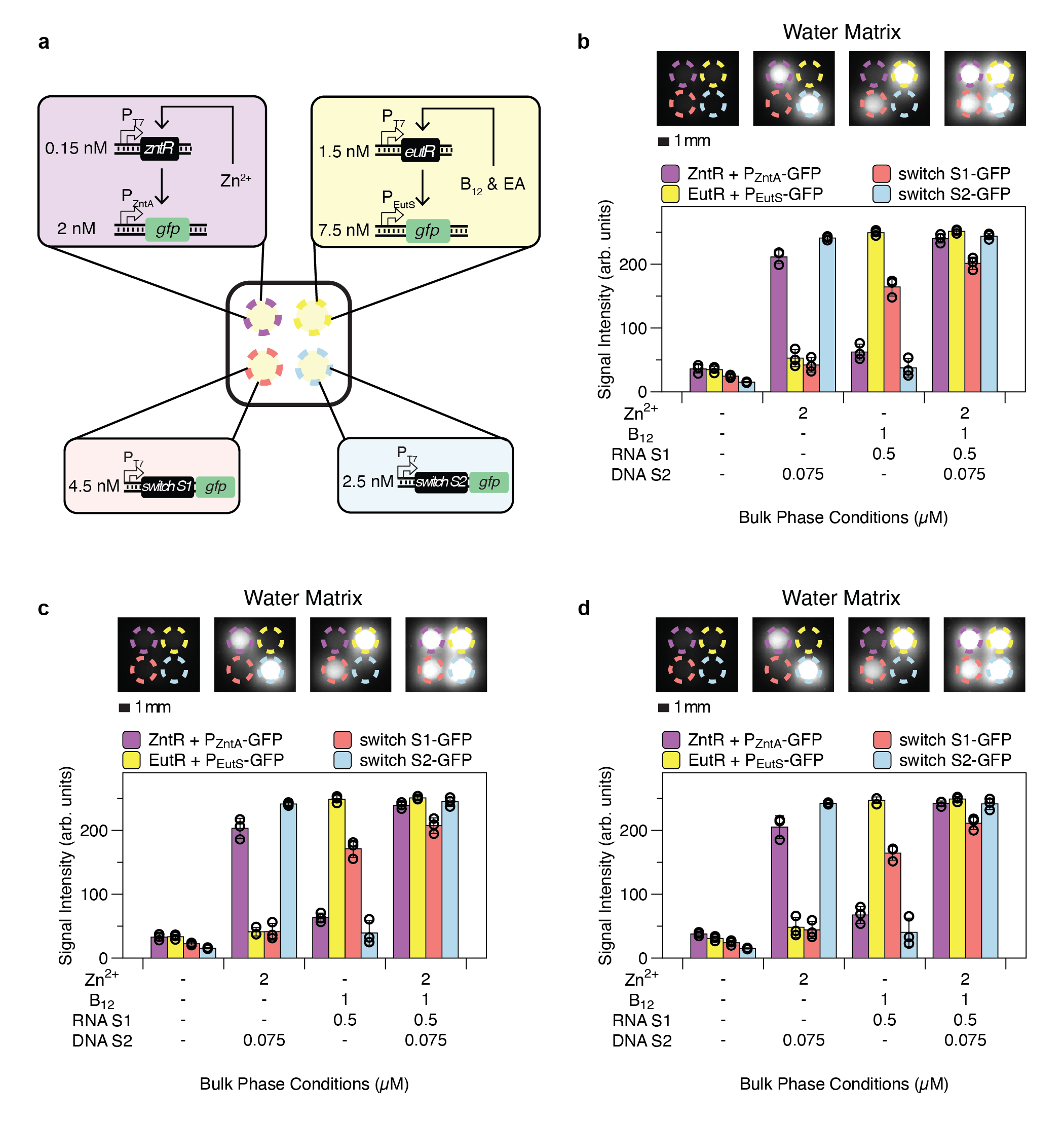


**Supplementary Fig. 11** Individual biological replicates for simultaneous detection of multiple clinically relevant biomarkers across multiple molecular classes in a water matrix (Fig. 5b). Reactions were incubated at 37˚C for 3 hours. **a** Schematic of protocell array setup for multi-modal detection of diverse classes of clinically relevant biomarkers. Purple and yellow circles indicate micro-basins containing zinc and vitamin B_12_ sensors, respectively. Red and blue circles indicate micro-basins containing Stx1 (S1) and Stx2 (S2) toehold switches, respectively.  **b-d** Each subpanel shows data from an independently assembled biological replicate on a different day. Representative fluorescence images for protocell sensor activation under different bulk phase conditions are provided for each biological replicate. Analytes added to the bulk phase for each condition are indicated in the corresponding bar graph below. Each protocell sensor is only activated when its cognate inducer is present in the bulk phase. Data are presented as mean values ± SD of 3 technical replicates, and hollow circles represent all data points. Scale bar is 1 mm. Details on CFE lysate used, plasmid concentrations, and reaction additives can be found in Supplementary Table 1.


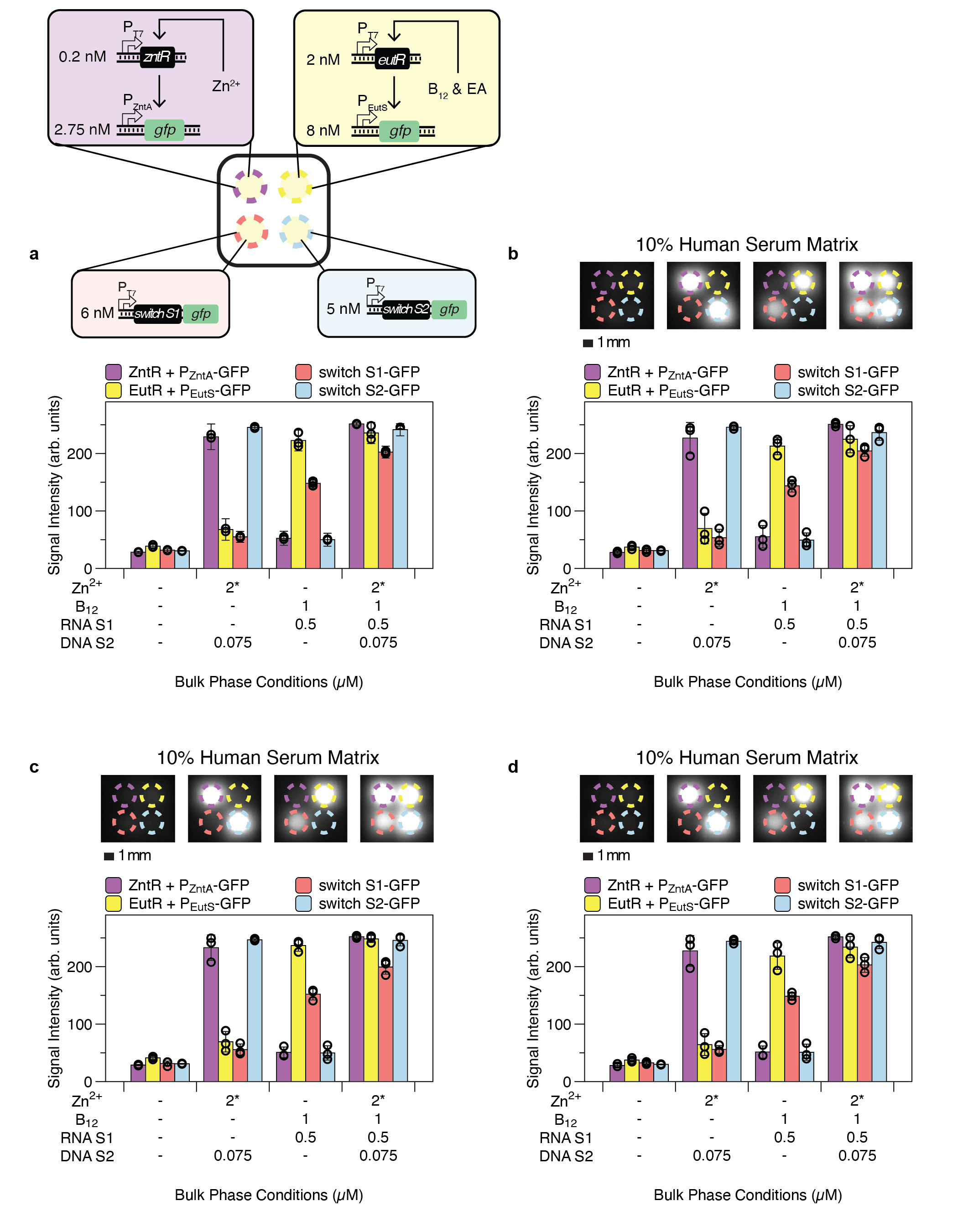


**Supplementary Fig. 12.** Characterization of multi-modal analyte detection in 10% human serum matrix with individual biological replicates. Reactions were incubated at 37 ˚C for 3 hours. **a** Schematic of protocell array setup for multi-modal detection of clinically relevant biomarkers in 10% human serum. Purple and yellow circles indicate micro-basins containing zinc and vitamin B_12_ sensors, respectively. Red and blue circles indicate micro-basins containing Stx1 (S1) and Stx2 (S2) toehold switches, respectively. Below the protocell array setup is the three-day average of multi-modal detection experiments run in 10% human serum. Each protocell sensor is only activated when its cognate inducer is present in the bulk phase. The asterisk (*) next to zinc concentration denotes the total Zn^2+^ in the bulk phase is 2 µM after accounting for the remaining zinc concentration in 10% chelated serum (0.22 µM) and the Zn^2+^ supplemented from solution (1.78 µM). Compared to reactions run in the water matrix, sensor plasmid concentrations were increased to detect analytes and produce similar GFP outputs. The inhibitory effects of human serum on CFE reactions, even after supplementing RNase inhibitor in the bulk phase, are consistent with published work^7^. Data are presented as mean values ± SD of 9 replicates (3 biological replicates x 3 technical replicates), and hollow circles represent the mean of each biological replicate. Details on CFE lysate used, plasmid concentrations, and reaction additives can be found in Supplementary Table 1. **b-d** Each subpanel represents an independently assembled biological replicate on a different day. Representative fluorescent images for protocell sensor activation under different bulk phase conditions are provided for each biological replicate. Data are presented as mean values ± SD of 3 technical replicates, and hollow circles represent all data points. Scale bar is 1 mm.


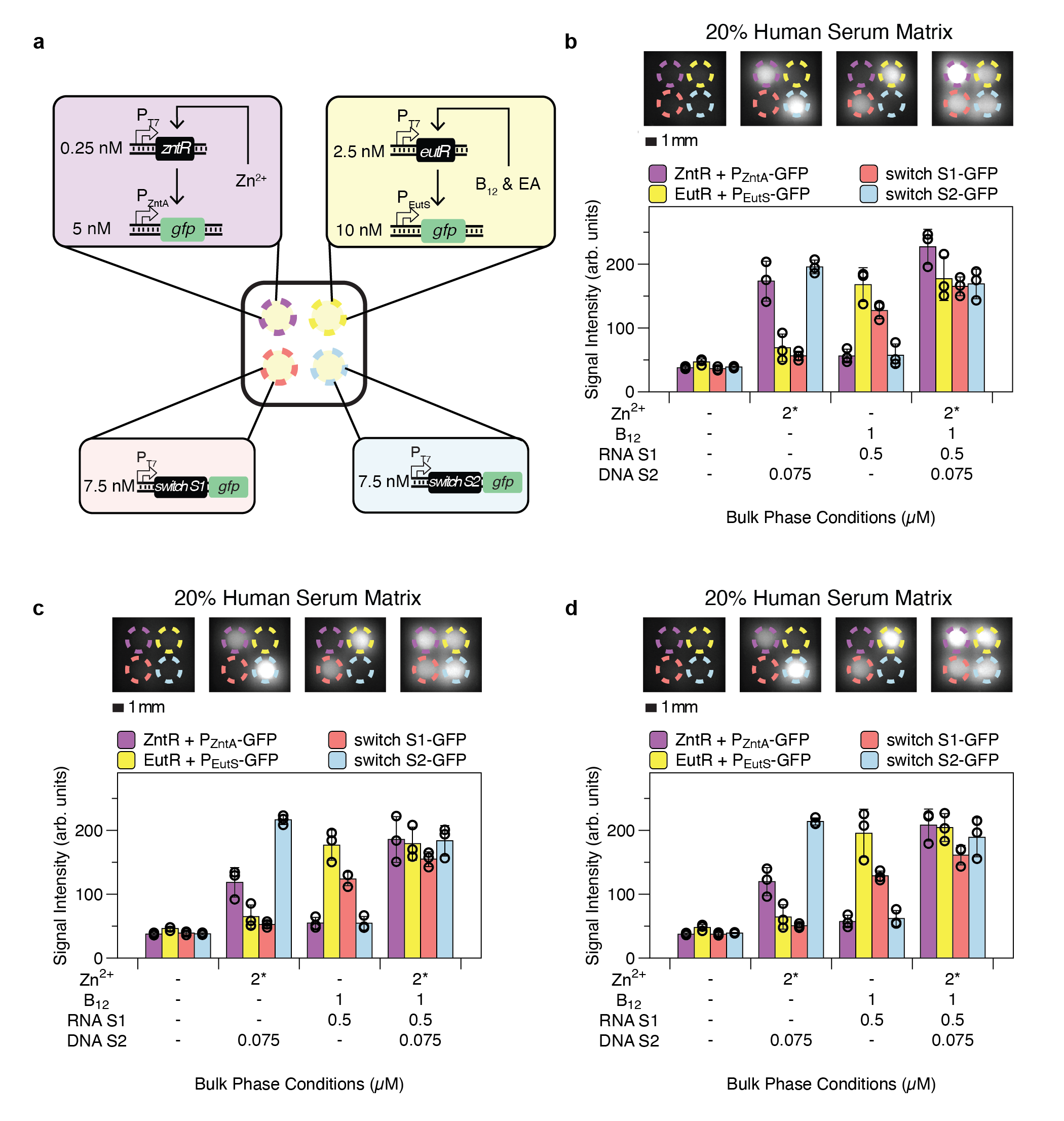


**Supplementary Fig. 13.** Individual biological replicates for simultaneous detection of multiple clinically relevant biomarkers across multiple molecular classes in a 20% serum matrix (Fig. 5c). Reactions were incubated at 37˚C for 3 hours. **a** Schematic of protocell array setup for multi-modal detection of diverse classes of clinically relevant biomarkers in 20% human serum. Purple and yellow circles indicate micro-basins containing zinc and vitamin B_12_ sensors, respectively. Red and blue circles indicate micro-basins containing Stx1 (S1) and Stx2 (S2) toehold switches, respectively.  **b-d** Each subpanel shows data from an independently assembled experiment on a different day. Representative fluorescence images for protocell sensor activation under different bulk phase conditions are provided for each biological replicate. Analytes added to the bulk phase for each condition are indicated in the corresponding bar graph below. Each protocell sensor is only activated when its cognate inducer is present in the bulk phase. Targets added to the bulk phase for each condition are indicated above each image. The asterisk (*) next to zinc concentration denotes the total Zn^2+^ in the bulk phase is 2 µM after accounting for the remaining zinc concentration in 20% chelated serum (0.43 µM) and the Zn^2+^ supplemented from solution (1.57 µM). Compared to reactions run in the water and 10% serum matrices, sensor plasmid concentrations needed to be increased to produce similar GFP output. The inhibitory effects of human serum on CFE reactions, even after supplementing the bulk phase with RNase inhibitor, are consistent with published work^7^. Data are presented as mean values ± SD of 3 technical replicates, and hollow circles represent all data points. Scale bar is 1 mm. Details on CFE lysate used, plasmid concentrations, and reaction additives can be found in Supplementary Table 1.


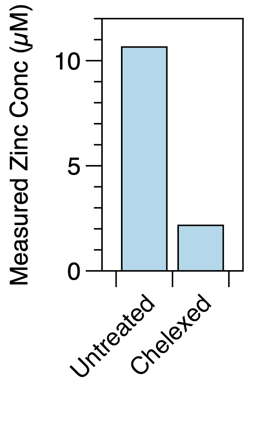


# **Supplementary Fig. 14** ICP-MS measurement of zinc concentrations in untreated and Chelex-100 treated human serum. Data presented are from one measurement.


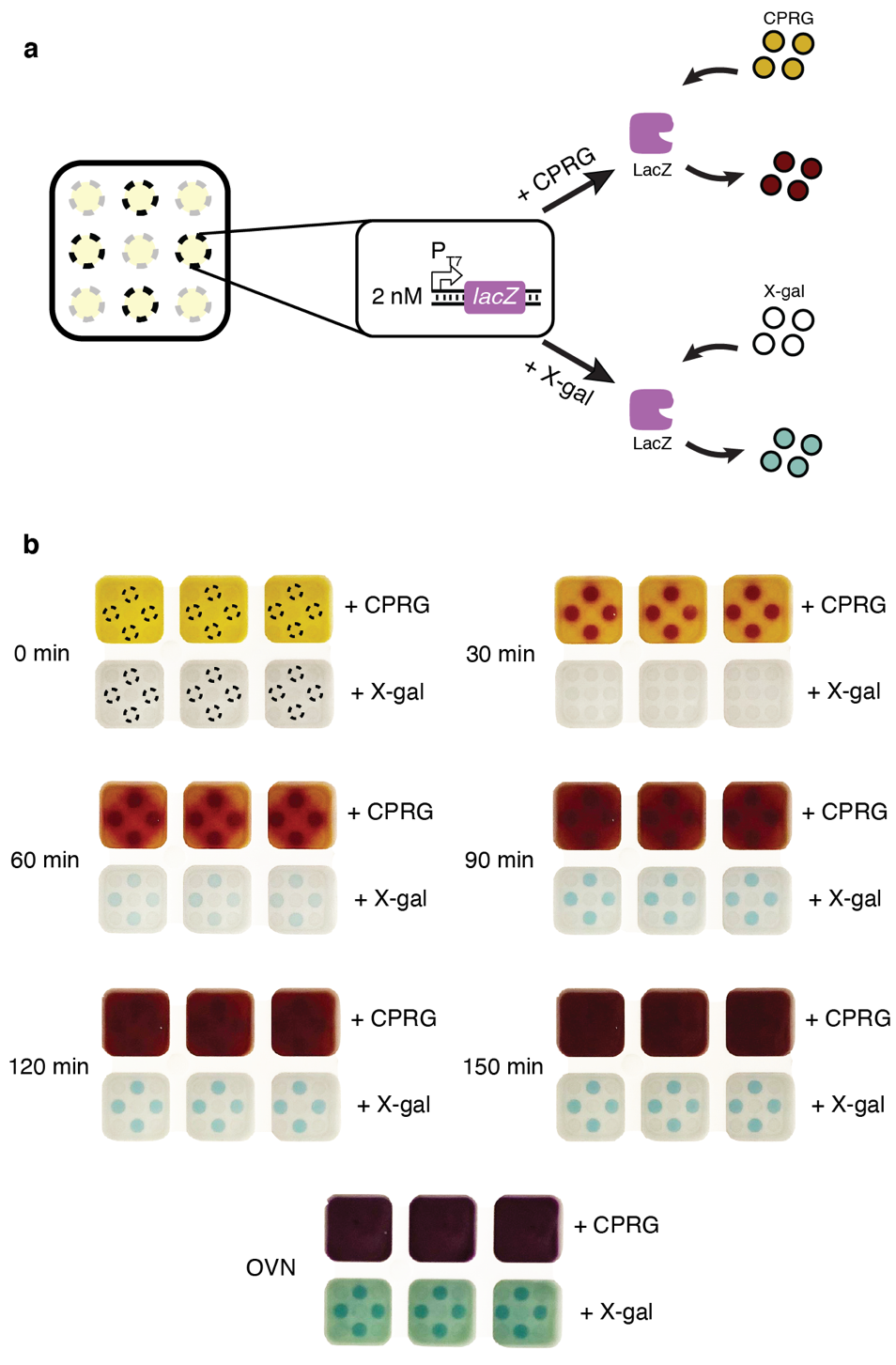


**Supplementary Fig. 15** Protocell array output can be interpreted without equipment. **a** Schematic of visually interpretable protocell array sensing reactions. Black circles represent micro-basins containing protocells that constitutively produce LacZ. The bulk phase contains either 0.6 mg/mL CPRG (yellow in panel **b**) or 0.2 mg/mL X-gal (colorless in panel **b**) as the substrate for pigment production. Once produced, the LacZ enzyme either cleaves CPRG to form chlorophenol red (CPR, red) or cleaves X-gal to form a blue precipitate. **b** Time course pigment production from two substrates. The bulk phase containing CPRG yields visible color change within 30 minutes of incubation, but the pigment readily diffuses into the bulk phase at later time points, making test results uninterpretable. Bulk phase containing X-gal produces visible color more slowly, with visible color change occurring at 1 hour. However, the color remains localized over the entire incubation period and still mostly localized even after overnight incubation (>14 hrs). Images presented are 3 technical replicates derived from the same reagent master mix. Details on CFE lysate used, plasmid concentrations, and reaction additives can be found in Supplementary Table 1.


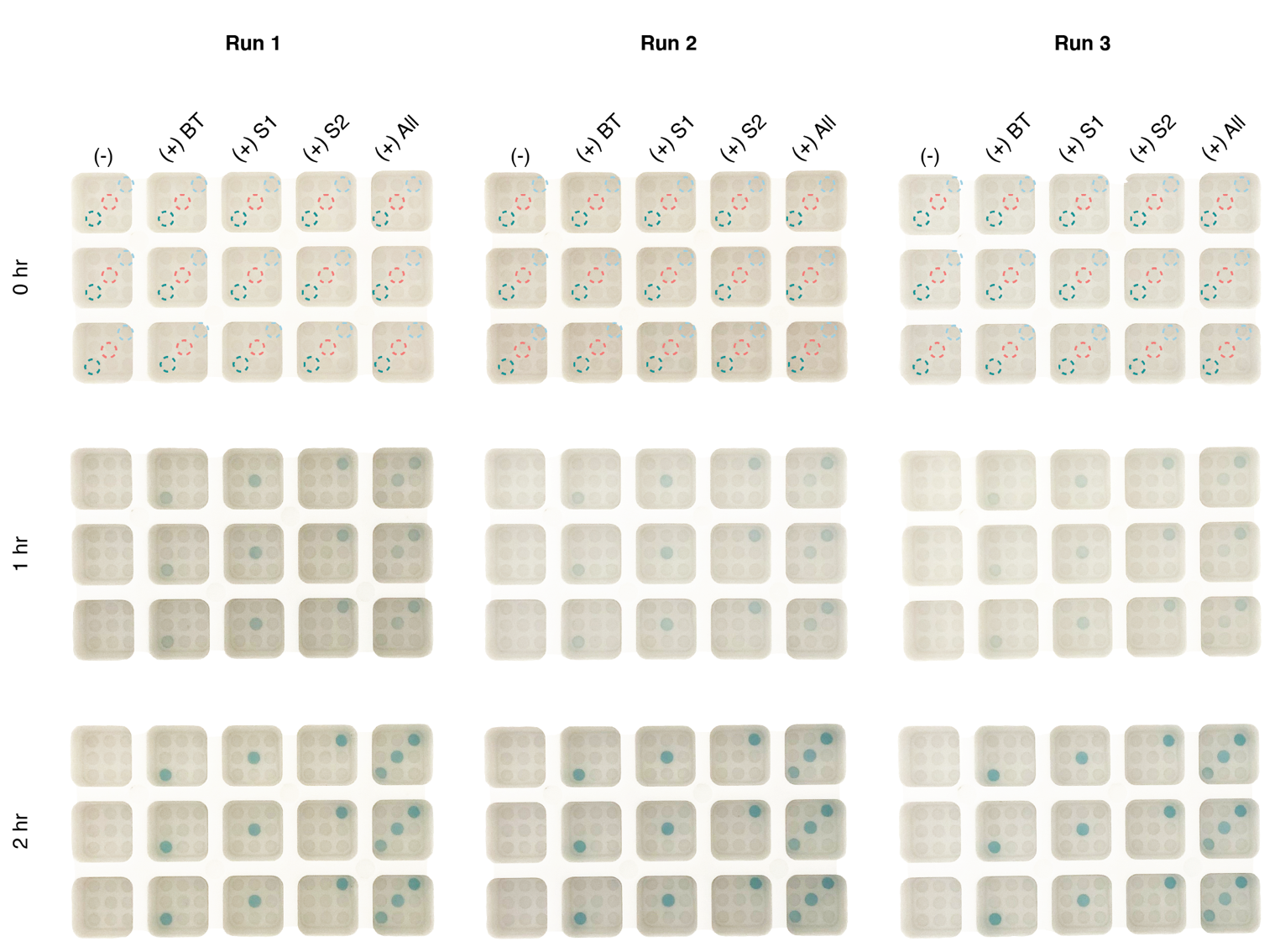


**Supplementary Fig. 16** Individual biological replicates for sensor activation and pigment production in freshly assembled colorimetric sensor reactions. Reactions were incubated at 37 ˚C for the specified amount of time indicated on the left of the figure. Each biological replicate run is an independently assembled experiment on a different day. Photos for protocell sensor activation under different bulk phase conditions are provided for each biological replicate, each with technical triplicates. Teal, red, and blue circles indicate micro-basins with protocells containing *B. theta* (BT), Stx1 (S1), and Stx2 (S2) toehold switches, respectively. Targets added to the bulk phase for each condition are indicated above each image. Linear triggers used were amplified from bacterial genomic DNA and added at 50 nM for each condition. Each protocell sensor was only activated when its cognate trigger was present in the bulk phase. Details on CFE lysate used, plasmid concentrations, and reaction additives can be found in Supplementary Table 1.


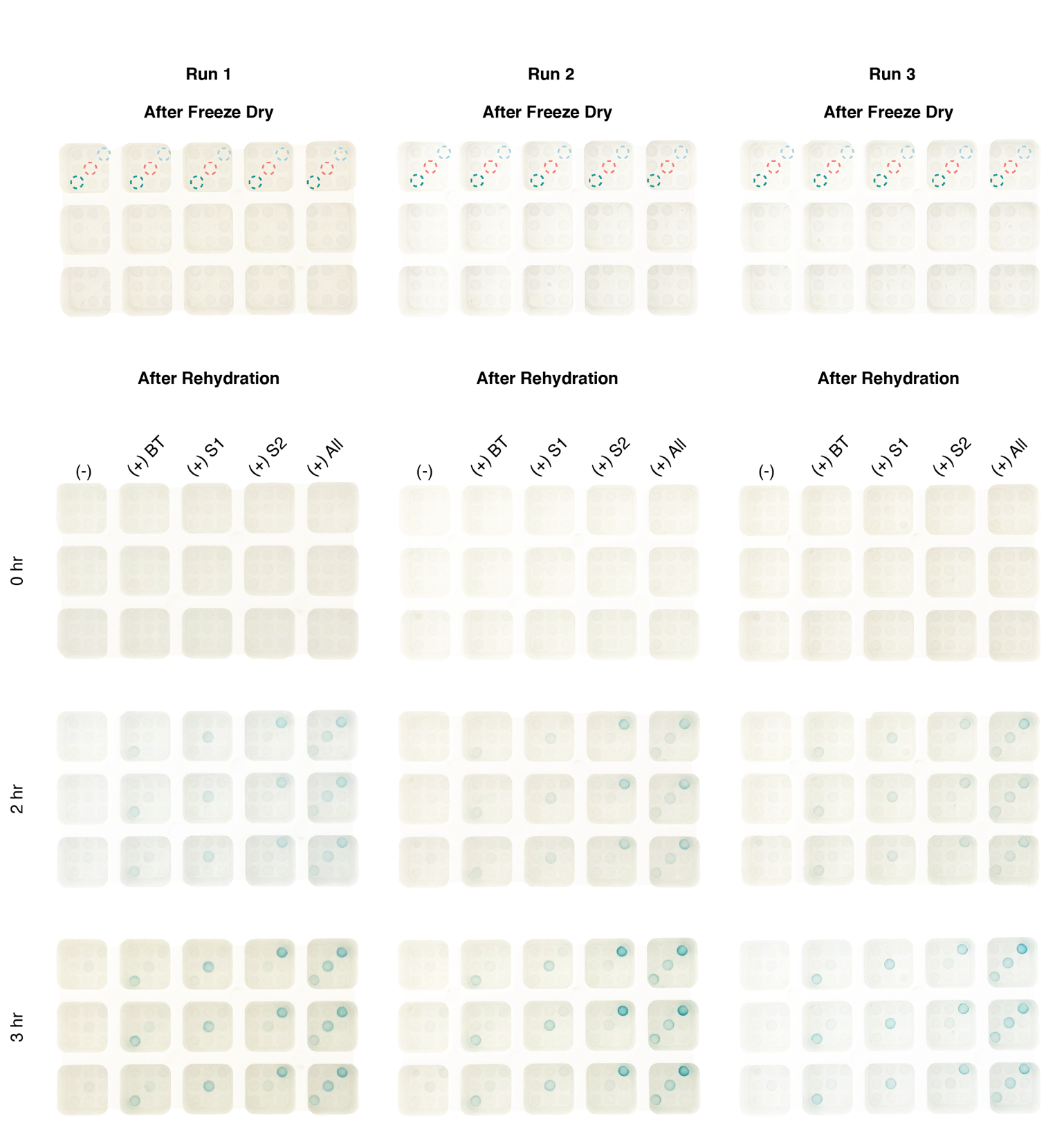


**Supplementary Fig. 17** Individual biological replicates for sensor activation and pigment production in freeze-dried and rehydrated reactions. Reactions were incubated at 37 ˚C for the specified amount of time indicated on the left of the figure. Each run is an independent experiment prepared and lyophilized on a different day. Photos for protocell sensor activation under different bulk phase conditions are provided for each biological replicate, each with technical triplicates. Teal, red, and blue circles indicate micro-basins with protocells containing *B. theta* (BT), Stx1 (S1), and Stx2 (S2) toehold switches, respectively. Targets added to the bulk phase for each condition are indicated above each image. Linear triggers used were amplified from bacterial genomic DNA and added at 50 nM for each condition. Each protocell sensor was only activated when its cognate inducer was present in the bulk phase, though with higher baseline activity of Stx1-switch at 3 hours. Details on CFE lysate used, plasmid concentrations, and reaction additives can be found in Supplementary Table 1.


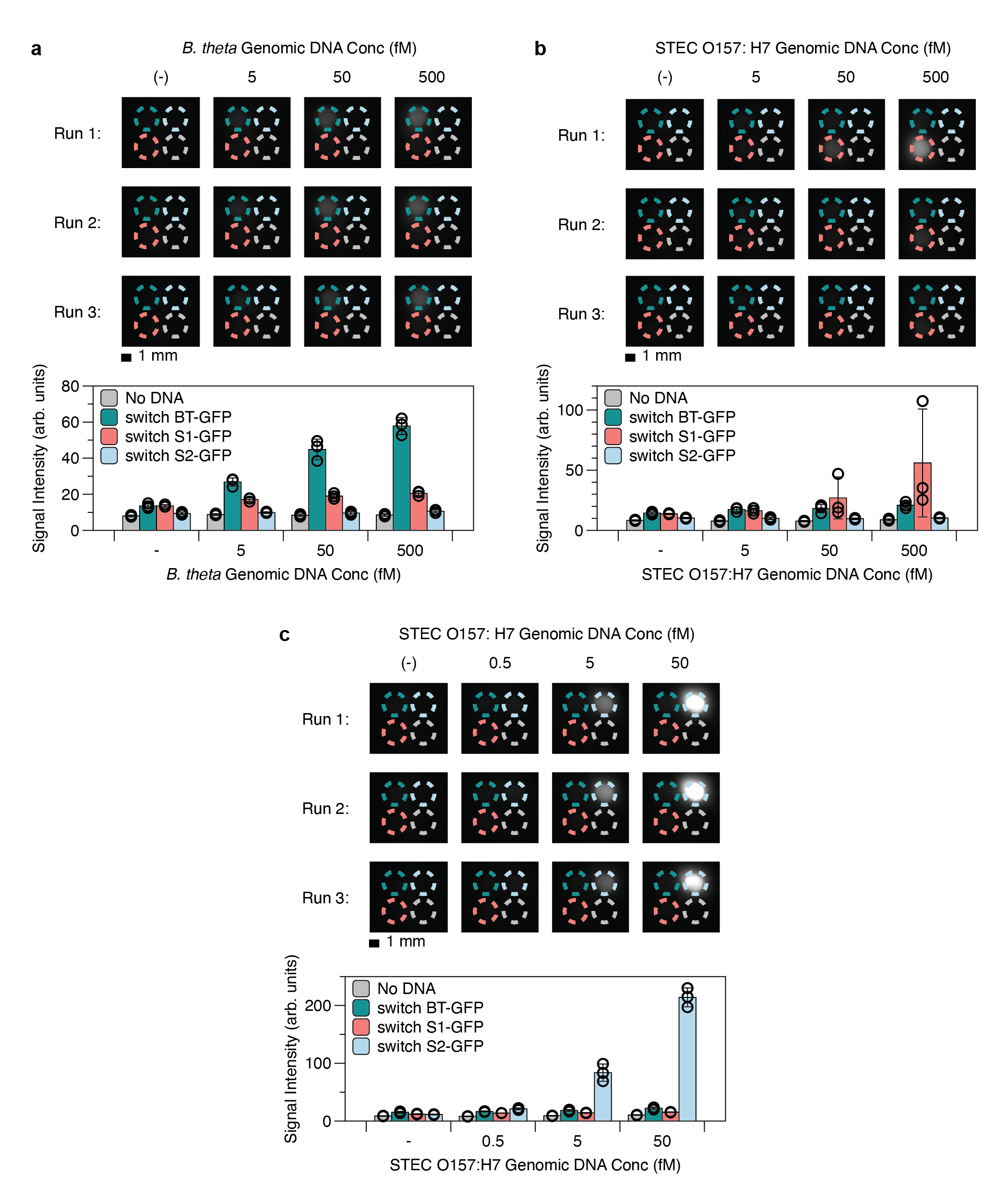


**Supplementary Fig. 18** Protocell arrays are compatible with the isothermal nucleic acid amplification technique. Recombinase polymerase amplification reaction kit (TwistAmp® Liquid Basic) was purchased from TwistDx. Briefly, 50 µL RPA reactions containing different concentrations of each bacteria’s genomic DNA were incubated with its corresponding primer pairs (Supplementary Table 2) at 37˚C for 40 minutes before adding the a solution containing 5% PEG and CFE energy mix to constitute the bulk phase. RPA reaction size and incubation times were chosen based on the manufacturer’s recommendation. Subsequent protocell reactions were assembled as described in Methods and incubated at 37 ˚C for 3 hours. In each subpanel, teal, red, and blue circles represent micro-basins containing *B. theta* (BT), Stx1 (S1), and Stx2 (S2) protocell sensors, respectively. The gray circle represents a CFE protocell without plasmid DNA. Each run represents a biological replicate assembled on a different day. Data are presented as mean values ± SD of 3 biological replicates, and hollow circles represent all data points. Scale bar is 1 mm. Details on CFE lysate used and plasmid concentrations can be found in Supplementary Table 1. **a** RPA-amplified *B. theta* trigger from *B. theta* genomic DNA showed observable activation in protocell arrays at femtomolar concentrations, demonstrating our platform’s robustness and compatibility with isothermal amplification reactions. **b** RPA-amplified Stx1 trigger from STEC O157:H7 genomic DNA showed observable activation in protocell array at femtomolar concentrations but exhibited higher run-to-run variabilities than other triggers amplified. **c** RPA-amplified Stx2 trigger from STEC O157:H7 genomic DNA showed observable activation at femtomolar concentrations. Compared to RPA amplified *B. theta* and Stx1 triggers, RPA-amplified Stx2 showed higher signal activation at lower genomic DNA concentrations. This is likely due to the longer annealing region used for the Stx2 primer (35 nts for forward and reverse primers) compared to the annealing regions of *B. theta* and Stx1 primers (<25 nts for forward and reverse primers)^8^. Simultaneous amplification and detection of *B. theta*, Stx1, and Stx2 triggers was not attempted due to the extensive primer pair and reaction condition optimization required.


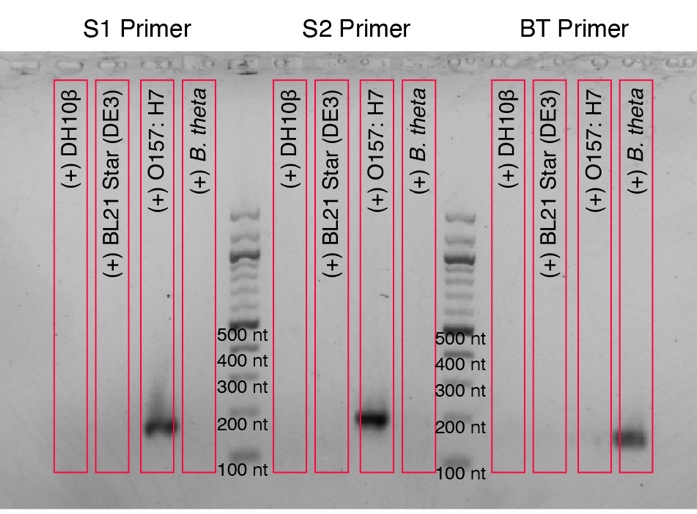


**Supplementary Fig. 19** Validation of target-specific trigger amplification. Primers were designed to amplify either Stx1 (S1), Stx2 (S2), or *B. theta* (BT) triggers. Target DNA was amplified when the appropriate template was added (STEC O157:H7 genomic DNA for Stx1 and Stx2, and *B. thetaiotaomicron* genomic DNA for *B. theta*). No amplification was observed on the genomic DNA of common lab *E. coli* strains DH10β or BL21 Star (DE3). PCR products were run on a 2% agarose gel. Data represented are from one experiment.

**Supplementary Fig. 20** Comparison of 𝜒DNA and GamS protein for their linear DNA protection capabilities. 𝜒DNA and GamS were added at 2 µM and 10 µM, respectively, to protect linear DNA trigger (100 nM) from nuclease degradation in lysate-based CFE reactions. Reactions were incubated at 37 ˚C for 3 hours. The fold change in the 𝜒DNA condition is higher: p = 1.24E-3 using a two-tailed Student’s t-test on 9 replicates (3 biological replicate x 3 technical replicates). Each biological replicate is an independently assembled reaction on a different day. Data are presented as mean values ± SD of 9 replicates. Solid-filled circles represent the mean of each biological replicate, and hollow circles represent all data points. Details on CFE lysate, plasmid concentration, and reaction additives are provided in Supplementary Table 1.


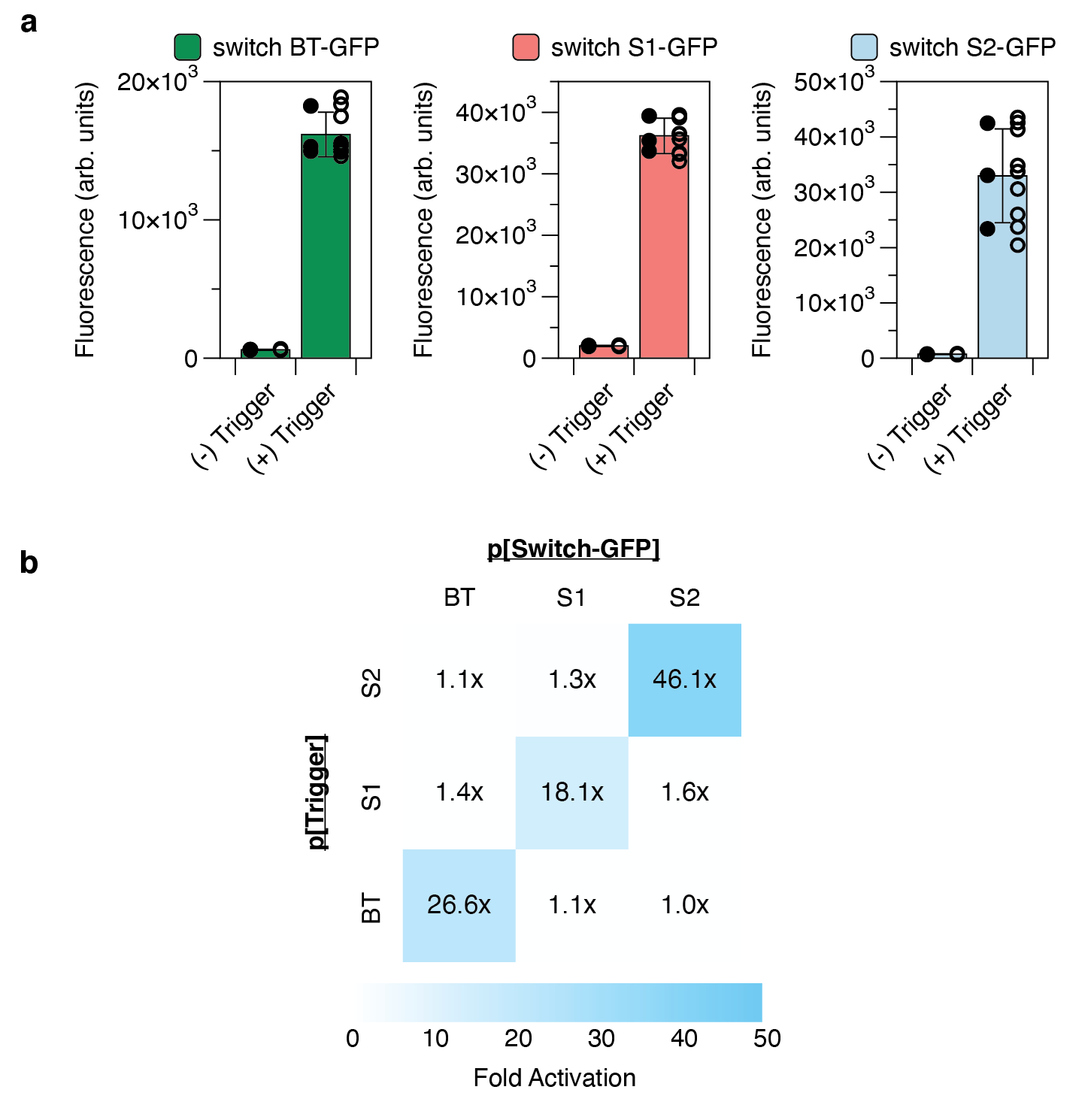


**Supplementary Fig. 21** Initial validation of *B. theta* (BT), Stx1 (S1), and Stx2 (S2) switch activation in response to their cognate triggers in CFE reactions. Reactions were incubated at 37 ˚C for 3 hours. **a** All triggers and switches were expressed from plasmids; see Supplementary Table 1 for plasmid concentrations used. Data are presented as mean values ± SD of 9 replicates (3 biological replicates x 3 technical replicates). Each biological replicate is an independently assembled reaction on a different day. Solid-filled circles represent the mean of each biological replicate, and hollow circles represent all data points. **b** Specificity assessment of previously developed *B. theta* toehold switch and newly developed Stx1 and Stx2 toehold switches. Only correctly paired triggers and switches showed high levels of GFP activation. Fold change shown represent average fold activation from 9 replicates (3 biological replicates x 3 technical replicates).

# **Supplementary Table 1** Description of lysates, plasmid concentrations, and reaction additives present in protocell arrays or CFE reactions in each figure.

| Figures | Detecting | Lysate Preparation | Plasmids | Reaction Additives |
| --- | --- | --- | --- | --- |
| Fig. 2,  Supplementary Fig. 2 |  | Uninduced  T7 RNAP  Batch U2 | 1.875 nM P_T7_-GFP |  |
| Fig. 3,  Supplementary Fig. 3 | IPTG | Uninduced  T7 RNAP  Batch U2 | 3.33 nM P_T7_-LacI  1 nM P_T7lacO_-GFP |  |
| Fig. 3,  Supplementary Fig. 3 | Arabinose | Uninduced  T7 RNAP  Batch U2 | 9.5 nM AraC-P_BAD_-GFP |  |
| Fig. 4c-d,  Supplementary Fig. 4, 6c | RNA B Trigger | Induced  T7 RNAP  Batch I2 | 7 nM P_T7_-switchB-GFP | 0.5 v/v% Rnase Inhibitor Murine in the bulk phase |
| Fig. 4c-d,  Supplementary Fig. 4, 6d | RNA H Trigger | Induced  T7 RNAP  Batch I2 | 7 nM P_T7_-switchH-GFP | 0.5 v/v% Rnase Inhibitor Murine in the bulk phase |
| Fig. 4e-f,  Supplementary Fig. 5, 7c | Linear DNA B Trigger | Induced  T7 RNAP  Batch I2 | 6 nM P_T7_-switchB-GFP | 2 µM 𝜒DNA in protocell |
| Fig. 4e-f,  Supplementary Fig. 5, 7d | Linear DNA H Trigger | Induced  T7 RNAP  Batch I2 | 6 nM P_T7_-switchH-GFP | 2 µM 𝜒DNA in protocell |
| Fig. 5b,  Supplementary Fig. 11 | Zinc | Uninduced  T7 RNAP  Batch U3 | 0.15 nM P_T7_-ZntR  2 nM P_ZntA_-GFP | 1.5 v/v% Rnase Inhibitor Murine in the bulk phase |
| Fig. 5b, Supplementary Fig. 11 | Vitamin B_12_ | Uninduced  T7 RNAP  Batch U3 | 1.5 nM P_T7_-EutR  7.5 nM P_EutS_-GFP | 1.5 v/v% Rnase Inhibitor Murine in the bulk phase |
| Fig. 5b,  Supplementary Fig. 11 | RNA Stx1 Trigger | Induced  T7 RNAP  Batch I1 | 4 nM P_T7_-Stx1 switch-GFP | 1.5 v/v% Rnase Inhibitor Murine in the bulk phase |
| Fig. 5b, Supplementary Fig. 11 | Linear DNA Stx2 Trigger | Induced  T7 RNAP  Batch I1 | 2.5 nM P_T7_-Stx2 switch-GFP | 1.5 v/v% Rnase Inhibitor Murine in the bulk phase  2 µM 𝜒DNA in protocell |
| Fig. 5c,  Supplementary Fig. 13 | Zinc | Uninduced  T7 RNAP  Batch U3 | 0.25 nM P_T7_-ZntR  5 nM P_ZntA_-GFP | 1.5 v/v% Rnase Inhibitor Murine in the bulk phase |
| Fig. 5c,  Supplementary Fig. 13 | Vitamin B_12_ | Uninduced  T7 RNAP  Batch U3 | 2.5 nM P_T7_-EutR  10 nM P_EutS_-GFP | 1.5 v/v% Rnase Inhibitor Murine in the bulk phase |
| Fig. 5c,  Supplementary Fig. 13 | RNA Stx1 Trigger | Induced  T7 RNAP  Batch I1 | 7.5 nM P_T7_-Stx1 switch-GFP | 1.5 v/v% Rnase Inhibitor Murine in the bulk phase |
| Fig. 5c,  Supplementary Fig. 13 | Linear DNA Stx2 Trigger | Induced  T7 RNAP  Batch I1 | 7.5 nM P_T7_-Stx2 switch-GFP | 1.5 v/v% Rnase Inhibitor Murine in the bulk phase  2 µM 𝜒DNA in protocell |
| Fig. 6b,  Supplementary Fig. 16 | Linear DNA  B. theta Trigger | Induced  T7 RNAP  Batch I1 | 1.5 nM P_T7_-B. theta switch-LacZ | 0.2 mg/mL X-gal in the bulk phase  2 µM 𝜒DNA in protocell |
| Fig. 6b,  Supplementary Fig. 16 | Linear DNA Stx1 Trigger | Induced  T7 RNAP  Batch I1 | 1.33 nM P_T7_-Stx1-LacZ | 0.2 mg/mL X-gal in the bulk phase  2 µM 𝜒DNA in protocell |
| Fig. 6b,  Supplementary Fig. 16 | Linear DNA Stx2 Trigger | Induced  T7 RNAP  Batch I1 | 1.33 nM P_T7_-Stx2-LacZ | 0.2 mg/mL X-gal in the bulk phase  2 µM 𝜒DNA in protocell |
| Fig. 6c,  Supplementary Fig. 17 | Linear DNA B. theta Trigger | Induced  T7 RNAP  Batch I1 | 4.5 nM P_T7_-B. theta switch-LacZ | 0.2 mg/mL X-gal in the bulk phase  2 µM 𝜒DNA in protocell |
| Fig. 6c,  Supplementary Fig. 17 | Linear DNA Stx1 Trigger | Induced  T7 RNAP  Batch I1 | 2.7 nM P_T7_-Stx1-LacZ | 0.2 mg/mL X-gal in the bulk phase  2 µM 𝜒DNA in protocell |
| Fig. 6c,  Supplementary Fig. 17 | Linear DNA Stx2 Trigger | Induced  T7 RNAP  Batch I1 | 3 nM P_T7_-Stx2-LacZ | 0.2 mg/mL X-gal in the bulk phase  2 µM 𝜒DNA in protocell |
| Supplementary Fig. 6a | RNA B Trigger | Induced  T7 RNAP  Batch I2 | 7 nM P_T7_-switchB-GFP | 0.5 v/v% Rnase Inhibitor Murine in CFE reaction |
| Supplementary Fig. 6b | RNA H Trigger | Induced  T7 RNAP  Batch I2 | 7 nM P_T7_-switchH-GFP | 0.5 v/v% Rnase Inhibitor Murine in CFE reaction |
| Supplementary Fig. 7a, 8a | Linear DNA B trigger | Induced  T7 RNAP  Batch I2 | 6 nM P_T7_-switchB-GFP | 2 µM 𝜒DNA in CFE reaction |
| Supplementary Fig. 7b, 8b | Linear DNA H Trigger | Induced  T7 RNAP  Batch I2 | 6 nM P_T7_-switchH-GFP | 2 µM 𝜒DNA in CFE reaction |
| Supplementary Fig. 9 | Zinc | Uninduced  T7 RNAP  Batch U1 | 0.25 nM P_T7_-ZntR  2.5 nM P_zntA_-GFP |  |
| Supplementary Fig. 9 | Vitamin B_12_ | Uninduced  T7 RNAP  Batch U1 | 2.5 nM P_T7_-EutR  10 nM P_eutS_-GFP |  |
| Supplementary Fig. 10 | Linear DNA  B. theta Trigger | Induced  T7 RNAP  Batch I1 | 5 nM P_T7_-B. theta switch-GFP | 2 µM 𝜒DNA in protocell  0.5 v/v% Rnase Inhibitor Murine in the bulk phase |
| Supplementary Fig. 10 | Linear DNA Stx1 Trigger | Induced  T7 RNAP  Batch I1 | 4 nM P_T7_-Stx1-GFP | 10 µM GamS in protocell  0.5 v/v% Rnase Inhibitor Murine in the bulk phase |
| Supplementary Fig. 10 | Linear DNA Stx2 Trigger | Induced  T7 RNAP  Batch I1 | 5 nM P_T7_-Stx2-GFP | 2 µM 𝜒DNA in protocell  0.5 v/v% Rnase Inhibitor Murine in the bulk phase |
| Supplementary Fig. 12 | Zinc | Uninduced  T7 RNAP  Batch U3 | 0.2 nM P_T7_-ZntR  2.75 nM P_zntA_-GFP | 1.5 v/v% Rnase Inhibitor Murine in the bulk phase |
| Supplementary Fig. 12 | Vitamin B_12_ | Uninduced  T7 RNAP  Batch U3 | 2 nM P_T7_-EutR  8 nM P_eutS_-GFP | 1.5 v/v% Rnase Inhibitor Murine in the bulk phase |
| Supplementary Fig. 12 | RNA Stx1 Trigger | Induced  T7 RNAP  Batch I1 | 6 nM P_T7_-Stx1 switch-GFP | 1.5 v/v% Rnase Inhibitor Murine in the bulk phase |
| Supplementary Fig. 12 | Linear DNA Stx2 Trigger | Induced  T7 RNAP  Batch I1 | 5 nM P_T7_-Stx2 switch-GFP | 1.5 v/v% Rnase Inhibitor Murine in the bulk phase  2 µM 𝜒DNA in protocell |
| Supplementary Fig. 15 | Cleave CPRG | Uninduced  T7 RNAP  Batch U1 | 2 nM P_T7_-LacZ | 0.6 mg/mL CPRG in the bulk phase |
| Supplementary Fig. 15 | Cleave X-gal | Uninduced  T7 RNAP  Batch U1 | 2 nM P_T7_-LacZ | 0.2 mg/mL X-gal in the bulk phase |
| Supplementary Fig. 18 | Linear DNA B. theta Trigger | Induced  T7 RNAP  Batch I2 | 5 nM P_T7_-B. theta switch-GFP | 25% RPA (50 µL in 200 µL)  2 µM 𝜒DNA in protocell |
| Supplementary Fig. 18 | Linear DNA Stx1 Trigger | Induced  T7 RNAP  Batch I2 | 4 nM P_T7_-Stx1-GFP | 25% RPA (50 µL in 200 µL)  2 µM 𝜒DNA in protocell |
| Supplementary Fig. 18 | Linear DNA Stx2 Trigger | Induced  T7 RNAP  Batch I2 | 5 nM P_T7_-Stx2-GFP | 25% RPA (50 µL in 200 µL)  2 µM 𝜒DNA in protocell |
| Supplementary Fig. 20 | Linear DNA Stx1 Trigger | Induced  T7 RNAP  Batch I2 | 2.5 nM P_T7_-Stx1 switch-GFP |  |
| Supplementary Fig. 20 | Linear DNA Stx1 Trigger | Induced  T7 RNAP  Batch I2 | 2.5 nM P_T7_-Stx1 switch-GFP | 2 µM 𝜒DNA in CFE reaction |
| Supplementary Fig. 20 | Linear DNA Stx1 Trigger | Induced  T7 RNAP  Batch I2 | 2.5 nM P_T7_-Stx1 switch-GFP | 10 µM GamS in CFE reaction |
| Supplementary Fig. 21 | Plasmid  B. theta Trigger | Induced  T7 RNAP  Batch I2 | 5 nM P_T7_-B. theta trigger  2.5 nM P_T7_-B. theta switch-GFP |  |
| Supplementary Fig. 21 | Plasmid Stx1 Trigger | Induced  T7 RNAP  Batch I2 | 5 nM P_T7_-Stx1 trigger  2.5 nM P_T7_-Stx1 switch-GFP |  |
| Supplementary Fig. 21 | Plasmid Stx2 Trigger | Induced  T7 RNAP  Batch I2 | 5 nM P_T7_-Stx2 trigger  2.5 nM P_T7_-Stx2 switch-GFP |  |

**Supplementary Table 2**. Primers used for trigger DNA amplification. Lowercase, unlabeled sequences are protective regions to decrease endonuclease degradation. Highlighted sequences indicate the T7 promoter, and uppercase sequences are primer annealing regions. Target-specific primers were designed to bind to >19 base pairs before and after the actual trigger sequence to prevent unintended primer activation of switches. The specificity of the developed switches was also validated on the genomic DNA of STEC O157: H7, *B. thetaiotaomicron*, and common laboratory *E. coli* strains DH10β and BL21 Star (DE3) (Supplementary Fig. 20).

| Amplifying | Primer Sequence |
| --- | --- |
| B or H | Fwd:  aacgccagcaacgcgatcccgcgaaatTAATACGACTCACTATAGGGAGA  Rev:  taatcagaattggctttcagcaaaAAACCCCTCAAGACCCGTT  Template: T7-triggerB or T7-triggerH in Sequence Information file included in the Source Data folder. |
| *B. theta* | Fwd:  ggaaaaacgccagcaacgcgatcccgcgaaattaatacgactcactataggCCGACTTCGGAACGCTTATAGA  Rev:  caaacgccgccgaaaggcggcgttttttttTGAAACGTATGCGGTAGCTGAA  Genomic Target: hypothetical protein SAMN029103 22_01913  ATGCATGCATACATTATCCAACAACTAACAAGAATTATATTGTTTATCACTATCGGTTTGCCTATAGGACTAAAAAGTTTTGCCCAAGAAACAAAACGTTTCTATATGGAACTGGACACTCCCCGCAATGGAGCCAAAGCAGGACAAGAGCTTGAATTAAAATACATCAGCACAGCCGATTTCGATTCTGTATCTCCACCCGACTTCGGAACGCTTATAGAAACAGTTGAAGGAGCAACACCACACAAAGCCGGTCATACAGTAAAAAACGGCATATTGACAGATATCTACGAGCAGGGATTCAGCTACCGCATACGTTTCAAGAAGCCAGGAAACACCAAACTACCTCTGGCATCCATCAAGGCAAACGGAAAGGAATACGAAACACCTCTGACCAGTGTATGGGTACATCCGGTCGATACCAATATCGACAGTGTAAAATGCAGCATTCAGCTGGAGGATTCTTATCGCAAAGGAGTTTTCACTGCCATCGGGATCTGTCTCTTAATCGCCTGGTTATTGATCCGCTTATCGTTTCAGAAACAAAAAAATAAAGAGACAGGATAA |
| Stx1 | Fwd:  ggaaaaacgccagcaacgcgatcccgcgaaattaatacgactcactataggATAAATCGCCATTCGTTGACTACT  Rev:  caaacgccgccgaaaggcggcgttttttttAAACCGTAACATCGCTCTTGCCA  Genomic Target: Shiga Toxin 1  Accession #: BA000007. Region: 2924904...2925851.  ATGAAAATAATTATTTTTAGAGTGCTAACTTTTTTCTTTGTTATCTTTTCAGTTAATGTGGTTGCGAAGGAATTTACCTTAGACTTCTCGACTGCAAAGACGTATGTAGATTCGCTGAATGTCATTCGCTCTGCAATAGGTACTCCATTACAGACTATTTCATCAGGAGGTACGTCTTTACTGATGATTGATAGTGGCACAGGGGATAATTTGTTTGCAGTTGATGTCAGAGGGATAGATCCAGAGGAAGGGCGGTTTAATAATCTACGGCTTATTGTTGAACGAAATAATTTATATGTGACAGGATTTGTTAACAGGACAAATAATGTTTTTTATCGCTTTGCTGATTTTTCACATGTTACCTTTCCAGGTACAACAGCGGTTACATTGTCTGGTGACAGTAGCTATACCACGTTACAGCGTGTTGCAGGGATCAGTCGTACGGGGATGCAGATAAATCGCCATTCGTTGACTACTTCTTATCTGGATTTAATGTCGCATAGTGGAACCTCACTGACGCAGTCTGTGGCAAGAGCGATGTTACGGTTTGTTACTGTGACAGCTGAAGCTTTACGTTTTCGGCAAATACAGAGGGGATTTCGTACAACACTGGATGATCTCAGTGGGCGTTCTTATGTAATGACTGCTGAAGATGTTGATCTTACATTGAACTGGGGAAGGTTGAGTAGTGTCCTGCCTGATTATCATGGACAAGACTCTGTTCGTGTAGGAAGAATTTCTTTTGGAAGCATTAATGCAATTCTGGGAAGCGTGGCATTAATACTGAATTGTCATCATCATGCATCGCGAGTTGCCAGAATGGCATCTGATGAGTTTCCTTCTATGTGTCCGGCAGATGGAAGAGTCCGTGGGATTACGCACAATAAAATATTGTGGGATTCATCCACTCTGGGGGCAATTCTGATGCGCAGAACTATTAGCAGTTG |
| Stx2 | Fwd:  ggaaaaacgccagcaacgcgatcccgcgaaattaatacgactcactataggGTATCCTATTCCCGGGAGTTTACGATAGACTTTTC  Rev:  caaacgccgccgaaaggcggcgttttttttGATATATGTTCAAGAGGGGTCGATATCTCTGTCCG  Genomic Target: Shiga Toxin 2  Accession #: BA000007. Region: 1267107…1268066.  ATGAAGTGTATATTATTTAAATGGGTACTGTGCCTGTTACTGGGTTTTTCTTCGGTATCCTATTCCCGGGAGTTTACGATAGACTTTTCGACCCAACAAAGTTATGTCTCTTCGTTAAATAGTATACGGACAGAGATATCGACCCCTCTTGAACATATATCTCAGGGGACCACATCGGTGTCTGTTATTAACCACACCCCACCGGGCAGTTATTTTGCTGTGGATATACGAGGGCTTGATGTCTATCAGGCGCGTTTTGACCATCTTCGTCTGATTATTGAGCAAAATAATTTATATGTGGCCGGGTTCGTTAATACGGCAACAAATACTTTCTACCGTTTTTCAGATTTTACACATATATCAGTGCCCGGTGTGACAACGGTTTCCATGACAACGGACAGCAGTTATACCACTCTGCAACGTGTCGCAGCGCTGGAACGTTCCGGAATGCAAATCAGTCGTCACTCACTGGTTTCATCATATCTGGCGTTAATGGAGTTCAGTGGTAATACAATGACCAGAGATGCATCCAGAGCAGTTCTGCGTTTTGTCACTGTCACAGCAGAAGCCTTACGCTTCAGGCAGATACAGAGAGAATTTCGTCAGGCACTGTCTGAAACTGCTCCTGTGTATACGATGACGCCGGGAGACGTGGACCTCACTCTGAACTGGGGGCGAATCAGCAATGTGCTTCCGGAGTATCGGGGAGAGGATGGTGTCAGAGTGGGGAGAATATCCTTTAATAATATATCAGCGATACTGGGGACTGTGGCCGTTATACTGAATTGCCATCATCAGGGGGCGCGTTCTGTTCGCGCCGTGAATGAAGAGAGTCAACCAGAATGTCAGATAACTGGCGACAGGCCCGTTATAAAAATAAACAATACATTATGGGAAAGTAATACAGCTGCAGCGTTTCTGAACAGAAAGTCACAGTTTTTATATACAACGGGTAAATAAAGGAGTTAAGCATGAAGAAGATGTTTATGGCGGTTTTATTTGCATTAGCTTCTGTTAATGCAATGGCGGCGGATTGTGCTAAAGGTAAAATTGAGTTTTCCAAGTATAATGAGGATGACACATTTACAGTGAAGGTTGACGGGAAAGAATACTGGACCAGTCGCTGGAATCTGCAACCGTTACTGCAAAGTGCTCAGTTGACAGGAATGACTGTCACAATCAAATCCAGTACCTGTGAATCAGGCTCCGGATTTGCTGAAGTGCAGTTTAATAATGACTGA |

**References**

1 Frampton, J. P. *et al.* Aqueous two-phase system patterning of detection antibody solutions for cross-reaction-free multiplex ELISA. *Sci Rep* 4, 4878, doi:10.1038/srep04878 (2014).

2 Simon, A. B. *et al.* Aqueous two-phase systems enable multiplexing of homogeneous immunoassays. *Technology (Singap World Sci)* 2, 176, doi:10.1142/S2339547814500150 (2014).

3 Tongdee, M. *et al.* One-incubation one-hour multiplex ELISA enabled by aqueous two-phase systems. *Analyst* 145, 3517-3527, doi:10.1039/d0an00383b (2020).

4 Eiden, L., Yamanishi, C., Takayama, S. & Dishinger, J. F. Aqueous Two-Phase System Rehydration of Antibody–Polymer Microarrays Enables Convenient Compartmentalized Multiplex Immunoassays. *Analytical Chemistry* 88, 11328-11334, doi:10.1021/acs.analchem.6b02960 (2016).

5 Jewett, M. C. & Swartz, J. R. Mimicking the Escherichia coli cytoplasmic environment activates long-lived and efficient cell-free protein synthesis. *Biotechnol Bioeng* 86, 19-26, doi:10.1002/bit.20026 (2004).

6 Takahashi, M. K. *et al.* A low-cost paper-based synthetic biology platform for analyzing gut microbiota and host biomarkers. *Nat Commun* 9, 3347, doi:10.1038/s41467-018-05864-4 (2018).

7 McNerney, M. P. *et al.* Point-of-care biomarker quantification enabled by sample-specific calibration. *Sci Adv* 5, eaax4473, doi:10.1126/sciadv.aax4473 (2019).

8 Lobato, I. M. & O'Sullivan, C. K. Recombinase polymerase amplification: Basics, applications and recent advances. *Trac-Trend Anal Chem* 98, 19-35, doi:10.1016/j.trac.2017.10.015 (2018).
