# Supplementary material for: Protocell arrays for simultaneous detection of diverse analytes
Source: Nat Commun. 2021 Sep 29;12:5724. doi: 10.1038/s41467-021-25989-3 (PMC8481512; doi:10.1038/s41467-021-25989-3)
Supplement: Supplementary file 3 — Reporting Summary [file 41467_2021_25989_MOESM3_ESM.pdf]

nature portfolio

Corresponding author(s): Shuichi Takayama, Mark P. Styczynski

Last updated by author(s): Aug 31, 2021

Reporting Summary

Nature Portfolio wishes to improve the reproducibility of the work that we publish. This form provides structure for consistency and transparency in reporting. For further information on Nature Portfolio policies, see our Editorial Policies and the Editorial Policy Checklist.

Statistics

For all statistical analyses, confirm that the following items are present in the figure legend, table legend, main text, or Methods section.

n/a	Confirmed	
☐	☒	The exact sample size (*n*) for each experimental group/condition, given as a discrete number and unit of measurement
☐	☒	A statement on whether measurements were taken from distinct samples or whether the same sample was measured repeatedly
☐	☒	The statistical test(s) used AND whether they are one- or two-sided<br>*Only common tests should be described solely by name; describe more complex techniques in the Methods section.*
☒	☐	A description of all covariates tested
☒	☐	A description of any assumptions or corrections, such as tests of normality and adjustment for multiple comparisons
☐	☒	A full description of the statistical parameters including central tendency (e.g. means) or other basic estimates (e.g. regression coefficient) AND variation (e.g. standard deviation) or associated estimates of uncertainty (e.g. confidence intervals)
☐	☒	For null hypothesis testing, the test statistic (e.g. *F*, *t*, *r*) with confidence intervals, effect sizes, degrees of freedom and *P* value noted<br>*Give P values as exact values whenever suitable.*
☒	☐	For Bayesian analysis, information on the choice of priors and Markov chain Monte Carlo settings
☒	☐	For hierarchical and complex designs, identification of the appropriate level for tests and full reporting of outcomes
☒	☐	Estimates of effect sizes (e.g. Cohen's *d*, Pearson's *r*), indicating how they were calculated

Our web collection on statistics for biologists contains articles on many of the points above.

Software and code

Policy information about availability of computer code

Data collection	Image Lab Software vS.2.1 was used to take custom-made microplate images from the ChemiDoc MP imaging system (BIO-RAD), Gens Software v2.07.17 was used to collect 384-well plate reader data from the BioTek Synergy H4 Microplate Reader (BioTek).
Data analysis	Fiji Software v2.1.0/1.53c was used for obtaining pixel intensities from custom-made microplate images, Microsoft Excel (for Mac) v16.52 was used for data analysis.

For manuscripts utilizing custom algorithms or software that are central to the research but not yet described in published literature, software must be made available to editors and reviewers. We strongly encourage code deposition in a community repository (e.g. GitHub). See the Nature Portfolio guidelines for submitting code & software for further information.

Data

Policy information about availability of data

All manuscripts must include a data availability statement. This statement should provide the following information, where applicable:

- Accession codes, unique identifiers, or web links for publicly available datasets
- A description of any restrictions on data availability
- For clinical datasets or third party data, please ensure that the statement adheres to our policy

Source data for all main text and supplementary figures are available in the Source Data folder provided with this manuscript. Sensor sequences used are available in the Sequence Information file inside the Source Data folder provided with this manuscript. Engineering drawings of the 96- and 32-well plates used in this study are provided in the Source Data folder.

Field-specific reporting

Please select the one below that is the best fit for your research. If you are not sure, read the appropriate sections before making your selection.

☒ Life sciences ☐ Behavioural & social sciences ☐ Ecological, evolutionary & environmental sciences

For a reference copy of the document with all sections, see nature.com/documents/nr-reporting-summary-flat.pdf

Life sciences study design

All studies must disclose on these points even when the disclosure is negative.

Sample size	All reported experiments (with the exception of Supplementary Fig. 9, 10, 14, 15, 19) are the average of at least 3 experimental replicates, each experiment replicate has at least 3 technical replicates. Supplementary Fig. 9, 10, and 15 have only technical triplicates: these figures were used only for sensor tuning (9 and 10) or new reporter characterization (15), and in these figures the biological replicates of sensor and substrate behaviors have been replicated in other figures. The choice of sample size used for fluorescent and colorimetric analyses was based on published articles to give a approximate sense of the variability in the data (10.1038/s41467-020-20639-6, 10.1038/s41467-019-13454-1, and 10.1038/s41467-018-05864-4)<br><br>Free protocells (not confined to micro plates) and gel characterizations of strain-specific amplification presented in Supplementary Fig. 2a and 19 came from one experiment, as they give visual, qualitative data.<br>Supplementary Fig. 14 present successful zinc removal and ICP-MS confirmation on one batch of human serum used throughout the manuscript.
Data exclusions	No data was excluded from the analyses.
Replication	All experiments have been independently replicated at least 3 times successfully. All attempts at replication were successful.
Randomization	Randomization was not relevant to this study. Samples were not allocated into groups and instead all processed simultaneously.
Blinding	Blinding was not relevant to this study because samples were not allocated into groups.

Reporting for specific materials, systems and methods

We require information from authors about some types of materials, experimental systems and methods used in many studies. Here, indicate whether each material, system or method listed is relevant to your study. If you are not sure if a list item applies to your research, read the appropriate section before selecting a response.

Materials & experimental systems

n/a	Involved in the study	
☒	☐	Antibodies
☒	☐	Eukaryotic cell lines
☒	☐	Palaeontology and archaeology
☒	☐	Animals and other organisms
☒	☐	Human research participants
☒	☐	Clinical data
☒	☐	Dual use research of concern

Methods

n/a	Involved in the study	
☒	☐	ChIP-seq
☒	☐	Flow cytometry
☒	☐	MRI-based neuroimaging
