# Supplementary material for: Protocell arrays for simultaneous detection of diverse analytes
Source: Nat Commun. 2021 Sep 29;12:5724. doi: 10.1038/s41467-021-25989-3 (PMC8481512; doi:10.1038/s41467-021-25989-3)
Supplement: Supplementary file 4 — Source Data [file 41467_2021_25989_MOESM4_ESM.zip › Sequence Information.pdf]

Sequence Information for

Protocell arrays for simultaneous detection of diverse analytes

Table of Contents

pJL1 2
P_{T7}-lacI 2
P_{T7lacO}-sfgfp 3
araC-P_{BAD}-sfgfp 3
P_{T7}-trigger B 4
P_{T7}-switch B-sfgfp 4
P_{T7}-trigger H 5
P_{T7}-switch H-sfgfp 5
P_{T7}-zntR 5
P_{ZntA}-sfgfp 6
P_{T7}-eutR 6
P_{EutS}-sfgfp 7
P_{T7}-trigger BT 8
P_{T7}-switch BT-sfgfp 8
P_{T7}-trigger S1 8
P_{T7}-switch S1-sfgfp 9
P_{T7}-trigger S2 9
P_{T7}-switch S2-sfgfp 9
P_{T7}-lacZ 10
χDNA 11
P_{T7}-Null 11
P_{T7}-trigger EC 11

Description of plasmid parts and DNA sequences in this paper:

<table>
<tr><th>Name</th><th>Construct Description</th></tr>
<tr><td>pJL1</td><td>Relevant part of the plasmid encoding superfolder GFP expression under P_{T7} promoter with strong ribosomal binding site</td></tr>
<tr><td colspan="2">P_{T7} Promoter-Stability Hairpin-StrongRBS-sfgfp-T7 Terminator

taatacgactcactatagggagaccacaacggtttccctctagaaataattttgtttaactttaagaaggagatatacatATGAGC
AAAGGTGAAGAACTGTTTACCGGCGTTGTGCCGATTCTGGTGGAACTGGATGGCGATGT
GAACGGTCACAAATTCAGCGTGCGTGGTGAAGGTGAAGGCGATGCCACGATTGGCAAA
CTGACGCTGAAATTTATCTGCACCACCGGCAAACTGCCGGTGCCGTGGCCGACGCTGG
TGACCACCCTGACCTATGGCGTTCAGTGTTTTAGTCGCTATCCGGATCACATGAAACGTC
ACGATTTCTTTAAATCTGCAATGCCGGAAGGCTATGTGCAGGAACGTACGATTAGCTTTA
AAGATGATGGCAAATATAAAACGCGCGCCGTTGTGAAATTTGAAGGCGATACCCTGGTG
AACCGCATTGAACTGAAAGGCACGGATTTTAAAGAAGATGGCAATATCCTGGGCCATAAA
CTGGAATACAACTTTAATAGCCATAATGTTTATATTACGGCGGATAAACAGAAAAATGGCA
TCAAAGCGAATTTTACCGTTCGCCATAACGTTGAAGATGGCAGTGTGCAGCTGGCAGAT
CATTATCAGCAGAATACCCCGATTGGTGATGGTCCGGTGCTGCTGCCGGATAATCATTAT
CTGAGCACGCAGACCGTTCTGTCTAAAGATCCGAACGAAAAAGGCACGCGGGACCACAT
GGTTCTGCACGAATATGTGAATGCGGCAGGTATTACGTGGAGCCATCCGCAGTTCGAAA
AATAAgtcgaccggctgctaacaaagcccgaaaggaagctgagttggctgctgccaccgctgagcaataactagcataaccc
cttggggcctctaaacgggtcttgaggggttttttg</td></tr>
</table>

<table>
<tr><td>P_{T7}-lacI</td><td>Relevant part of the plasmid encoding LacI expression under P_{T7} promoter with strong ribosomal binding site</td></tr>
<tr><td colspan="2">P_{T7} Promoter-Stability Hairpin-StrongRBS-lacI-T7 Terminator

taatacgactcactatagggagaccacaacggtttccctctagaaataattttgtttaactttaagaaggagatatacatATGAAA
CCAGTAACGTTATACGATGTCGCAGAGTATGCCGGTGTCTCTTATCAGACCGTTTCCCG
CGTGGTGAACCAGGCCAGCCACGTTTCTGCGAAAACGCGGGAAAAAGTGGAAGCGGCG
ATGGCGGAGCTGAATTACATTCCCAACCGCGTGGCACAACAACTGGCGGGCAAACAGT
CGTTGCTGATTGGCGTTGCCACCTCCAGTCTGGCCCTGCACGCGCCGTCGCAAATTGTC
GCGGCGATTAAATCTCGCGCCGATCAACTGGGTGCCAGCGTGGTGGTGTCGATGGTAG
AACGAAGCGGCGTCGAAGCCTGTAAAGCGGCGGTGCACAATCTTCTCGCGCAACGCGT
CAGTGGGCTGATCATTAACTATCCGCTGGATGACCAGGATGCCATTGCTGTGGAAGCTG
CCTGCACTAATGTTCCGGCGTTATTTCTTGATGTCTCTGACCAGACACCCATCAACAGTA
TTATTTTCTCCCATGAAGACGGTACGCGACTGGGCGTGGAGCATCTGGTCGCATTGGGT
CACCAGCAAATCGCGCTGTTAGCGGGCCCATTAAGTTCTGTCTCGGCGCGTCTGCGTCT
GGCTGGCTGGCATAAATATCTCACTCGCAATCAAATTCAGCCGATAGCGGAACGGGAAG
GCGACTGGAGTGCCATGTCCGGTTTTCAACAAACCATGCAAATGCTGAATGAGGGCATC
GTTCCCACTGCGATGCTGGTTGCCAACGATCAGATGGCGCTGGGCGCAATGCGCGCCA
TTACCGAGTCCGGGCTGCGCGTTGGTGCGGATATCTCGGTAGTGGGATACGACGATAC
CGAAGACAGCTCATGTTATATCCCGCCGTTAACCACCATCAAACAGGATTTTCGCCTGCT
GGGGCAAACCAGCGTGGACCGCTTGCTGCAACTCTCTCAGGGCCAGGCGGTGAAGGG
CAATCAGCTGTTGCCCGTCTCACTGGTGAAAAGAAAAACCACCCTGGCGCCCAATACGC
AAACCGCCTCTCCCCGCGCGTTGGCCGATTCATTAATGCAGCTGGCACGACAGGTTTCC</td></tr>
</table>

CGACTGGAAAGCGGGCAGTGATAAgtcgaccggctgctaacaaagcccgaaaggaagctgagttggctgctgc<br>caccgctgagcaataactagcataaccccttggggcctctaaacgggtcttgaggggttttttg

**P_{T7lacO}-*sfgfp***	Relevant part of the plasmid encoding superfolder GFP expression under P_{T7lacO} promoter with strong ribosomal binding site
P_{T7} Promoter-lacO-Stability Hairpin-StrongRBS-*sfgfp*- T7 Terminator<br><br>taatacgactcactatagggagatgtgagcggataacaaccacaacggtttccctctagaaataattttgtttaactttaagaaggag<br>atatacatATGAGCAAAGGTGAAGAACTGTTTACCGGCGTTGTGCCGATTCTGGTGGAACTG<br>GATGGCGATGTGAACGGTCACAAATTCAGCGTGCGTGGTGAAGGTGAAGGCGATGCCA<br>CGATTGGCAAACTGACGCTGAAATTTATCTGCACCACCGGCAAACTGCCGGTGCCGTGG<br>CCGACGCTGGTGACCACCCTGACCTATGGCGTTCAGTGTTTTAGTCGCTATCCGGATCA<br>CATGAAACGTCACGATTTCTTTAAATCTGCAATGCCGGAAGGCTATGTGCAGGAACGTAC<br>GATTAGCTTTAAAGATGATGGCAAATATAAAACGCGCGCCGTTGTGAAATTTGAAGGCGA<br>TACCCTGGTGAACCGCATTGAACTGAAAGGCACGGATTTTAAAGAAGATGGCAATATCCT<br>GGGCCATAAACTGGAATACAACTTTAATAGCCATAATGTTTATATTACGGCGGATAAACA<br>GAAAAATGGCATCAAAGCGAATTTTACCGTTCGCCATAACGTTGAAGATGGCAGTGTGCA<br>GCTGGCAGATCATTATCAGCAGAATACCCCGATTGGTGATGGTCCGGTGCTGCTGCCGG<br>ATAATCATTATCTGAGCACGCAGACCGTTCTGTCTAAAGATCCGAACGAAAAAGGCACGC<br>GGGACCACATGGTTCTGCACGAATATGTGAATGCGGCAGGTATTACGTGGAGCCATCCG<br>CAGTTCGAAAAATAAgtcgaccggctgctaacaaagcccgaaaggaagctgagttggctgctgccaccgctgagcaat<br>aactagcataaccccttggggcctctaaacgggtcttgaggggttttttg	

araC*-P_{BAD}-*sfgfp	Relevant part of the plasmid with divergent P_{BAD} promoter for constitutive AraC expression and arabinose modulated superfolder GFP expression.
araC-P_{BAD}-Stability Hairpin-StrongRBS-*sfgfp*- T7 Terminator<br><br>TTATGACAACTTGACGGCTACATCATTCACTTTTTCTTCACAACCGGCACGGAACTCGCT<br>CGGGCTGGCCCCGGTGCATTTTTTAAATACCCGCGAGAAATAGAGTTGATCGTCAAAAC<br>CAACATTGCGACCGACGGTGGCGATAGGCATCCGGGTGGTGCTCAAAAGCAGCTTCGC<br>CTGGCTGATACGTTGGTCCTCGCGCCAGCTTAAGACGCTAATCCCTAACTGCTGGCGGA<br>AAAGATGTGACAGACGCGACGGCGACAAGCAAACATGCTGTGCGACGCTGGCGATATC<br>AAAATTGCTGTCTGCCAGGTGATCGCTGATGTACTGACAAGCCTCGCGTACCCGATTAT<br>CCATCGGTGGATGGAGCGACTCGTTAATCGCTTCCATGCGCCGCAGTAACAATTGCTCA<br>AGCAGATTTATCGCCAGCAGCTCCGAATAGCGCCCTTCCCCTTGCCCGGCGTTAATGAT<br>TTGCCCAAACAGGTCGCTGAAATGCGGCTGGTGCGCTTCATCCGGGCGAAAGAACCCC<br>GTATTGGCAAATATTGACGGCCAGTTAAGCCATTCATGCCAGTAGGCGCGCGGACGAAA<br>GTAAACCCACTGGTGATACCATTCGCGAGCCTCCGGATGACGACCGTAGTGATGAATCT<br>CTCCTGGCGGGAACAGCAAAATATCACCCGGTCGGCAAACAAATTCTCGTCCCTGATTT<br>TTCACCACCCCCTGACCGCGAATGGTGAGATTGAGAATATAACCTTTCATTCCCAGCGGT<br>CGGTCGATAAAAAAATCGAGATAACCGTTGGCCTCAATCGGCGTTAAACCCGCCACCAG<br>ATGGGCATTAAACGAGTATCCCGGCAGCAGGGGATCATTTTGCGCTTCAGCCATacttttcat<br>actcccgccattcagagaagaaaccaattgtccatattgcatcagacattgccgtcactgcgtcttttactggctcttctcgctaacca<br>aaccggtaaccccgcttattaaaagcattctgtaacaaagcgggaccaaagccatgacaaaaacgcgtaacaaaagtgtctata<br>atcacggcagaaaagtccacattgattatttgcacggcgtcacactttgctatgccatagcatttttatccataagattagcggatccta<br>cctgacgctttttatcgcaactctctactgtttctccatacccgtttttttgggctagcccacaacggtttccctctagaaataattttgtttaa	

ctttaagaaggagatatacatATGAGCAAAGGTGAAGAACTGTTTACCGGCGTTGTGCCGATTCTG
GTGGAACTGGATGGCGATGTGAACGGTCACAAATTCAGCGTGCGTGGTGAAGGTGAAG
GCGATGCCACGATTGGCAAACTGACGCTGAAATTTATCTGCACCACCGGCAAACTGCCG
GTGCCGTGGCCGACGCTGGTGACCACCCTGACCTATGGCGTTCAGTGTTTTAGTCGCTA
TCCGGATCACATGAAACGTCACGATTTCTTTAAATCTGCAATGCCGGAAGGCTATGTGCA
GGAACGTACGATTAGCTTTAAAGATGATGGCAAATATAAAACGCGCGCCGTTGTGAAATT
TGAAGGCGATACCCTGGTGAACCGCATTGAACTGAAAGGCACGGATTTTAAAGAAGATG
GCAATATCCTGGGCCATAAACTGGAATACAACTTTAATAGCCATAATGTTTATATTACGGC
GGATAAACAGAAAAATGGCATCAAAGCGAATTTTACCGTTCGCCATAACGTTGAAGATGG
CAGTGTGCAGCTGGCAGATCATTATCAGCAGAATACCCCGATTGGTGATGGTCCGGTGC
TGCTGCCGGATAATCATTATCTGAGCACGCAGACCGTTCTGTCTAAAGATCCGAACGAAA
AAGGCACGCGGGACCACATGGTTCTGCACGAATATGTGAATGCGGCAGGTATTACGTG
GAGCCATCCGCAGTTCGAAAAATAAgtcgaccggctgctaacaaagcccgaaaggaagctgagttggctgctg
ccaccgctgagcaataactagcataaccccttggggcctctaaacgggtcttgaggggttttttg

**P$_{T7}$-*trigger B***	Linear DNA encoding expression of RNA trigger B under a P$_{T7}$ Promoter. Additional nucleotides (>20bp) are included in 5' and 3' end as extra protection against nuclease degradation.

P$_{T7}$ Promoter-*trigger B*-T7 Terminator

aacgccagcaacgcgatcccgcgaaattaatacgactcactatagggagaGGGATGCCCGTAGTTCATTCTAC
GGGCATGAATAACGACATACAGCAAGCGATTTACTTATACTAtagcataaccccttggggcctctaaa
cgggtcttgaggggttttttgtttgctgaaagccaattctgatta

**P$_{T7}$-*switch B-sfgfp***	Relevant part of the plasmid encoding superfolder GFP expression under P$_{T7}$ promoter and toehold switch B

P$_{T7}$ Promoter-*switch B*-*sfgfp*-TrrnB-T7 Terminator

taatacgactcactatagggagaGGGTATAAGTAAATCGCTTGCTGTATGTCGTTAAACAGAGGAG
ATAACGAATGACAGCAAGCAACCTGGCGGCAGCGCAAAAGATGAGCAAAGGTGAAGAA
CTGTTTACCGGCGTTGTGCCGATTCTGGTGGAACTGGATGGCGATGTGAACGGTCACAA
ATTCAGCGTGCGTGGTGAAGGTGAAGGCGATGCCACGATTGGCAAACTGACGCTGAAAT
TTATCTGCACCACCGGCAAACTGCCGGTGCCGTGGCCGACGCTGGTGACCACCCTGAC
CTATGGCGTTCAGTGTTTTAGTCGCTATCCGGATCACATGAAACGTCACGATTTCTTTAA
ATCTGCAATGCCGGAAGGCTATGTGCAGGAACGTACGATTAGCTTTAAAGATGATGGCA
AATATAAAACGCGCGCCGTTGTGAAATTTGAAGGCGATACCCTGGTGAACCGCATTGAA
CTGAAAGGCACGGATTTTAAAGAAGATGGCAATATCCTGGGCCATAAACTGGAATACAAC
TTTAATAGCCATAATGTTTATATTACGGCGGATAAACAGAAAAATGGCATCAAAGCGAATT
TTACCGTTCGCCATAACGTTGAAGATGGCAGTGTGCAGCTGGCAGATCATTATCAGCAG
AATACCCCGATTGGTGATGGTCCGGTGCTGCTGCCGGATAATCATTATCTGAGCACGCA
GACCGTTCTGTCTAAAGATCCGAACGAAAAAGGCACGCGGGACCACATGGTTCTGCACG
AATATGTGAATGCGGCAGGTATTACGTGGAGCCATCCGCAGTTCGAAAAATAA
ggatctgaagcttgggcccgaacaaaaactcatctcagaagaggatctgaatagcgccgtcgaccatcatcatcatcatcattga
gtttaaacggtctccagcttggctgttttggcggatgagagaagattttcagcctgatacagattaaatcagaacgcagaagcggtct
gataaaacagaatttgcctggcggcagtagcgcggtggtcccacctgaccccatgccgaactcagaagtgaaacgccgtagcg
ccgatggtagtgtggggtctccccatgcgagagtagggaactgccaggcatcaaataaaacgaaaggctcagtcgaaagactg

<table>
<tr><td>ggcctttcgttttatctgttgtttgtcggtgaactggatcgtcgaccggctgctaacaaagcccgaaaggaagctgagttggctgctgc
caccgctgagcaataactagcataaccccttggggcctctaaacgggtcttgaggggttttttg</td></tr>
</table>

<table>
<tr><td>P_{T7}-trigger H</td><td>Linear DNA encoding expression of RNA trigger H under a P_{T7} Promoter. Additional nucleotides (>20bp) are included in 5’ and 3’ end as extra protection against nuclease degradation.</td></tr>
<tr><td colspan="2">P_{T7} Promoter-trigger H-T7 Terminator

aacgccagcaacgcgatcccgcgaaattaatacgactcactatagggagaGGGACCGTGGACCGCATGAGGT
CCACGGTAAACATAACTATAACAAGCCTACAATTCATTCAAACtagcataaccccttggggcctctaaa
cgggtcttgaggggttttttgtttgctgaaagccaattctgatta</td></tr>
</table>

<table>
<tr><td>P_{T7}-switch H-sfgfp</td><td>Relevant part of the plasmid encoding superfolder GFP expression under P_{T7} promoter and toehold switch H</td></tr>
<tr><td colspan="2">P_{T7} Promoter-switch H-sfgfp-TrrnB-T7 Terminator

taatacgactcactatagggagaGGGTGAATGAATTGTAGGCTTGTTATAGTTATGAACAGAGGAG
ACATAACATGAACAAGCCTAACCTGGCGGCAGCGCAAAAGATGAGCAAAGGTGAAGAAC
TGTTTACCGGCGTTGTGCCGATTCTGGTGGAACTGGATGGCGATGTGAACGGTCACAAA
TTCAGCGTGCGTGGTGAAGGTGAAGGCGATGCCACGATTGGCAAACTGACGCTGAAATT
TATCTGCACCACCGGCAAACTGCCGGTGCCGTGGCCGACGCTGGTGACCACCCTGACC
TATGGCGTTCAGTGTTTTAGTCGCTATCCGGATCACATGAAACGTCACGATTTCTTTAAAT
CTGCAATGCCGGAAGGCTATGTGCAGGAACGTACGATTAGCTTTAAAGATGATGGCAAA
TATAAAACGCGCGCCGTTGTGAAATTTGAAGGCGATACCCTGGTGAACCGCATTGAACT
GAAAGGCACGGATTTTAAAGAAGATGGCAATATCCTGGGCCATAAACTGGAATACAACTT
TAATAGCCATAATGTTTATATTACGGCGGATAAACAGAAAAATGGCATCAAAGCGAATTTT
ACCGTTCGCCATAACGTTGAAGATGGCAGTGTGCAGCTGGCAGATCATTATCAGCAGAA
TACCCCGATTGGTGATGGTCCGGTGCTGCTGCCGGATAATCATTATCTGAGCACGCAGA
CCGTTCTGTCTAAAGATCCGAACGAAAAAGGCACGCGGGACCACATGGTTCTGCACGAA
TATGTGAATGCGGCAGGTATTACGTGGAGCCATCCGCAGTTCGAAAAATAAggatctgaagctt
gggcccgaacaaaaactcatctcagaagaggatctgaatagcgccgtcgaccatcatcatcatcatcattgagtttaaacggtctc
cagcttggctgttttggcggatgagagaagattttcagcctgatacagattaaatcagaacgcagaagcggtctgataaaacagaa
tttgcctggcggcagtagcgcggtggtcccacctgaccccatgccgaactcagaagtgaaacgccgtagcgccgatggtagtgtg
gggtctccccatgcgagagtagggaactgccaggcatcaaataaaacgaaaggctcagtcgaaagactgggcctttcgttttatct
gttgtttgtcggtgaactggatcgtcgaccggctgctaacaaagcccgaaaggaagctgagttggctgctgccaccgctgagcaat
aactagcataaccccttggggcctctaaacgggtcttgaggggttttttg</td></tr>
</table>

<table>
<tr><td>P_{T7}-zntR</td><td>Relevant part of the plasmid encoding ZntR expression under P_{T7} promoter with strong ribosomal binding site</td></tr>
<tr><td colspan="2">P_{T7} Promoter-Stability Hairpin-StrongRBS-zntR-T7 Terminator

taatacgactcactatagggagaccacaacggtttccctctagaaataattttgtttaactttaagaaggagatatacatATGTAT
CGCATTGGTGAGCTGGCAAAAATGGCGGAAGTAACACCCGACACGATTCGTTATTACGA
AAAACAGCAGATGATGGAGCATGAAGTGCGTACTGAAGGTGGGTTTCGCCTATATACCG
AAAGCGATCTCCAGCGATTGAAATTTATCCGCCATGCCAGACAACTAGGTTTCAGTCTGG
AGTCGATCCGCGAGTTGCTGTCGATCCGCATCGATCCTGAACACCATACCTGTCAGGAG
TCAAAAGGCATTGTGCAGGAAAGATTGCAGGAAGTCGAAGCACGGATAGCCGAGTTGCA</td></tr>
</table>

GAGTATGCAGCGTTCCTTGCAACGCCTTAACGATGCCTGTTGTGGGACTGCTCATAGCA<br>GTGTTTATTGTTCGATTCTTGAAGCTCTTGAACAAGGGGCGAGTGGCGTTAAGAGTGGTT<br>GTTGATAAgtcgaccggctgctaacaaagcccgaaaggaagctgagttggctgctgccaccgctgagcaataactagcat<br>aacccttggggcctctaaacgggtcttgaggggttttttg

P_{ZntA}-***sfgfp***	Relevant part of the plasmid encoding superfolder GFP expression under ZntR regulated P_{ZntA} promoter
P_{ZntA}-Stability Hairpin-StrongRBS-*sfgfp*- T7 Terminator<br><br>CTGTATCTCTGATAAAACTTGACTCTGGAGTCGACTCCAGAGTGTATCCTTCGGTTAAT<br>ccacaacggtttccctctagaaataattttgtttaactttaagaaggagatatacatATGAGCAAAGGTGAAGAACTGT<br>TTACCGGCGTTGTGCCGATTCTGGTGGAACTGGATGGCGATGTGAACGGTCACAAATTC<br>AGCGTGCGTGGTGAAGGTGAAGGCGATGCCACGATTGGCAAACTGACGCTGAAATTTAT<br>CTGCACCACCGGCAAACTGCCGGTGCCGTGGCCGACGCTGGTGACCACCCTGACCTAT<br>GGCGTTCAGTGTTTTAGTCGCTATCCGGATCACATGAAACGTCACGATTTCTTTAAATCT<br>GCAATGCCGGAAGGCTATGTGCAGGAACGTACGATTAGCTTTAAAGATGATGGCAAATA<br>TAAAACGCGCGCCGTTGTGAAATTTGAAGGCGATACCCTGGTGAACCGCATTGAACTGA<br>AAGGCACGGATTTTAAAGAAGATGGCAATATCCTGGGCCATAAACTGGAATACAACTTTA<br>ATAGCCATAATGTTTATATTACGGCGGATAAACAGAAAAATGGCATCAAAGCGAATTTTAC<br>CGTTCGCCATAACGTTGAAGATGGCAGTGTGCAGCTGGCAGATCATTATCAGCAGAATA<br>CCCCGATTGGTGATGGTCCGGTGCTGCTGCCGGATAATCATTATCTGAGCACGCAGACC<br>GTTCTGTCTAAAGATCCGAACGAAAAAGGCACGCGGGACCACATGGTTCTGCACGAATA<br>TGTGAATGCGGCAGGTATTACGTGGAGCCATCCGCAGTTCGAAAAATAAgtcgaccggctgcta<br>acaaagcccgaaaggaagctgagttggctgctgccaccgctgagcaataactagcataacccttggggcctctaaacgggtctt<br>gaggggttttttg	

P_{T7}-***eutR***	Relevant part of the plasmid encoding EutR expression under P_{T7} promoter with strong ribosomal binding site.
P_{T7} Promoter-Stability Hairpin-StrongRBS-*eutR*-T7 Terminator<br><br>taatacgactcactatagggagaccacaacggtttccctctagaaataattttgtttaactttaagaaggagatatacatATGAAA<br>AAGACCCGTACAGCCAATTTGCACCATCTTTATCATGAACCCTTACCCGAAAACCTGAAG<br>CTCACGCCGAAGGTCGAAGTGGATAATGTTCATCAACGACAGACAACGGATGTCTATGA<br>ACATGCTTTAACGATTACCGCCTGGCAGCAGATTTACGATCAGCTGCATCCGGGCAAGT<br>TTCATGGTGAATTTACGGAAATTCTACTCGATGATATTCAGGTTTTTCGTGAATACACCGG<br>TCTGGCGCTGCGTCAGTCGTGCCTGGTCTGGCCGAACTCGTTCTGGTTTGGCATTCCGG<br>CGACGCGCGGTGAGCAGGGATTTATCGGTTCGCAATGTCTGGGAAGCGCGGAAATCGC<br>CACCCGCCCTGGTGGCACTGAATTTGAACTGAGCACGCCGGATGATTACACGATCCTGG<br>GCGTGGTGCTTTCTGAAGATGTCATCACCCGGCAGGCTAACTTTTTGCATAACCCGGAT<br>CGGGTATTACATATGTTGCGTAACCAGTCGGCGCTGGAAGTGAAAGAGCAGCATAAAGC<br>CGCGCTGTGGGGCTTTGTCCAACAGGCGCTGGCGACGTTTTGCGAGAATCCGGAAAAT<br>CTCCATCAGCCAGCAGTGCGAAAAGTGCTGGGGGATAATTTGCTAATGGCGATGGGGG<br>CCATGCTGGAAGAAGCGCAACCAATGGTGACGGCGGAAAGCATCAGTCATCAGAGTTAC<br>CGTCGATTGCTTTCCCGCGCCCGTGAATATGTGCTGGAAAACATGTCCGAACCGGTGAC<br>GGTGCTGGATTTGTGTAATCAACTGCATGTCAGCCGCCGCACGCTACAAAACGCGTTTC<br>ACGCTATTTTAGGCATTGGCCCGAACGCGTGGCTGAAACGCATTCGCCTGAACGCCGTA<br>CGCCGCGAACTGATAAGTCCGTGGTCGCAAAGTATGACGGTAAAAGACGCCGCCATGC<br>AGTGGGGATTCTGGCATCTGGGGCAATTTGCCACGGATTACCAGCAGCTGTTTTCCGAG	

AAGCCGTCACTGACGCTGCATCAGCGGATGCGGGAGTGGGGGTGAgtcgaccggctgctaaca
aagcccgaaaggaagctgagttggctgctgccaccgctgagcaataactagcataaccccttggggcctctaaacgggtcttga
ggggttttttg

**P_{EutS}-*sfgfp***	Relevant part of the plasmid encoding superfolder GFP expression under EutR regulated P_{EutS} promoter

P_{EutS}-Stability Hairpin-StrongRBS-*sfgfp*- T7 Terminator

AACAGAGCGAAGTGGTGGCTTCAGCGTATGGCGATCAGGATCTGAGCTTTGGTCCGGA
ATACATCATTCCAAAACCGTTTGATCCGCGCTTGATCGTTAAGATCGCTCCTGCGGTCGC
TAAAGCCGCGATGGAGTCGGGCGTGGCGACTCGTCCGATTGCTGATTTCGACGTCTACA
TCGACAAGCTGACTGAGTTCGTTTACAAAACCAACCTGTTTATGAAGCCGATTTTCTCCC
AGGCTCGCAAAGCGCCGAAGCGCGTTGTTCTGCCGGAAGGGGAAGAGGCGCGCGTTC
TGCATGCCACTCAGGAACTGGTAACGCTGGGACTGGCGAAACCGATCCTTATCGGTCGT
CCGAACGTGATCGAAATGCGCATTCAGAAACTGGGCTTGCAGATCAAAGCGAGCGTTGA
TTTTGAGATCGTCAATAACGAATCCGATCCGCGCTTTAAAGAGTACTGGACCGAATACTT
CCAGATCATGAAGCGTCGCGGCGTCACTCAGGAACAGGCGCAGCGGGCGCTGATCAGT
AACCCGACAGTGATCGGCGCGATCATGGTTCAGCGTGGGGAAGCCGATGCAATGATTT
GCGGTACGGTGGGTGATTATCATGAACATTTTAGCGTGGTGAAAAATGTCTTTGGTTATC
GCGATGGCGTTCACACCGCAGGTGCCATGAACGCGCTGCTGCTGCCGAGTGGTAACAC
CTTTATTGCCGATACATATGTTAATGATGAACCGGATGCAGAAGAGCTGGCGGAGATCA
CCTTGATGGCGGCAGAAACTGTCCGTCGTTTTGGTATTGAGCCGCGCGTTGCTTTGTTG
TCGCACTCCAACTTTGGTTCTTCTGACTGCCCGTCGTCGAGCAAAATGCGTCAGGCGCT
GGAACTGGTCAGGGAACGTGCACCAGAACTGATGATTGATGGTGAAATGCACGGCGAT
GCAGCGCTGGTGGAAGCGATTCGCAACGACCGTATGCCGGACAGCTCTTTGAAAGGTT
CCGCCAATATTCTGGTGATGCCGAACATGGAAGCTGCCCGCATTAGTTACAACTTACTG
CGTGTTTCCAGCTCGGAAGGTGTGACTGTCGGCCCGGTGCTGATGGGTGTGGCGAAAC
CGGTTCACGTGTTAACGCCGATCGCATCGGTGCGTCGTATCGTCAACATGGTGGCGCTG
GCCGTGGTAGAAGCGCAAACCCAACCGCTGTAATTTTTTTTAACTCTCACGCTTATCCTG
AATATTCAGGGTAAGCAGTTTAGCTGCAATATATTAGTAAAGCTTATTACTGAGTTTGCGA
ATAATAAAAAAAAGCAGTCTATATAATATCTCGATATTATTTTATTTATATTCATGCGTTGC
ATATGAAAGTTTATGCACCACAGCGAATATCTCTCCATTCTTAGTGATCTACCTCACCTTT
TAAACGCGCTTGCCGAATTTTGTTATTTACTCTGACGAAAAATTGTCACGATACACGAAA
GTTTTTCACAGGCGGCGACTCccacaacggtttccctctagaaataattttgtttaactttaagaaggagatatacatA
TGAGCAAAGGTGAAGAACTGTTTACCGGCGTTGTGCCGATTCTGGTGGAACTGGATGGC
GATGTGAACGGTCACAAATTCAGCGTGCGTGGTGAAGGTGAAGGCGATGCCACGATTG
GCAAACTGACGCTGAAATTTATCTGCACCACCGGCAAACTGCCGGTGCCGTGGCCGAC
GCTGGTGACCACCCTGACCTATGGCGTTCAGTGTTTTAGTCGCTATCCGGATCACATGA
AACGTCACGATTTCTTTAAATCTGCAATGCCGGAAGGCTATGTGCAGGAACGTACGATTA
GCTTTAAAGATGATGGCAAATATAAAACGCGCGCCGTTGTGAAATTTGAAGGCGATACCC
TGGTGAACCGCATTGAACTGAAAGGCACGGATTTTAAAGAAGATGGCAATATCCTGGGC
CATAAACTGGAATACAACTTTAATAGCCATAATGTTTATATTACGGCGGATAAACAGAAAA
ATGGCATCAAAGCGAATTTTACCGTTCGCCATAACGTTGAAGATGGCAGTGTGCAGCTG
GCAGATCATTATCAGCAGAATACCCCGATTGGTGATGGTCCGGTGCTGCTGCCGGATAA
TCATTATCTGAGCACGCAGACCGTTCTGTCTAAAGATCCGAACGAAAAAGGCACGCGGG
ACCACATGGTTCTGCACGAATATGTGAATGCGGCAGGTATTACGTGGAGCCATCCGCAG
TTCGAAAAATAAgtcgaccggctgctaacaaagcccgaaaggaagctgagttggctgctgccaccgctgagcaataact
agcataaccccttggggcctctaaacgggtcttgaggggttttttg

**P_{T7}-*trigger BT***	Linear DNA encoding expression of RNA trigger BT under a P_{T7} Promoter. Additional nucleotides (~30bp) are included in 5' and 3' end as extra protection against nuclease degradation.
P_{T7} Promoter-*trigger BT*<br><br>ggaaaaacgccagcaacgcgatcccgcgaaattaatacgactcactataggCCGACTTCGGAACGCTTATAGA<br>AAGGAGCAACACCACACAAAGCCGGTCATACAGTAATTCAGCTACCGCATACGTTTCAaa<br>aaaaaacgccgcctttcggcggcgtttg	

**P_{T7}-*switch BT-sfgfp***	Relevant part of the plasmid encoding superfolder GFP expression under P_{T7} promoter and toehold switch BT
T7 Promoter-*switch BT*-*sfgfp*-TrrnB-T7 Terminator<br><br>taatacgactcactatagggagaGTTACTGTATGACCGGCTTTGTGTGGTGTTGCTCCTTGGACTTT<br>AGAACAGAGGAGATAAAGATGAAGGAGCAACACAACCTGGCGGCAGCGCAAAAGATGA<br>GCAAAGGTGAAGAACTGTTTACCGGCGTTGTGCCGATTCTGGTGGAACTGGATGGCGAT<br>GTGAACGGTCACAAATTCAGCGTGCGTGGTGAAGGTGAAGGCGATGCCACGATTGGCA<br>AACTGACGCTGAAATTTATCTGCACCACCGGCAAACTGCCGGTGCCGTGGCCGACGCT<br>GGTGACCACCCTGACCTATGGCGTTCAGTGTTTTAGTCGCTATCCGGATCACATGAAAC<br>GTCACGATTTCTTTAAATCTGCAATGCCGGAAGGCTATGTGCAGGAACGTACGATTAGCT<br>TTAAAGATGATGGCAAATATAAAACGCGCGCCGTTGTGAAATTTGAAGGCGATACCCTG<br>GTGAACCGCATTGAACTGAAAGGCACGGATTTTAAAGAAGATGGCAATATCCTGGGCCA<br>TAAACTGGAATACAACTTTAATAGCCATAATGTTTATATTACGGCGGATAAACAGAAAAT<br>GGCATCAAAGCGAATTTTACCGTTCGCCATAACGTTGAAGATGGCAGTGTGCAGCTGGC<br>AGATCATTATCAGCAGAATACCCCGATTGGTGATGGTCCGGTGCTGCTGCCGGATAATC<br>ATTATCTGAGCACGCAGACCGTTCTGTCTAAAGATCCGAACGAAAAAGGCACGCGGGAC<br>CACATGGTTCTGCACGAATATGTGAATGCGGCAGGTATTACGTGGAGCCATCCGCAGTT<br>CGAAAAATAAggatctgaagcttgggcccgaacaaaaactcatctcagaagaggatctgaatagcgccgtcgaccatcat<br>catcatcatcattgagtttaaacggtctccagcttggctgttttggcggatgagagaagattttcagcctgatacagattaaatcagaa<br>cgcagaagcggtctgataaaacagaatttgcctggcggcagtagcgcggtggtcccacctgaccccatgccgaactcagaagtg<br>aaacgccgtagcgccgatggtagtgtggggtctccccatgcgagagtagggaactgccaggcatcaaataaaacgaaaggctc<br>agtcgaaagactgggcctttcgttttatctgttgtttgtcggtgaactggatcgtcgaccggctgctaacaaagcccgaaaggaagct<br>gagttggctgctgccaccgctgagcaataactagcataaccccttggggcctctaaacgggtcttgaggggttttttg	

**P_{T7}-*trigger S1***	Linear DNA encoding expression of RNA trigger S1 under a P_{T7} Promoter. Additional nucleotides (~30bp) are included in 5' and 3' end as extra protection against nuclease degradation.
P_{T7} Promoter-*trigger S1*<br><br>ggaaaaacgccagcaacgcgatcccgcgaaattaatacgactcactataggATAAATCGCCATTCGTTGACTAC<br>TTCTTATCTGGATTTAATGTCGCATAGTGGAACCTCACTGACGCAGTCTGTGGCAAGAGC<br>GATGTTACGGTTTaaaaaaaacgccgcctttcggcggcgtttg	

<table>
<tr><td>P_{T7}-switch S1-sfgfp</td><td>Relevant part of the plasmid encoding superfolder GFP expression under P_{T7} promoter and toehold switch S1</td></tr>
<tr><td colspan="2">P_{T7} Promoter-switch S1-sfgfp-TrrnB-T7 Terminator

taatacgactcactatagggagaGGGCGTCAGTGAGGTTCCACTATGCGACATTAAATCCAGGGAC
TTTAGAACAGAGGAGATAAAGATGCTGGATTTAATTAACCTGGCGGCAGCGCAAAAGAT
GAGCAAAGGTGAAGAACTGTTTACCGGCGTTGTGCCGATTCTGGTGGAACTGGATGGC
GATGTGAACGGTCACAAATTCAGCGTGCGTGGTGAAGGTGAAGGCGATGCCACGATTG
GCAAACTGACGCTGAAATTTATCTGCACCACCGGCAAACTGCCGGTGCCGTGGCCGAC
GCTGGTGACCACCCTGACCTATGGCGTTCAGTGTTTTAGTCGCTATCCGGATCACATGA
AACGTCACGATTTCTTTAAATCTGCAATGCCGGAAGGCTATGTGCAGGAACGTACGATTA
GCTTTAAAGATGATGGCAAATATAAAACGCGCGCCGTTGTGAAATTTGAAGGCGATACCC
TGGTGAACCGCATTGAACTGAAAGGCACGGATTTTAAAGAAGATGGCAATATCCTGGGC
CATAAACTGGAATACAACTTTAATAGCCATAATGTTTATATTACGGCGGATAAACAGAAAA
ATGGCATCAAAGCGAATTTTACCGTTCGCCATAACGTTGAAGATGGCAGTGTGCAGCTG
GCAGATCATTATCAGCAGAATACCCCGATTGGTGATGGTCCGGTGCTGCTGCCGGATAA
TCATTATCTGAGCACGCAGACCGTTCTGTCTAAAGATCCGAACGAAAAAGGCACGCGGG
ACCACATGGTTCTGCACGAATATGTGAATGCGGCAGGTATTACGTGGAGCCATCCGCAG
TTCGAAAAATAAggatctgaagcttgggcccgaacaaaaactcatctcagaagaggatctgaatagcgccgtcgaccatc
atcatcatcatcattgagtttaaacggtctccagcttggctgttttggcggatgagagaagattttcagcctgatacagattaaatcaga
acgcagaagcggtctgataaaacagaatttgcctggcggcagtagcgcggtggtcccacctgaccccatgccgaactcagaagt
gaaacgccgtagcgccgatggtagtgtggggtctccccatgcgagagtagggaactgccaggcatcaaataaaacgaaaggct
cagtcgaaagactgggcctttcgttttatctgttgtttgtcggtgaactggatcgtcgaccggctgctaacaaagcccgaaaggaagc
tgagttggctgctgccaccgctgagcaataactagcataaccccttggggcctctaaacgggtcttgaggggttttttg</td></tr>
</table>

<table>
<tr><td>P_{T7}-trigger S2</td><td>Linear DNA encoding expression of RNA trigger S2 under a P_{T7} Promoter. Additional nucleotides (~30bp) are included in 5' and 3' end as extra protection against nuclease degradation.</td></tr>
<tr><td colspan="2">P_{T7} Promoter-trigger S2

ggaaaaacgccagcaacgcgatcccgcgaaattaatacgactcactataggGTATCCTATTCCCGGGAGTTTAC
GATAGACTTTTCGACCCAACAAAGTTATGTCTCTTCGTTAAATAGTATACGGACAGAGATA
TCGACCCCTCTTGAACATATATCaaaaaaaacgccgcctttcggcggcgtttg</td></tr>
</table>

<table>
<tr><td>P_{T7}-switch S2-sfgfp</td><td>Relevant part of the plasmid encoding superfolder GFP expression under P_{T7} promoter and toehold switch S2</td></tr>
<tr><td colspan="2">P_{T7} Promoter-switch S2-sfgfp-TrrnB-T7 Terminator

taatacgactcactatagggagaGGGATACTATTTAACGAAGAGACATAACTTTGTTGGGTCGGACT
TTAGAACAGAGGAGATAAAGATGGACCCAACAAAGATGAGCAAAGGTGAAGAACTGTTT
ACCGGCGTTGTGCCGATTCTGGTGGAACTGGATGGCGATGTGAACGGTCACAAATTCAG
CGTGCGTGGTGAAGGTGAAGGCGATGCCACGATTGGCAAACTGACGCTGAAATTTATCT
GCACCACCGGCAAACTGCCGGTGCCGTGGCCGACGCTGGTGACCACCCTGACCTATGG
CGTTCAGTGTTTTAGTCGCTATCCGGATCACATGAAACGTCACGATTTCTTTAAATCTGCA
ATGCCGGAAGGCTATGTGCAGGAACGTACGATTAGCTTTAAAGATGATGGCAAATATAAA
ACGCGCGCCGTTGTGAAATTTGAAGGCGATACCCTGGTGAACCGCATTGAACTGAAAGG
CACGGATTTTAAAGAAGATGGCAATATCCTGGGCCATAAACTGGAATACAACTTTAATAG</td></tr>
</table>

CCATAATGTTTATATTACGGCGGATAAACAGAAAAATGGCATCAAAGCGAATTTTACCGTT<br>CGCCATAACGTTGAAGATGGCAGTGTGCAGCTGGCAGATCATTATCAGCAGAATACCCC<br>GATTGGTGATGGTCCGGTGCTGCTGCCGGATAATCATTATCTGAGCACGCAGACCGTTC<br>TGTCTAAAGATCCGAACGAAAAAGGCACGCGGACCACATGGTTCTGCACGAATATGTG<br>AATGCGGCAGGTATTACGTGGAGCCATCCGCAGTTCGAAAAATAAggatctgaagcttgggcccg<br>aacaaaaactcatctcagaagaggatctgaatagcgccgtcgaccatcatcatcatcatcattgagtttaaacggtctccagcttgg<br>ctgttttggcggatgagagaagattttcagcctgatacagattaaatcagaacgcagaagcggtctgataaaacagaatttgcctgg<br>cggcagtagcgcggtggtcccacctgaccccatgccgaactcagaagtgaaacgccgtagcgccgatggtagtgtggggtctcc<br>ccatgcgagagtagggaactgccaggcatcaaataaaacgaaaggctcagtcgaaagactgggcctttcgttttatctgttgtttgt<br>cggtgaactggatcgtcgaccggctgctaacaaagcccgaaaggaagctgagttggctgctgccaccgctgagcaataactag<br>cataaccccttggggcctctaaacgggtcttgaggggttttttg

**P_{T7}-*lacZ***	Relevant part of the plasmid encoding β-galactosidase (LacZ) expression under P_{T7} promoter
P_{T7} Promoter- Stability Hairpin-StrongRBS-*lacZ*-T7 Terminator<br><br>taatacgactcactatagggagaccacaacggtttccctctagaaataattttgtttaactttaagaaggagatatacatatgACCA<br>TGATTACGGATTCACTGGCCGTCGTTTTACAACGTCGTGACTGGGAAAACCCTGGCGTT<br>ACCCAACTTAATCGCCTTGCAGCACATCCCCCTTTCGCCAGCTGGCGTAATAGCGAAGA<br>GGCCCGCACCGATCGCCCTTCCCAACAGTTGCGCAGCCTGAATGGCGAATGGCGCTTT<br>GCCTGGTTTCCGGCACCAGAAGCGGTGCCGGAAAGCTGGCTGGAGTGCGATCTTCCTG<br>AGGCCGATACTGTCGTCGTCCCCTCAAACTGGCAGATGCACGGTTACGATGCGCCCATC<br>TACACCAACGTGACCTATCCCATTACGGTCAATCCGCCGTTTGTTCCCACGGAGAATCC<br>GACGGGTTGTTACTCGCTCACATTTAATGTTGATGAAAGCTGGCTACAGGAAGGCCAGA<br>CGCGAATTATTTTTGATGGCGTTAACTCGGCGTTTCATCTGTGGTGCAACGGGCGCTGG<br>GTCGGTTACGGCCAGGACAGTCGTTTGCCGTCTGAATTTGACCTGAGCGCATTTTTACG<br>CGCCGGAGAAAACCGCCTCGCGGTGATGGTGCTGCGCTGGAGTGACGGCAGTTATCTG<br>GAAGATCAGGATATGTGGCGGATGAGCGGCATTTTCCGTGACGTCTCGTTGCTGCATAA<br>ACCGACTACACAAATCAGCGATTTCCATGTTGCCACTCGCTTTAATGATGATTTCAGCCG<br>CGCTGTACTGGAGGCTGAAGTTCAGATGTGCGGCGAGTTGCGTGACTACCTACGGGTA<br>ACAGTTTCTTTATGGCAGGGTGAAACGCAGGTCGCCAGCGGCACCGCGCCTTTCGGCG<br>GTGAAATTATCGATGAGCGTGGTGGTTATGCCGATCGCGTCACACTACGTCTGAACGTC<br>GAAAACCCGAAACTGTGGAGCGCCGAAATCCCGAATCTCTATCGTGCGGTGGTTGAACT<br>GCACACCGCCGACGGCACGCTGATTGAAGCAGAAGCCTGCGATGTCGGTTTCCGCGAG<br>GTGCGGATTGAAAATGGTCTGCTGCTGCTGAACGGCAAGCCGTTGCTGATTCGAGGCGT<br>TAACCGTCACGAGCATCATCCTCTGCATGGTCAGGTCATGGATGAGCAGACGATGGTGC<br>AGGATATCCTGCTGATGAAGCAGAACAACTTTAACGCCGTGCGCTGTTCGCATTATCCG<br>AACCATCCGCTGTGGTACACGCTGTGCGACCGCTACGGCCTGTATGTGGTGGATGAAG<br>CCAATATTGAAACCCACGGCATGGTGCCAATGAATCGTCTGACCGATGATCCGCGCTGG<br>CTACCGGCGATGAGCGAACGCGTAACGCGAATGGTGCAGCGCGATCGTAATCACCCGA<br>GTGTGATCATCTGGTCGCTGGGGAATGAATCAGGCCACGGCGCTAATCACGACGCGCT<br>GTATCGCTGGATCAAATCTGTCGATCCTTCCCGCCCGGTGCAGTATGAAGGCGGCGGA<br>GCCGACACCACGGCCACCGATATTATTTGCCCGATGTACGCGCGCGTGGATGAAGACC<br>AGCCCTTCCCGGCTGTGCCGAAATGGTCCATCAAAAAATGGCTTTCGCTACCTGGAGAG<br>ACGCGCCCGCTGATCCTTTGCGAATACGCCCACGCGATGGGTAACAGTCTTGGCGGTTT<br>CGCTAAATACTGGCAGGCGTTTCGTCAGTATCCCCGTTTACAGGGCGGCTTCGTCTGGG<br>ACTGGGTGGATCAGTCGCTGATTAAATATGATGAAAACGGCAACCCGTGGTCGGCTTAC<br>GGCGGTGATTTTGGCGATACGCCGAACGATCGCCAGTTCTGTATGAACGGTCTGGTCTT	

<table>
<tr><td>TGCCGACCGCACGCCGCATCCAGCGCTGACGGAAGCAAAACACCAGCAGCAGTTTTTC
CAGTTCCGTTTATCCGGGCAAACCATCGAAGTGACCAGCGAATACCTGTTCCGTCATAG
CGATAACGAGCTCCTGCACTGGATGGTGGCGCTGGATGGTAAGCCGCTGGCAAGCGGT
GAAGTGCCTCTGGATGTCGCTCCACAAGGTAAACAGTTGATTGAACTGCCTGAACTACC
GCAGCCGGAGAGCGCCGGGCAACTCTGGCTCACAGTACGCGTAGTGCAACCGAACGC
GACCGCATGGTCAGAAGCCGGACACATCAGCGCCTGGCAGCAGTGGCGTCTGGCTGAA
AACCTCAGCGTGACACTCCCCGCCGCGTCCCACGCCATCCCGCATCTGACCACCAGCG
AAATGGATTTTTGCATCGAGCTGGGTAATAAGCGTTGGCAATTTAACCGCCAGTCAGGCT
TTCTTTCACAGATGTGGATTGGCGATAAAAAACAACTGCTGACGCCGCTGCGCGATCAG
TTCACCCGTGCACCGCTGGATAACGACATTGGCGTAAGTGAAGCGACCCGCATTGACCC
TAACGCCTGGGTCGAACGCTGGAAGGCGGCGGGCCATTACCAGGCCGAAGCAGCGTT
GTTGCAGTGCACGGCAGATACACTTGCTGATGCGGTGCTGATTACGACCGCTCACGCGT
GGCAGCATCAGGGGAAAACCTTATTTATCAGCCGGAAAACCTACCGGATTGATGGTAGT
GGTCAAATGGCGATTACCGTTGATGTTGAAGTGGCGAGCGATACACCGCATCCGGCGC
GGATTGGCCTGAACTGCCAGCTGGCGCAGGTAGCAGAGCGGGTAAACTGGCTCGGATT
AGGGCCGCAAGAAAACTATCCCGACCGCCTTACTGCCGCCTGTTTTGACCGCTGGGATC
TGCCATTGTCAGACATGTATACCCCGTACGTCTTCCCGAGCGAAAACGGTCTGCGCTGC
GGGACGCGCGAATTGAATTATGGCCCACACCAGTGGCGCGGCGACTTCCAGTTCAACA
TCAGCCGCTACAGTCAACAGCAACTGATGGAAACCAGCCATCGCCATCTGCTGCACGCG
GAAGAAGGCACATGGCTGAATATCGACGGTTTCCATATGGGGATTGGTGGCGACGACTC
CTGGAGCCCGTCAGTATCGGCGGAATTCCAGCTGAGCGCCGGTCGCTACCATTACCAG
TTGGTCTGGTGTCAAAAAtaagtcgaccggctgctaacaaagcccgaaaggaagctgagttggctgctgccaccgct
gagcaataactagcataaccccttggggcctctaaacgggtcttgaggggttttttg</td></tr>
</table>

<table>
<tr><td>χDNA</td><td>DNA oligo containing 6 χ sites to stall endonuclease degradation on linear DNA templates.</td></tr>
<tr><td colspan="2">FW:
TCACTTCACTGCTGGTGGCCACTGCTGGTGGCCACTGCTGGTGGCCACTGCTGGTGGC
CACTGCTGGTGGCCACTGCTGGTGGCCA</td></tr>
<tr><td colspan="2">RV:
TGGCCACCAGCAGTGGCCACCAGCAGTGGCCACCAGCAGTGGCCACCAGCAGTGGCC
ACCAGCAGTGGCCACCAGCAGTGAAGTGA</td></tr>
</table>

<table>
<tr><td>P_{T7}-Null</td><td>Linear DNA encoding expression of random RNA sequences under a P_{T7} Promoter. Additional nucleotides (>20bp) are included in 5’ and 3’ end as extra protection against nuclease degradation.</td></tr>
<tr><td colspan="2">P_{T7} Promoter-Null

aacgccagcaacgcgatcccgcgaaattaatacgactcactatagggagaGTCGACCGGCTGCTAACAAAGCC
CGAAAGGAAGCTGAGTTGGCTGCTGCCACCGCTGAGCAATAACtagcataaccccttggggcctct
aaacgggtcttgaggggttttttgtttgctgaaagccaattctga</td></tr>
</table>

<table>
<tr><td>P_{T7}-trigger EC</td><td>Linear DNA encoding expression of RNA trigger EC under a P_{T7} Promoter. Additional nucleotides (~20bp) are included in 5’ and 3’ end as extra protection against nuclease degradation.</td></tr>
<tr><td colspan="2">P_{T7} Promoter-trigger EC</td></tr>
</table>

aacgccagcaacgcgatcccgcgaaattaatacgactcactatagggagaAAGACGGATATCTATTTCGTTTCC
ACGTTTGAACCGtagcataaccccttggggcctctaaacgggtcttgaggggttttttgtttgctgaaagccaattctga
