# Supplementary figures and images for: Protocell arrays for simultaneous detection of diverse analytes
Source: Nat Commun. 2021 Sep 29;12:5724. doi: 10.1038/s41467-021-25989-3 (PMC8481512; doi:10.1038/s41467-021-25989-3)

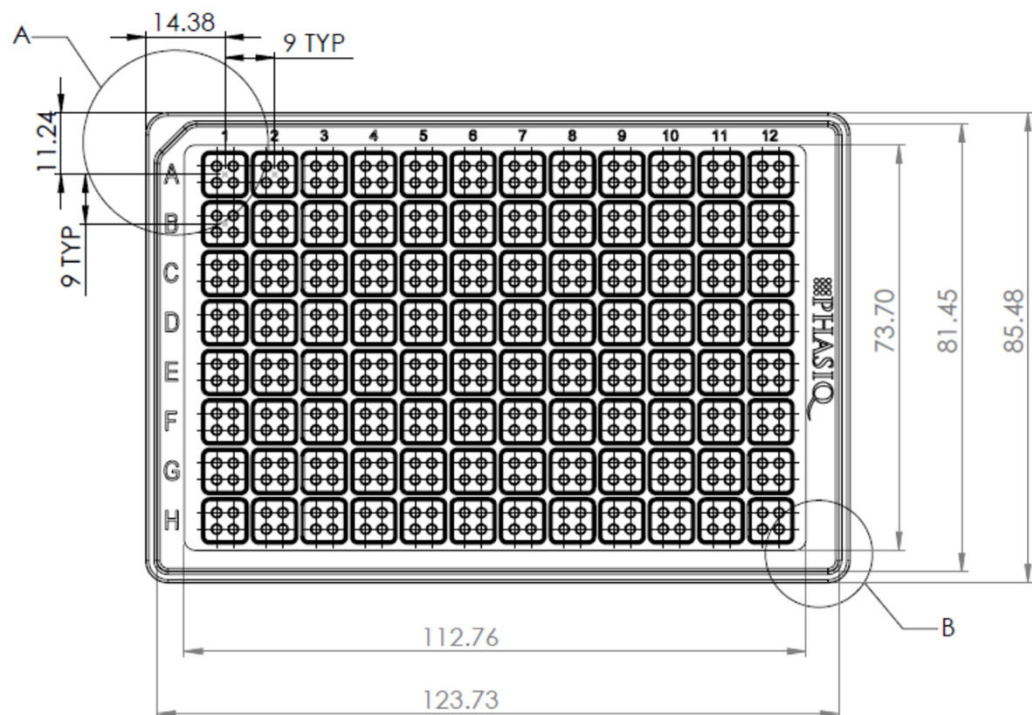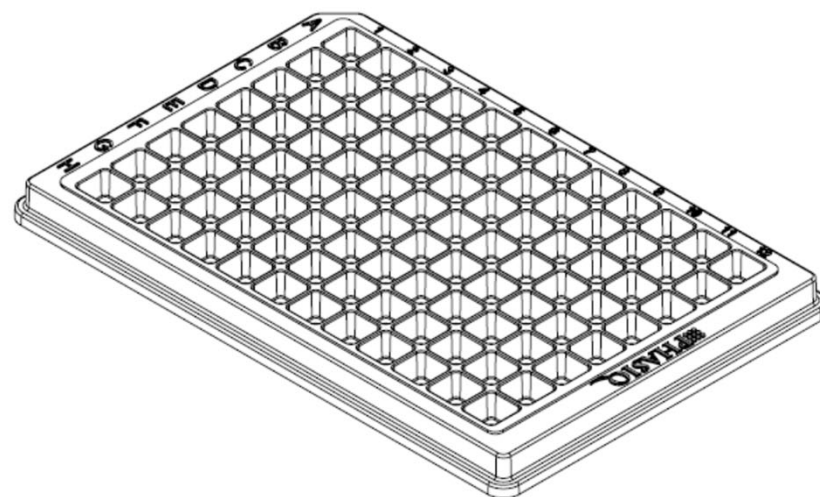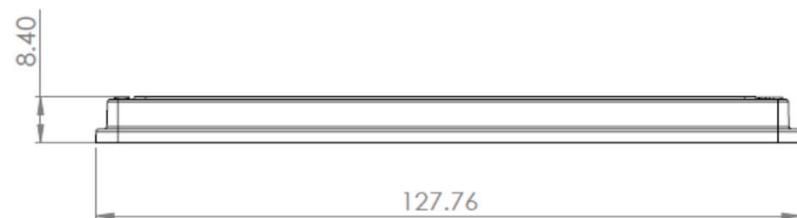

Supplement: Supplementary file 4 — Source Data [file 41467_2021_25989_MOESM4_ESM.zip › 96well4microbasinb.PDF]

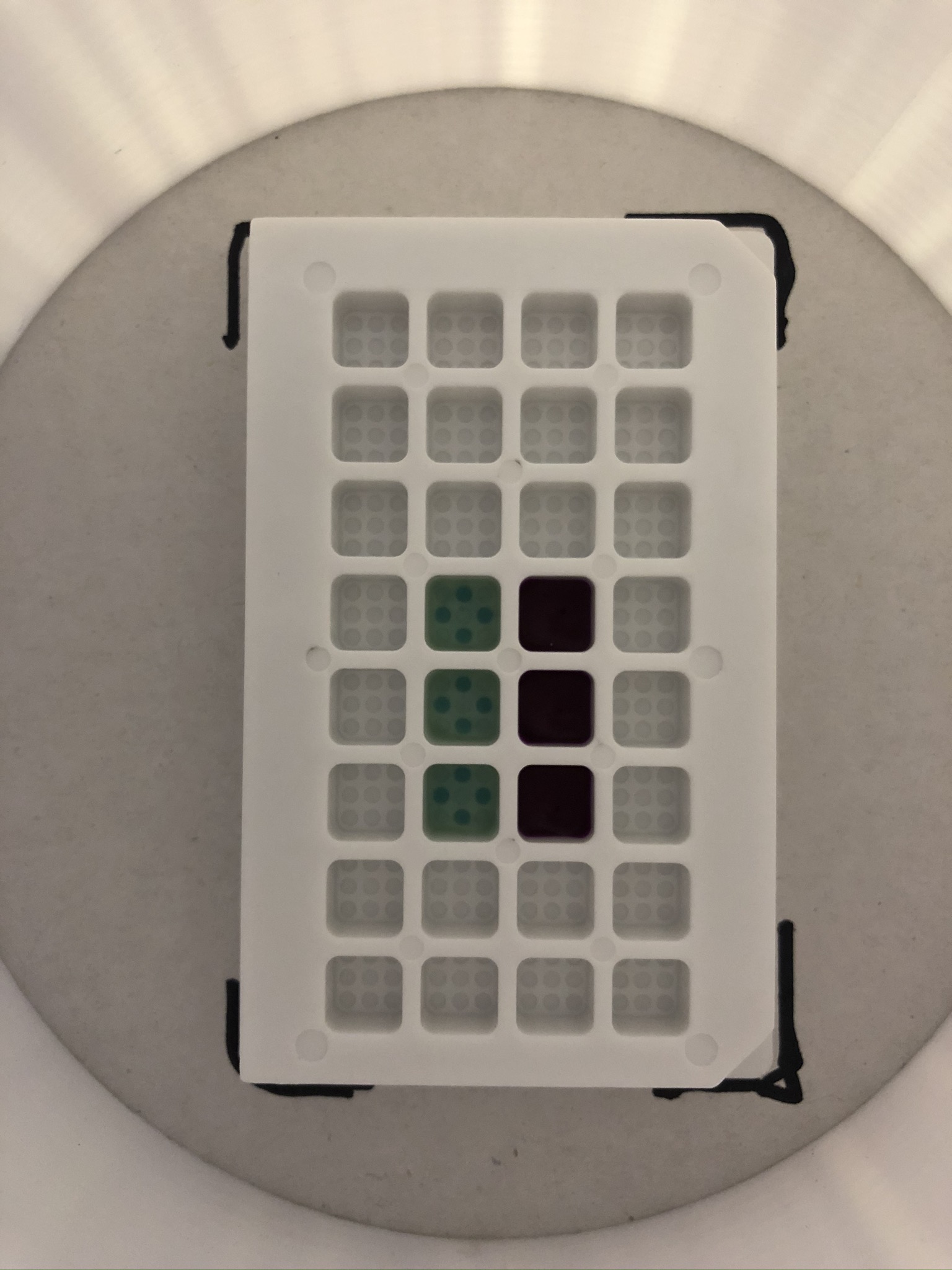

Supplement: Supplementary file 4 — Source Data [file 41467_2021_25989_MOESM4_ESM.zip › Image Files/Supp Fig 15/OVN.JPEG]

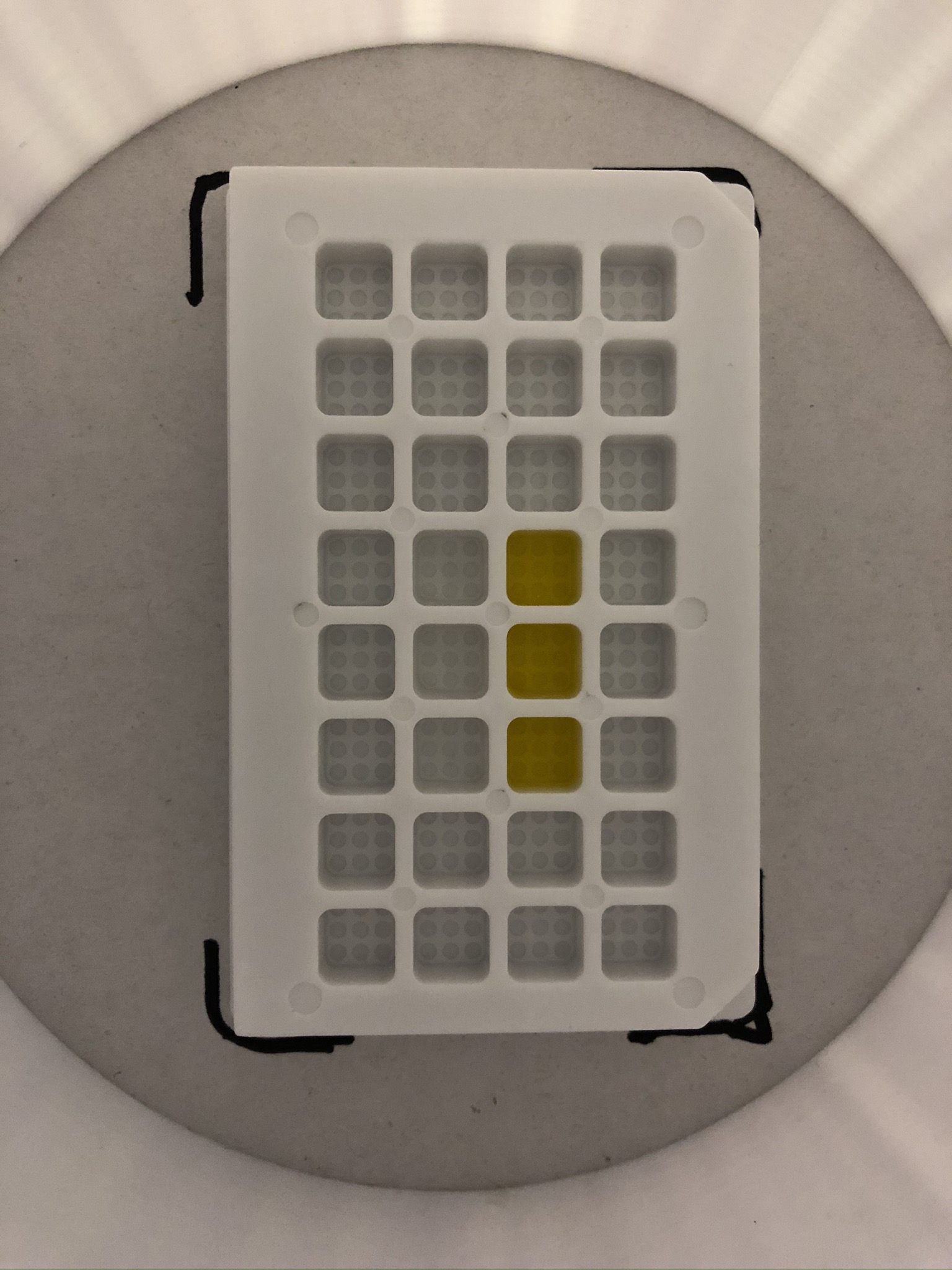

Supplement: Supplementary file 4 — Source Data [file 41467_2021_25989_MOESM4_ESM.zip › Image Files/Supp Fig 15/0min.JPEG]

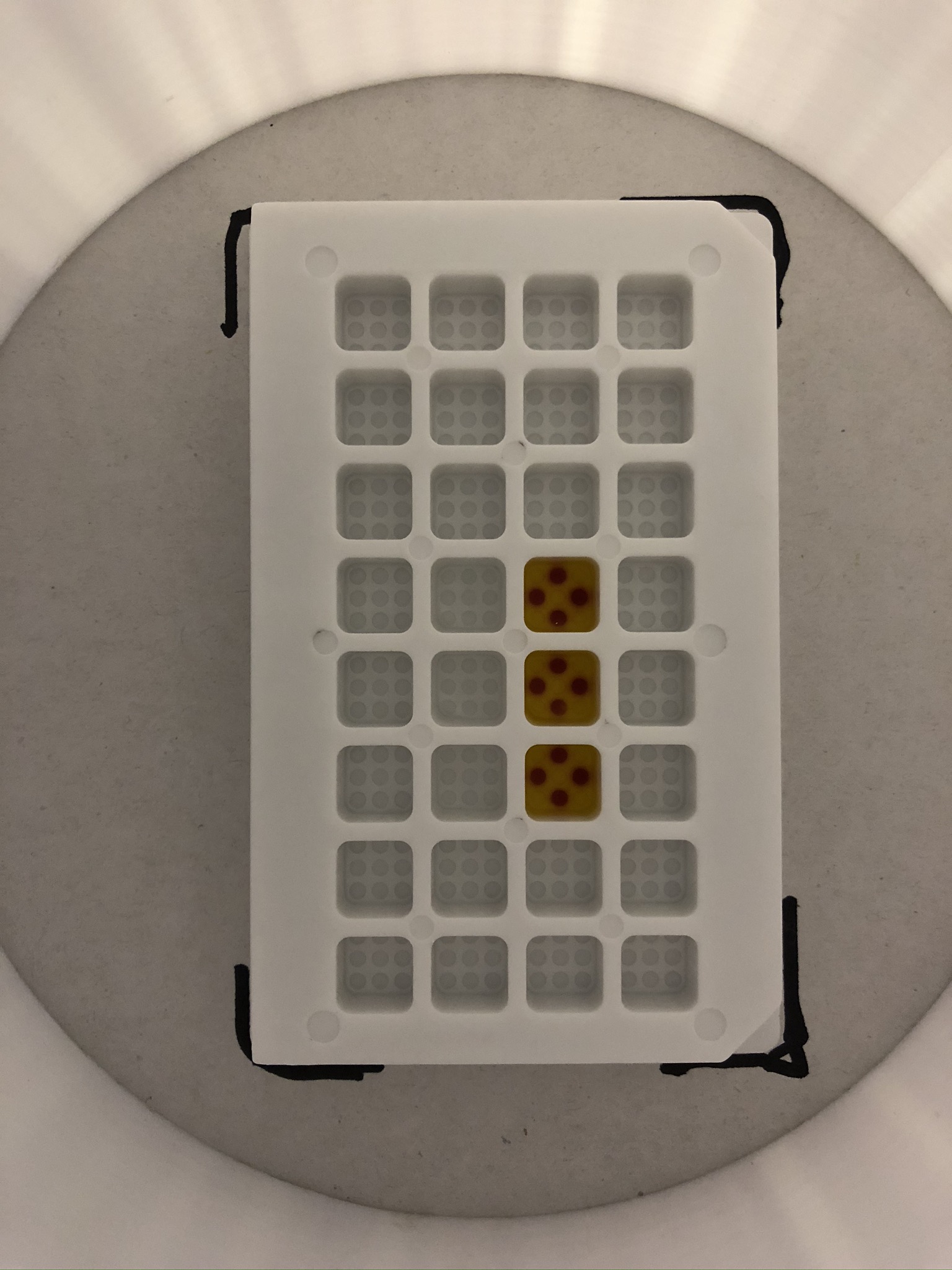

Supplement: Supplementary file 4 — Source Data [file 41467_2021_25989_MOESM4_ESM.zip › Image Files/Supp Fig 15/30min.JPEG]

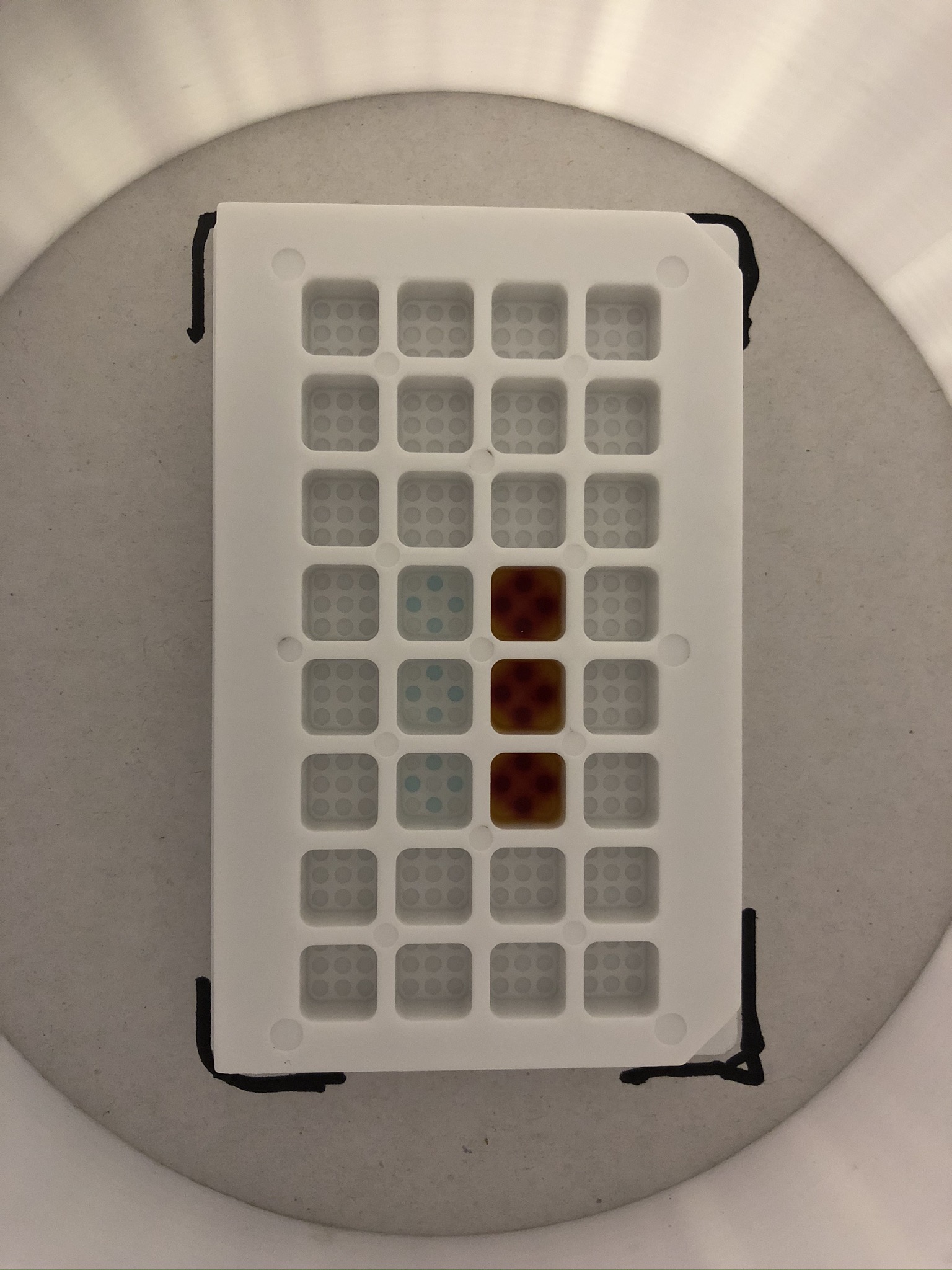

Supplement: Supplementary file 4 — Source Data [file 41467_2021_25989_MOESM4_ESM.zip › Image Files/Supp Fig 15/60min.JPEG]

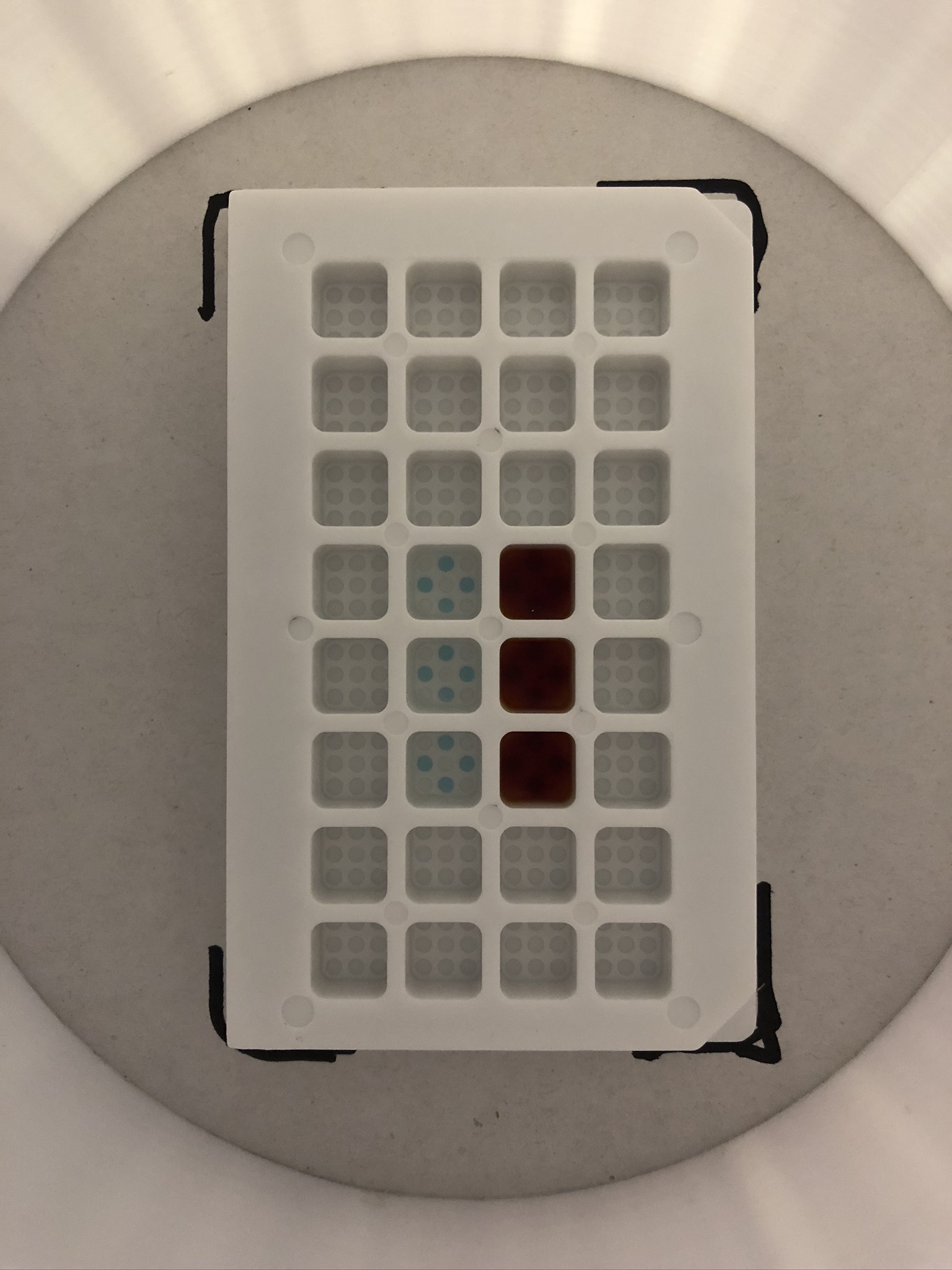

Supplement: Supplementary file 4 — Source Data [file 41467_2021_25989_MOESM4_ESM.zip › Image Files/Supp Fig 15/90min.JPEG]

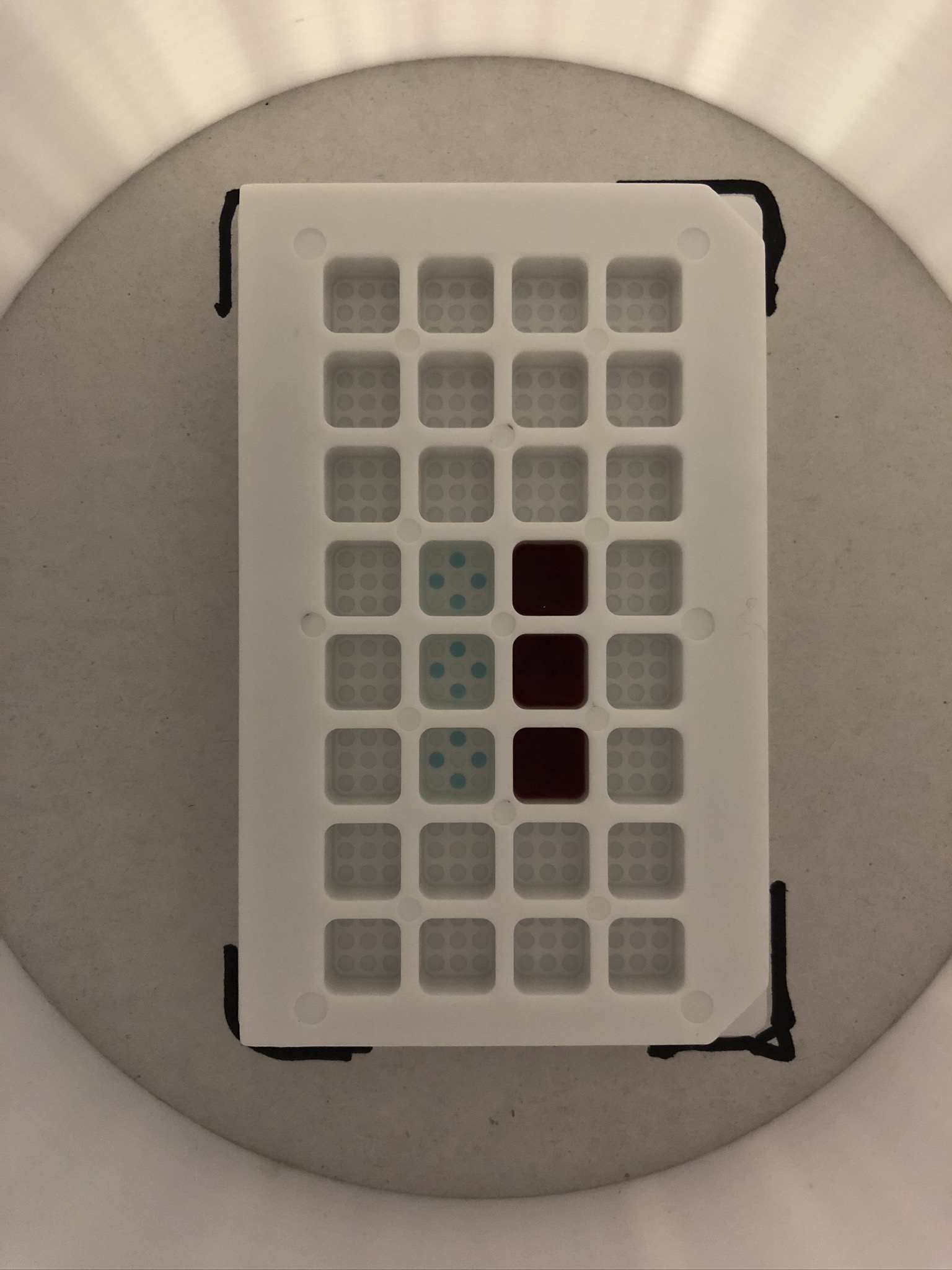

Supplement: Supplementary file 4 — Source Data [file 41467_2021_25989_MOESM4_ESM.zip › Image Files/Supp Fig 15/150min.JPEG]

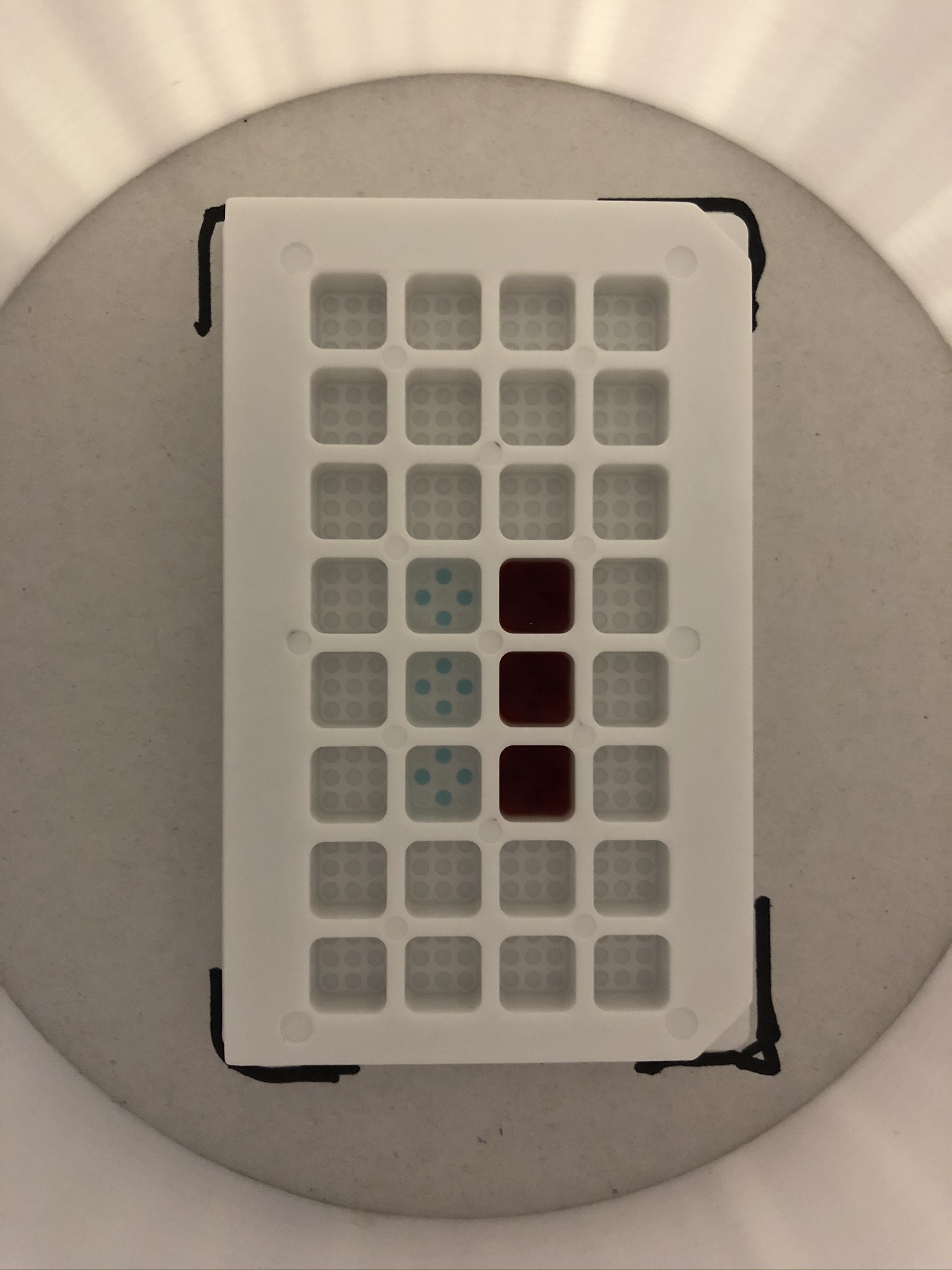

Supplement: Supplementary file 4 — Source Data [file 41467_2021_25989_MOESM4_ESM.zip › Image Files/Supp Fig 15/120min.JPEG]

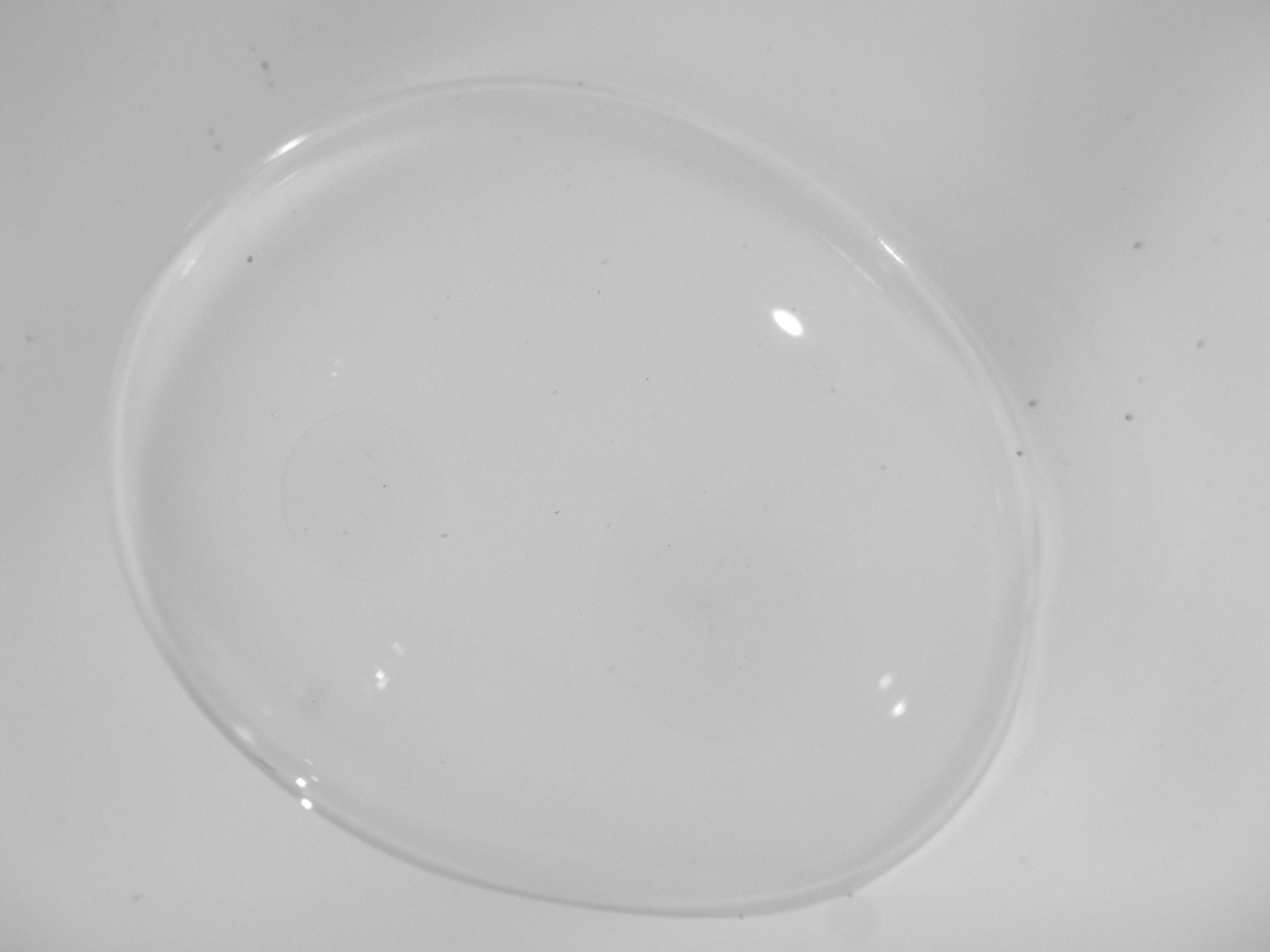

Supplement: Supplementary file 4 — Source Data [file 41467_2021_25989_MOESM4_ESM.zip › Image Files/Supp Fig 2A/Fic&Dex.tif]

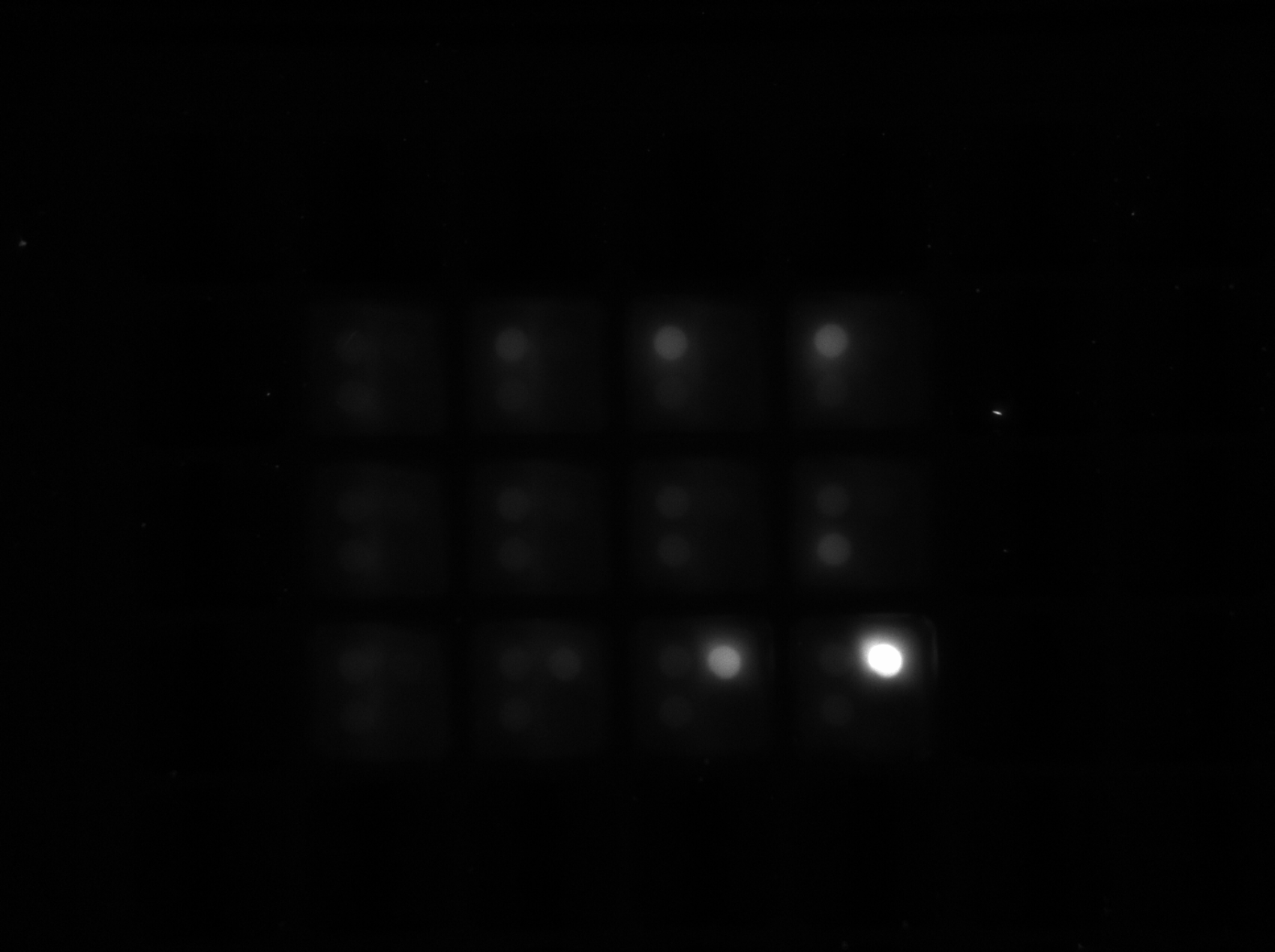

Supplement: Supplementary file 4 — Source Data [file 41467_2021_25989_MOESM4_ESM.zip › Image Files/Supp Fig 18/RPA_run2.tif]

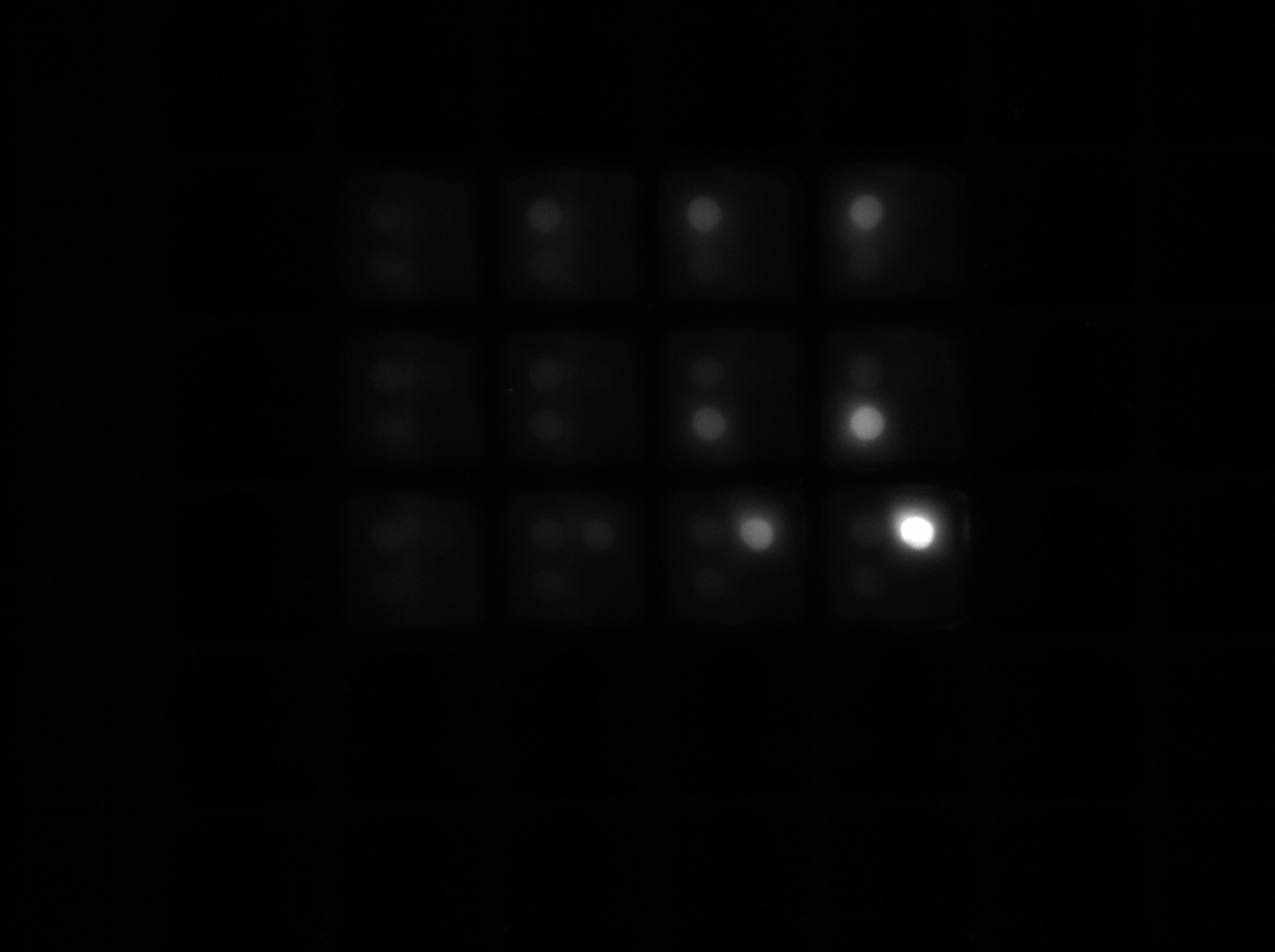

Supplement: Supplementary file 4 — Source Data [file 41467_2021_25989_MOESM4_ESM.zip › Image Files/Supp Fig 18/RPA_run1.tif]

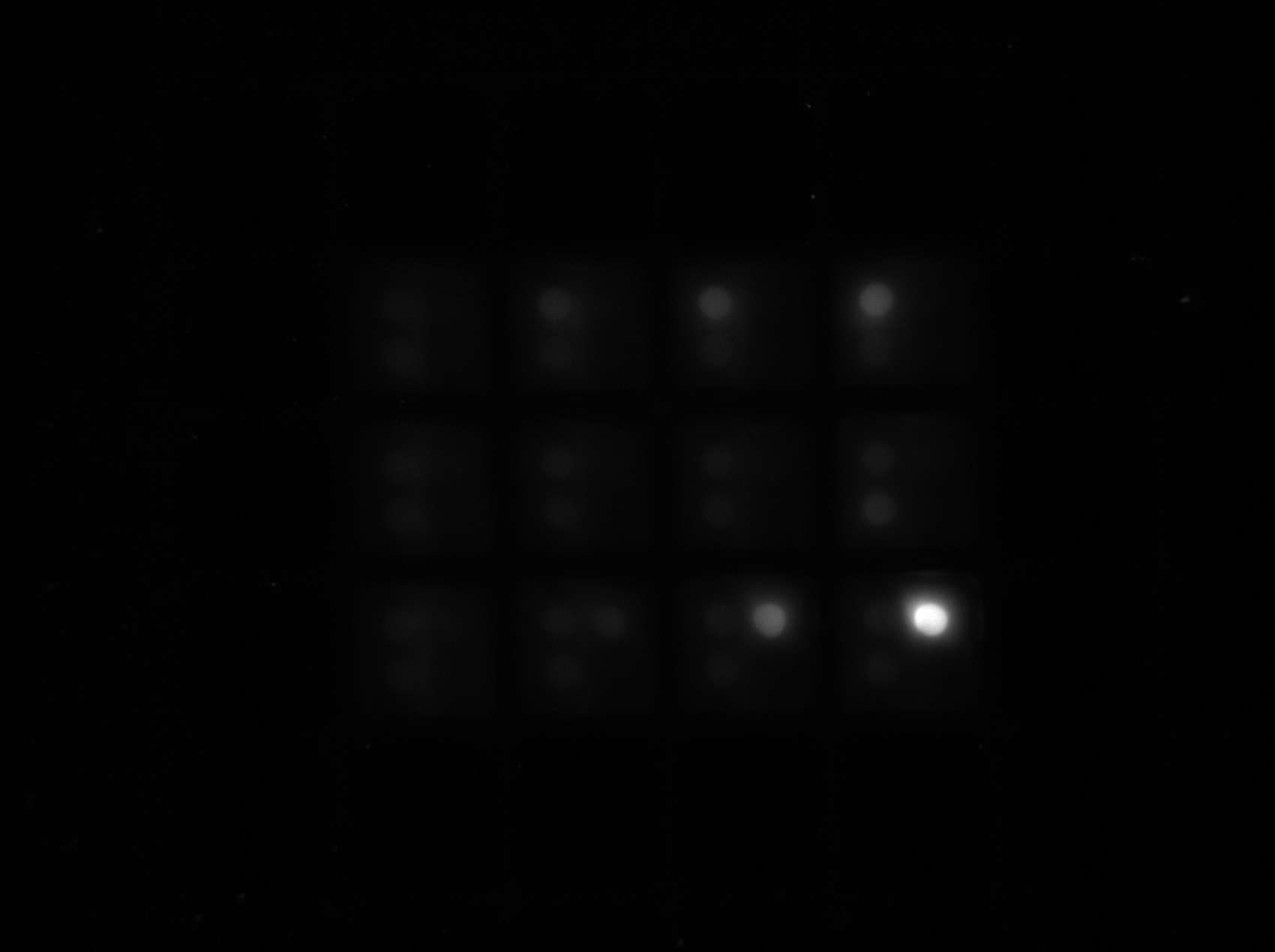

Supplement: Supplementary file 4 — Source Data [file 41467_2021_25989_MOESM4_ESM.zip › Image Files/Supp Fig 18/RPA_run3.tif]

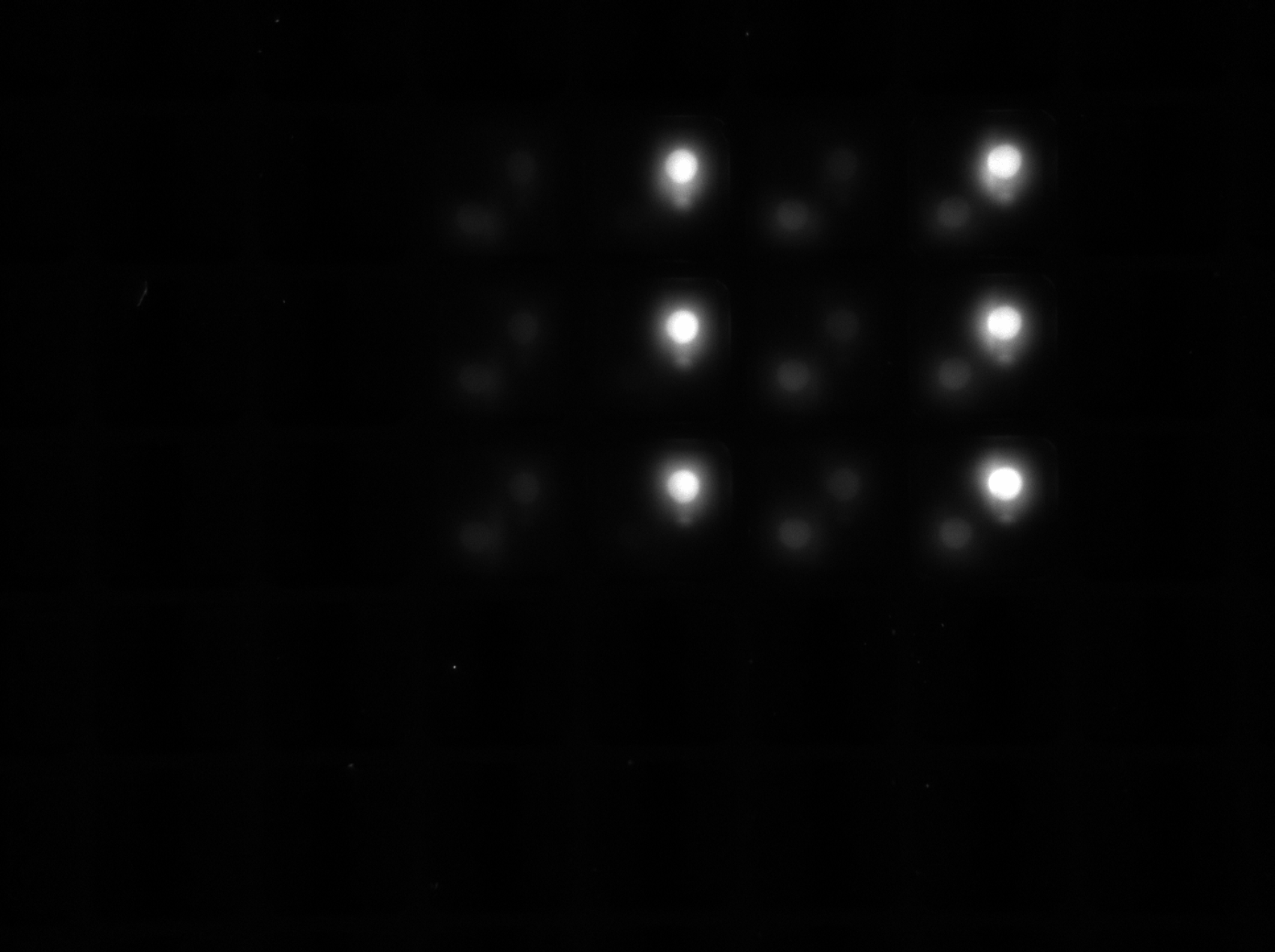

Supplement: Supplementary file 4 — Source Data [file 41467_2021_25989_MOESM4_ESM.zip › Image Files/Supp Fig 9B/ZnB12_Med.tif]

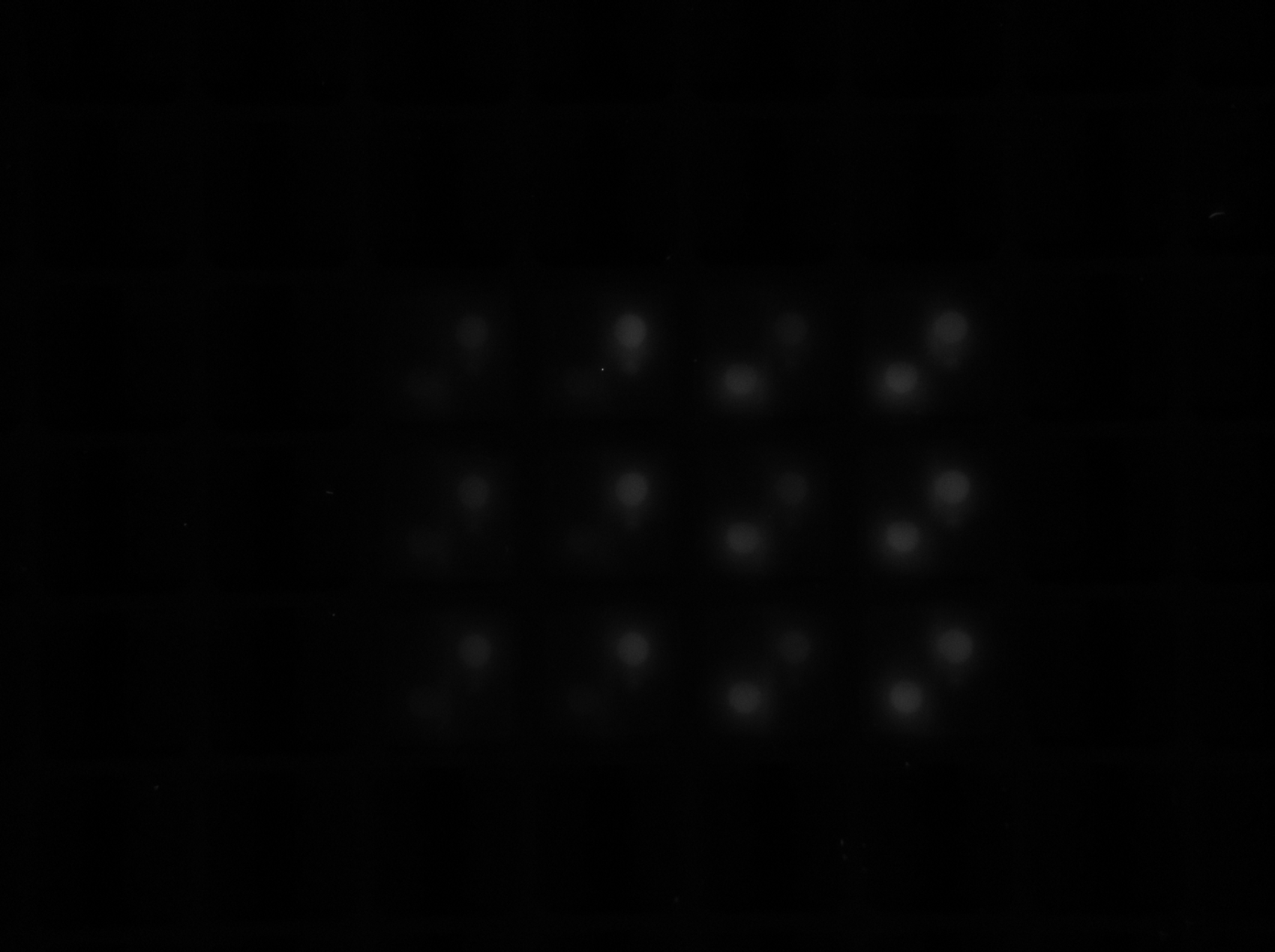

Supplement: Supplementary file 4 — Source Data [file 41467_2021_25989_MOESM4_ESM.zip › Image Files/Supp Fig 9B/ZnB12_Low.tif]

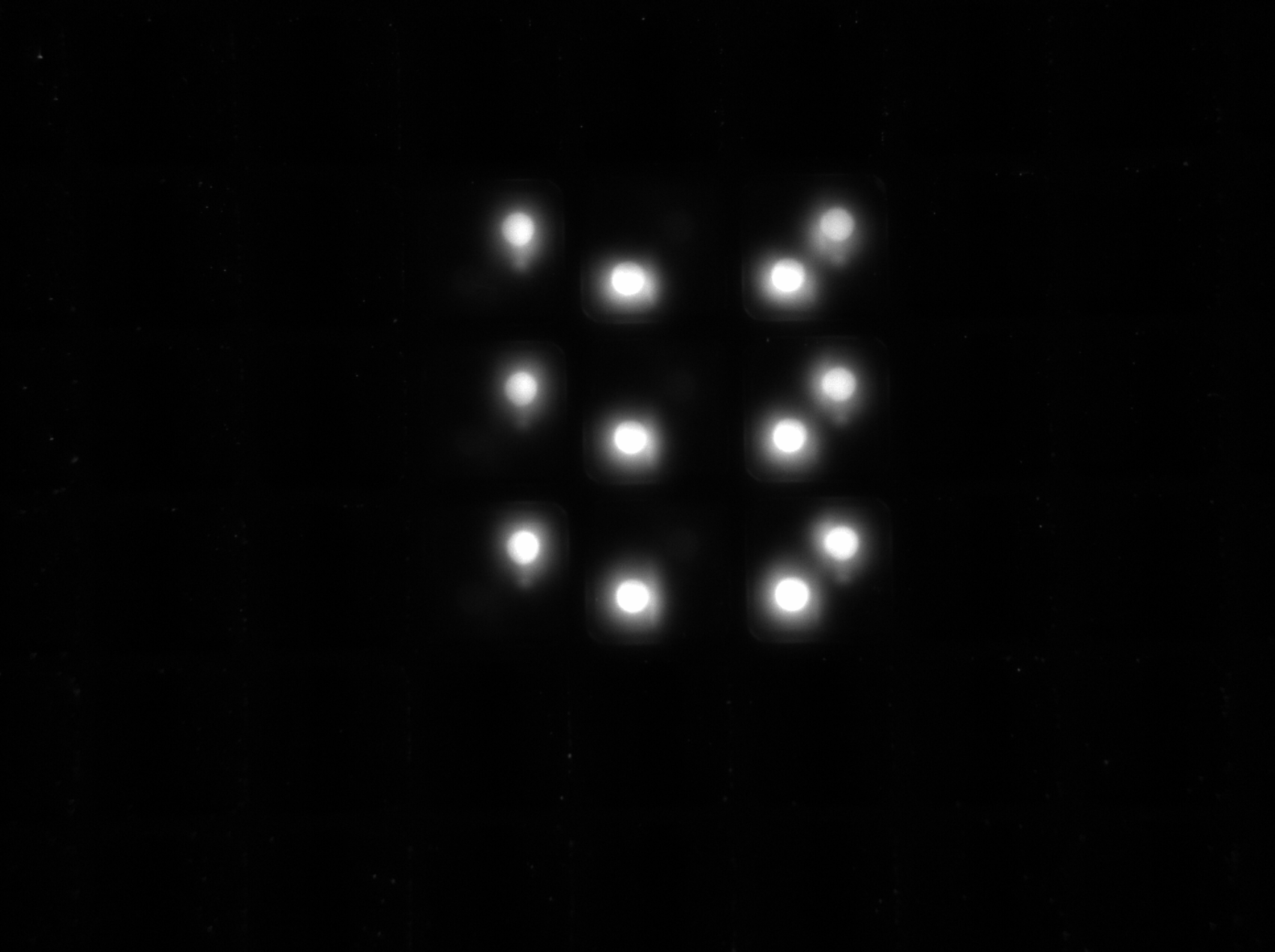

Supplement: Supplementary file 4 — Source Data [file 41467_2021_25989_MOESM4_ESM.zip › Image Files/Supp Fig 9B/ZnB12_High.tif]

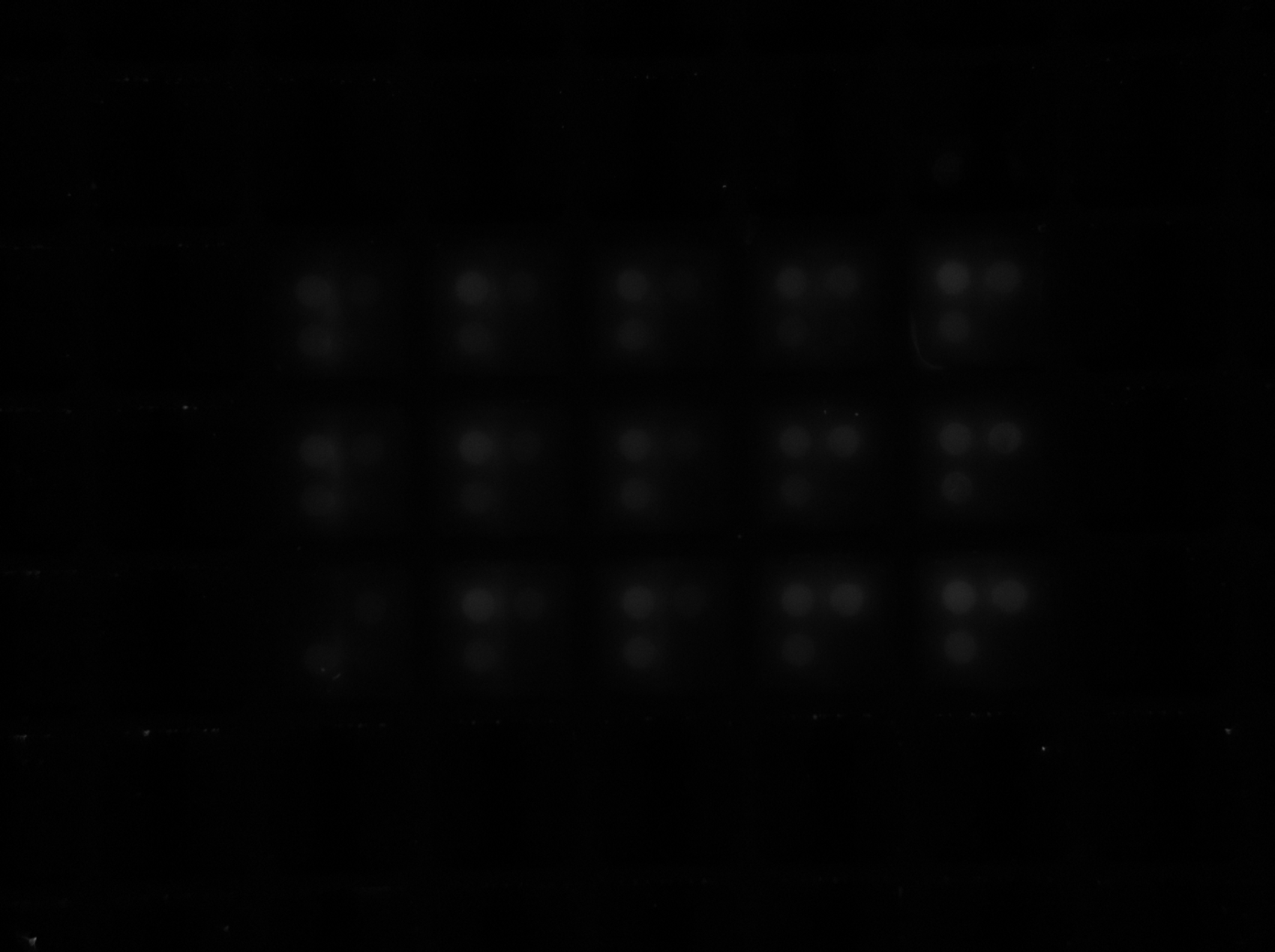

Supplement: Supplementary file 4 — Source Data [file 41467_2021_25989_MOESM4_ESM.zip › Image Files/Supp Fig 10B/Patho_2nM.tif]

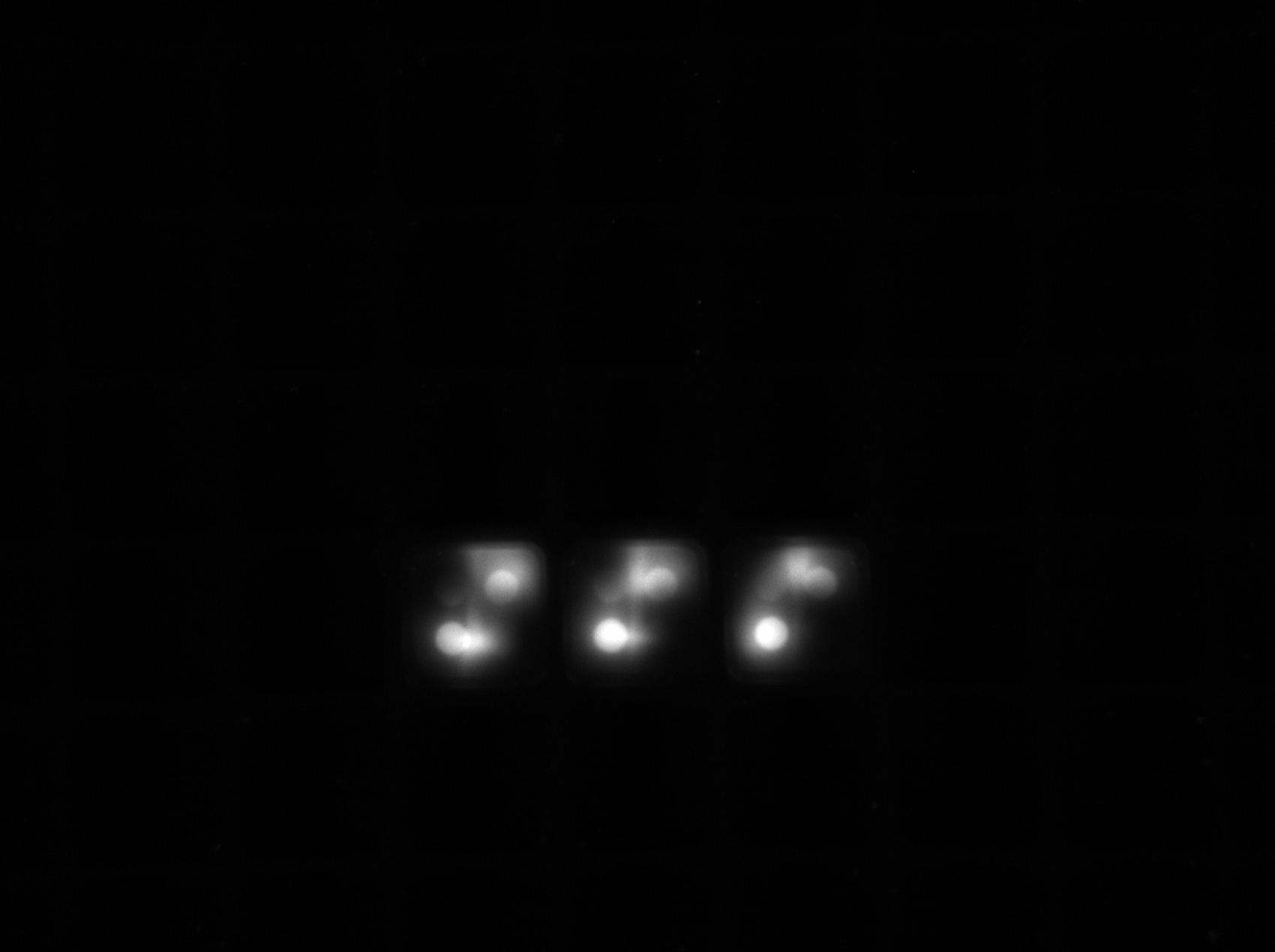

Supplement: Supplementary file 4 — Source Data [file 41467_2021_25989_MOESM4_ESM.zip › Image Files/Fig 2C/3hr_FicDex_day3.tif]

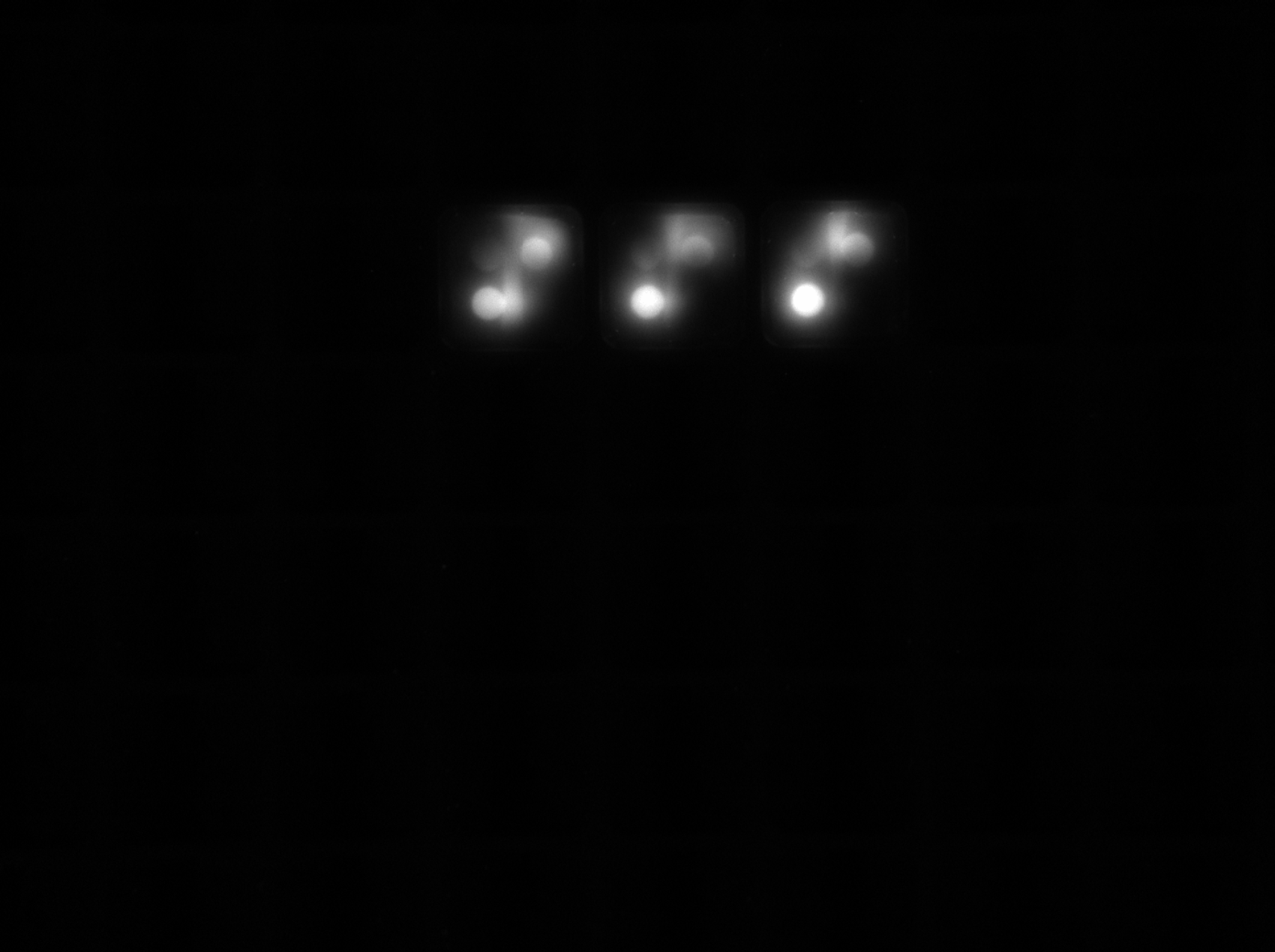

Supplement: Supplementary file 4 — Source Data [file 41467_2021_25989_MOESM4_ESM.zip › Image Files/Fig 2C/3hr_FicDex_day2.tif]

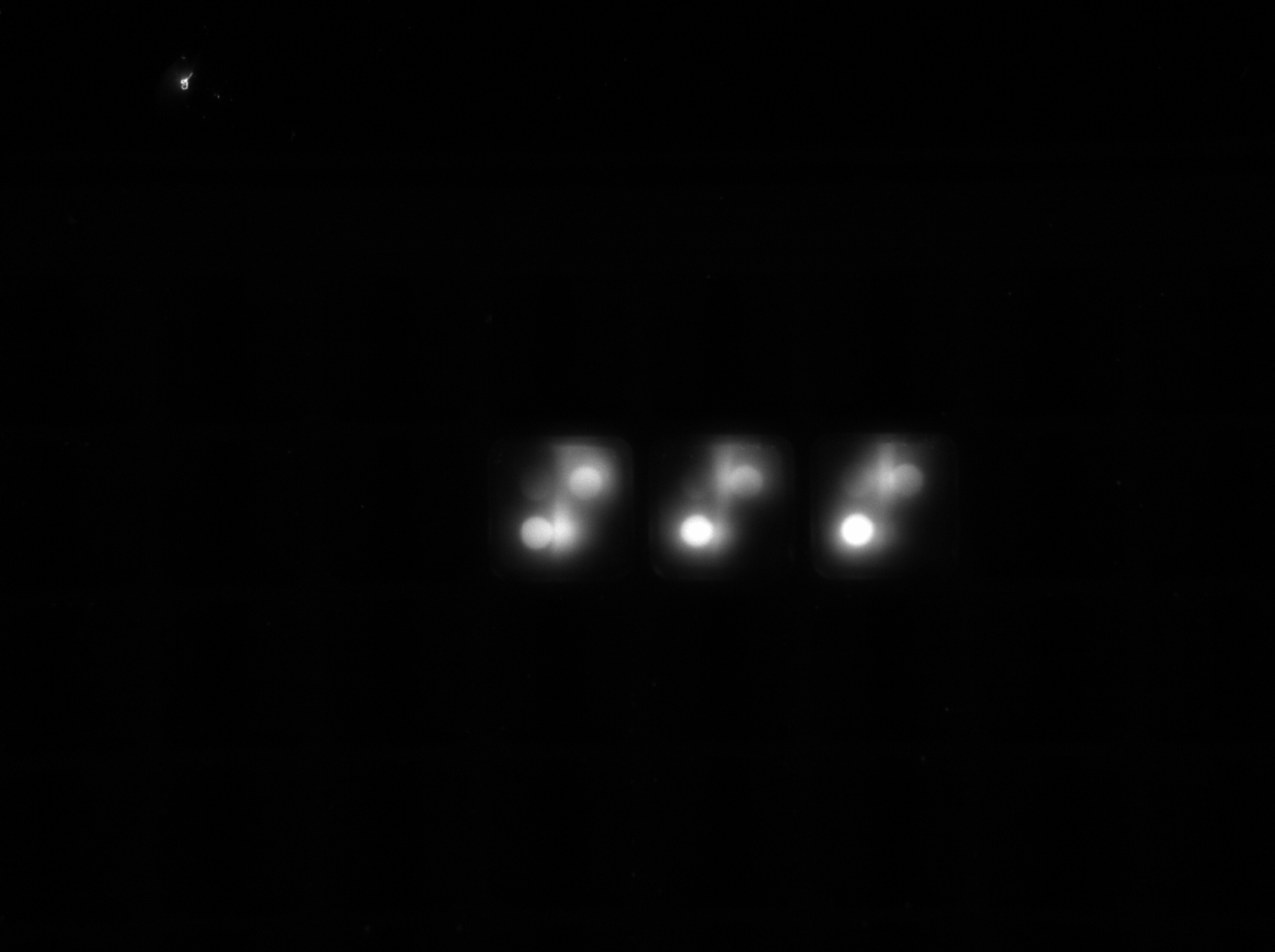

Supplement: Supplementary file 4 — Source Data [file 41467_2021_25989_MOESM4_ESM.zip › Image Files/Fig 2C/3hr_FicDex_day1.tif]

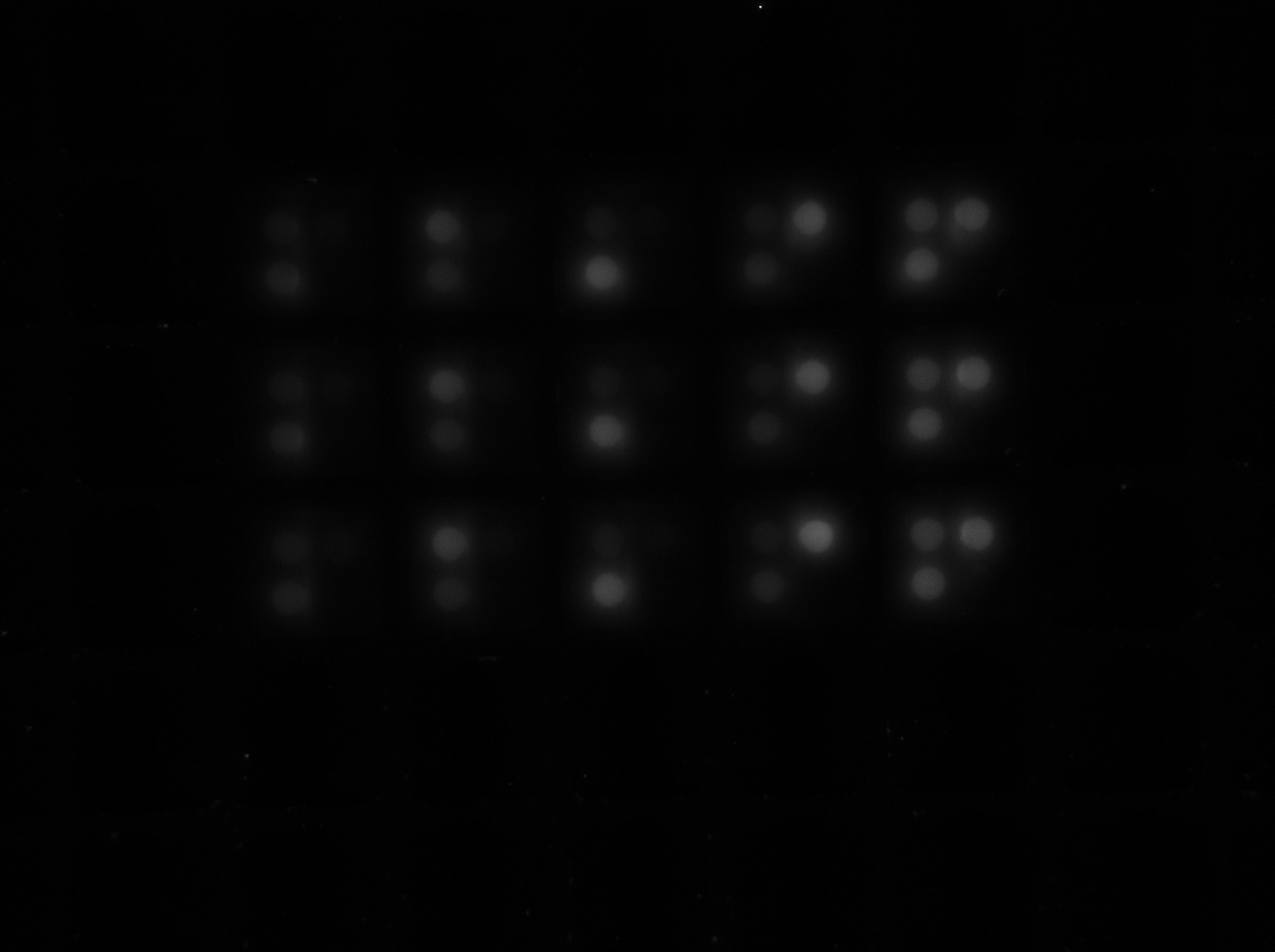

Supplement: Supplementary file 4 — Source Data [file 41467_2021_25989_MOESM4_ESM.zip › Image Files/Supp Fig 10B/Patho_20nM.tif]

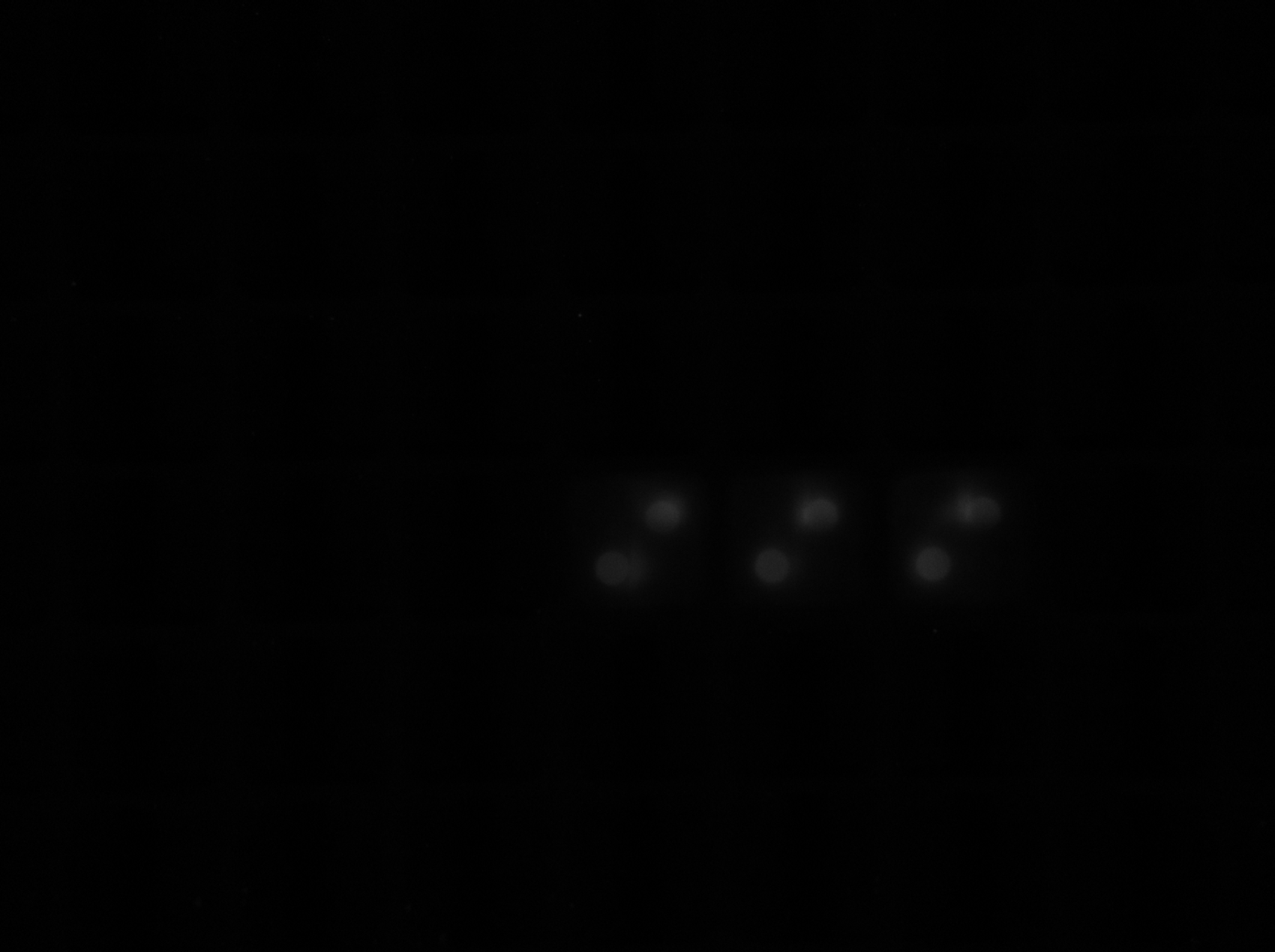

Supplement: Supplementary file 4 — Source Data [file 41467_2021_25989_MOESM4_ESM.zip › Image Files/Supp Fig 2E/1hr/Day3_1hr.tif]

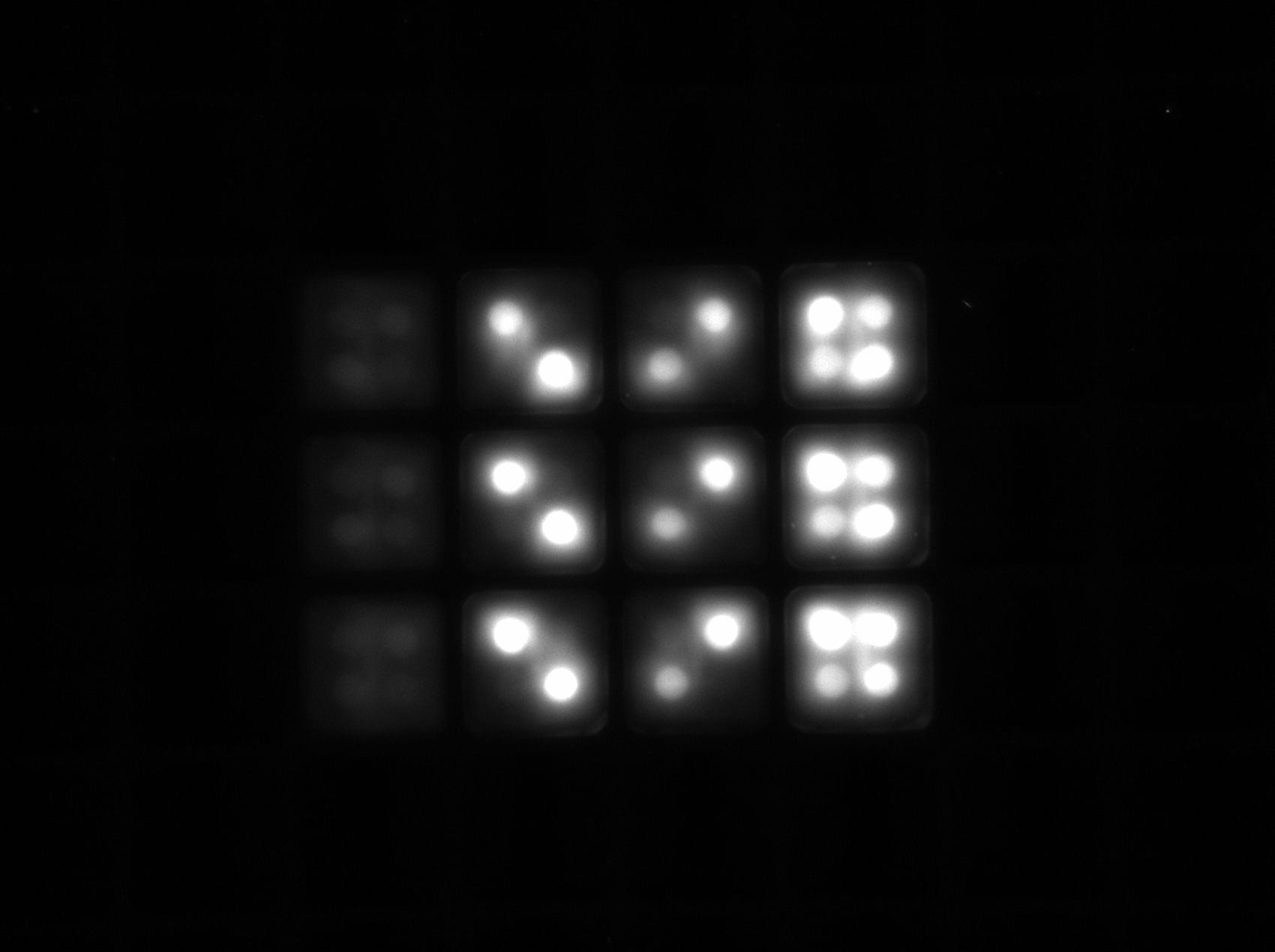

Supplement: Supplementary file 4 — Source Data [file 41467_2021_25989_MOESM4_ESM.zip › Image Files/Supp Fig 12/Serum10_day3.tif]

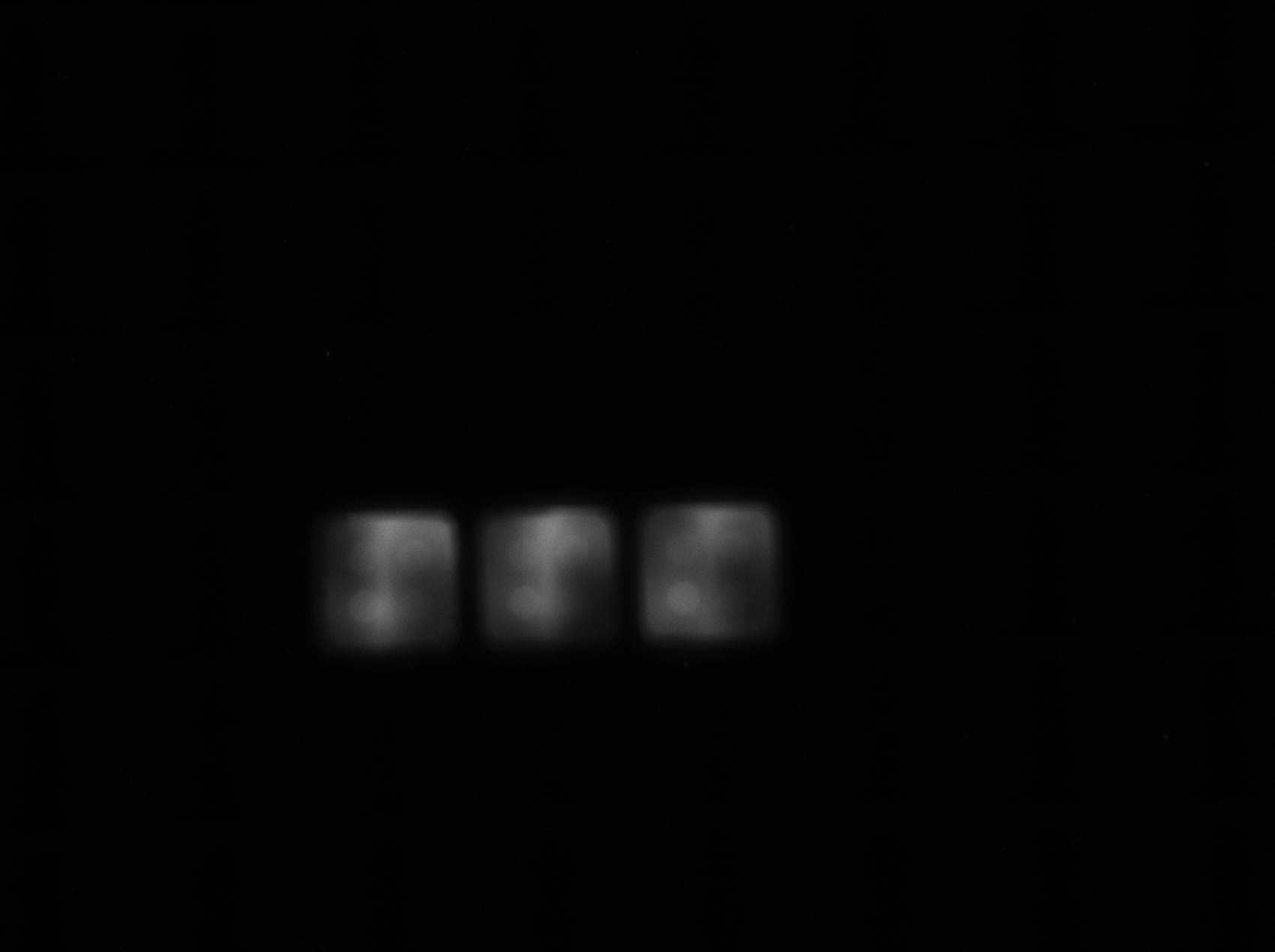

Supplement: Supplementary file 4 — Source Data [file 41467_2021_25989_MOESM4_ESM.zip › Image Files/Supp Fig 2E/8hr/Day3_8hr.tif]

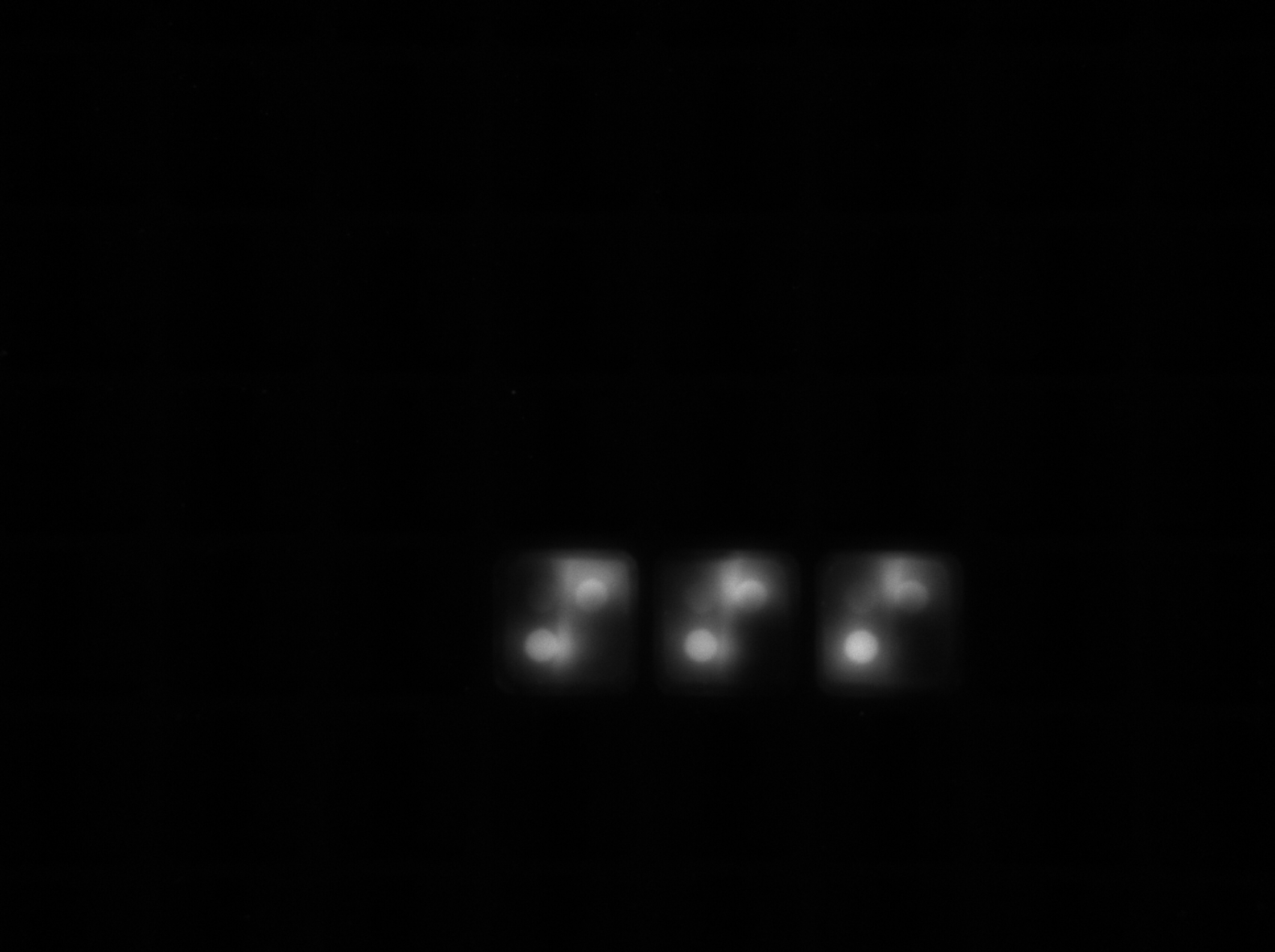

Supplement: Supplementary file 4 — Source Data [file 41467_2021_25989_MOESM4_ESM.zip › Image Files/Supp Fig 2E/4hr/Day3_4hr.tif]

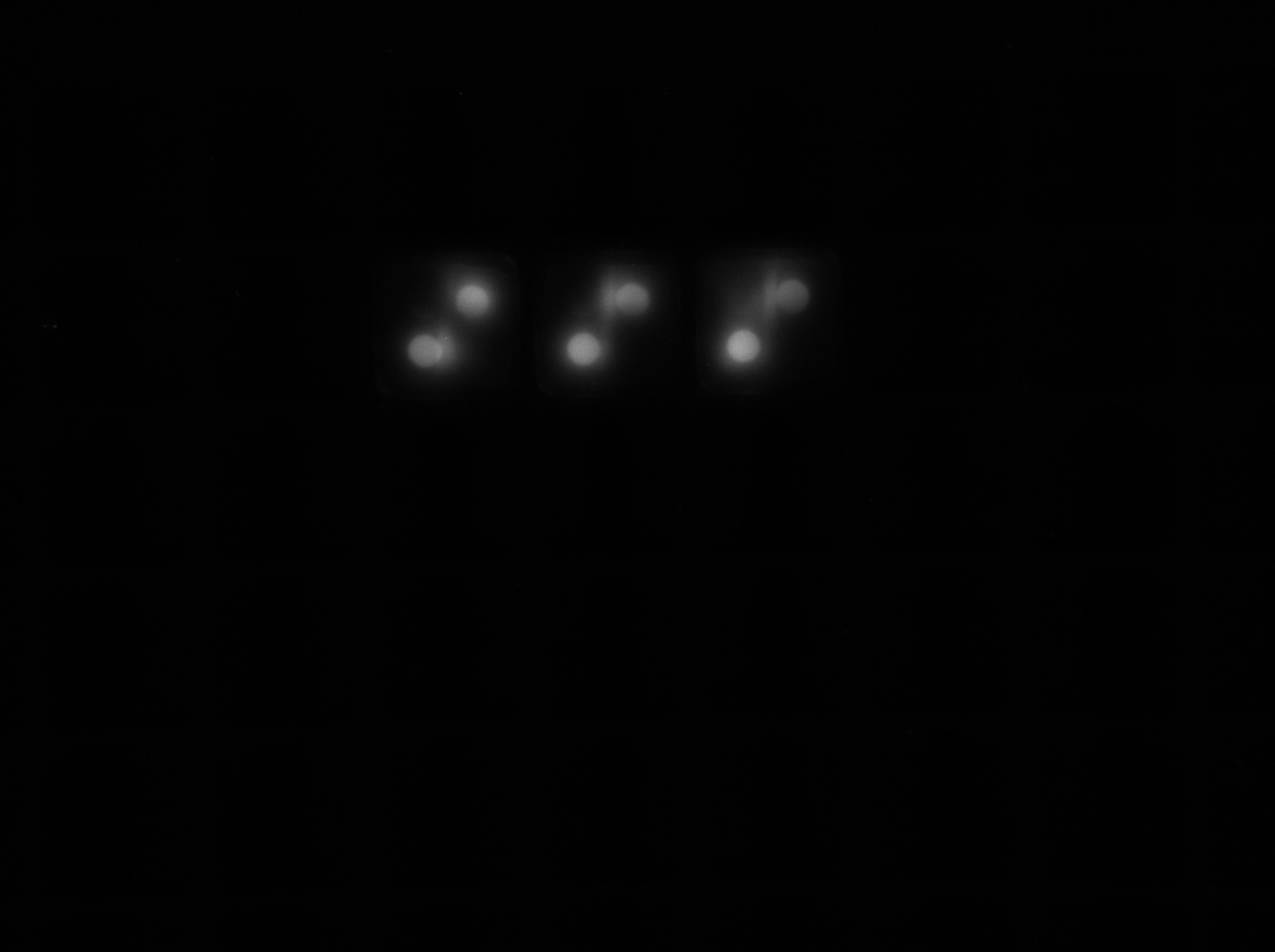

Supplement: Supplementary file 4 — Source Data [file 41467_2021_25989_MOESM4_ESM.zip › Image Files/Supp Fig 2E/2hr/Day1_2hr.tif]

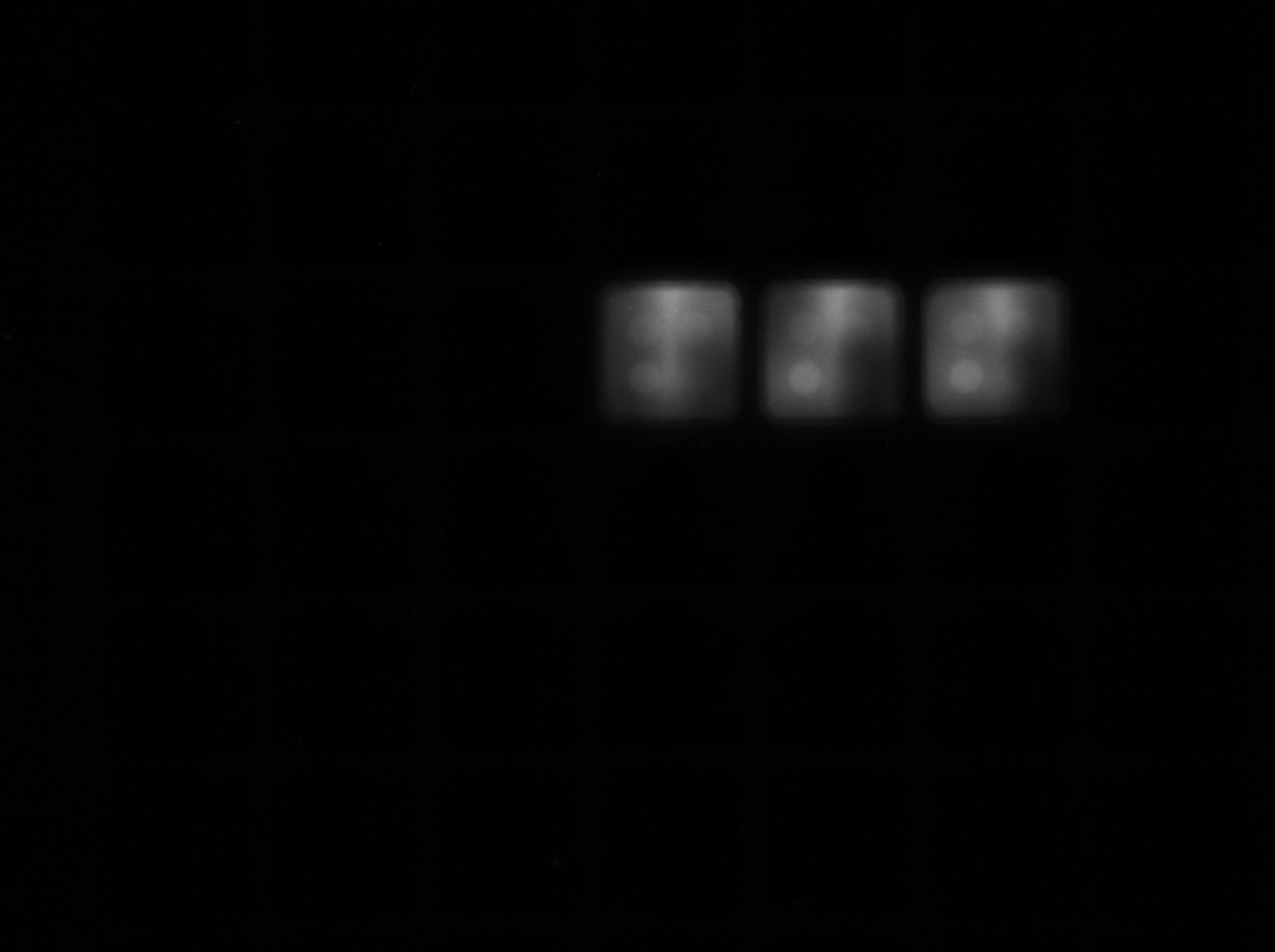

Supplement: Supplementary file 4 — Source Data [file 41467_2021_25989_MOESM4_ESM.zip › Image Files/Supp Fig 2E/8hr/Day2_8hr.tif]

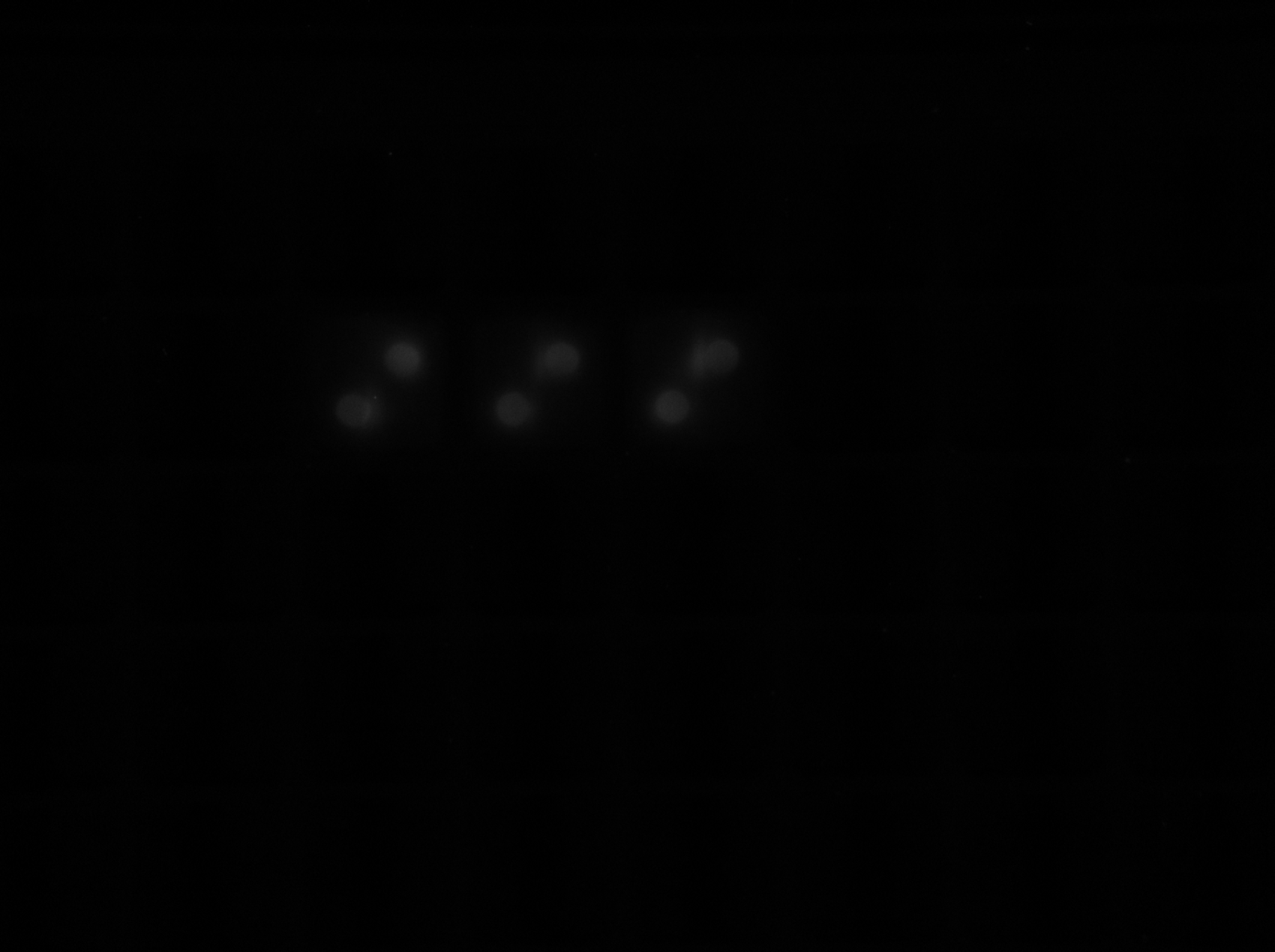

Supplement: Supplementary file 4 — Source Data [file 41467_2021_25989_MOESM4_ESM.zip › Image Files/Supp Fig 2E/1hr/Day1_1hr.tif]

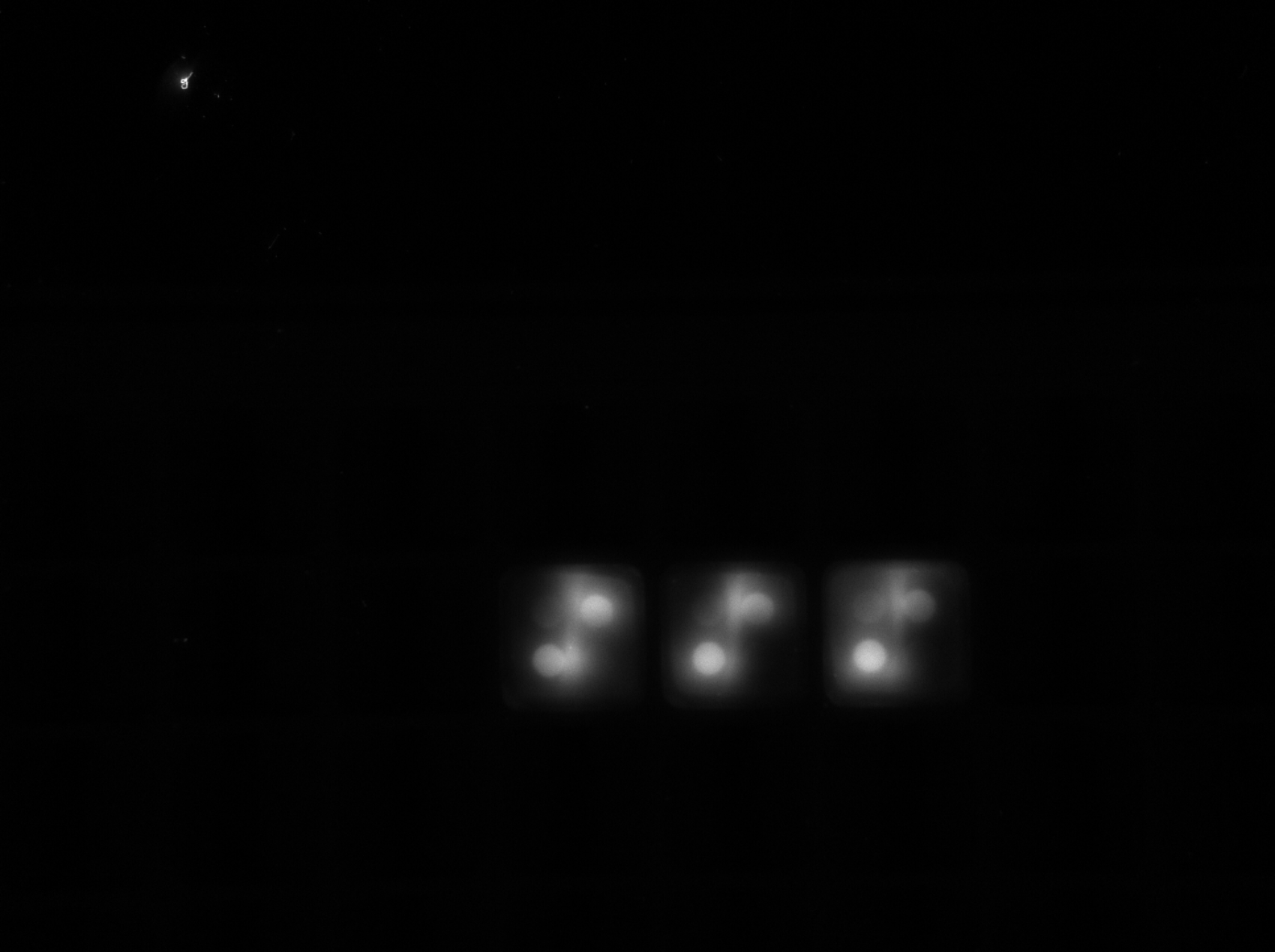

Supplement: Supplementary file 4 — Source Data [file 41467_2021_25989_MOESM4_ESM.zip › Image Files/Supp Fig 2E/4hr/Day1_4hr.tif]

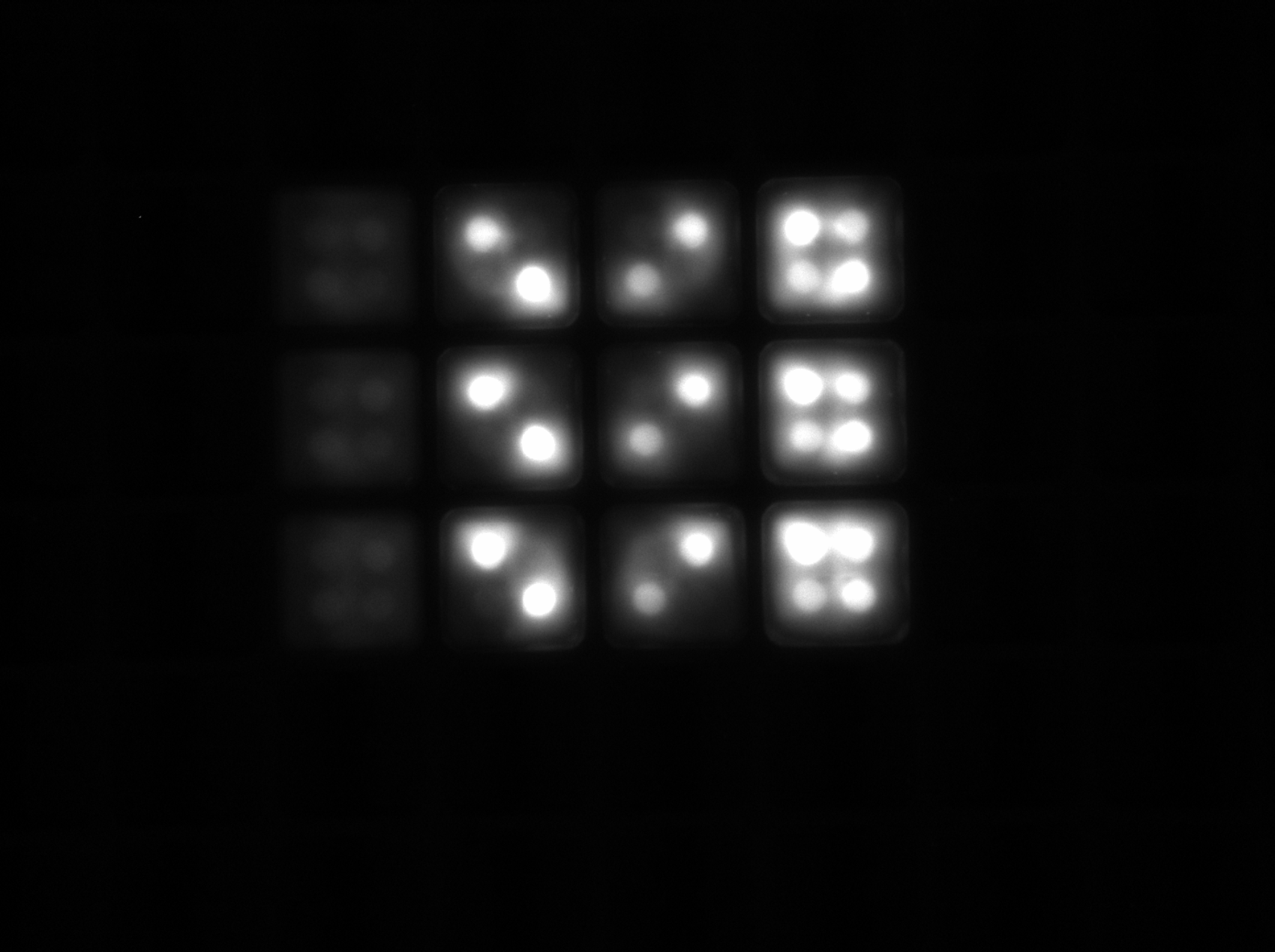

Supplement: Supplementary file 4 — Source Data [file 41467_2021_25989_MOESM4_ESM.zip › Image Files/Supp Fig 12/Serum10_day1.tif]

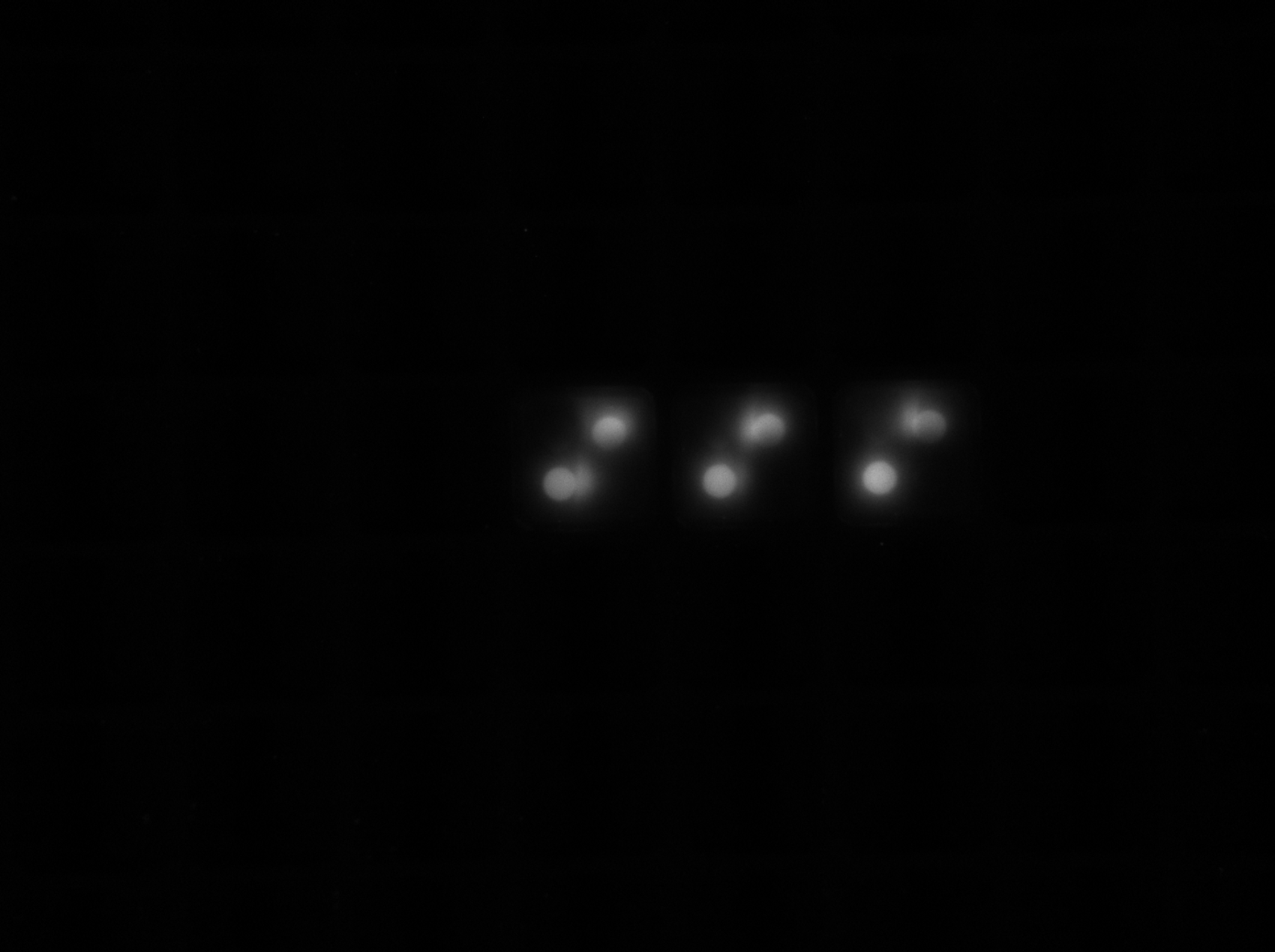

Supplement: Supplementary file 4 — Source Data [file 41467_2021_25989_MOESM4_ESM.zip › Image Files/Supp Fig 2E/2hr/Day3_2hr.tif]

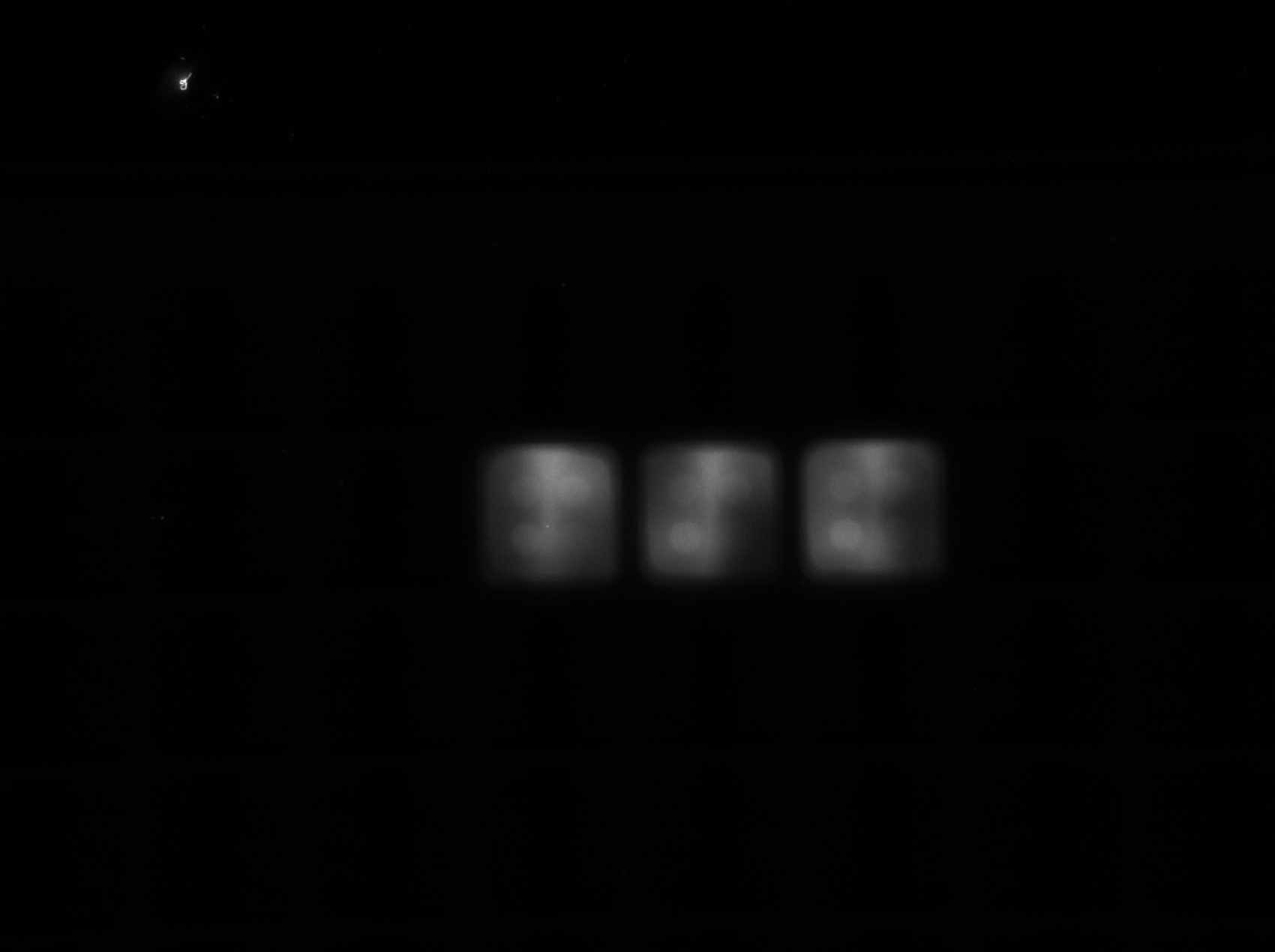

Supplement: Supplementary file 4 — Source Data [file 41467_2021_25989_MOESM4_ESM.zip › Image Files/Supp Fig 2E/8hr/Day1_8hr.tif]

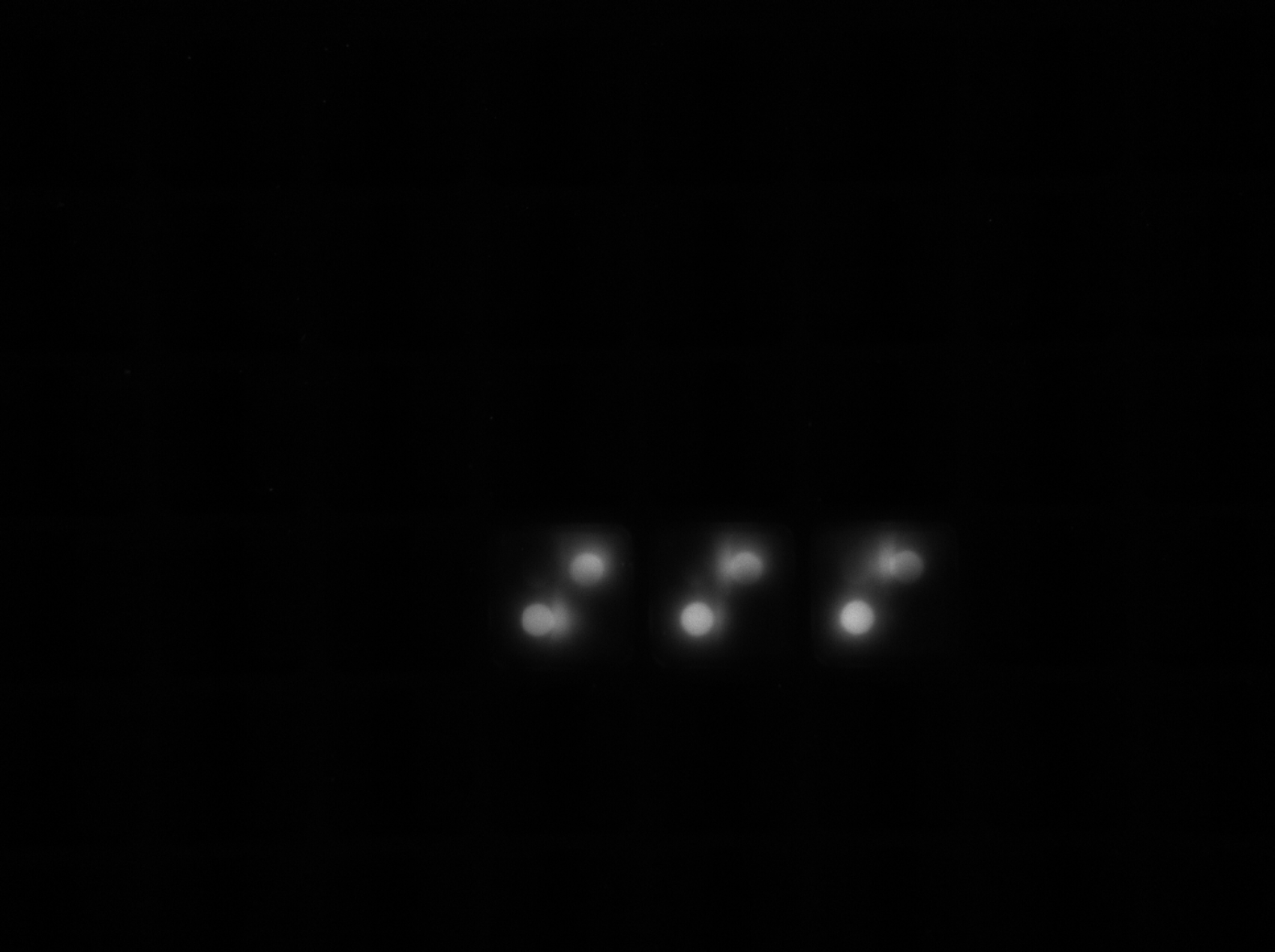

Supplement: Supplementary file 4 — Source Data [file 41467_2021_25989_MOESM4_ESM.zip › Image Files/Supp Fig 2E/2hr/Day2_2hr.tif]

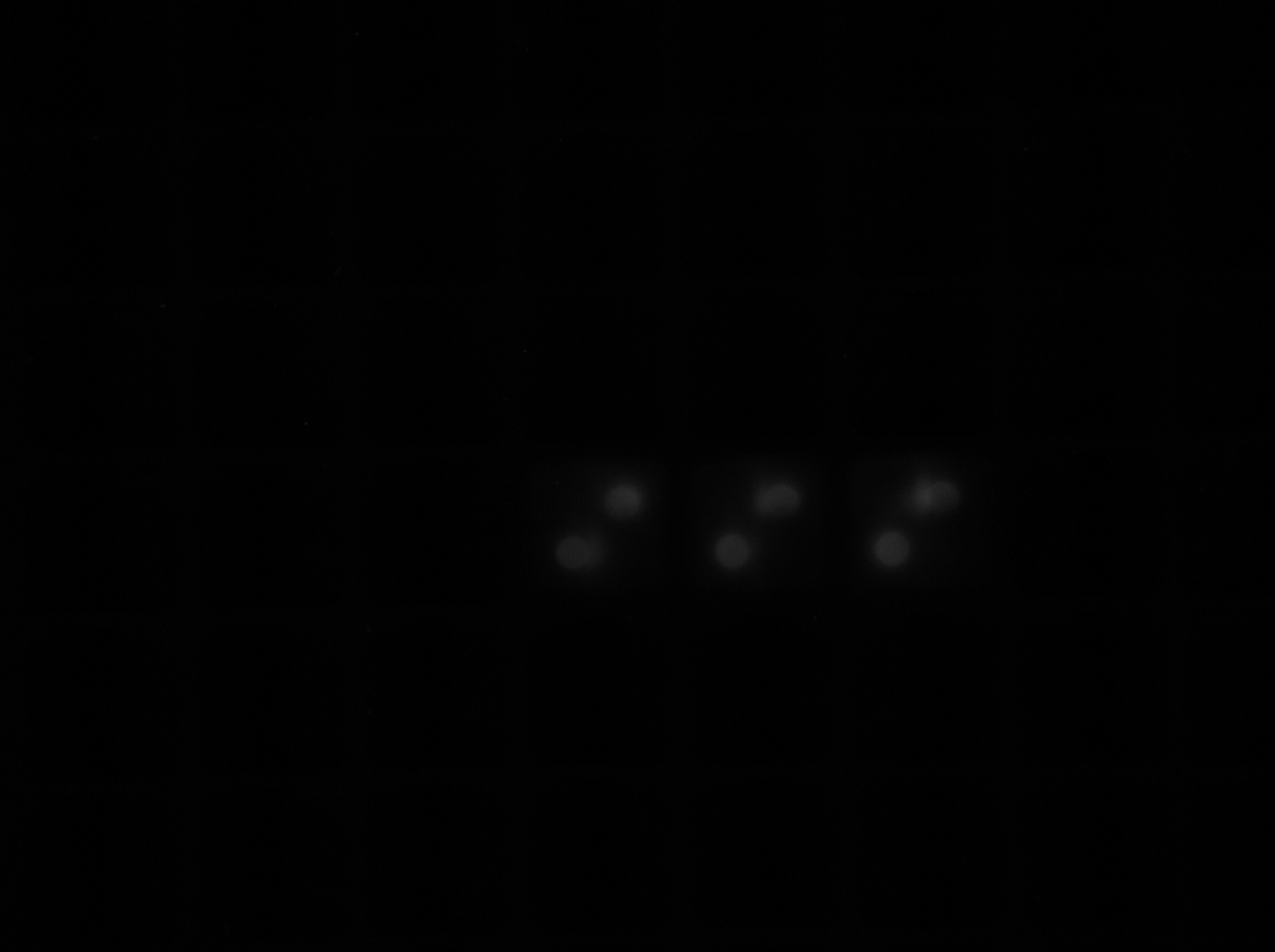

Supplement: Supplementary file 4 — Source Data [file 41467_2021_25989_MOESM4_ESM.zip › Image Files/Supp Fig 2E/1hr/Day2_1hr.tif]

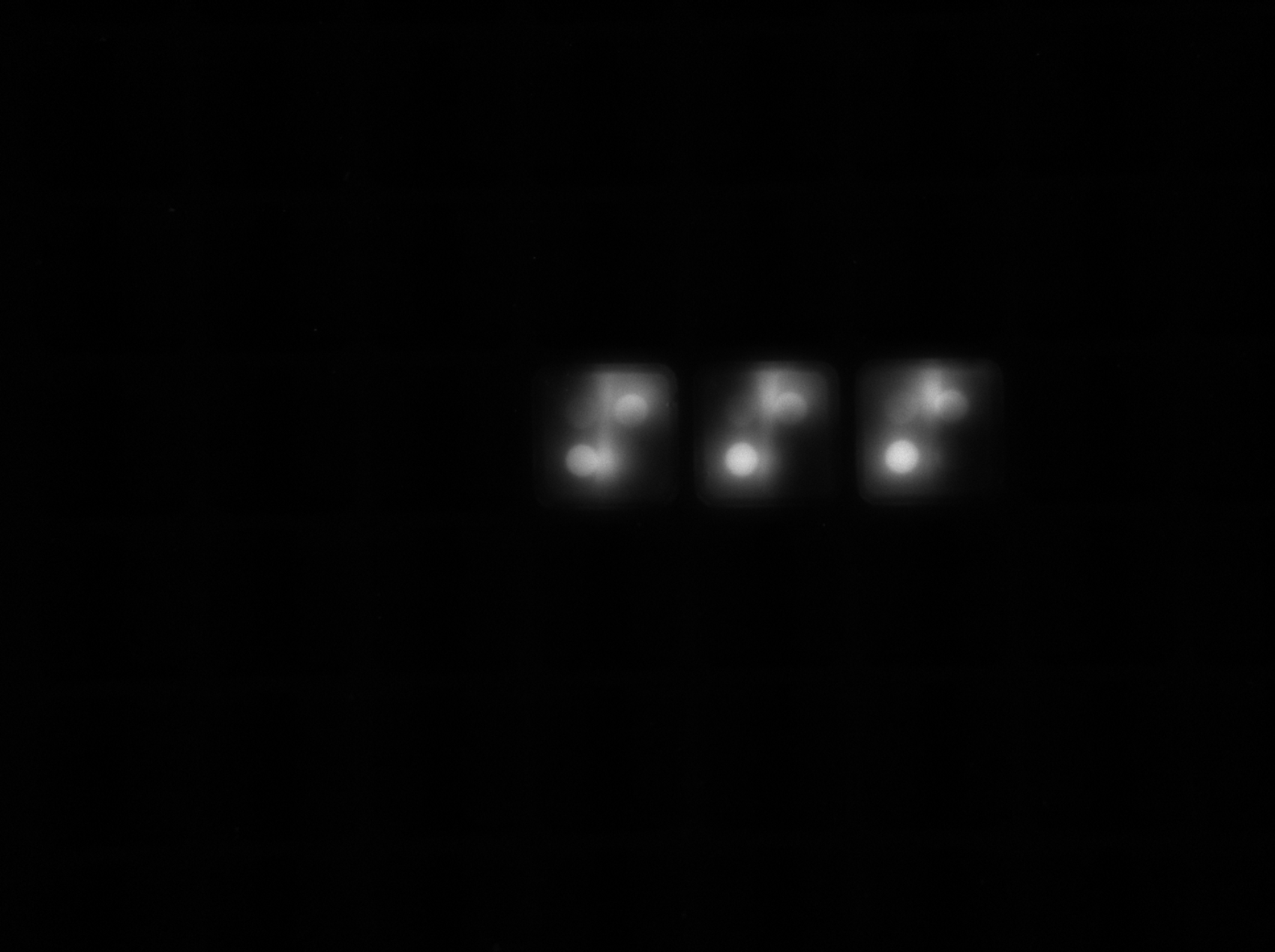

Supplement: Supplementary file 4 — Source Data [file 41467_2021_25989_MOESM4_ESM.zip › Image Files/Supp Fig 2E/4hr/Day2_4hr.tif]

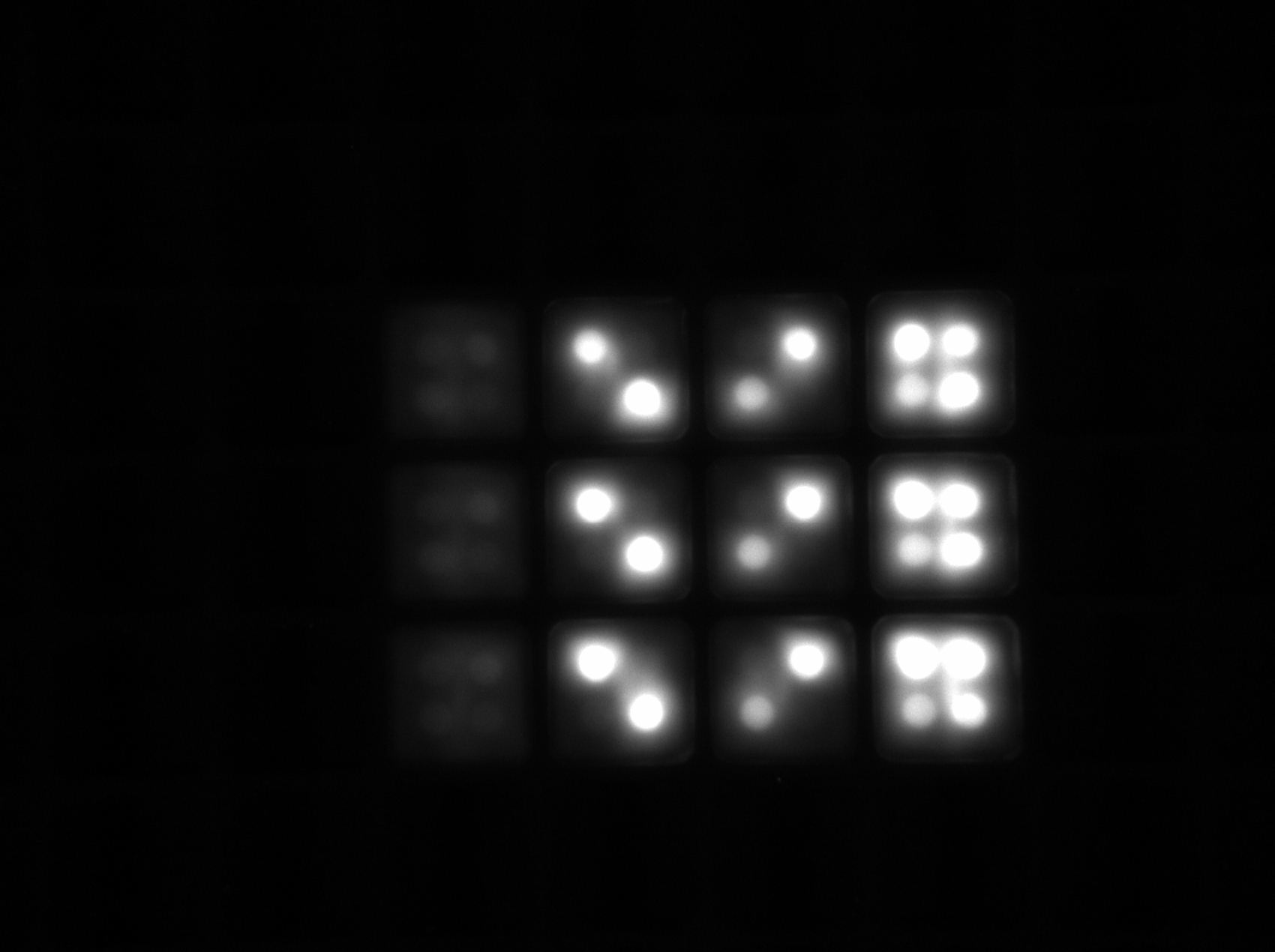

Supplement: Supplementary file 4 — Source Data [file 41467_2021_25989_MOESM4_ESM.zip › Image Files/Supp Fig 12/Serum10_day2.tif]

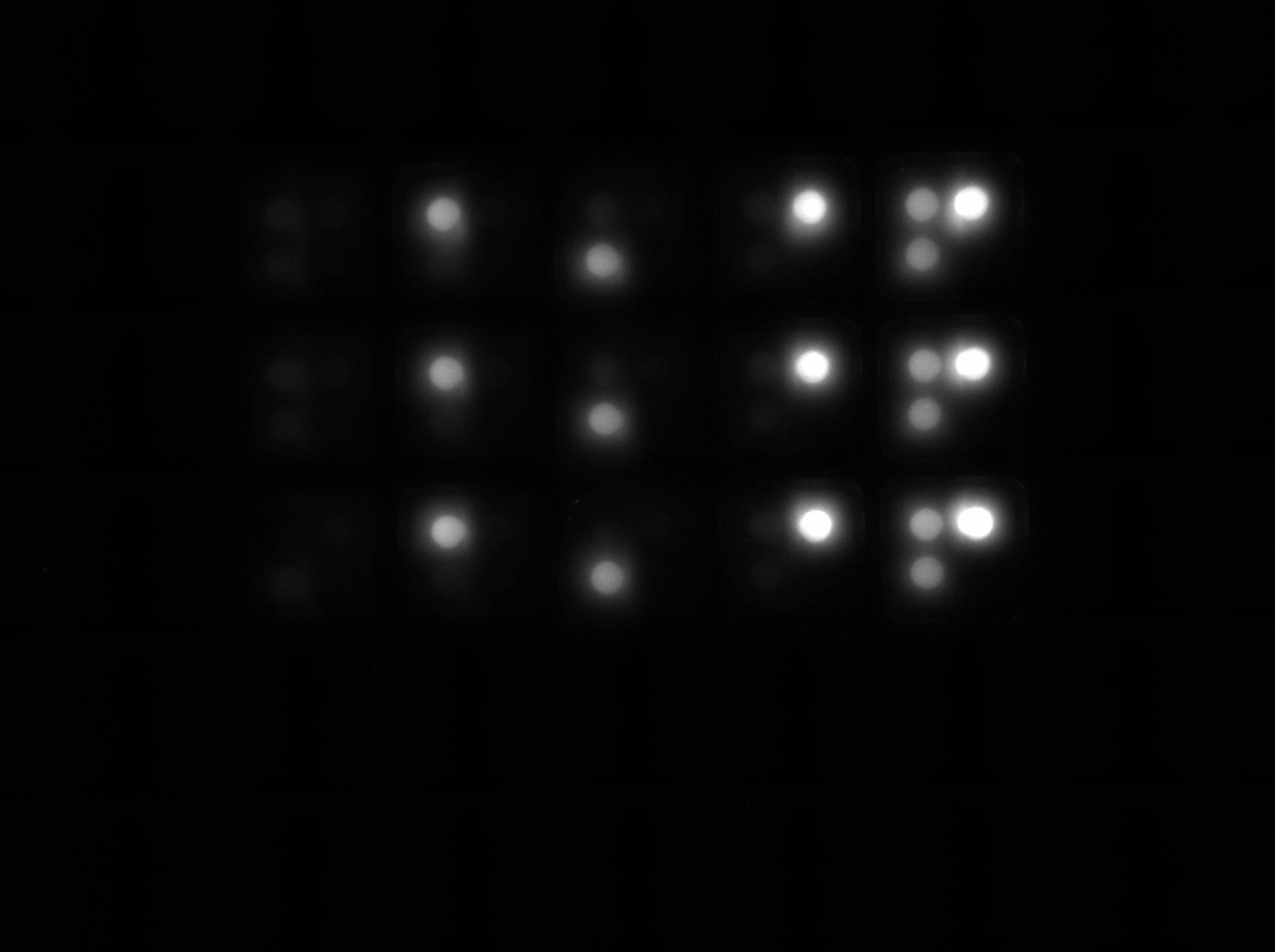

Supplement: Supplementary file 4 — Source Data [file 41467_2021_25989_MOESM4_ESM.zip › Image Files/Supp Fig 10B/Patho_100nM.tif]

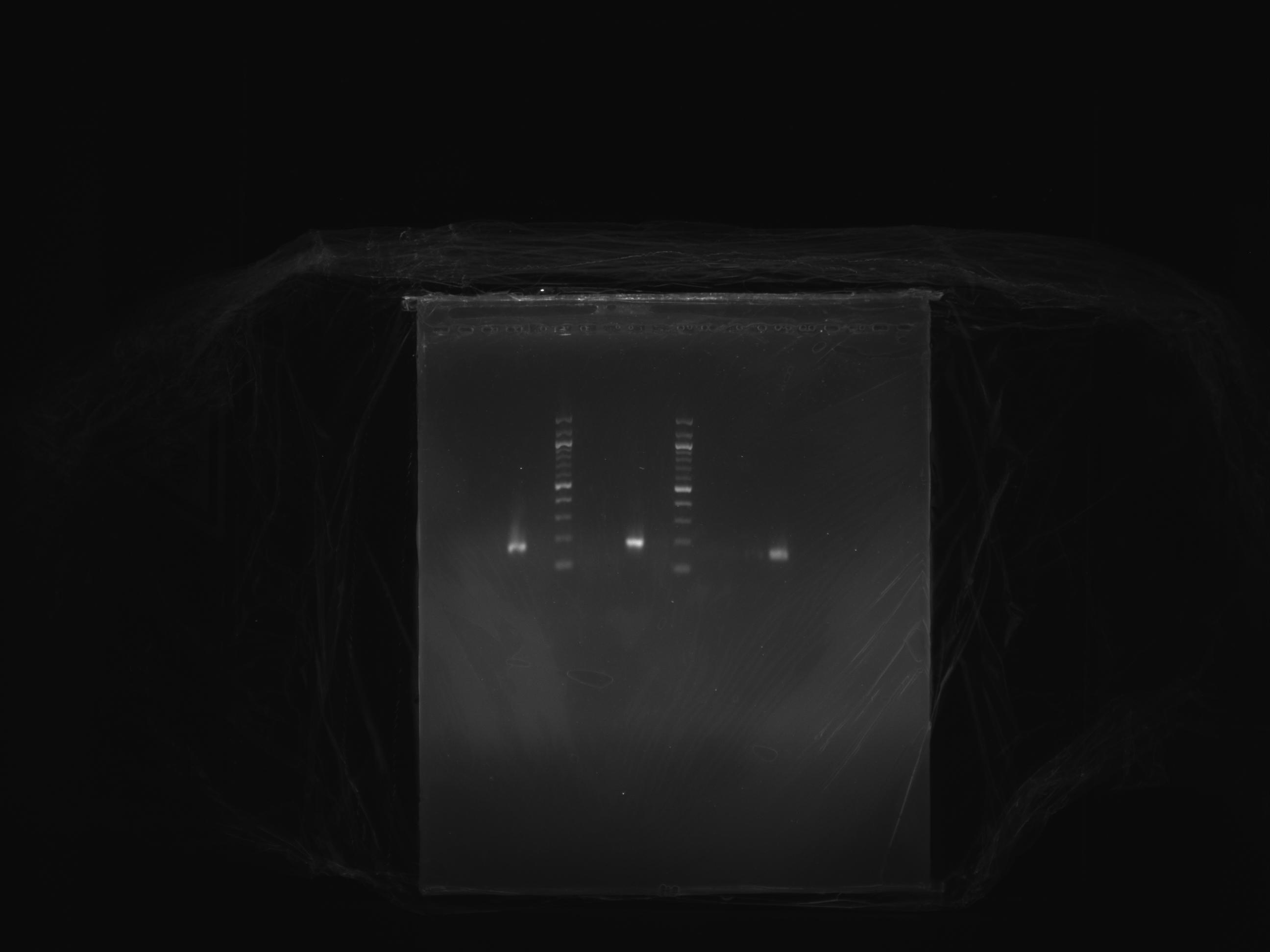

Supplement: Supplementary file 4 — Source Data [file 41467_2021_25989_MOESM4_ESM.zip › Image Files/Supp Fig 19/2020-01-13_10-23-48.jpg]

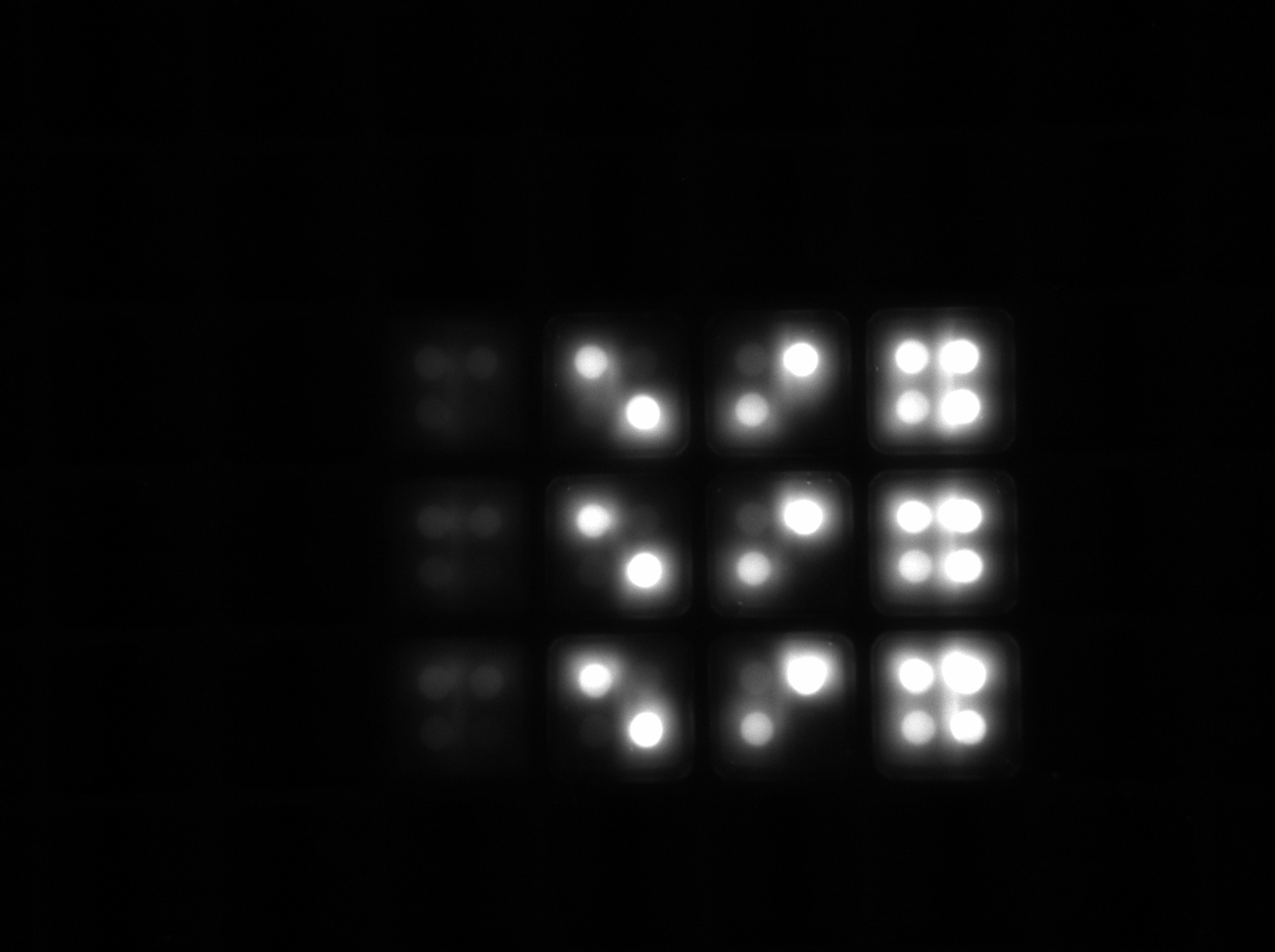

Supplement: Supplementary file 4 — Source Data [file 41467_2021_25989_MOESM4_ESM.zip › Image Files/Fig 5B & Supp Fig 11/Serum0_run2.tif]

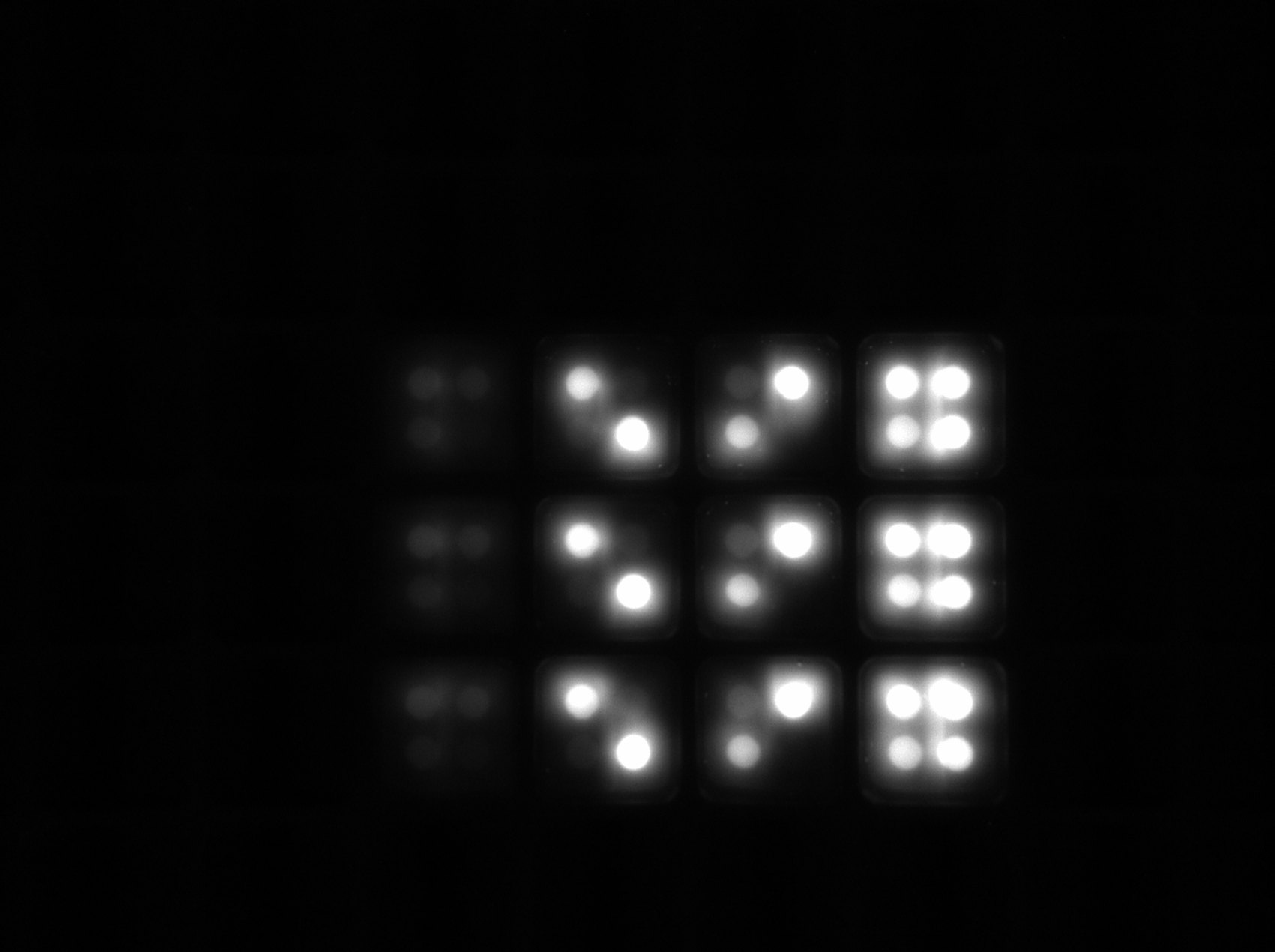

Supplement: Supplementary file 4 — Source Data [file 41467_2021_25989_MOESM4_ESM.zip › Image Files/Fig 5B & Supp Fig 11/Serum0_run3.tif]

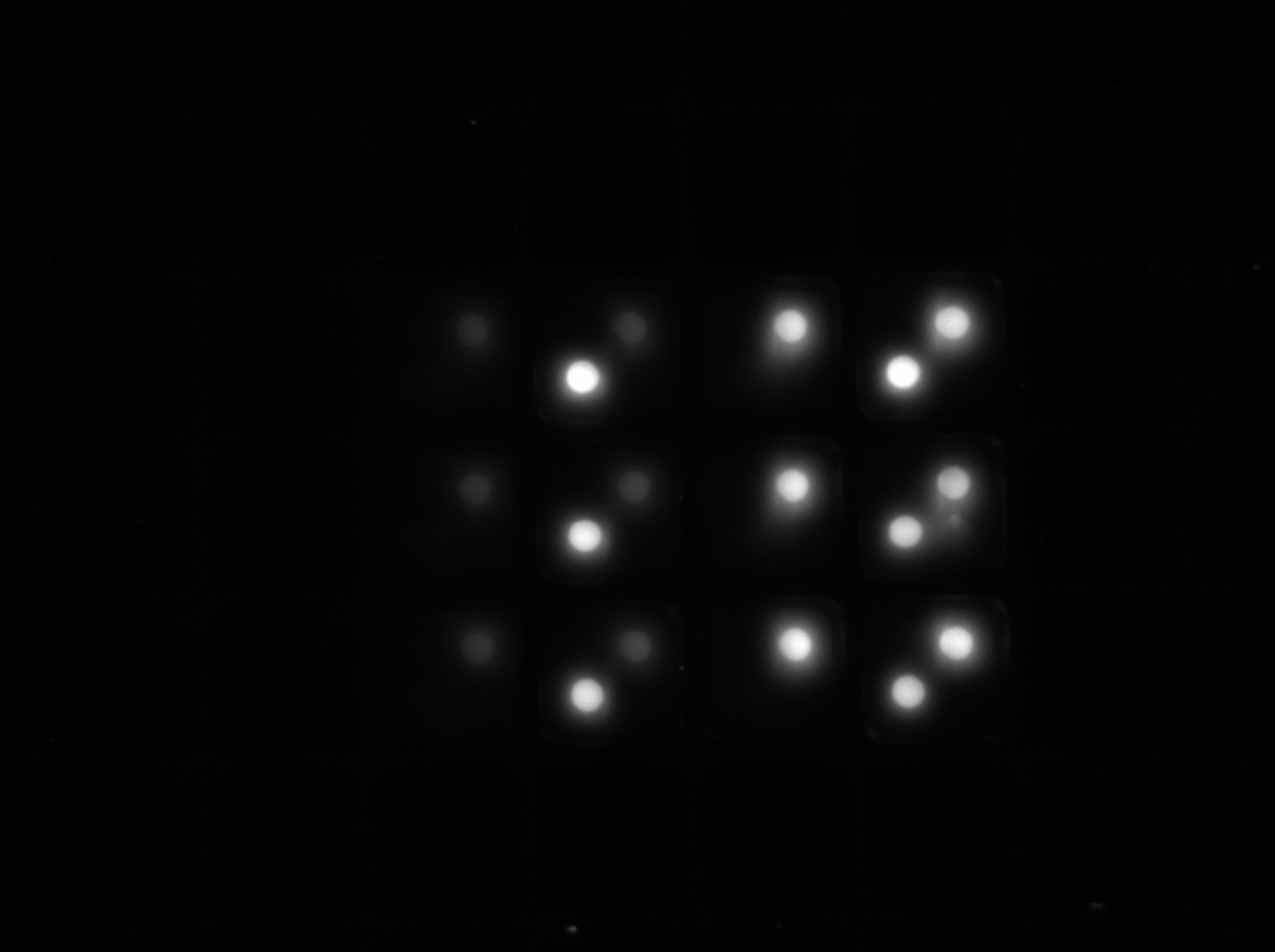

Supplement: Supplementary file 4 — Source Data [file 41467_2021_25989_MOESM4_ESM.zip › Image Files/Fig 3B & Supp Fig 3/AraIPTG_run1.tif]

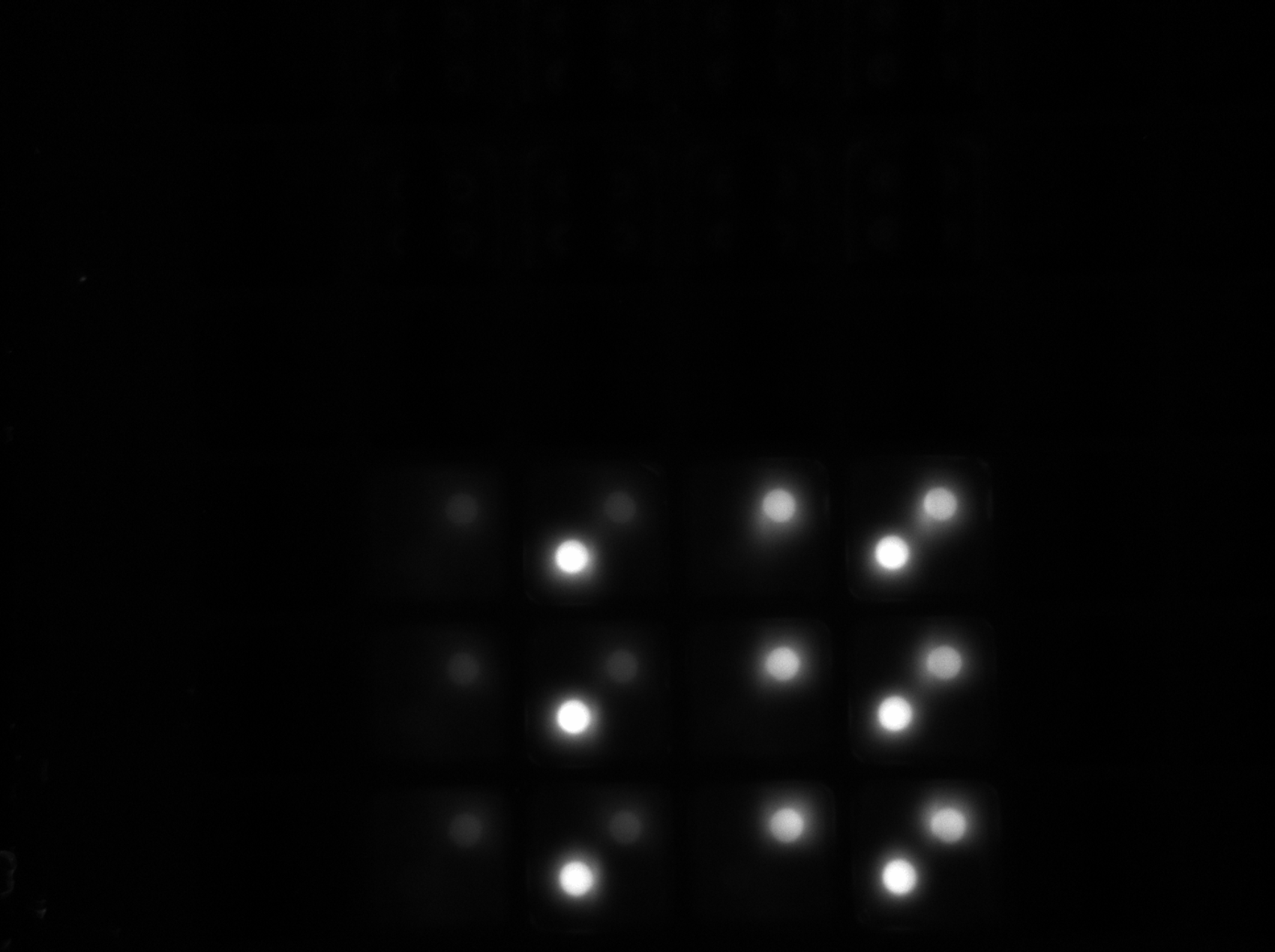

Supplement: Supplementary file 4 — Source Data [file 41467_2021_25989_MOESM4_ESM.zip › Image Files/Fig 3B & Supp Fig 3/AraIPTG_run3.tif]

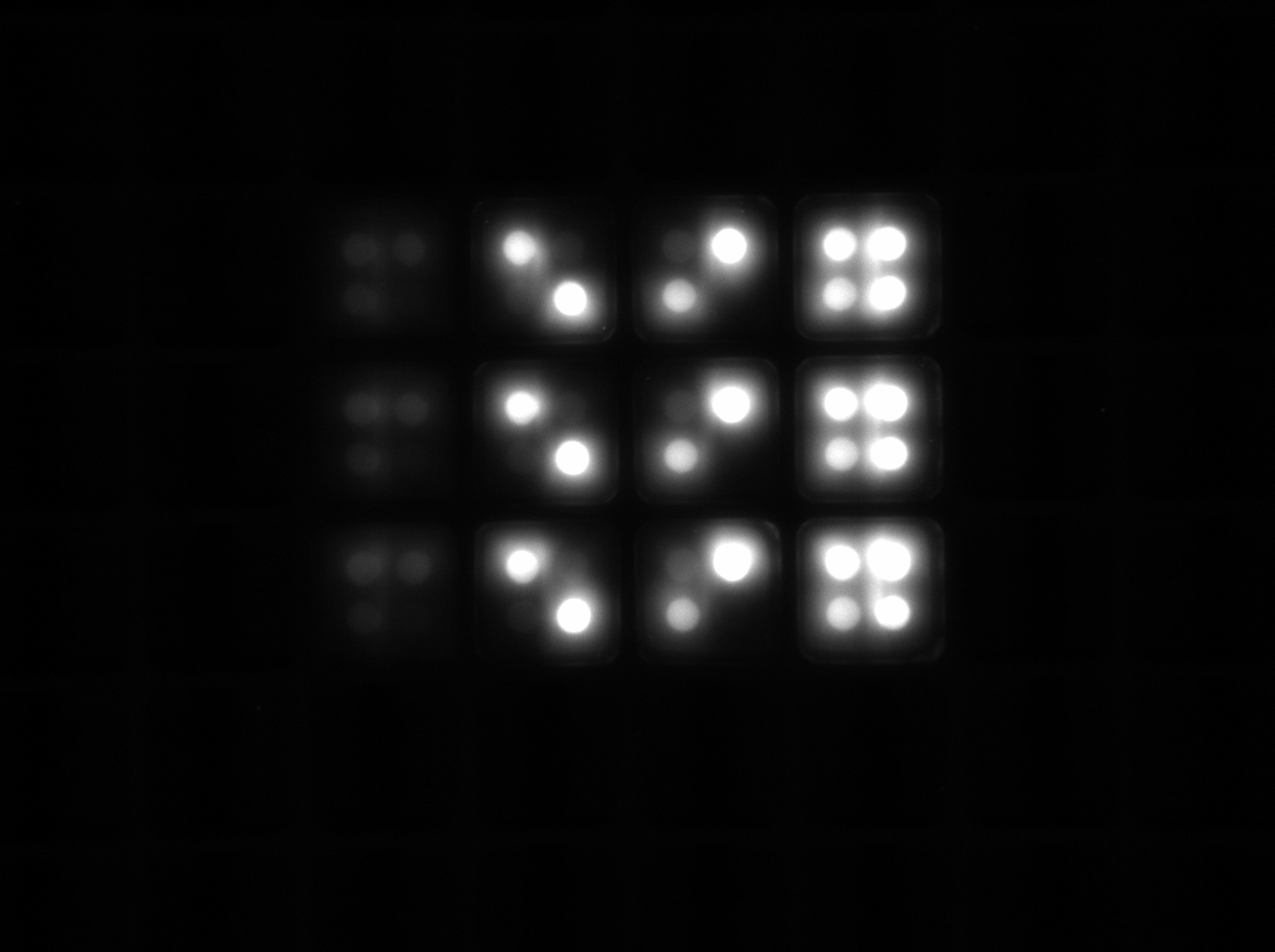

Supplement: Supplementary file 4 — Source Data [file 41467_2021_25989_MOESM4_ESM.zip › Image Files/Fig 5B & Supp Fig 11/Serum0_run1.tif]

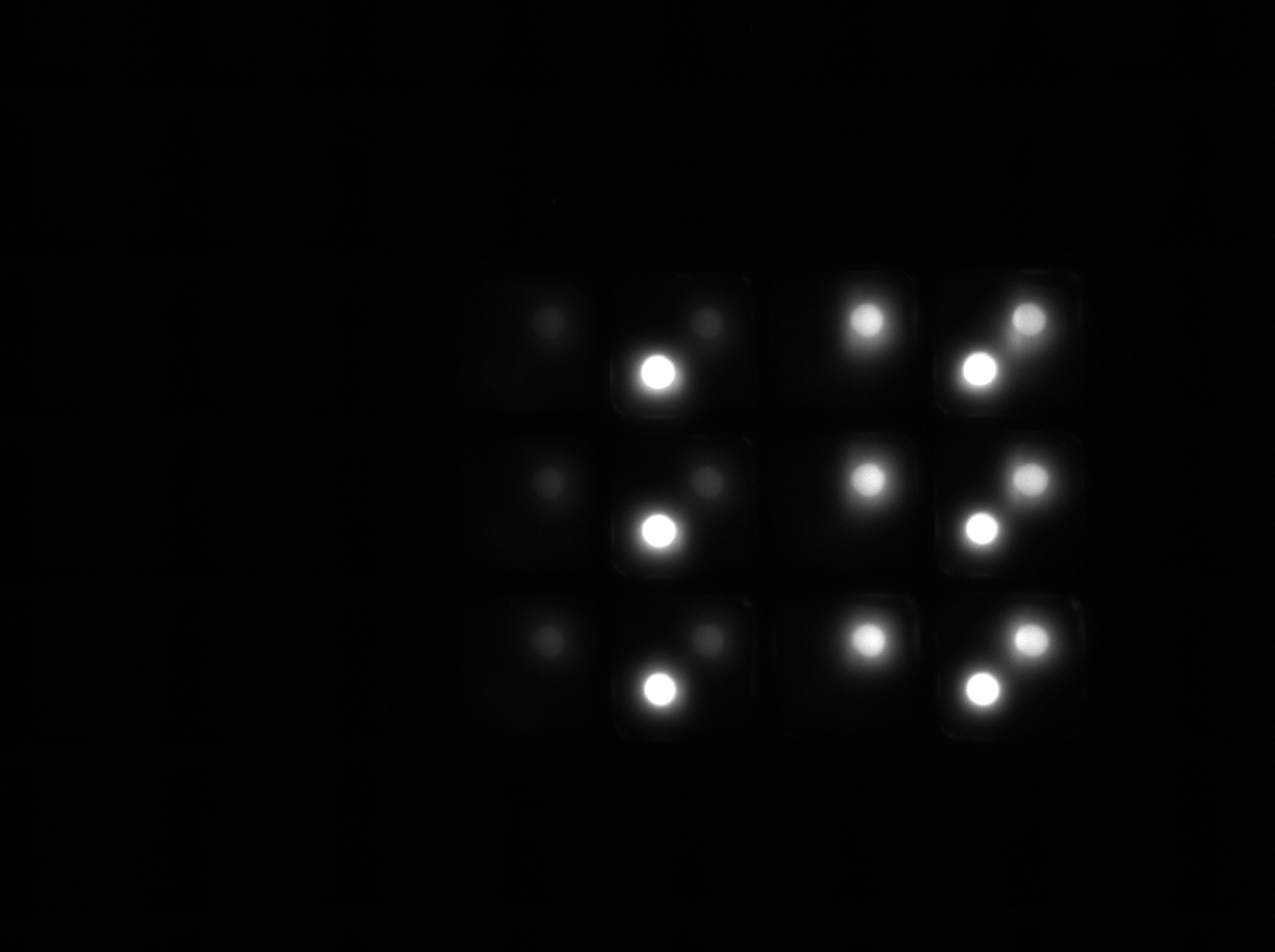

Supplement: Supplementary file 4 — Source Data [file 41467_2021_25989_MOESM4_ESM.zip › Image Files/Fig 3B & Supp Fig 3/AraIPTG_run2.tif]

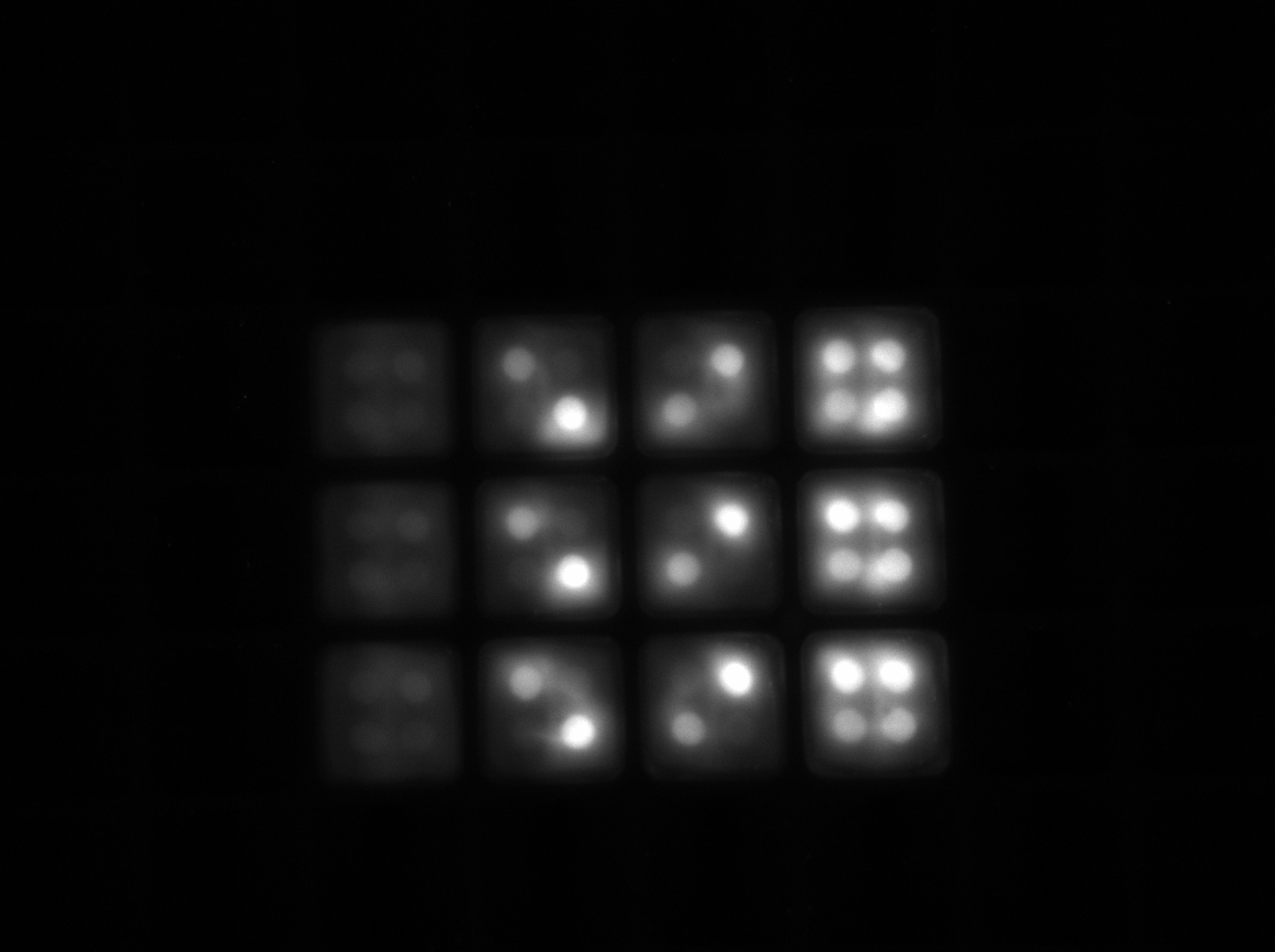

Supplement: Supplementary file 4 — Source Data [file 41467_2021_25989_MOESM4_ESM.zip › Image Files/Fig 5C & Supp Fig 13/Serum20_run3.tif]

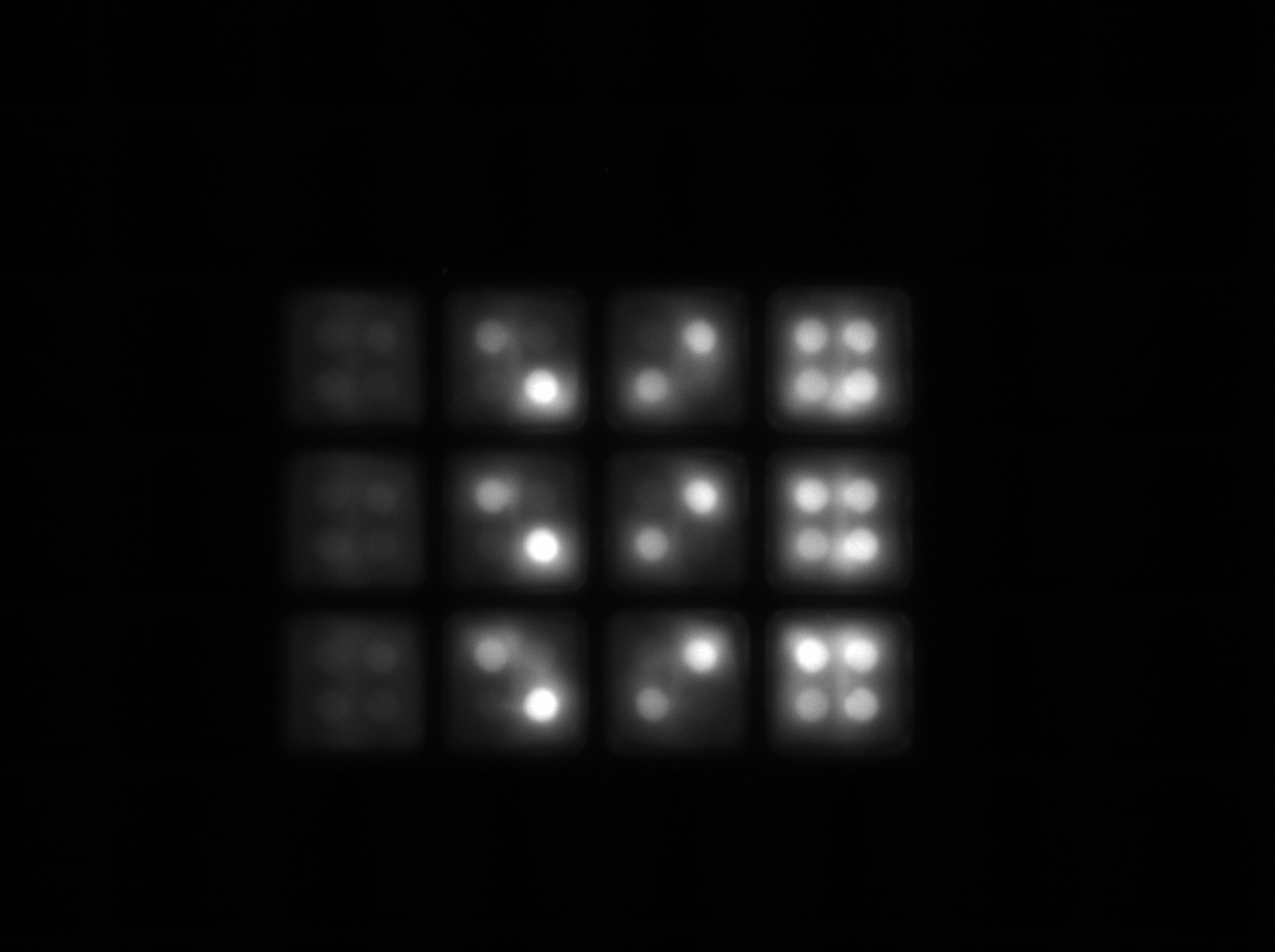

Supplement: Supplementary file 4 — Source Data [file 41467_2021_25989_MOESM4_ESM.zip › Image Files/Fig 5C & Supp Fig 13/Serum20_run2.tif]

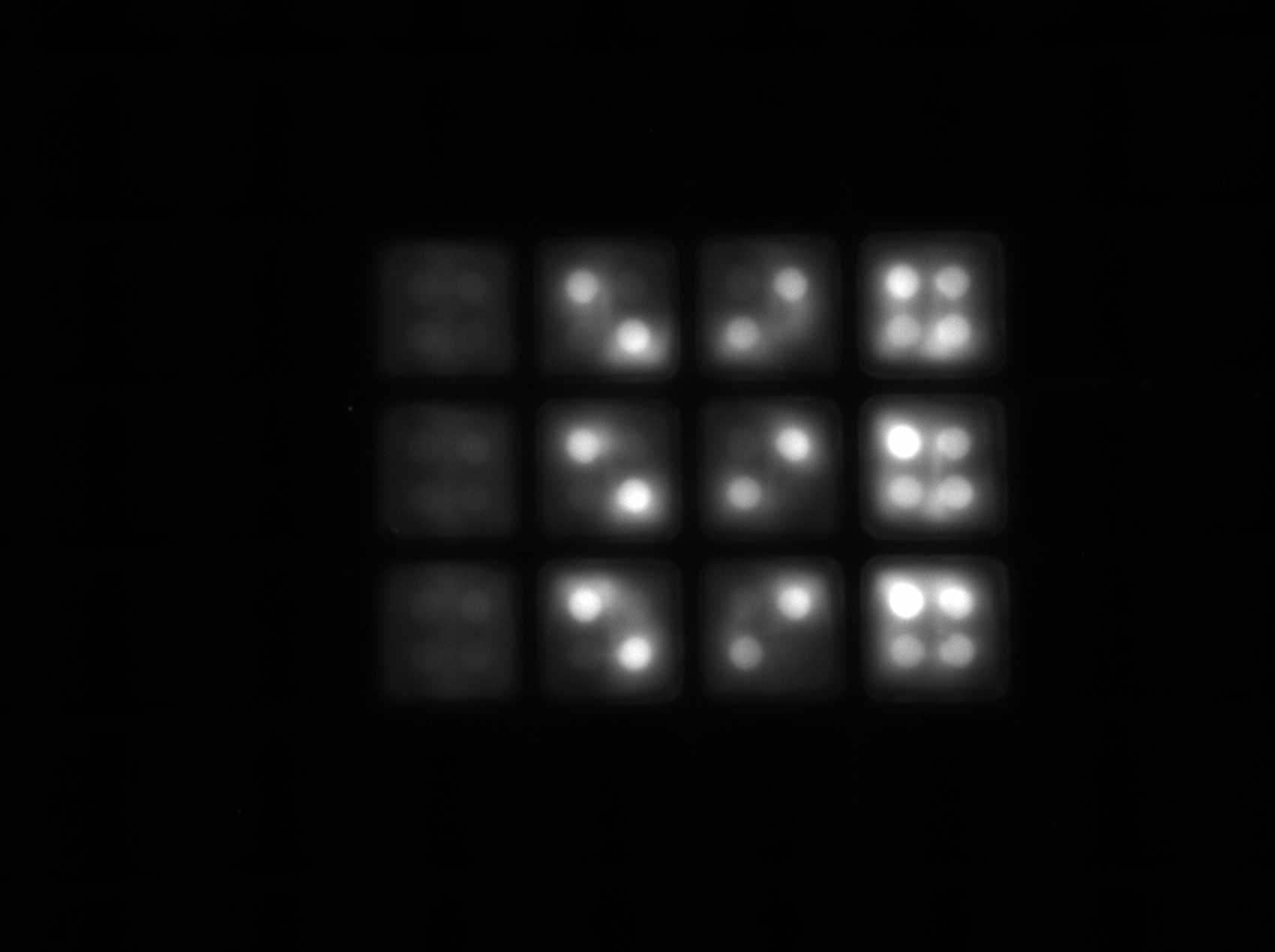

Supplement: Supplementary file 4 — Source Data [file 41467_2021_25989_MOESM4_ESM.zip › Image Files/Fig 5C & Supp Fig 13/Serum20_run1.tif]

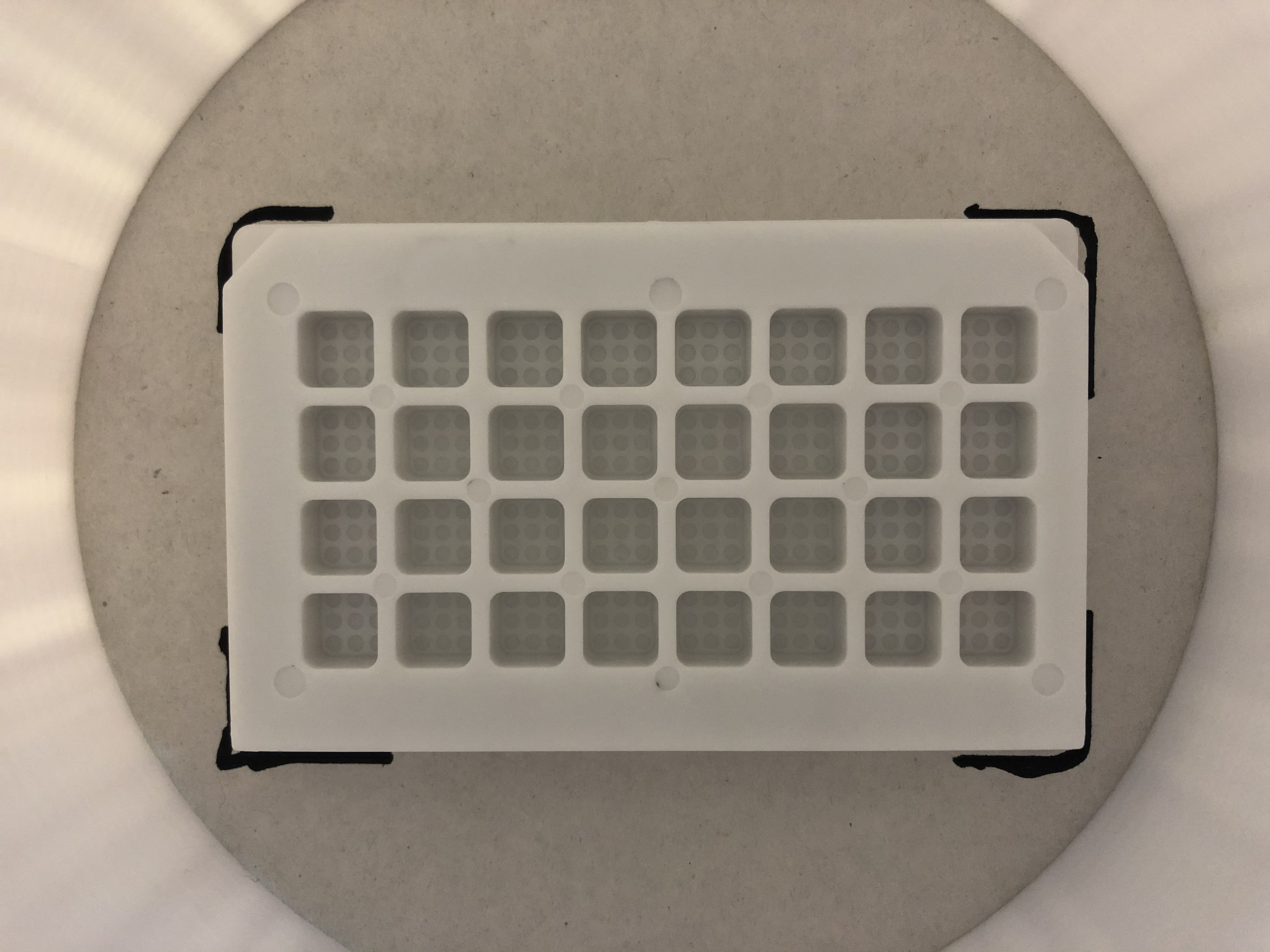

Supplement: Supplementary file 4 — Source Data [file 41467_2021_25989_MOESM4_ESM.zip › Image Files/Fig 6B & Supp Fig 16/Fresh_Run3/Run3_0hr.jpeg]

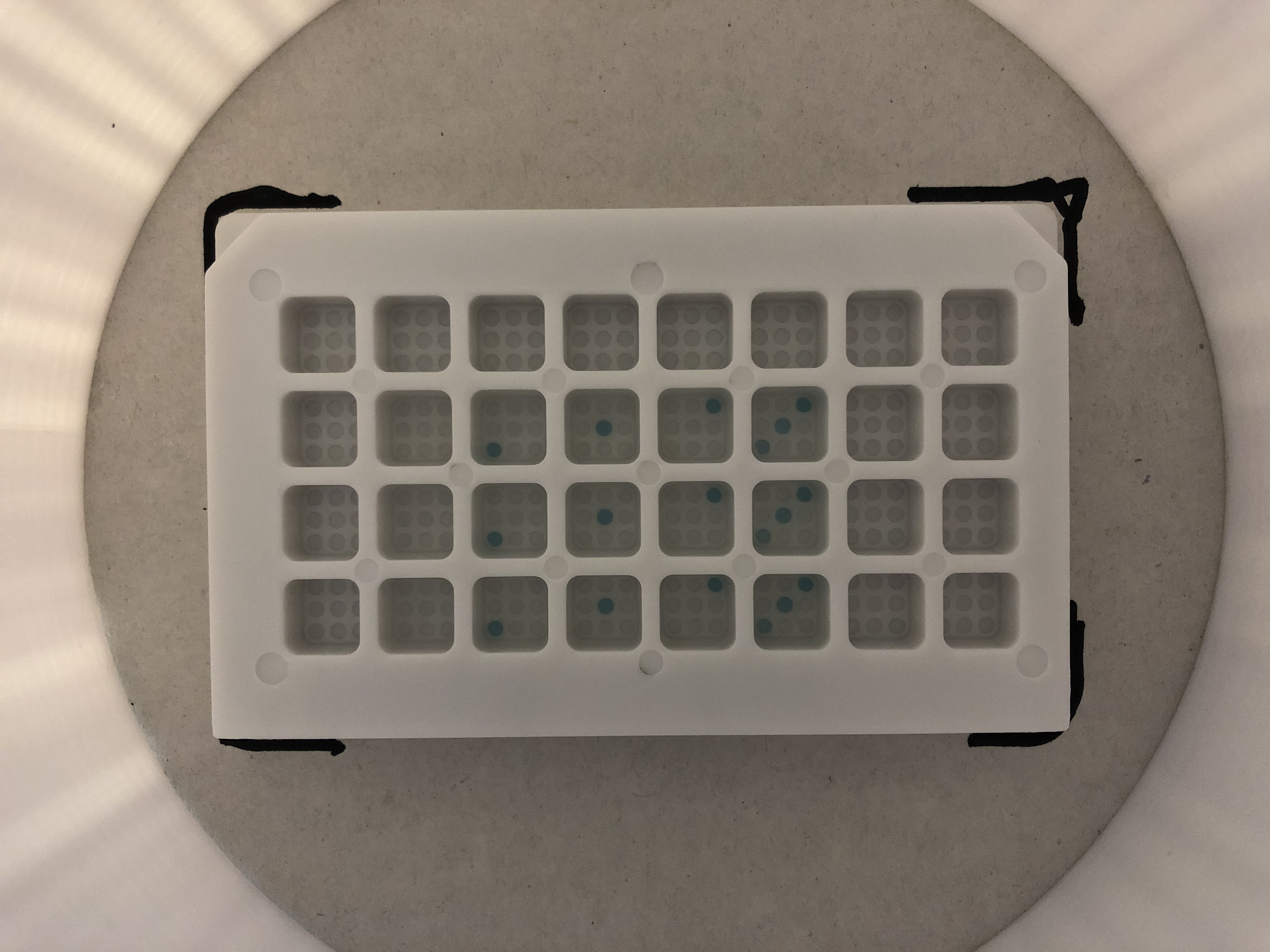

Supplement: Supplementary file 4 — Source Data [file 41467_2021_25989_MOESM4_ESM.zip › Image Files/Fig 6B & Supp Fig 16/Fresh_Run2/Run2_2hr.jpeg]

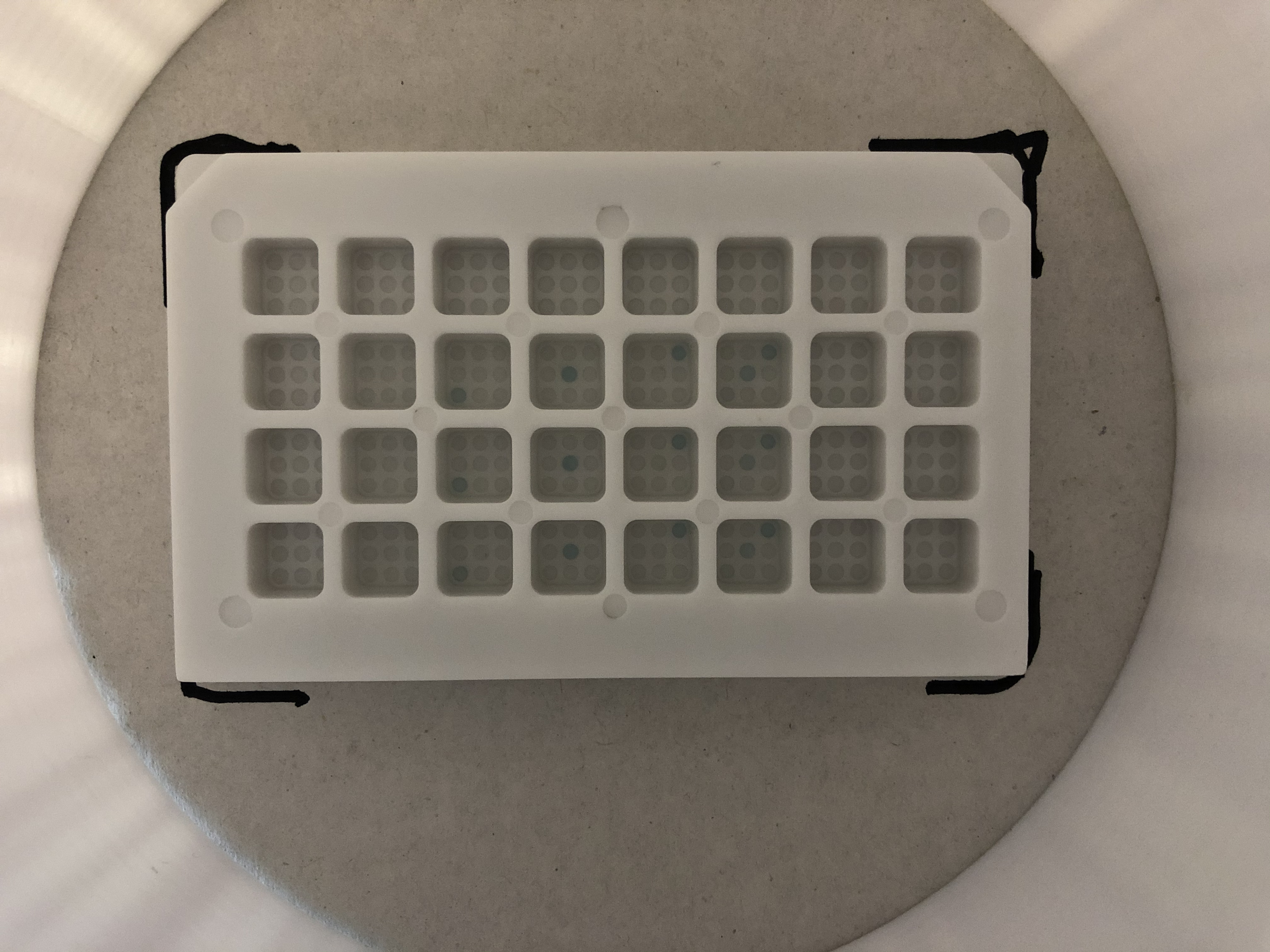

Supplement: Supplementary file 4 — Source Data [file 41467_2021_25989_MOESM4_ESM.zip › Image Files/Fig 6B & Supp Fig 16/Fresh_Run1/Run1_1hr.jpeg]

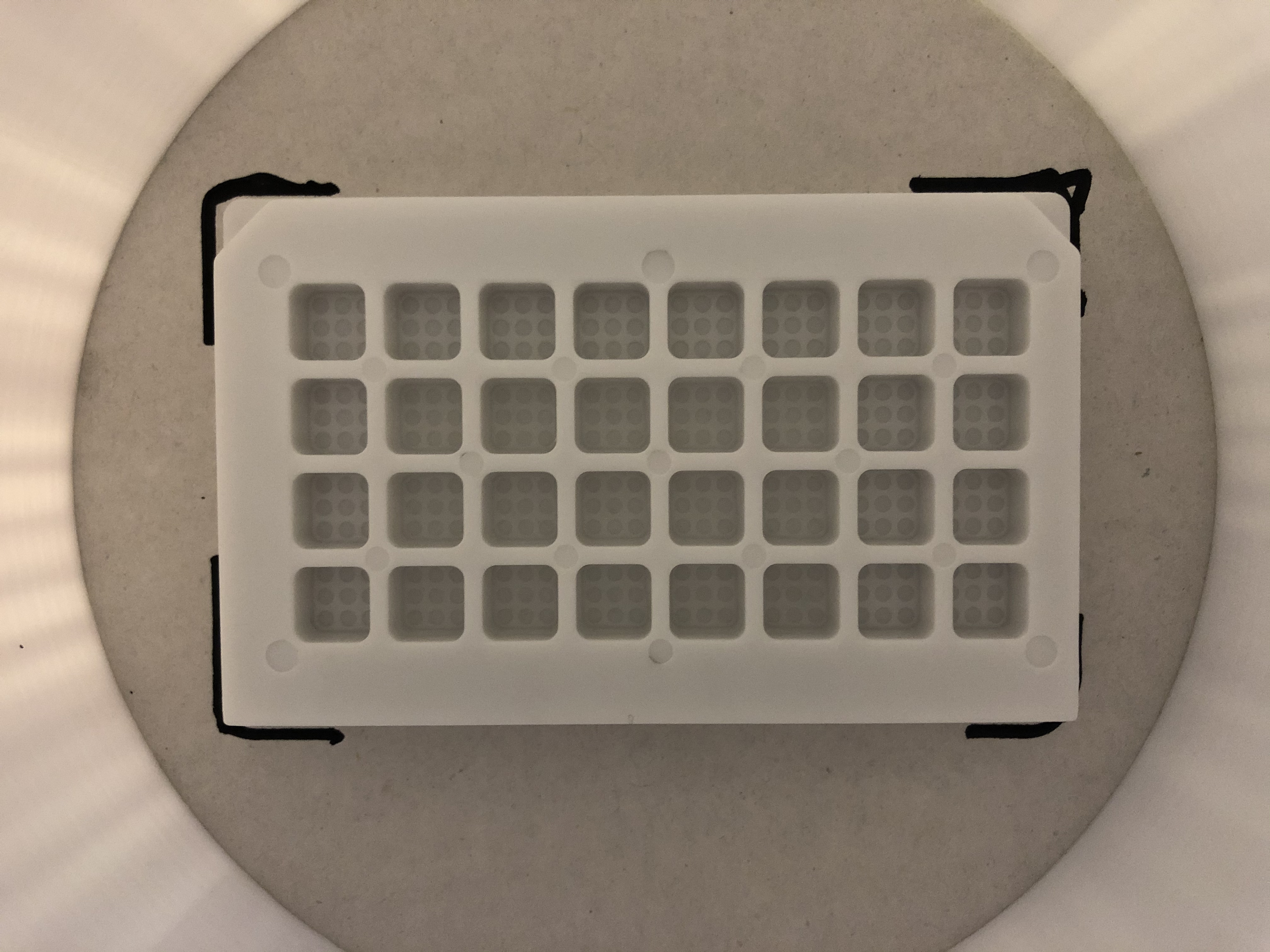

Supplement: Supplementary file 4 — Source Data [file 41467_2021_25989_MOESM4_ESM.zip › Image Files/Fig 6B & Supp Fig 16/Fresh_Run2/Run2_0hr.jpeg]

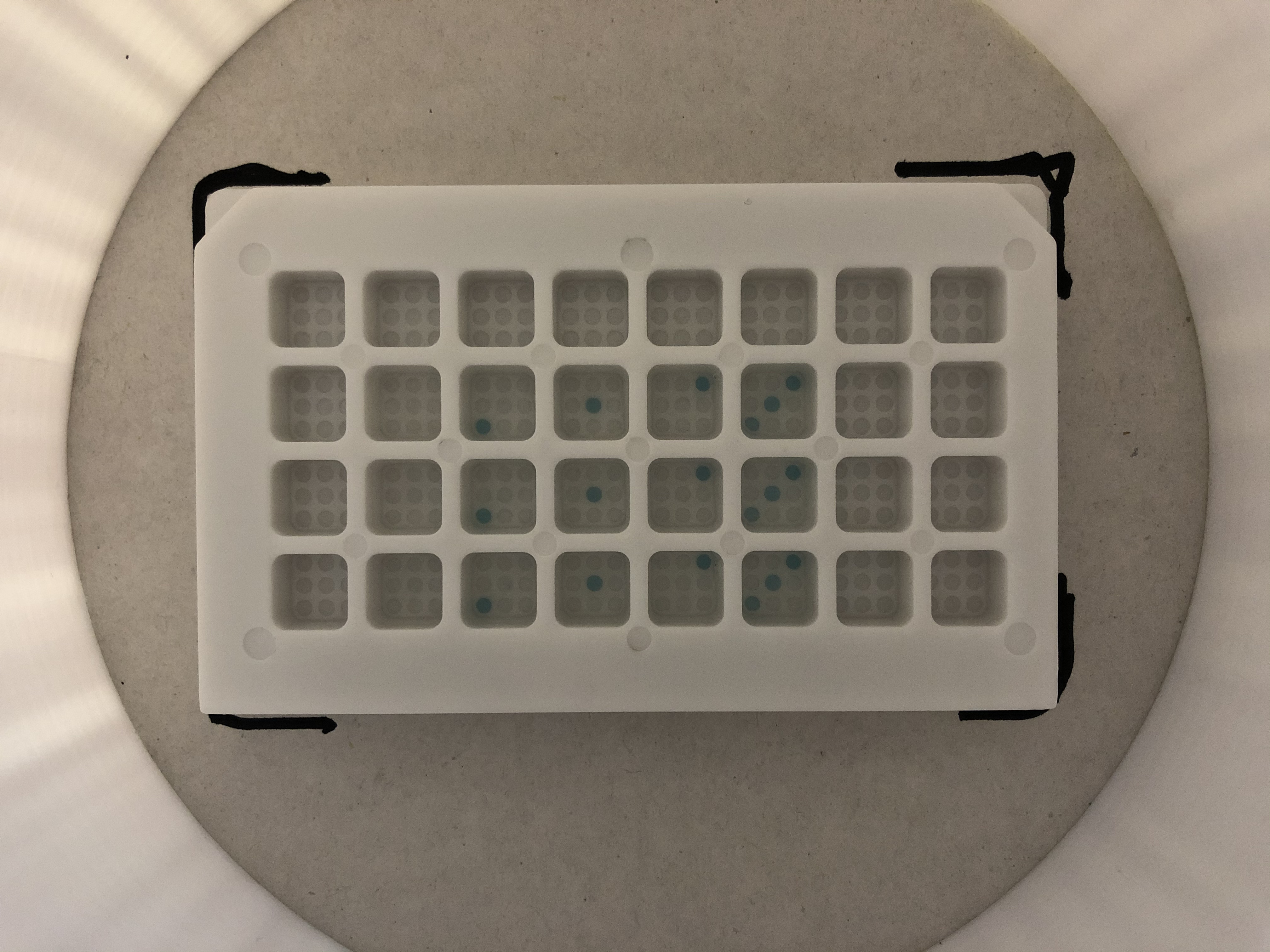

Supplement: Supplementary file 4 — Source Data [file 41467_2021_25989_MOESM4_ESM.zip › Image Files/Fig 6B & Supp Fig 16/Fresh_Run1/Run1_2hr.jpeg]

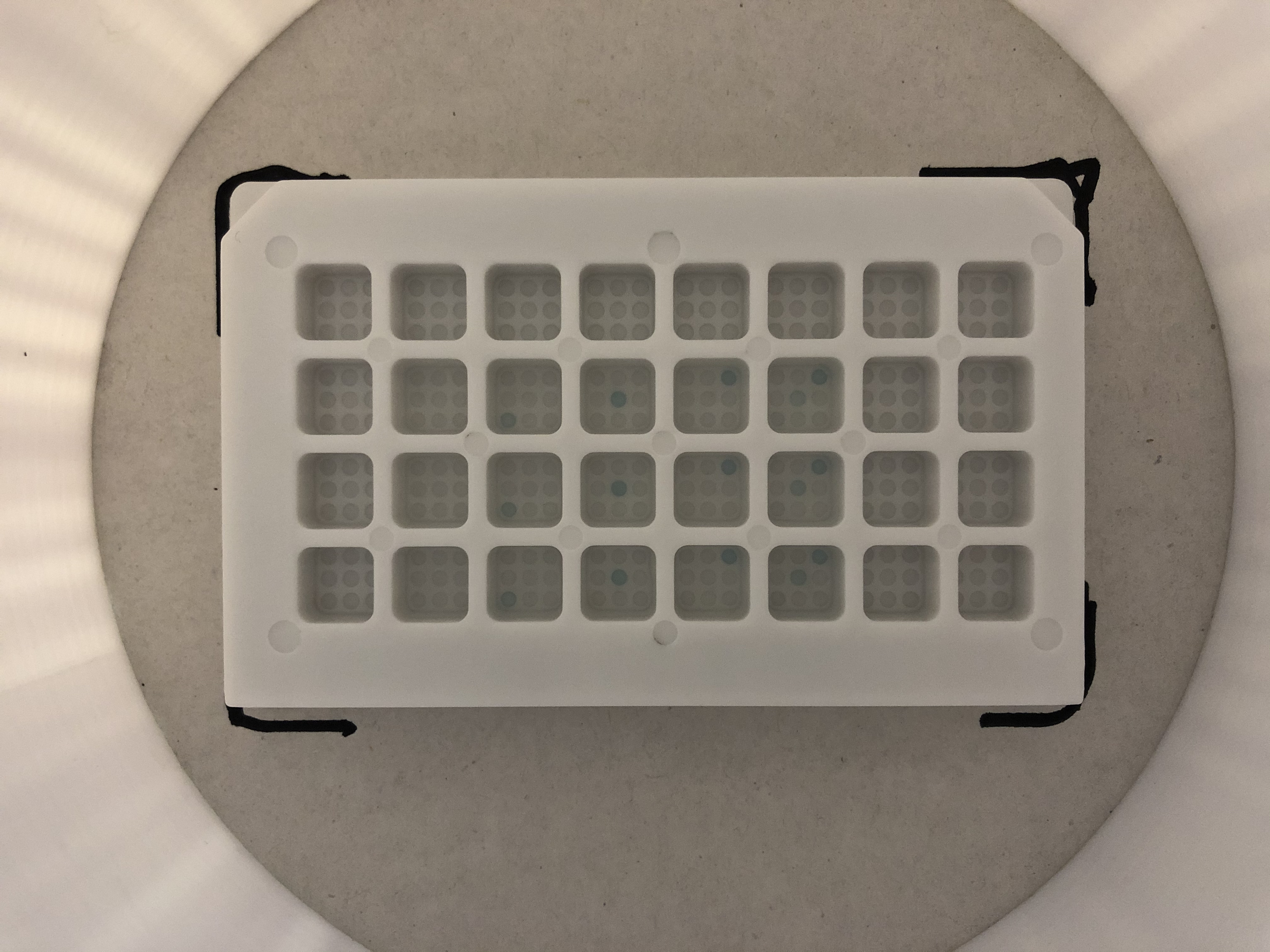

Supplement: Supplementary file 4 — Source Data [file 41467_2021_25989_MOESM4_ESM.zip › Image Files/Fig 6B & Supp Fig 16/Fresh_Run2/Run2_1hr.jpeg]

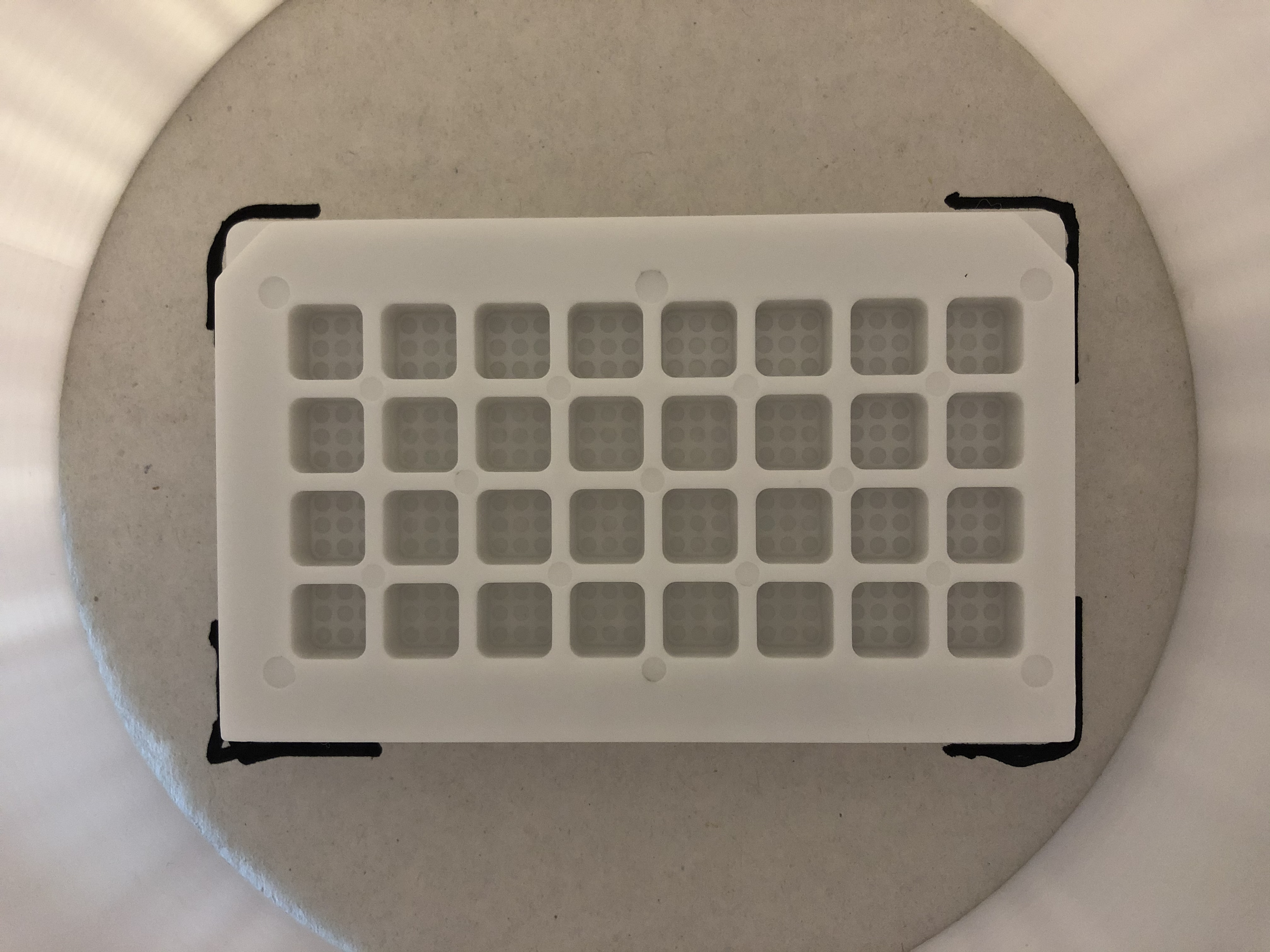

Supplement: Supplementary file 4 — Source Data [file 41467_2021_25989_MOESM4_ESM.zip › Image Files/Fig 6B & Supp Fig 16/Fresh_Run1/Run1_0hr.jpeg]

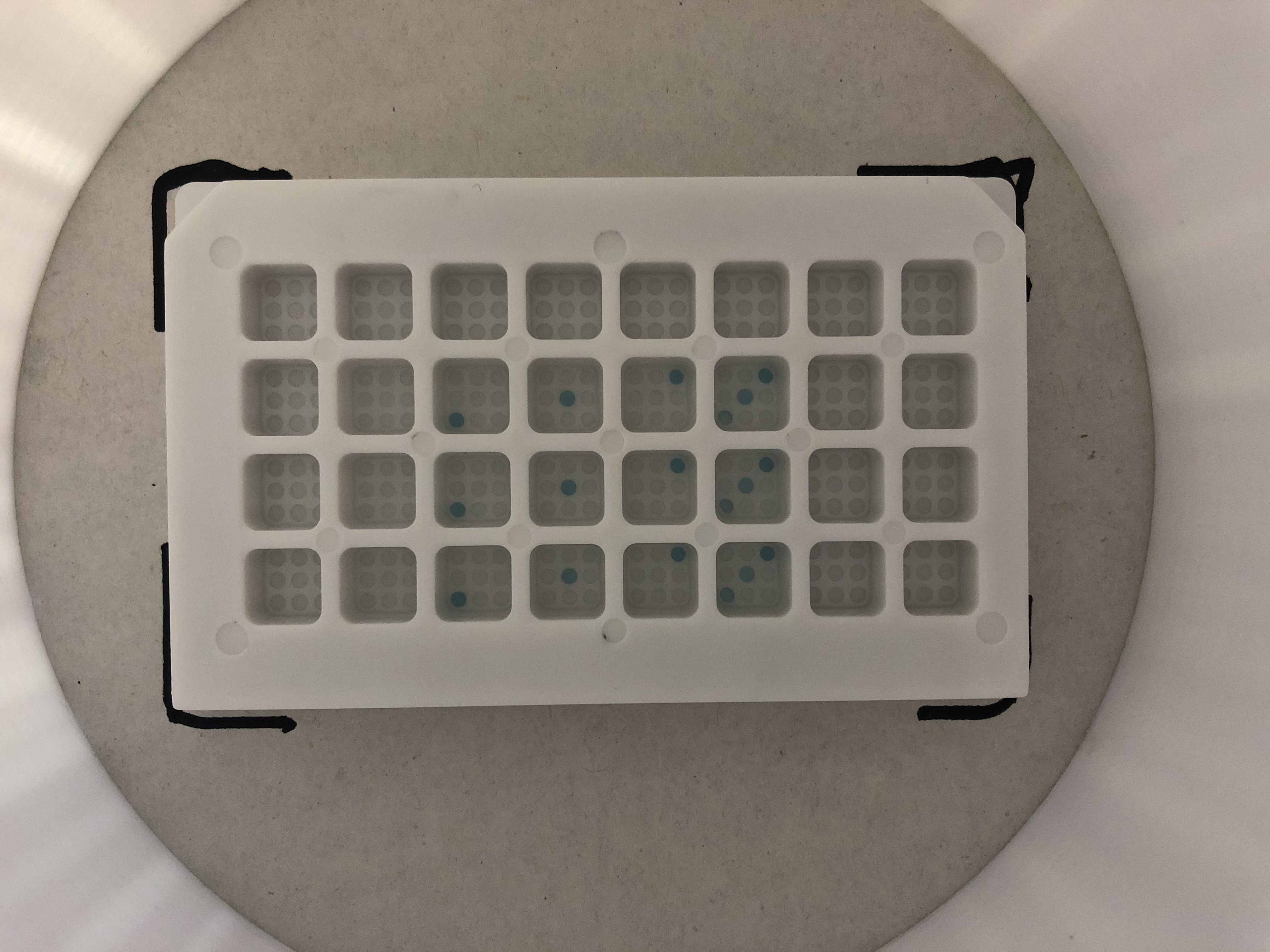

Supplement: Supplementary file 4 — Source Data [file 41467_2021_25989_MOESM4_ESM.zip › Image Files/Fig 6B & Supp Fig 16/Fresh_Run3/Run3_2hr.jpeg]

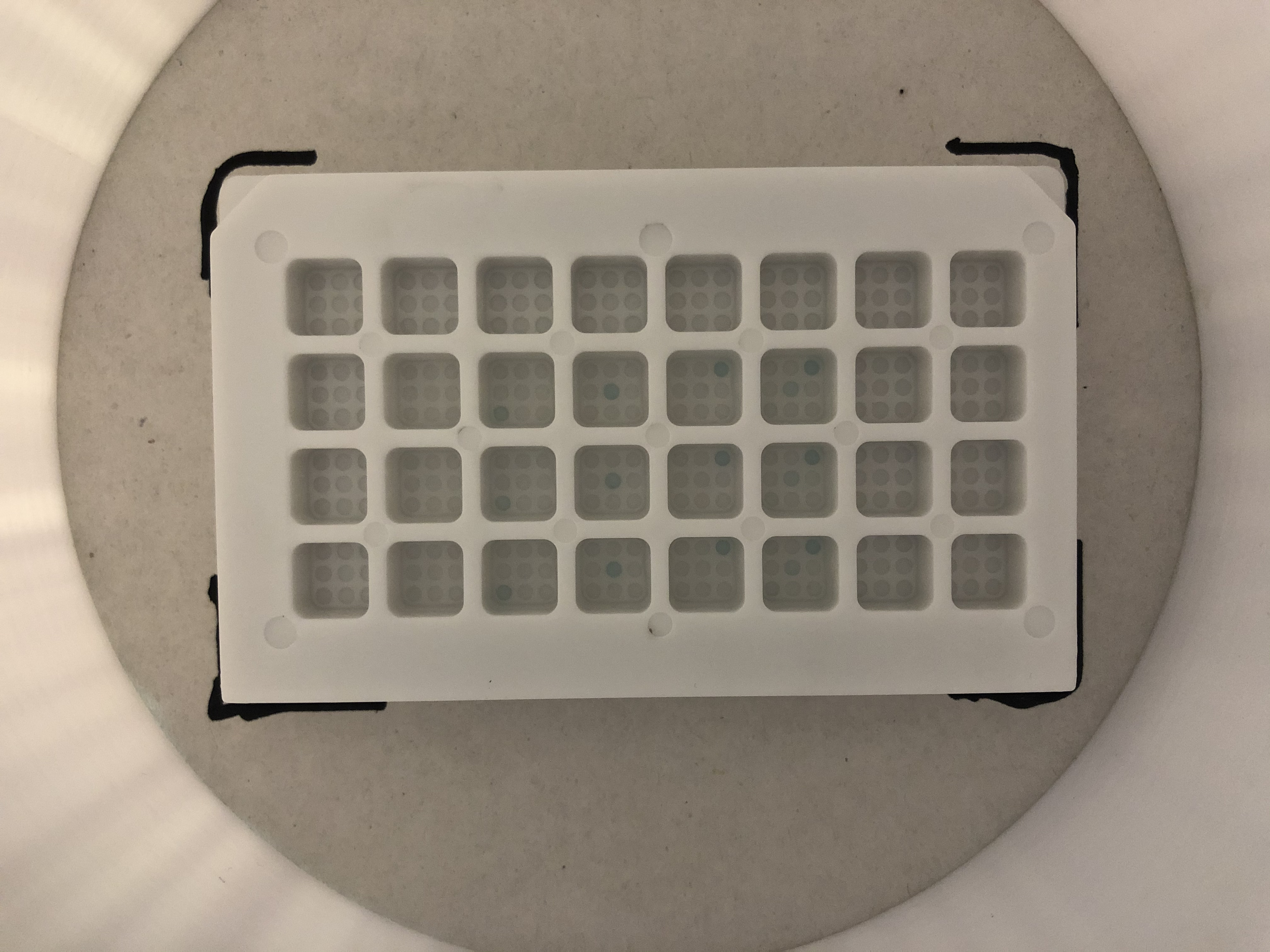

Supplement: Supplementary file 4 — Source Data [file 41467_2021_25989_MOESM4_ESM.zip › Image Files/Fig 6B & Supp Fig 16/Fresh_Run3/Run3_1hr.jpeg]

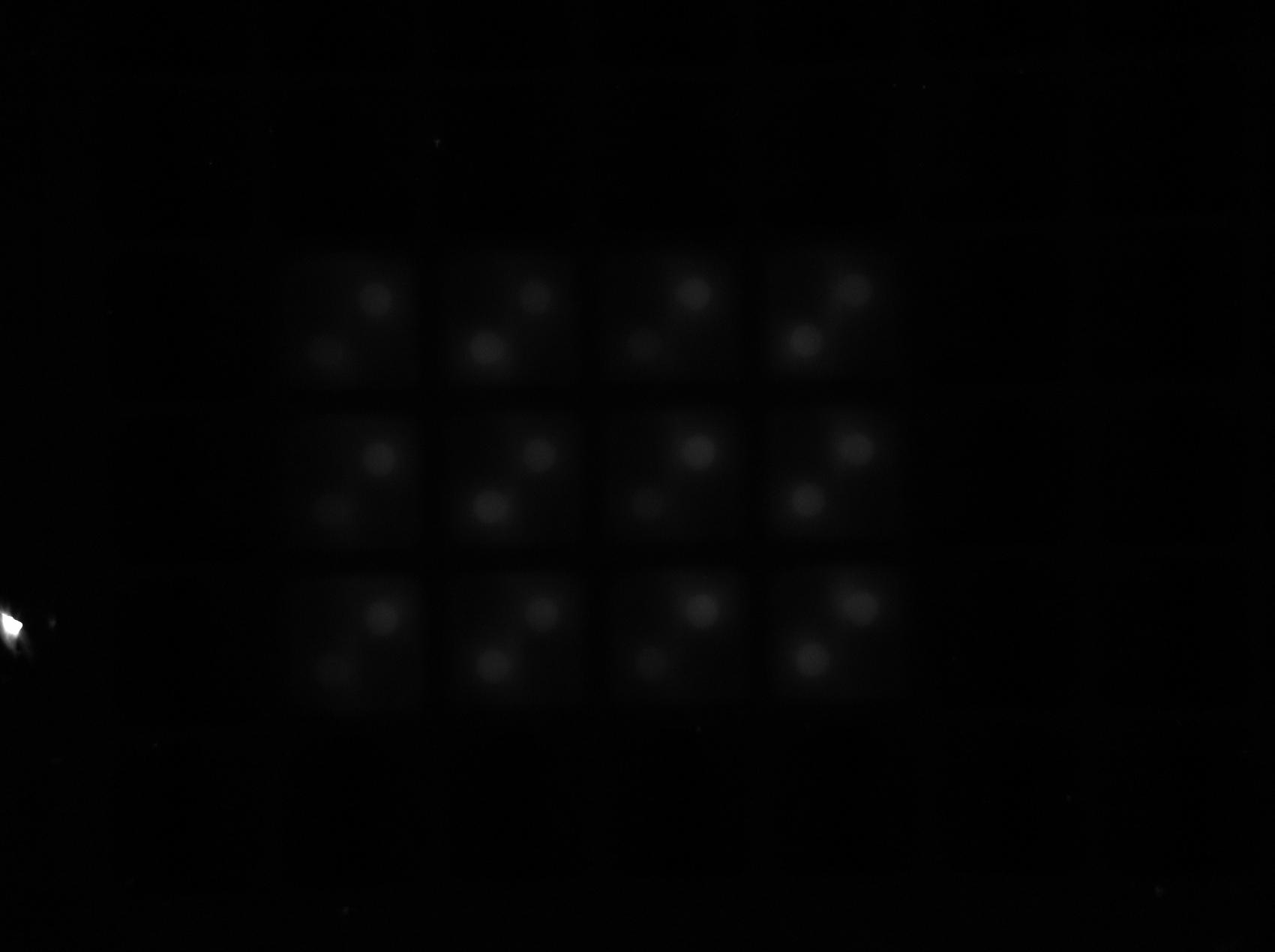

Supplement: Supplementary file 4 — Source Data [file 41467_2021_25989_MOESM4_ESM.zip › Image Files/Fig 4C & Supp Fig 4/RNA 10nM/RNA_10nM_day3.tif]

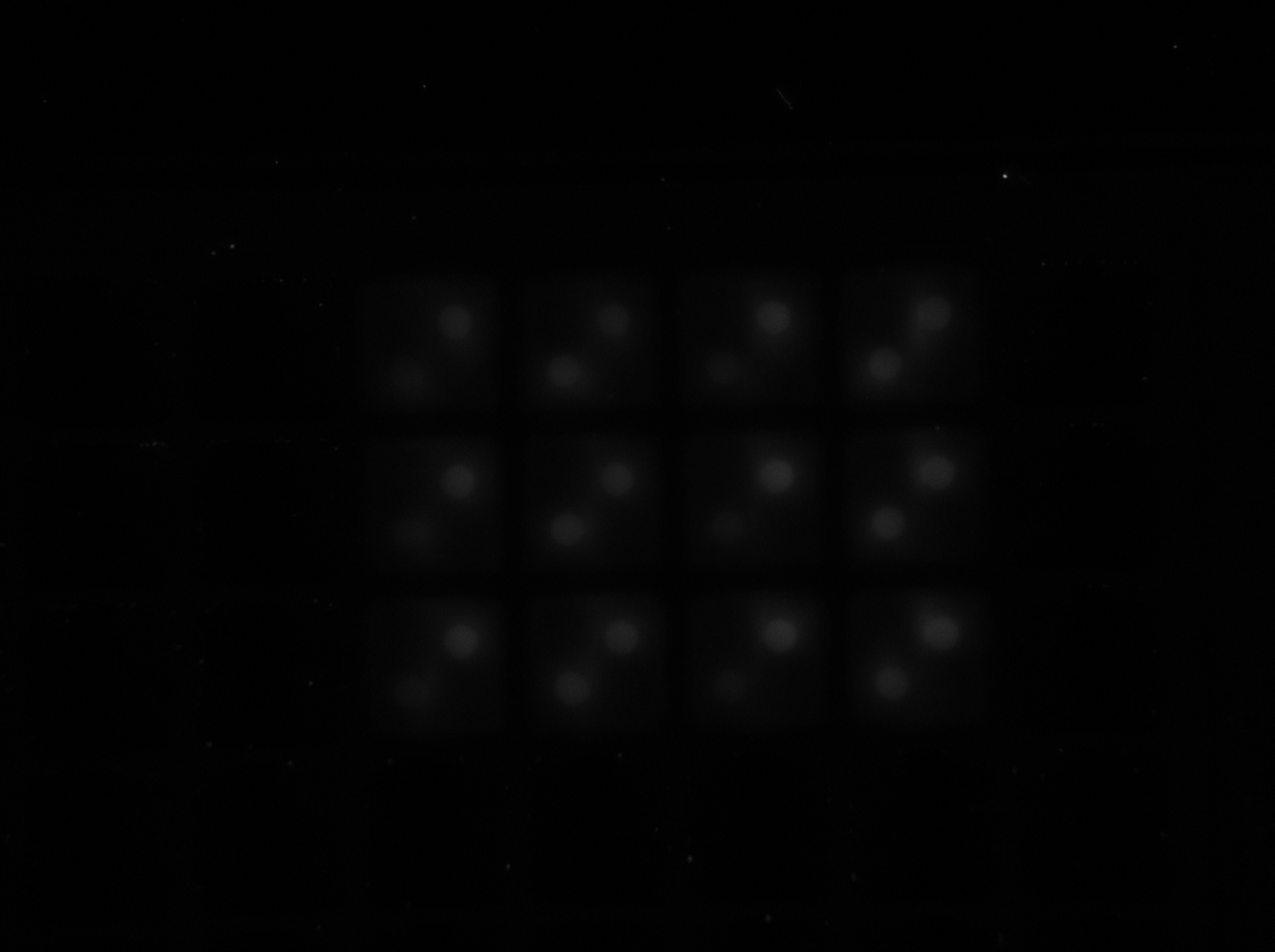

Supplement: Supplementary file 4 — Source Data [file 41467_2021_25989_MOESM4_ESM.zip › Image Files/Fig 4C & Supp Fig 4/RNA 10nM/RNA_10nM_day2.tif]

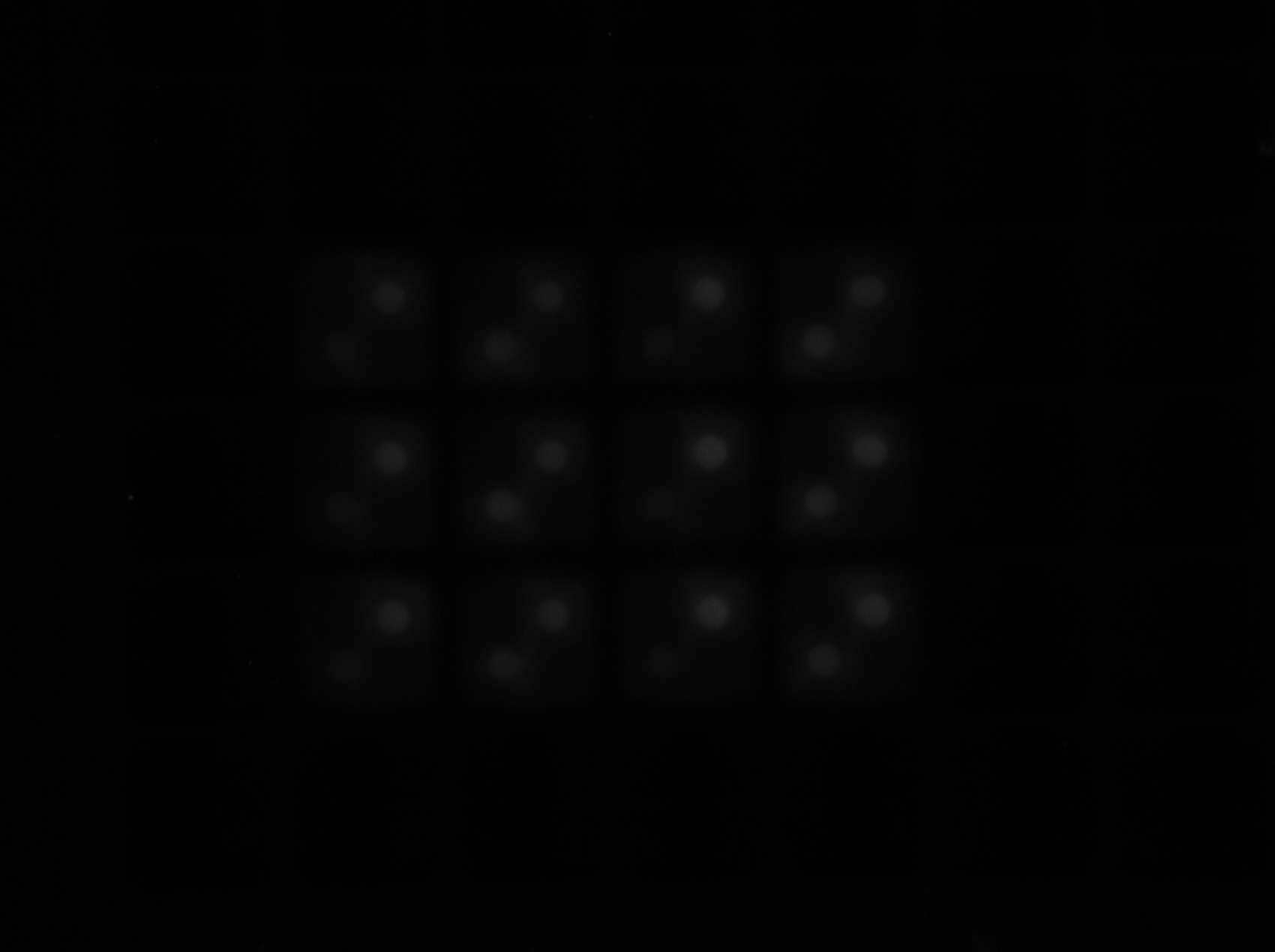

Supplement: Supplementary file 4 — Source Data [file 41467_2021_25989_MOESM4_ESM.zip › Image Files/Fig 4C & Supp Fig 4/RNA 10nM/RNA_10nM_day1.tif]

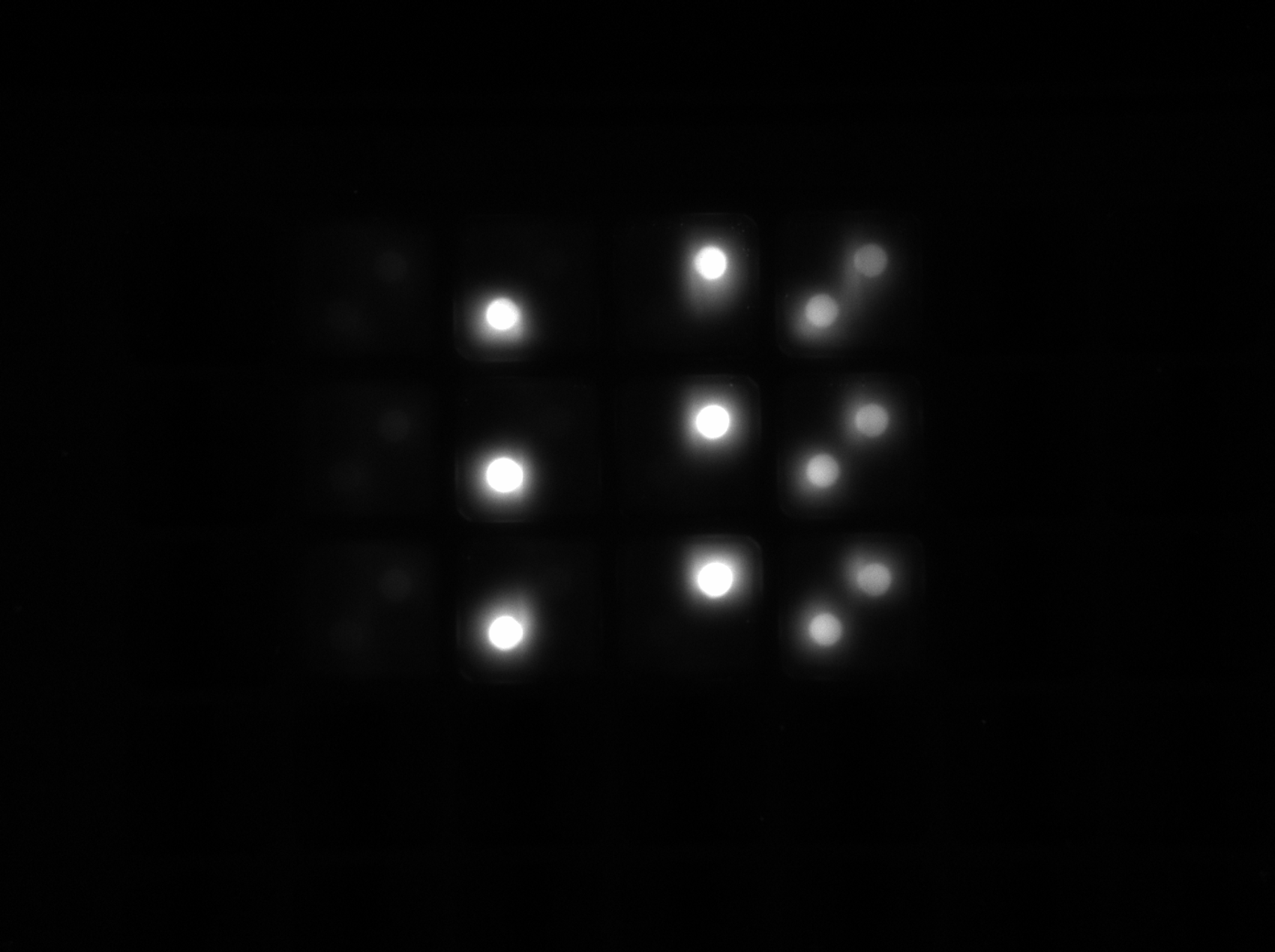

Supplement: Supplementary file 4 — Source Data [file 41467_2021_25989_MOESM4_ESM.zip › Image Files/Fig 4C & Supp Fig 4/RNA 1000nM/RNA_1uM_day3.tif]

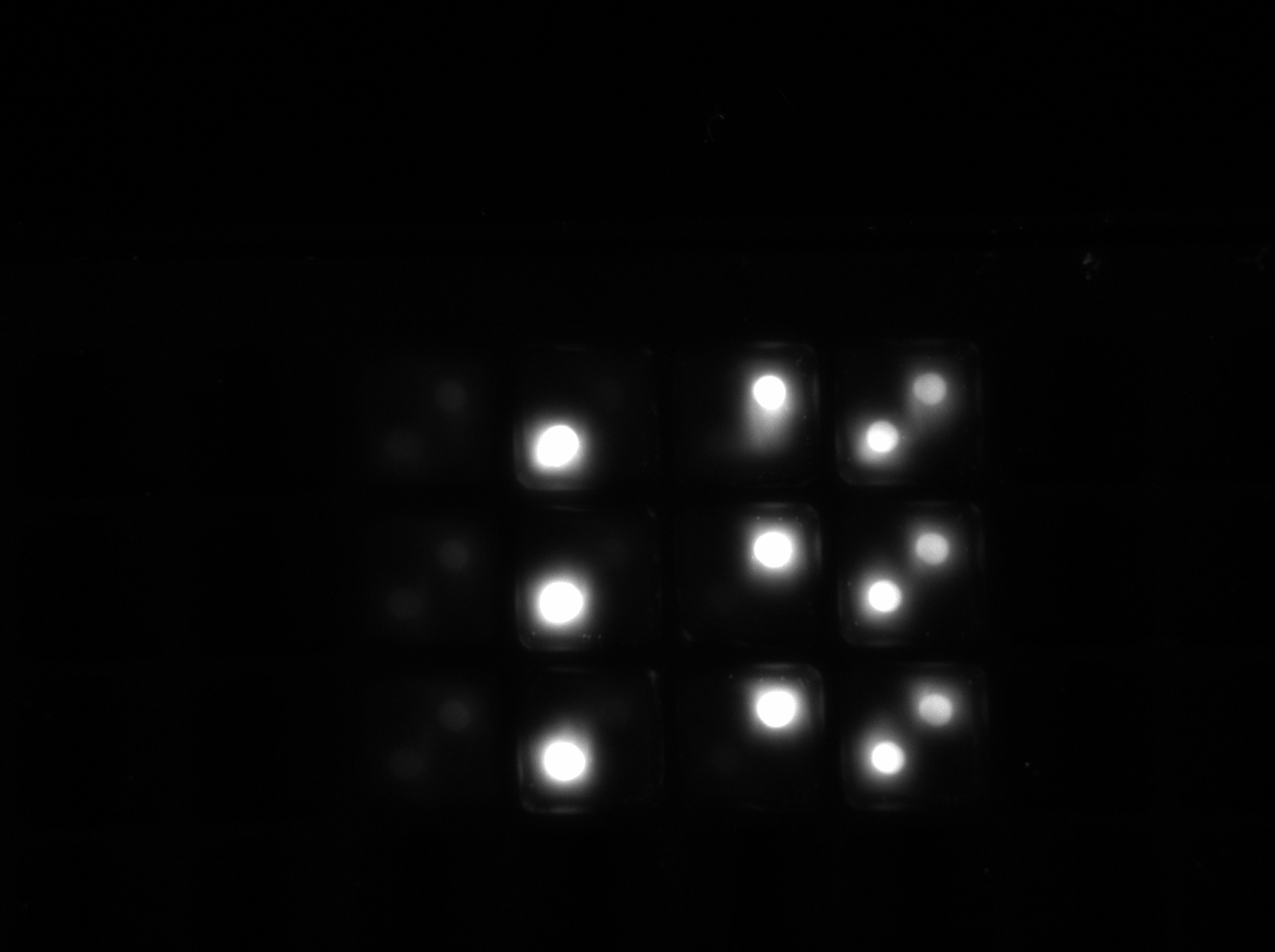

Supplement: Supplementary file 4 — Source Data [file 41467_2021_25989_MOESM4_ESM.zip › Image Files/Fig 4C & Supp Fig 4/RNA 1000nM/RNA_1uM_day1.tif]

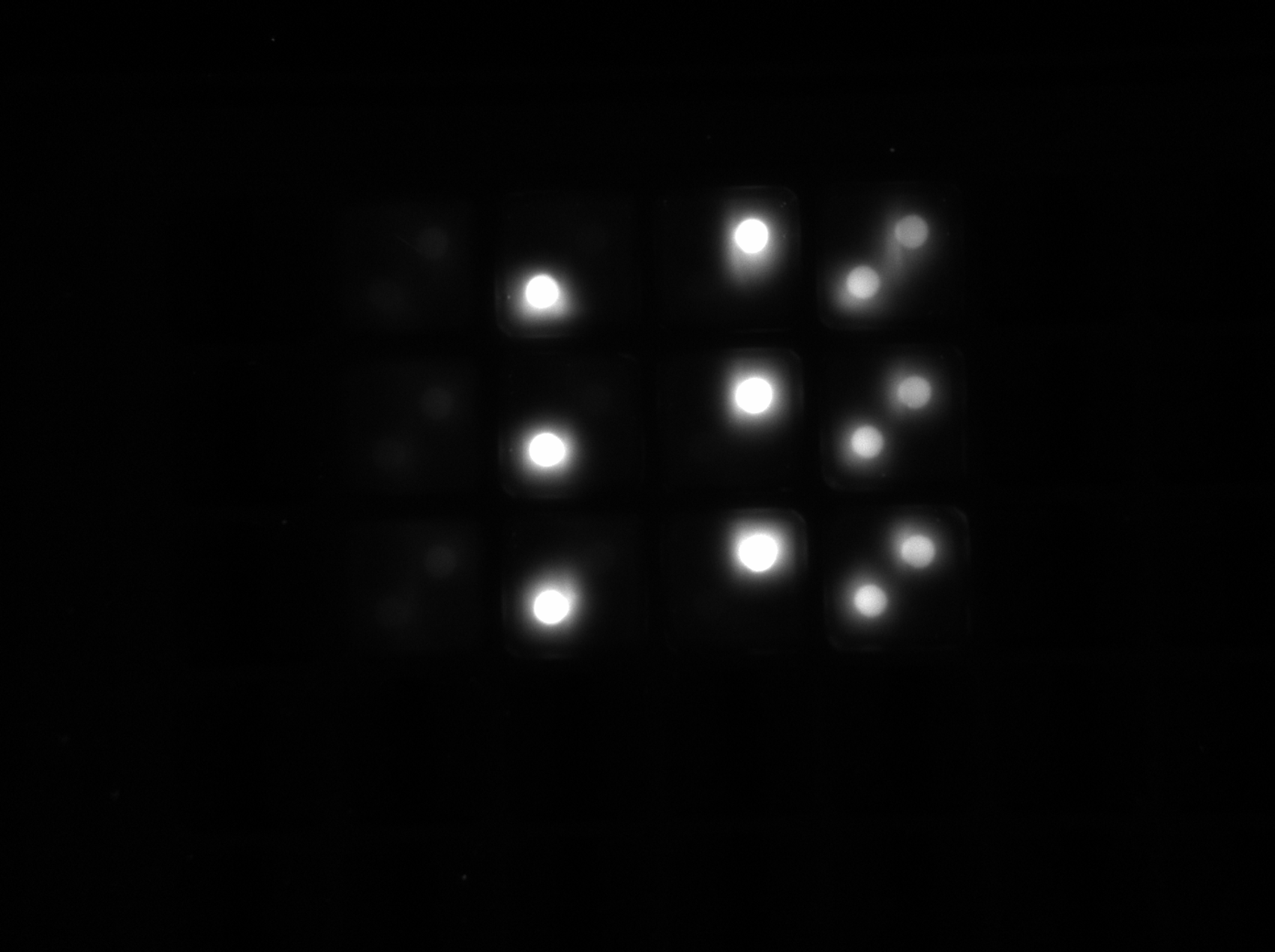

Supplement: Supplementary file 4 — Source Data [file 41467_2021_25989_MOESM4_ESM.zip › Image Files/Fig 4C & Supp Fig 4/RNA 1000nM/RNA_1uM_day2.tif]

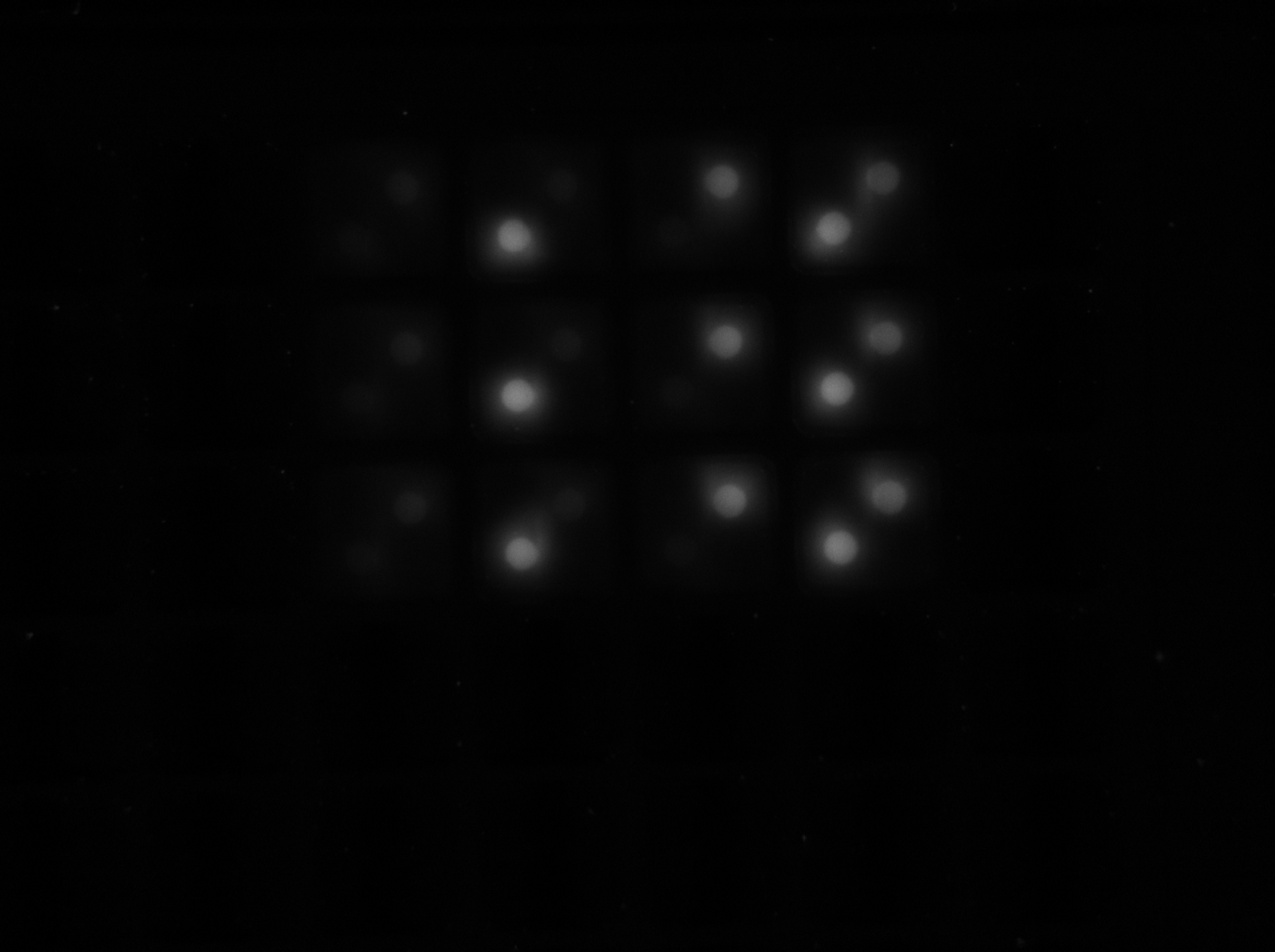

Supplement: Supplementary file 4 — Source Data [file 41467_2021_25989_MOESM4_ESM.zip › Image Files/Fig 4C & Supp Fig 4/RNA 100nM/RNA_100nM_day3.tif]

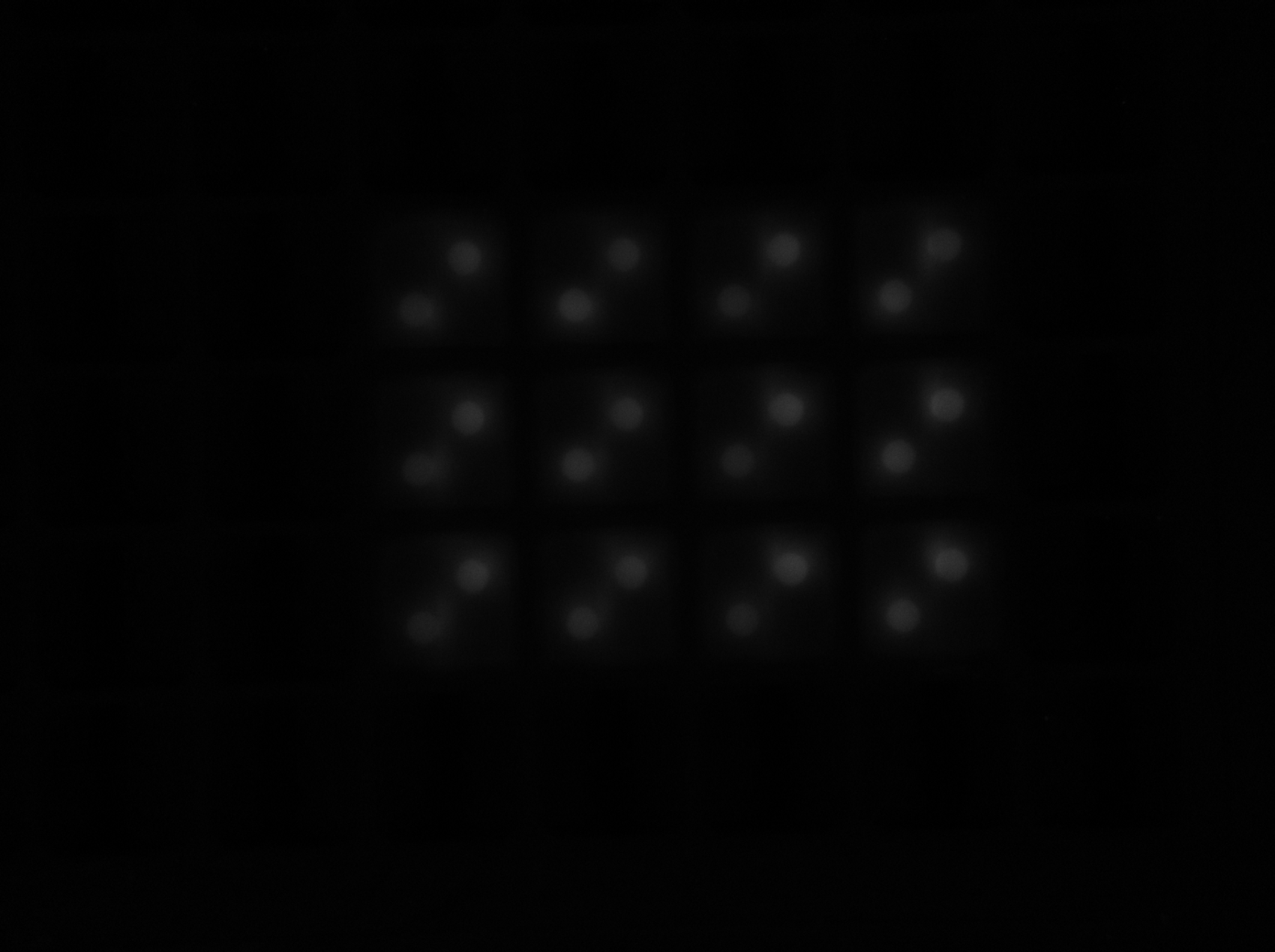

Supplement: Supplementary file 4 — Source Data [file 41467_2021_25989_MOESM4_ESM.zip › Image Files/Fig 4E & Supp Fig 5/DNA 20pM/LnDNA_20pM_day2.tif]

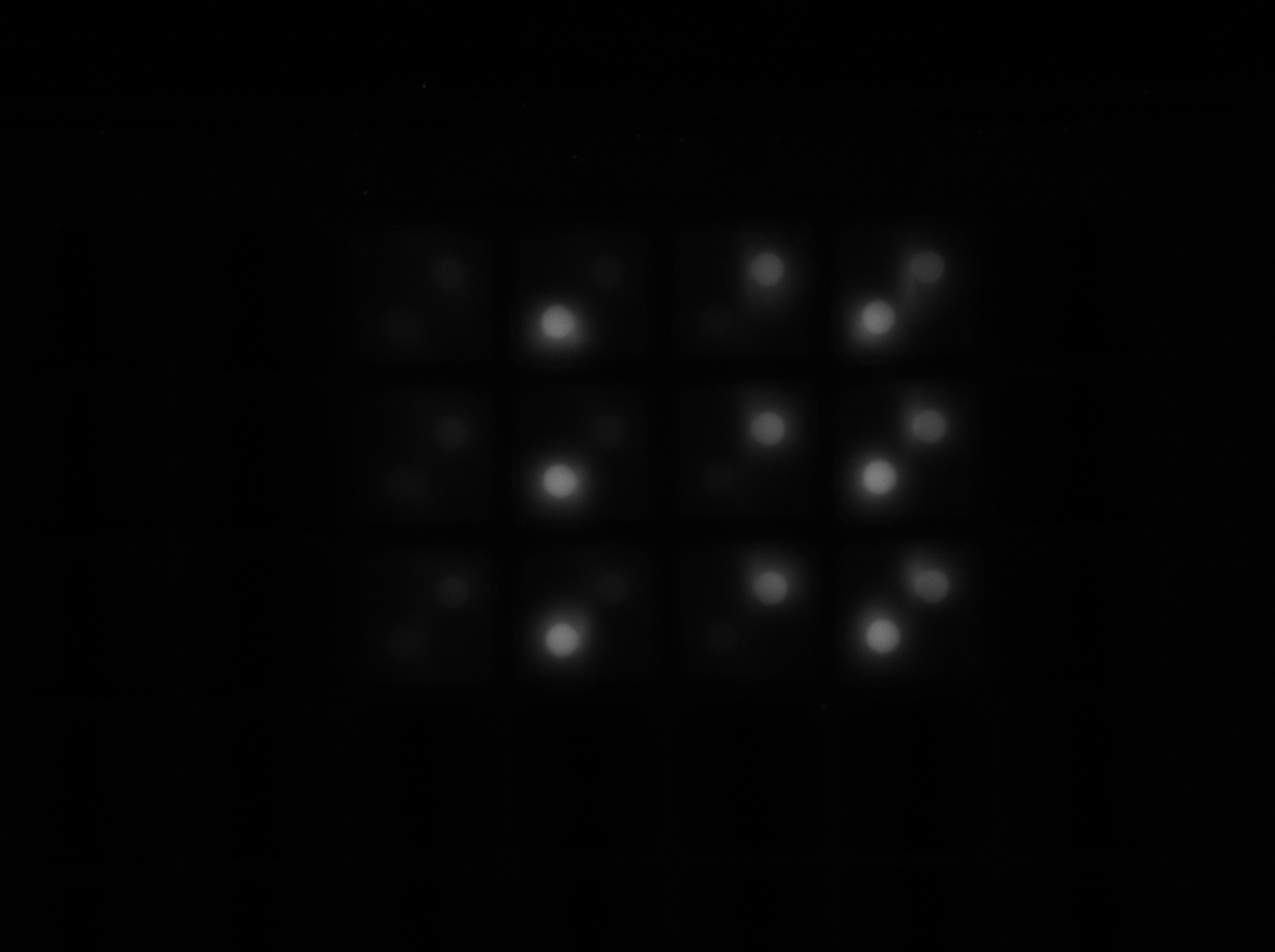

Supplement: Supplementary file 4 — Source Data [file 41467_2021_25989_MOESM4_ESM.zip › Image Files/Fig 4C & Supp Fig 4/RNA 100nM/RNA_100nM_day2.tif]

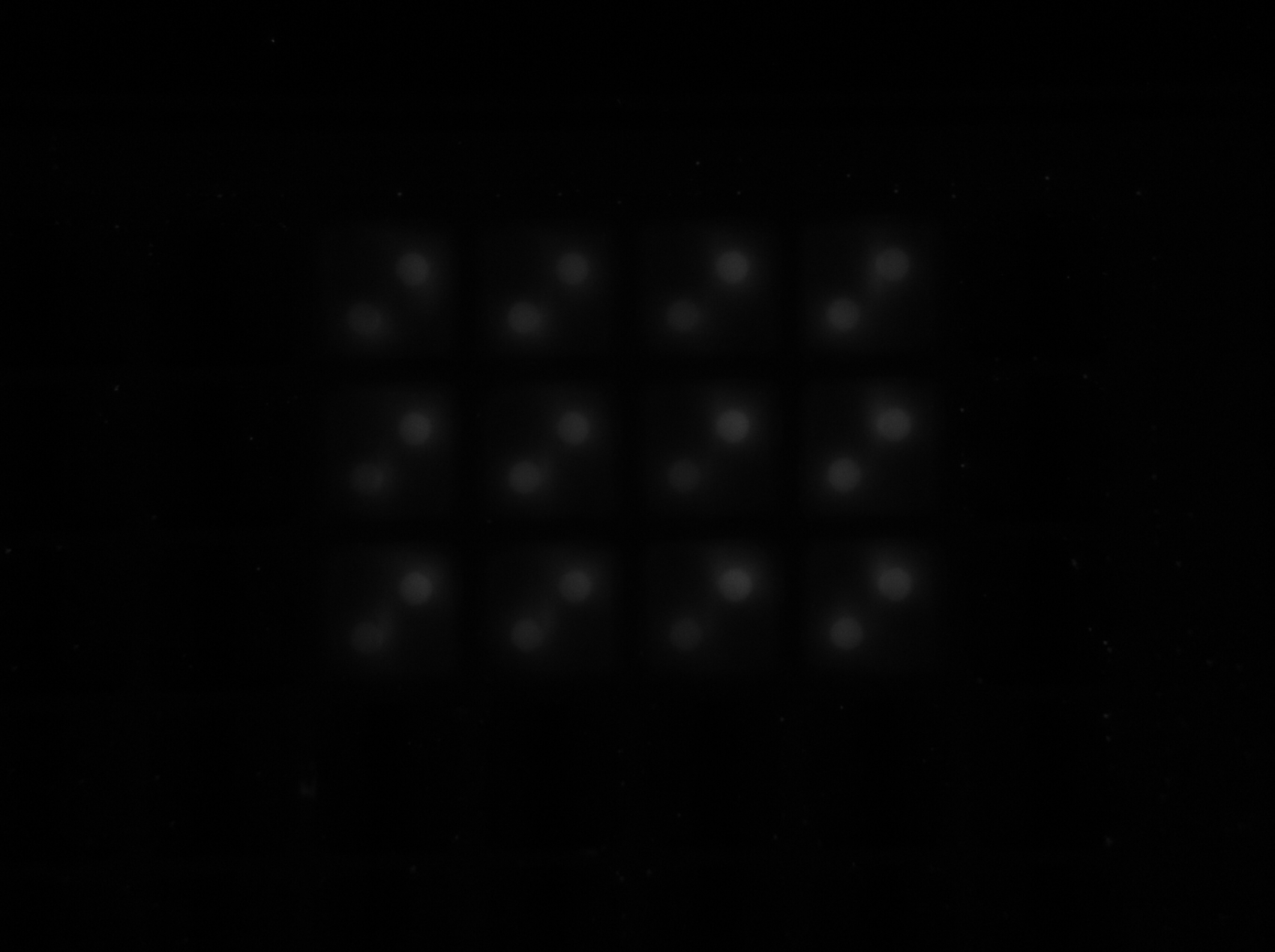

Supplement: Supplementary file 4 — Source Data [file 41467_2021_25989_MOESM4_ESM.zip › Image Files/Fig 4E & Supp Fig 5/DNA 20pM/LnDNA_20pM_day3.tif]

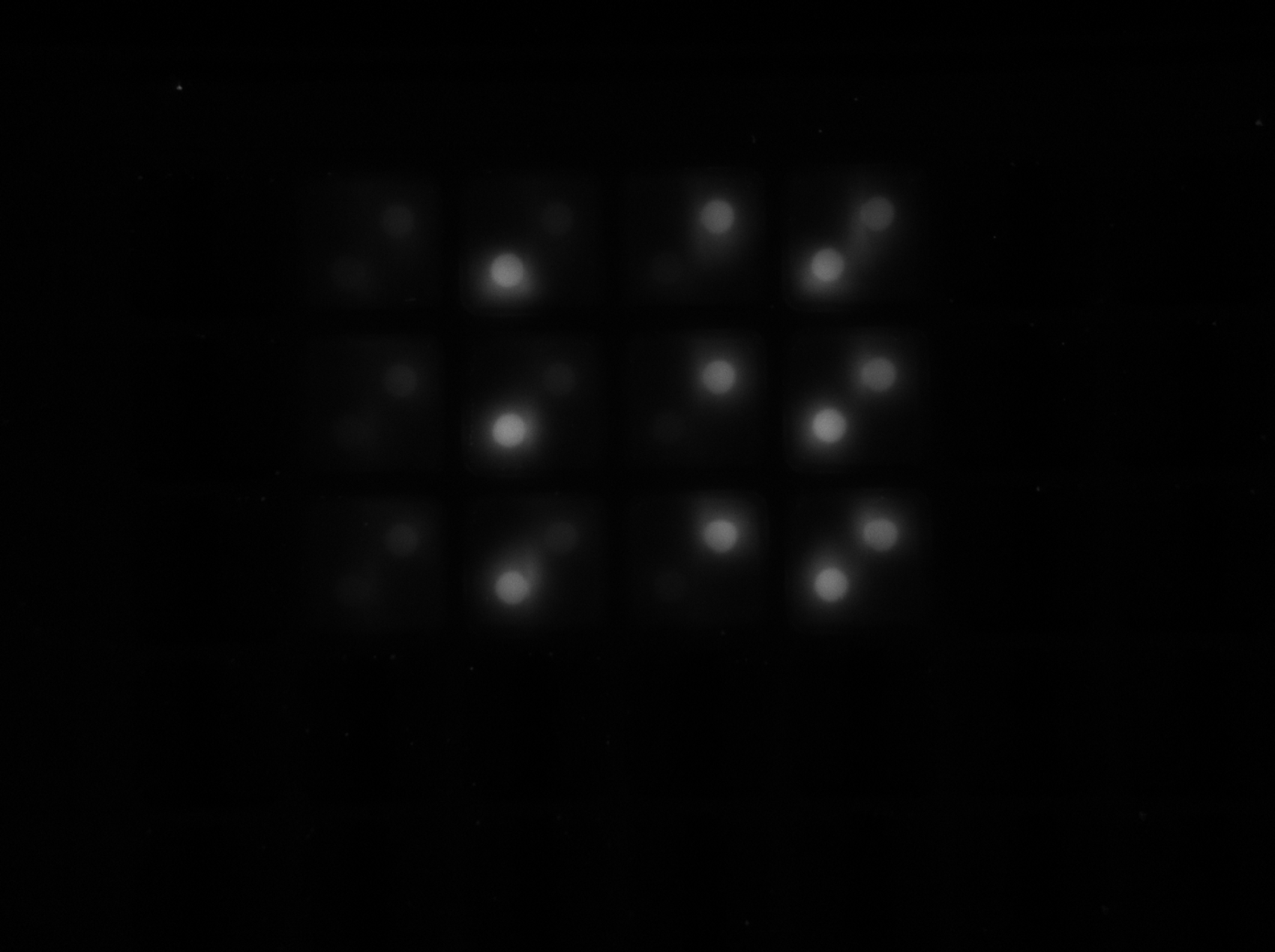

Supplement: Supplementary file 4 — Source Data [file 41467_2021_25989_MOESM4_ESM.zip › Image Files/Fig 4C & Supp Fig 4/RNA 100nM/RNA_100nM_day1.tif]

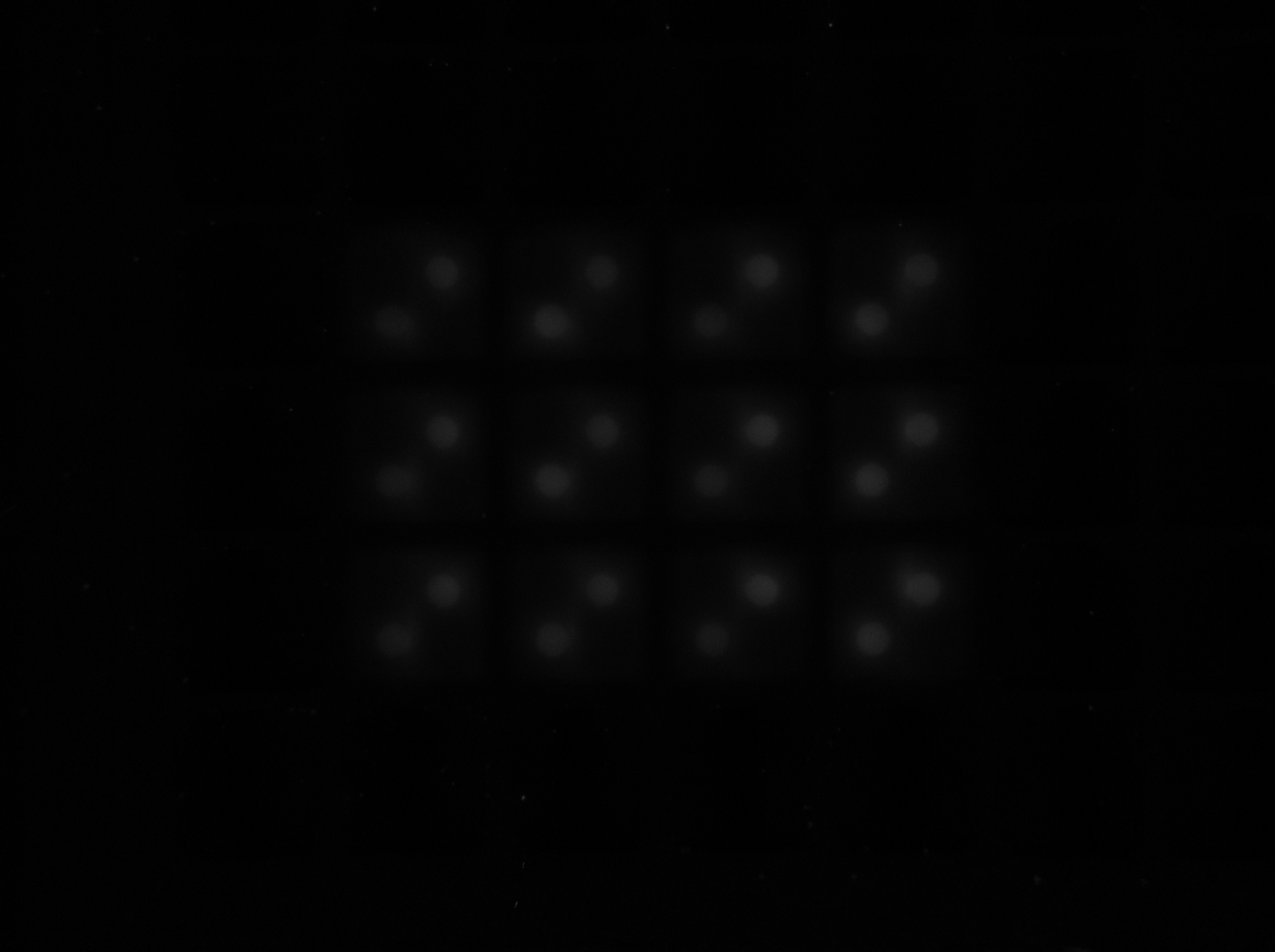

Supplement: Supplementary file 4 — Source Data [file 41467_2021_25989_MOESM4_ESM.zip › Image Files/Fig 4E & Supp Fig 5/DNA 20pM/LnDNA_20pM_day1.tif]

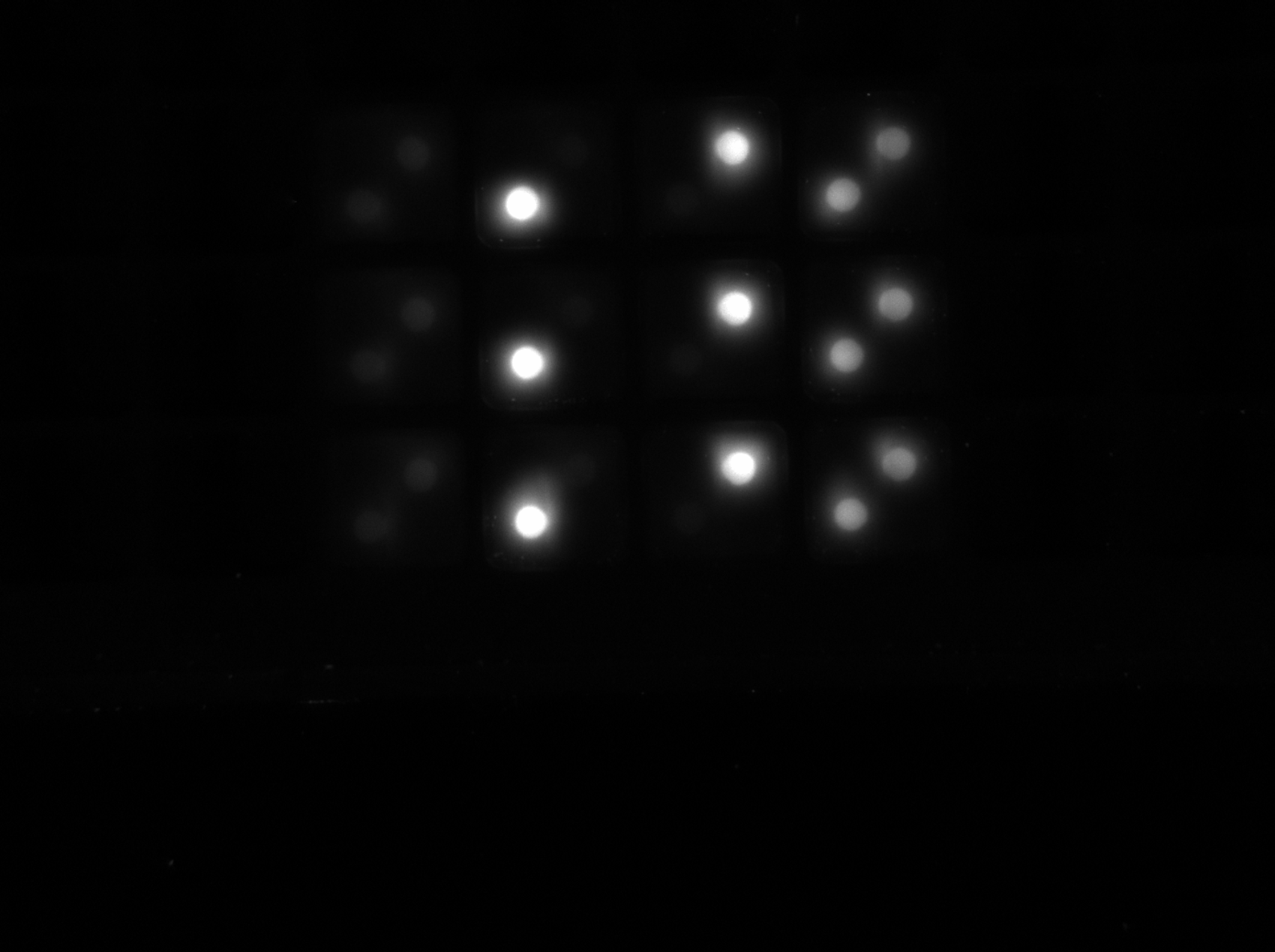

Supplement: Supplementary file 4 — Source Data [file 41467_2021_25989_MOESM4_ESM.zip › Image Files/Fig 4E & Supp Fig 5/DNA 2000pM/LnDNA_2nM_day2.tif]

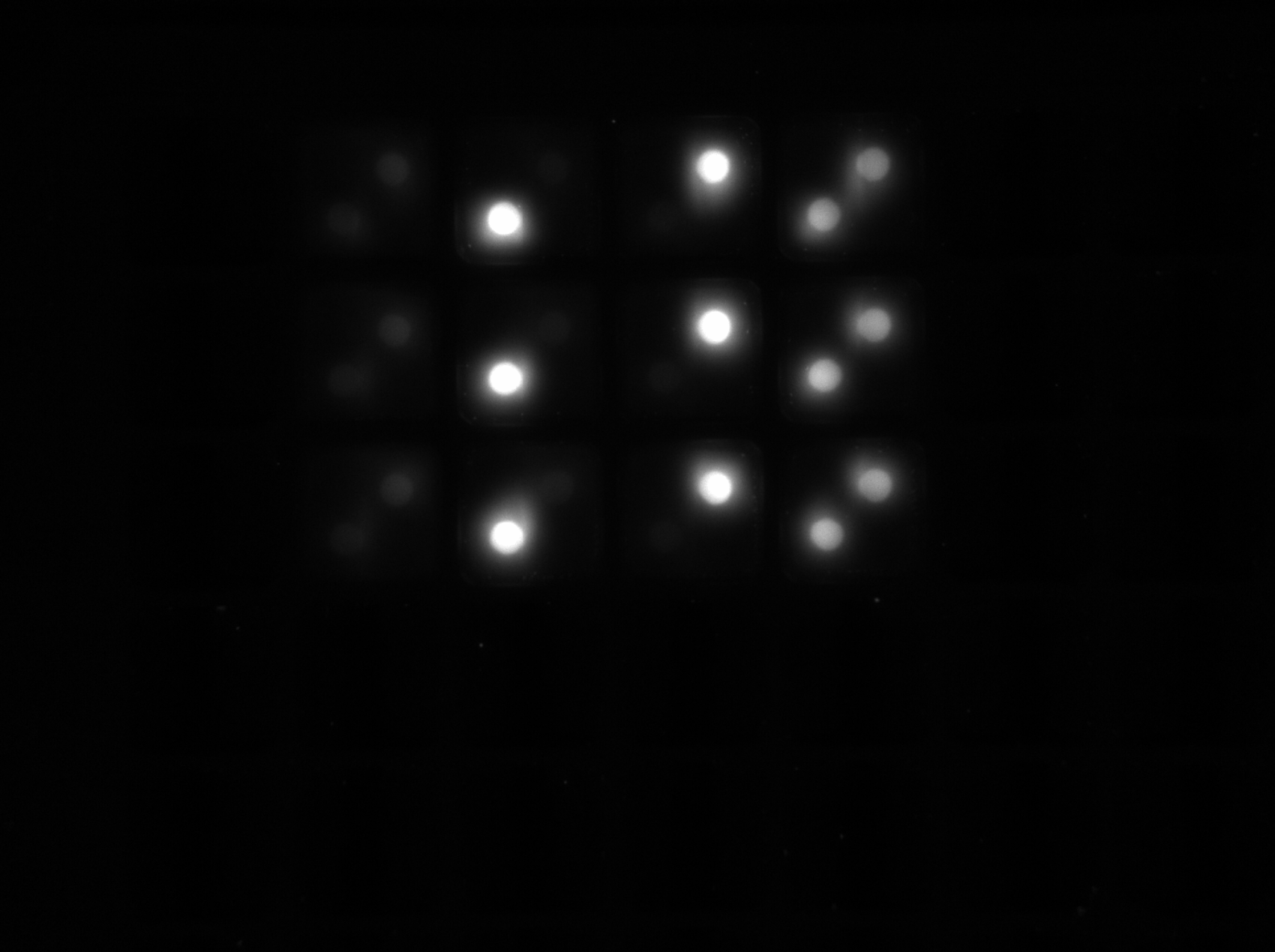

Supplement: Supplementary file 4 — Source Data [file 41467_2021_25989_MOESM4_ESM.zip › Image Files/Fig 4E & Supp Fig 5/DNA 2000pM/LnDNA_2nM_day3.tif]

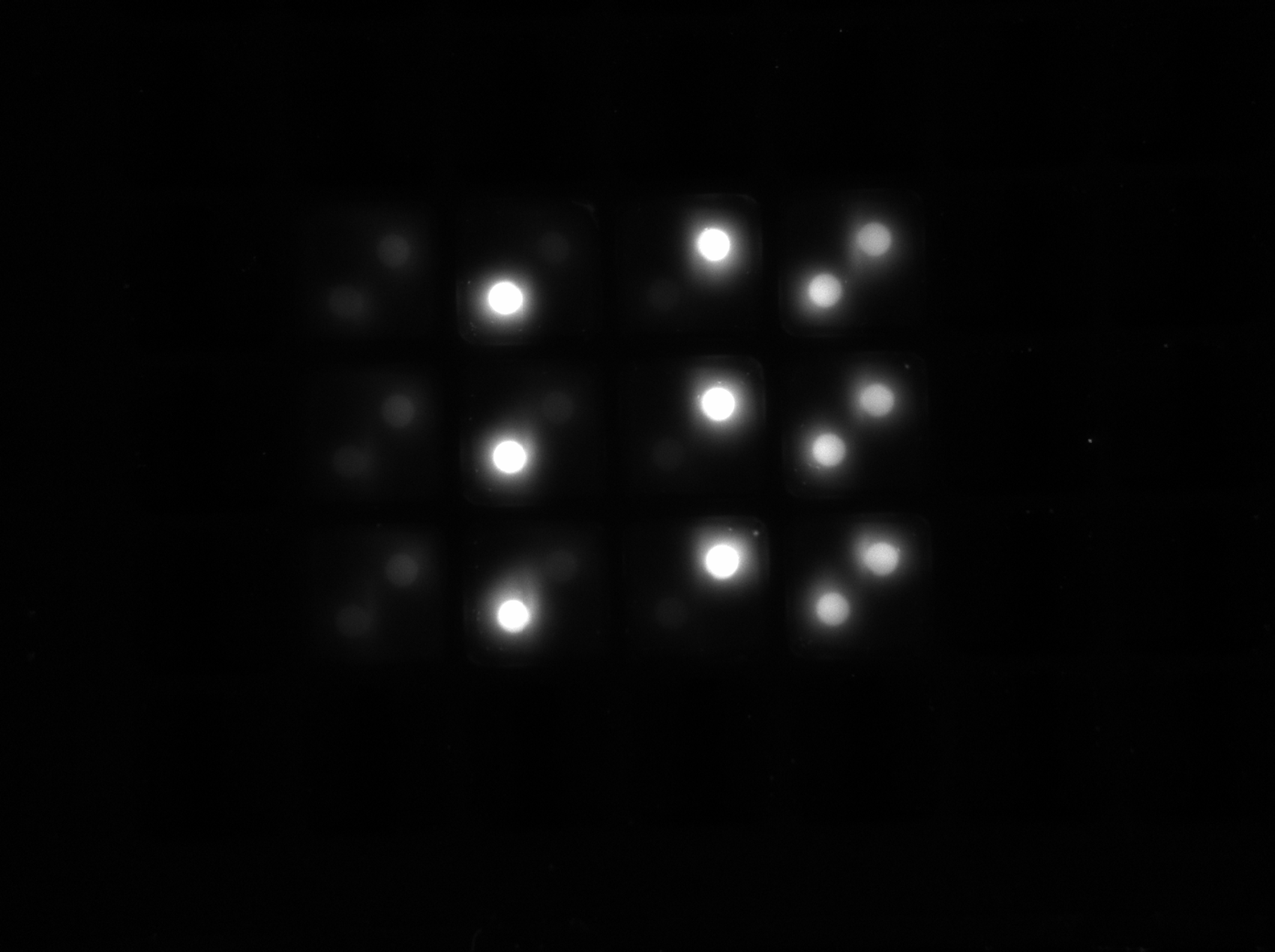

Supplement: Supplementary file 4 — Source Data [file 41467_2021_25989_MOESM4_ESM.zip › Image Files/Fig 4E & Supp Fig 5/DNA 2000pM/LnDNA_2nM_day1.tif]

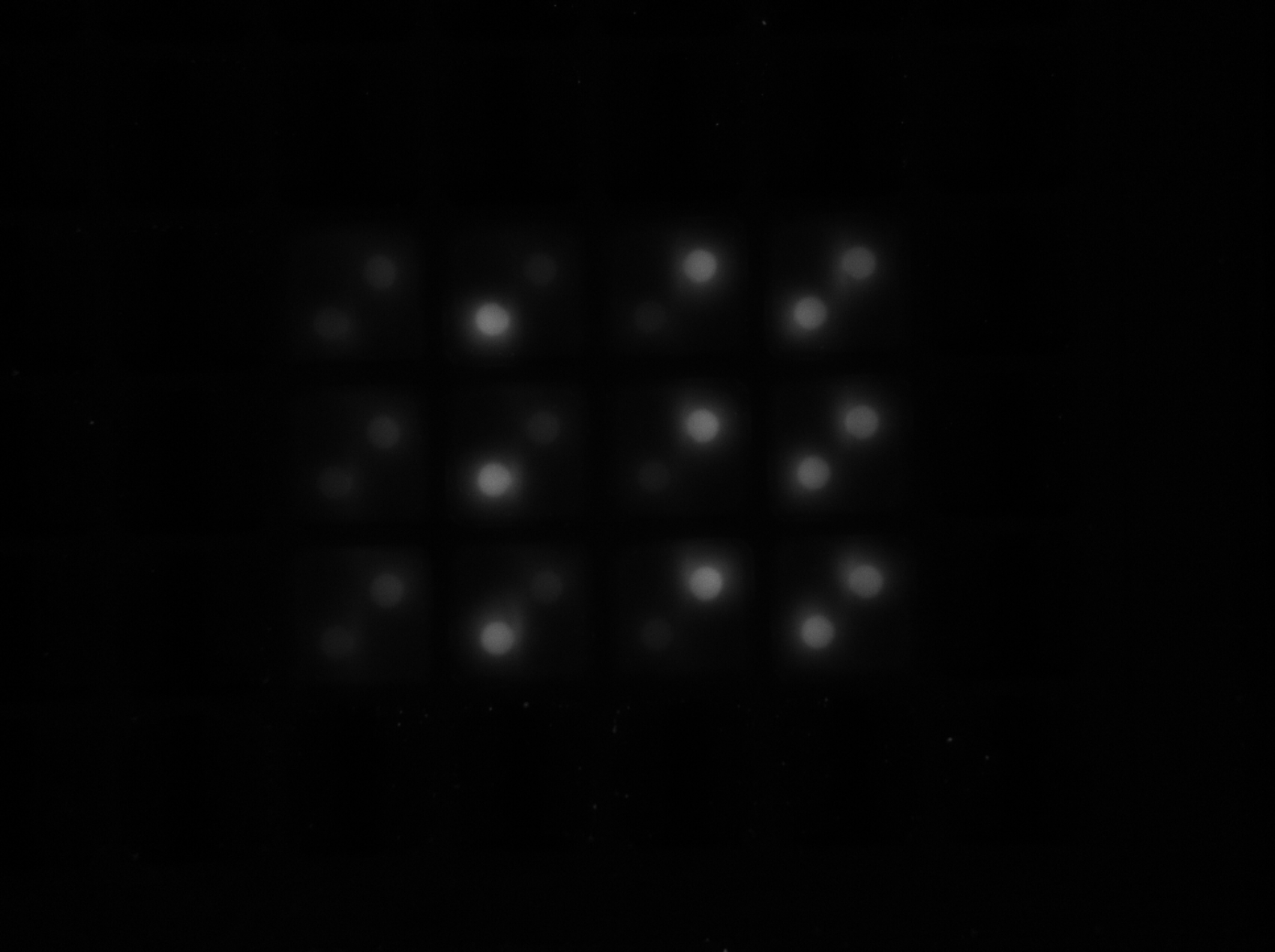

Supplement: Supplementary file 4 — Source Data [file 41467_2021_25989_MOESM4_ESM.zip › Image Files/Fig 4E & Supp Fig 5/DNA 200pM/LnDNA_200pM_day2.tif]

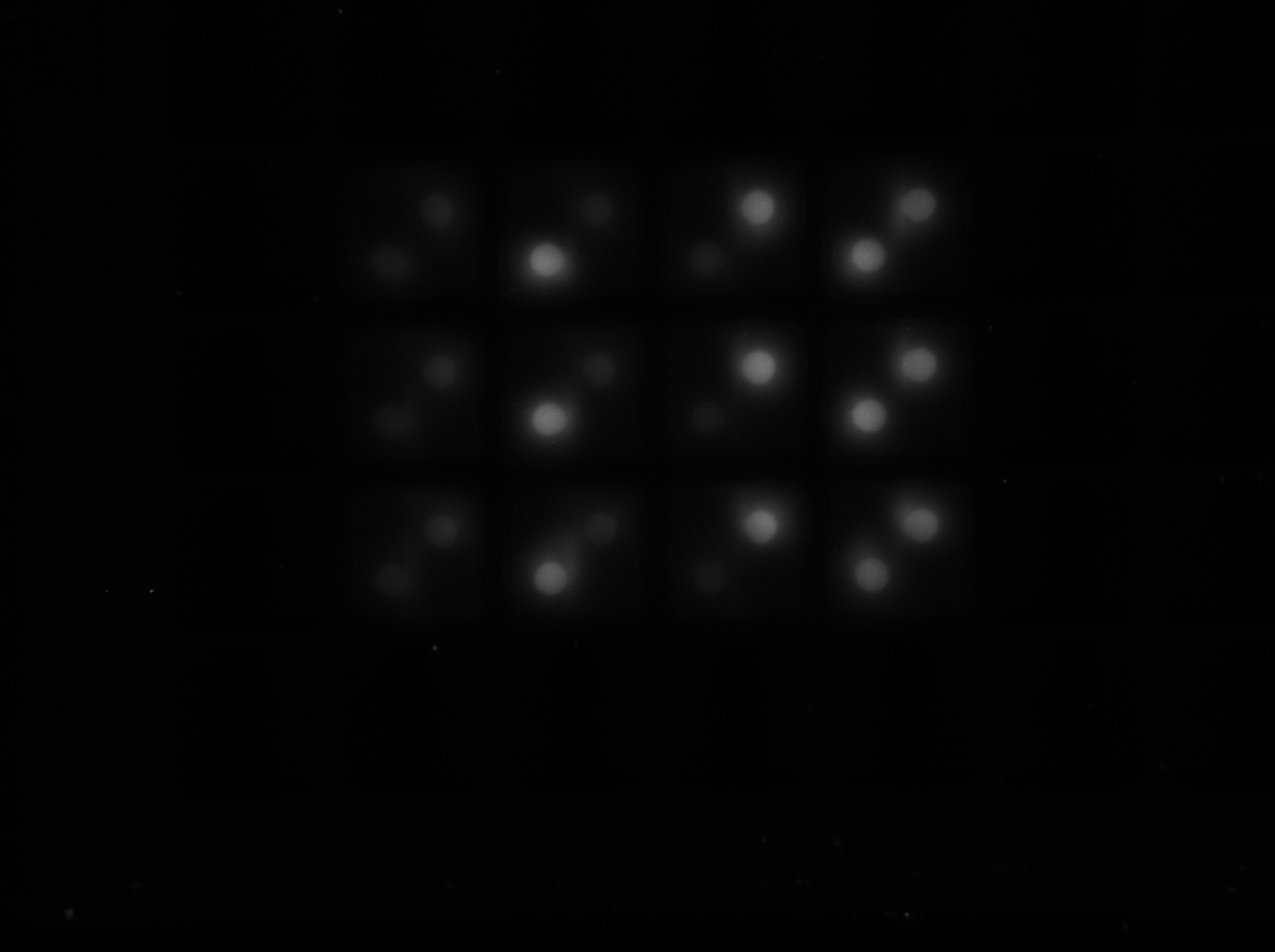

Supplement: Supplementary file 4 — Source Data [file 41467_2021_25989_MOESM4_ESM.zip › Image Files/Fig 4E & Supp Fig 5/DNA 200pM/LnDNA_200pM_day1.tif]

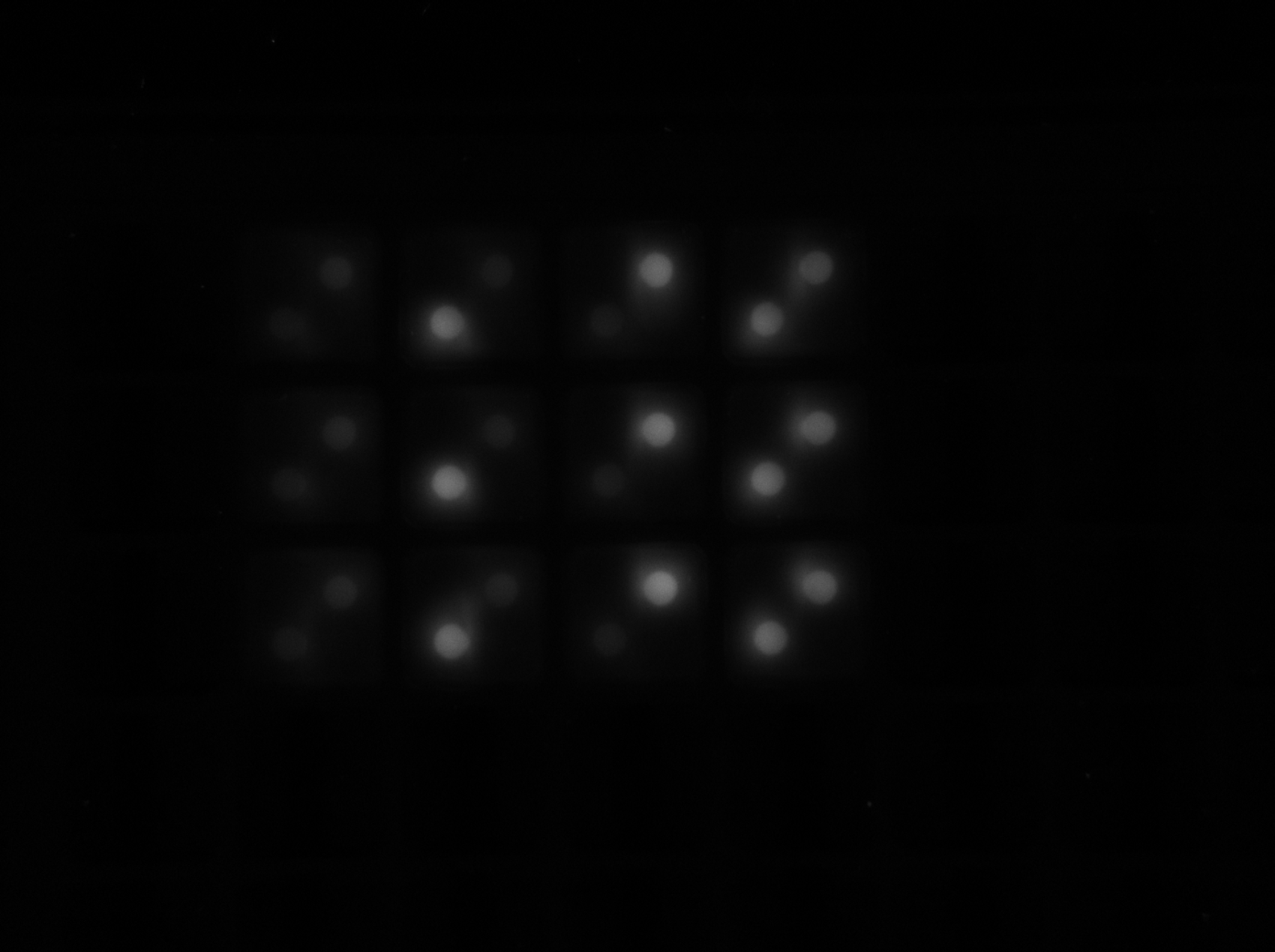

Supplement: Supplementary file 4 — Source Data [file 41467_2021_25989_MOESM4_ESM.zip › Image Files/Fig 4E & Supp Fig 5/DNA 200pM/LnDNA_200pM_day3.tif]

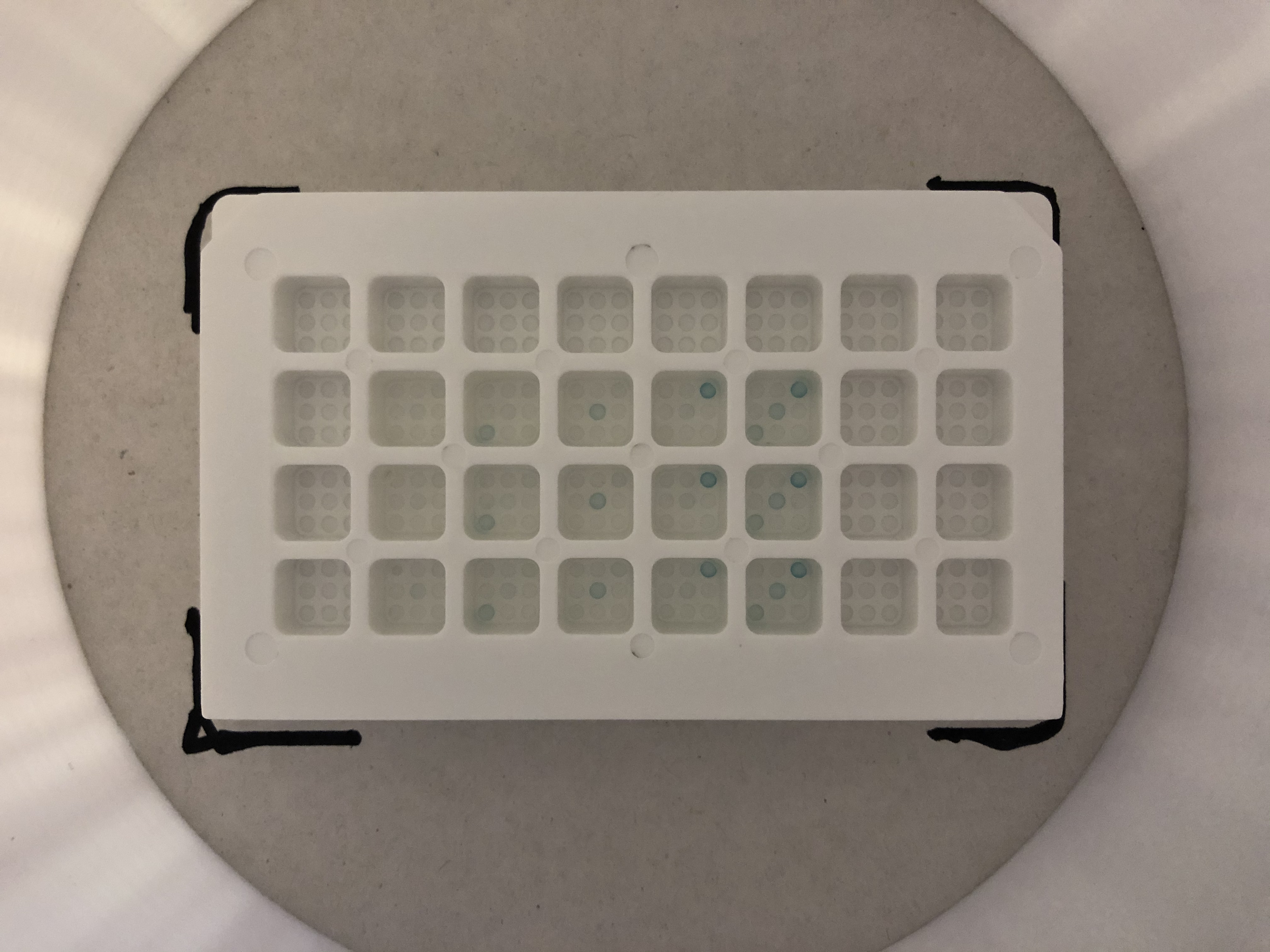

Supplement: Supplementary file 4 — Source Data [file 41467_2021_25989_MOESM4_ESM.zip › Image Files/Fig 6C & Supp Fig 17/FreezeDried_Run2/Run2_FD_2hr.jpeg]

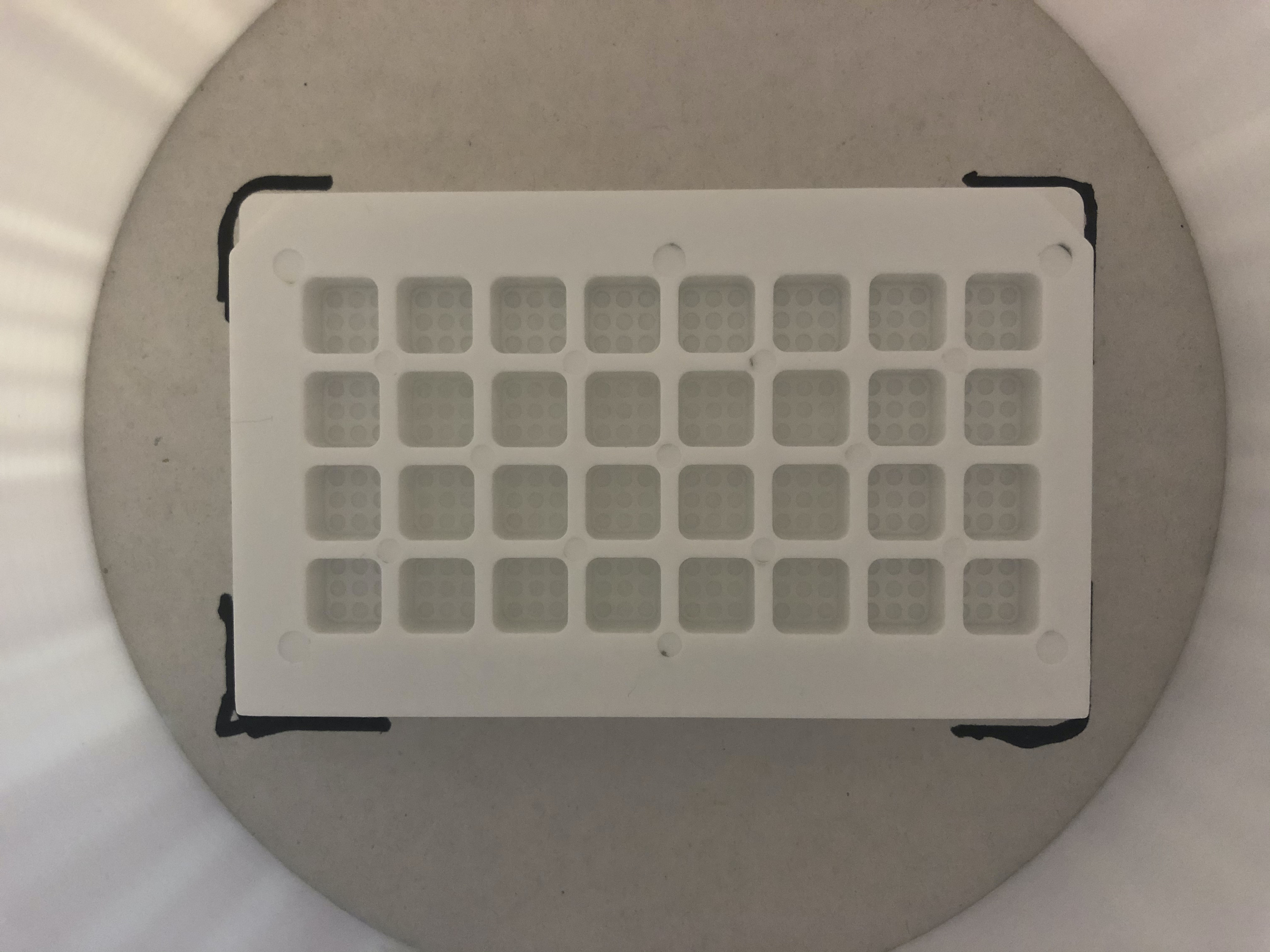

Supplement: Supplementary file 4 — Source Data [file 41467_2021_25989_MOESM4_ESM.zip › Image Files/Fig 6C & Supp Fig 17/FreezeDried_Run1/Run1_FD_0hr.jpeg]

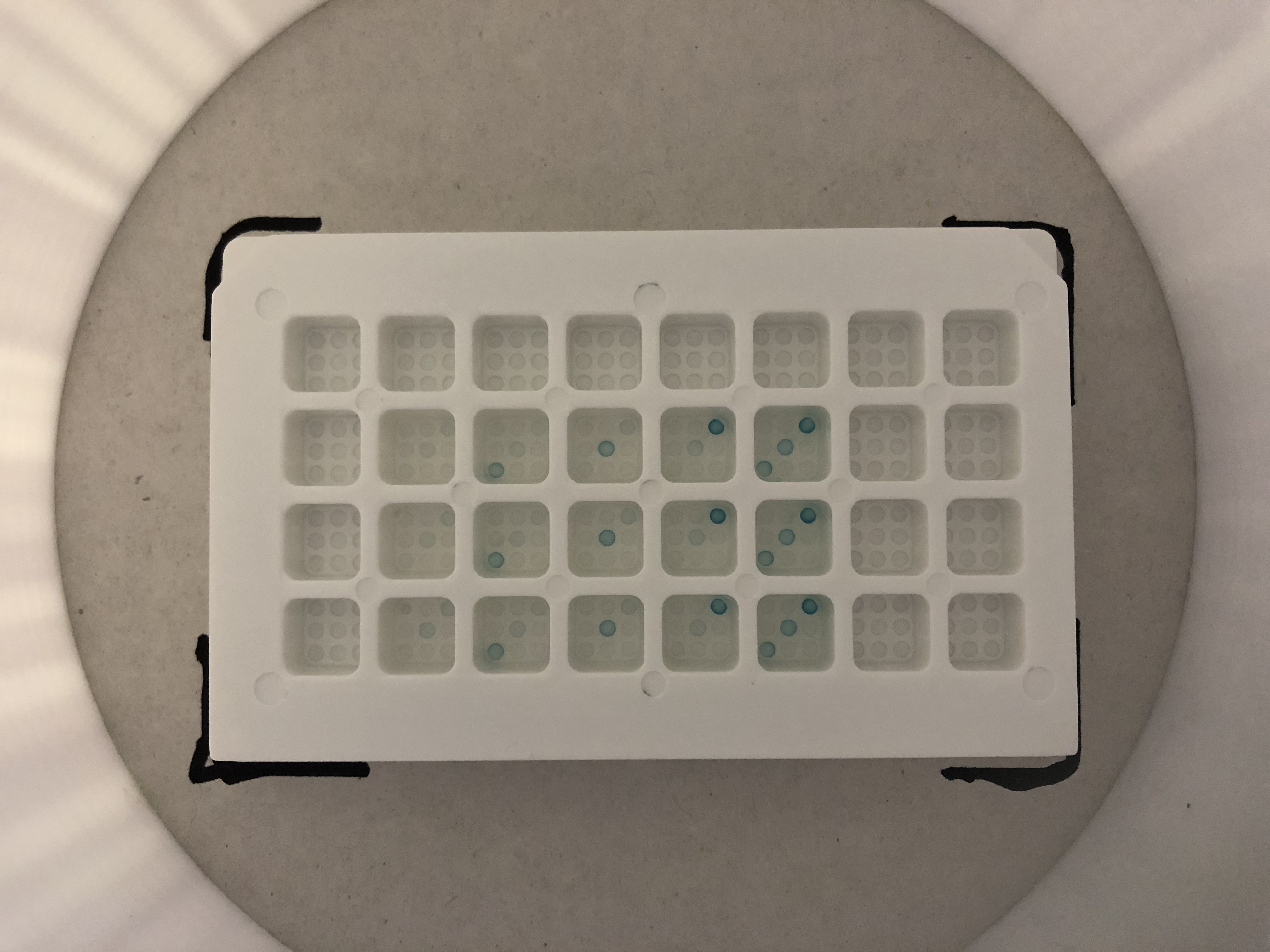

Supplement: Supplementary file 4 — Source Data [file 41467_2021_25989_MOESM4_ESM.zip › Image Files/Fig 6C & Supp Fig 17/FreezeDried_Run2/Run2_FD_3hr.jpeg]

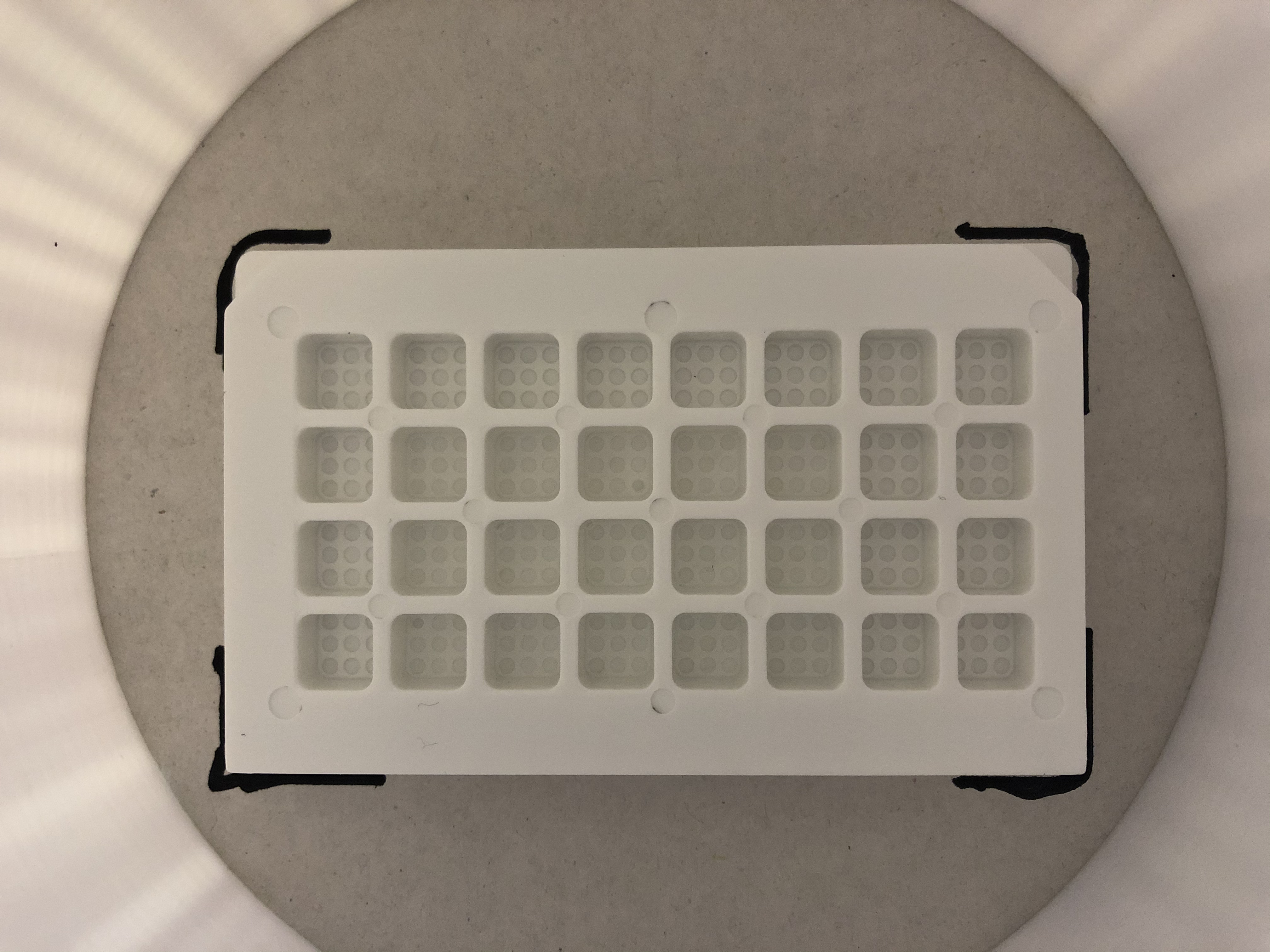

Supplement: Supplementary file 4 — Source Data [file 41467_2021_25989_MOESM4_ESM.zip › Image Files/Fig 6C & Supp Fig 17/FreezeDried_Run3/Run3_FD_0hr.jpeg]

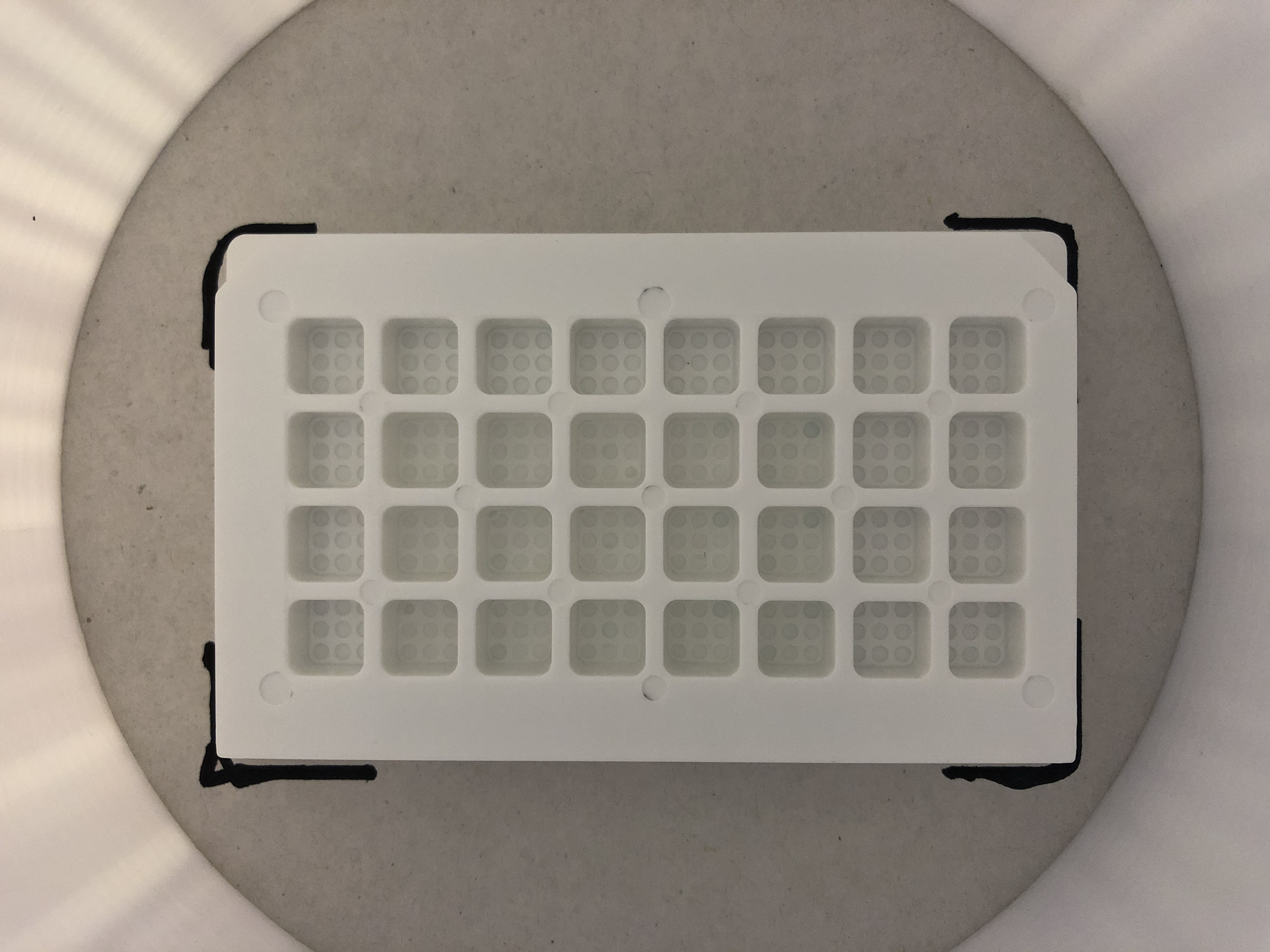

Supplement: Supplementary file 4 — Source Data [file 41467_2021_25989_MOESM4_ESM.zip › Image Files/Fig 6C & Supp Fig 17/FreezeDried_Run3/Run3_FD_1hr.jpeg]

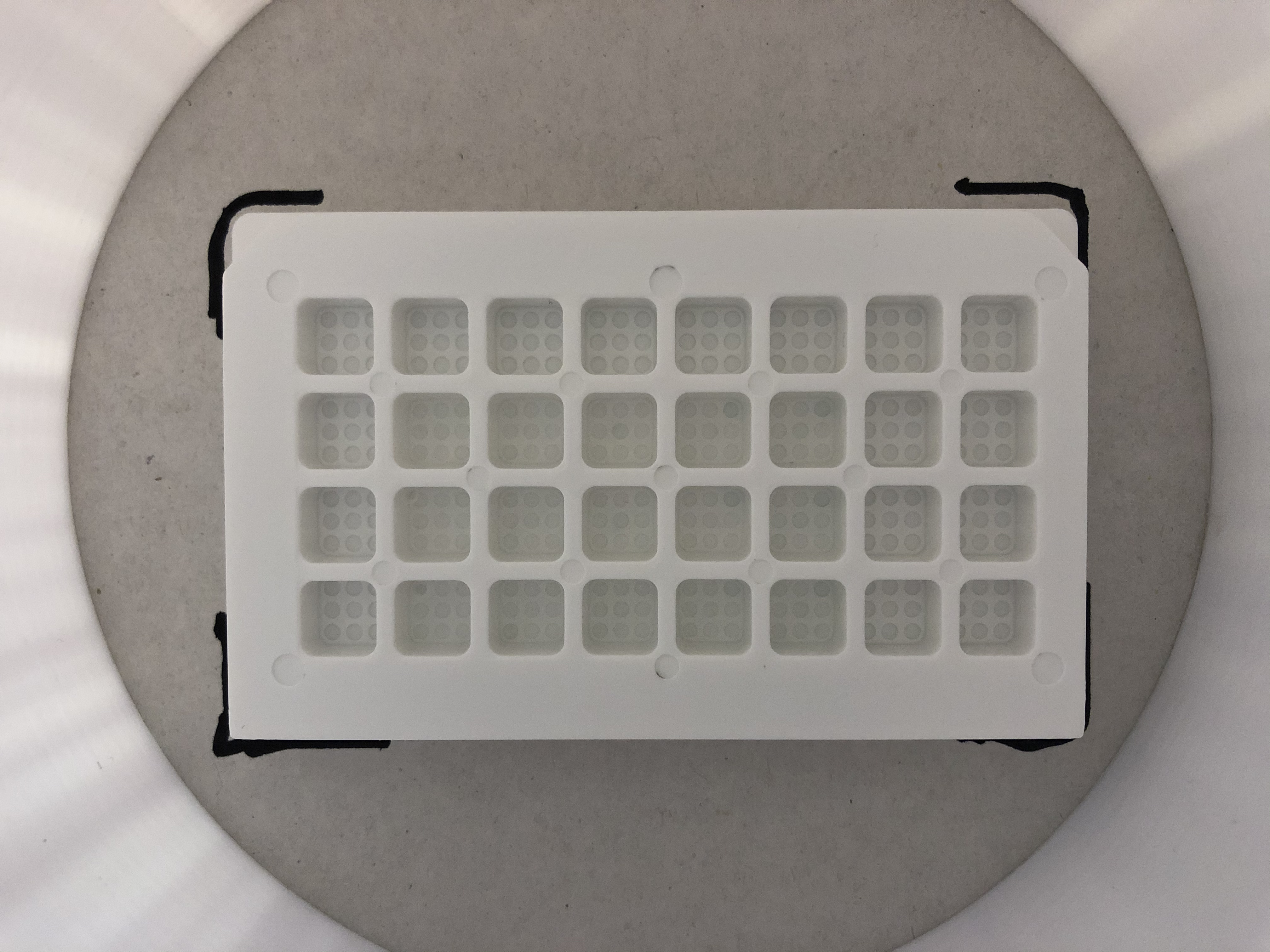

Supplement: Supplementary file 4 — Source Data [file 41467_2021_25989_MOESM4_ESM.zip › Image Files/Fig 6C & Supp Fig 17/FreezeDried_Run2/Run2_FD_1hr.jpeg]

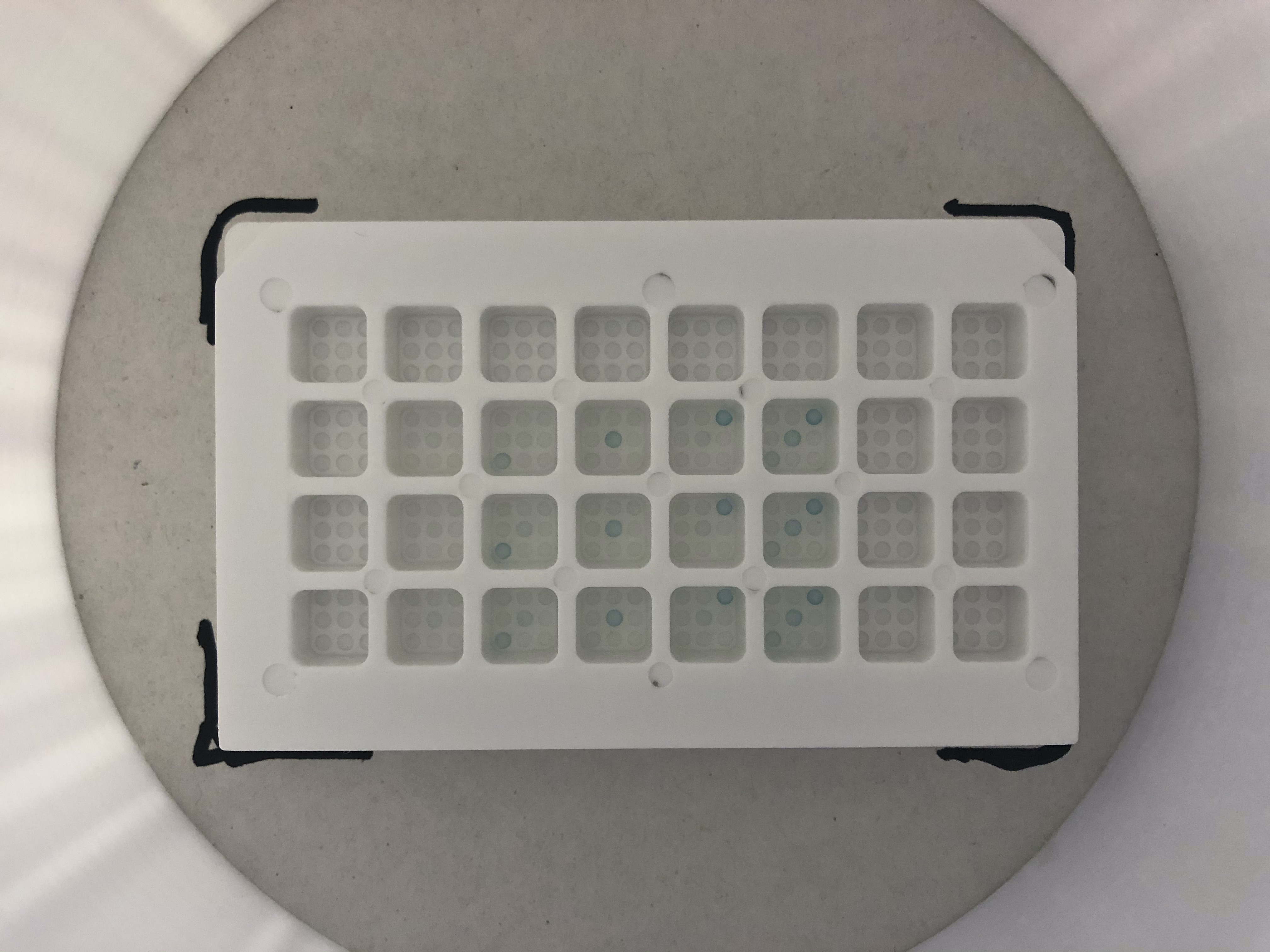

Supplement: Supplementary file 4 — Source Data [file 41467_2021_25989_MOESM4_ESM.zip › Image Files/Fig 6C & Supp Fig 17/FreezeDried_Run1/Run1_FD_2hr.jpeg]

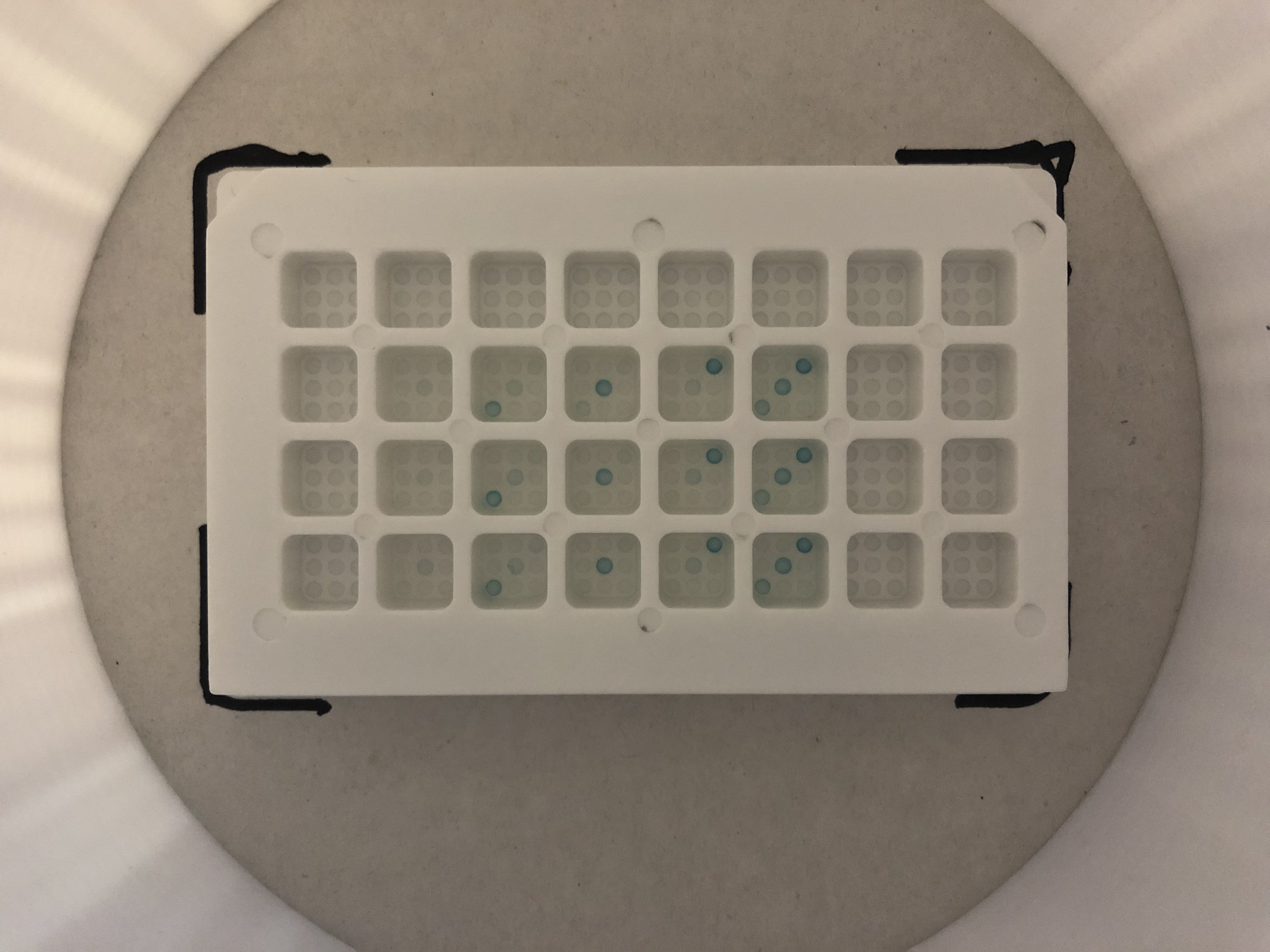

Supplement: Supplementary file 4 — Source Data [file 41467_2021_25989_MOESM4_ESM.zip › Image Files/Fig 6C & Supp Fig 17/FreezeDried_Run1/Run1_FD_3hr.jpeg]

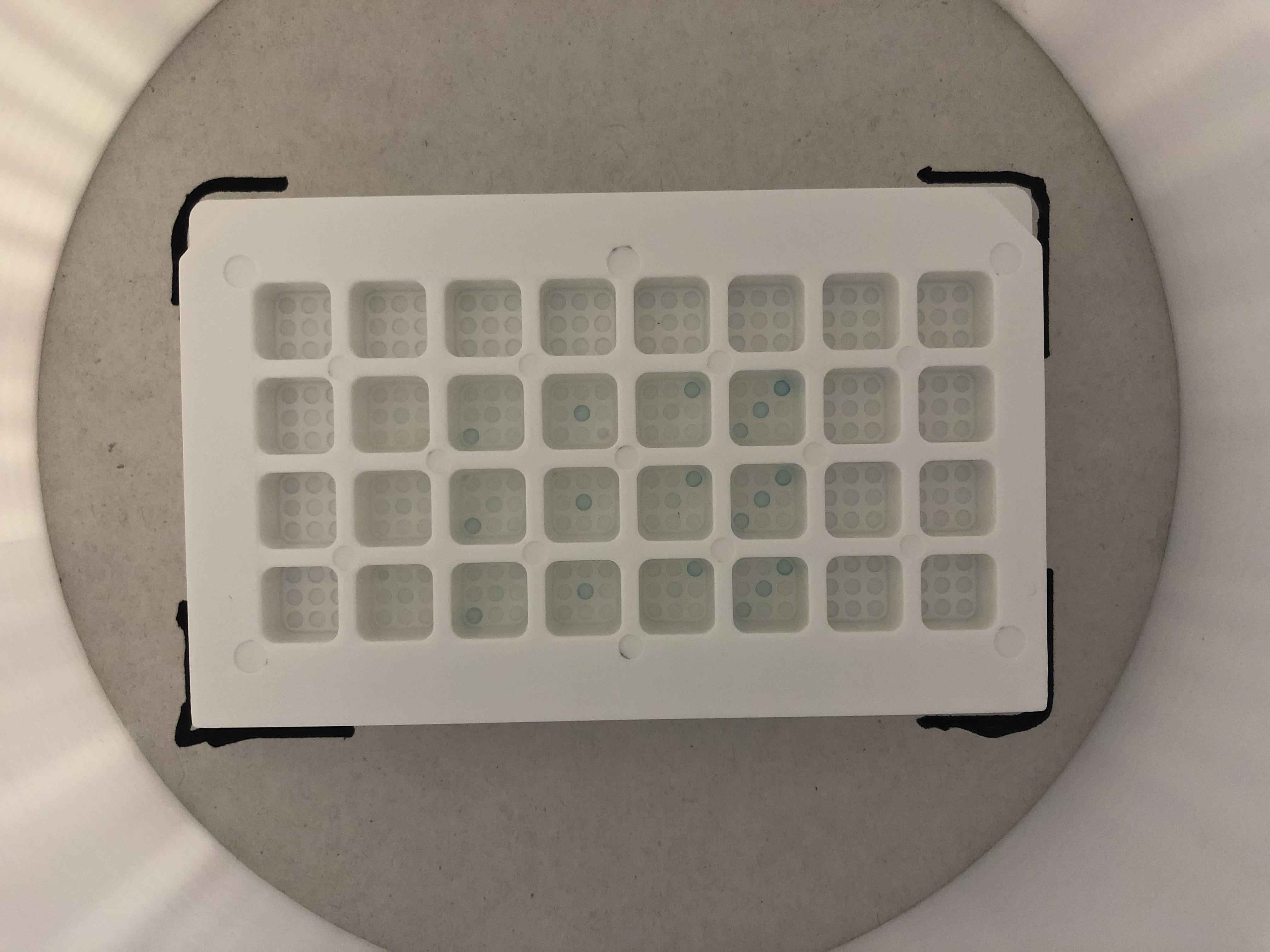

Supplement: Supplementary file 4 — Source Data [file 41467_2021_25989_MOESM4_ESM.zip › Image Files/Fig 6C & Supp Fig 17/FreezeDried_Run3/Run3_FD_2hr.jpeg]

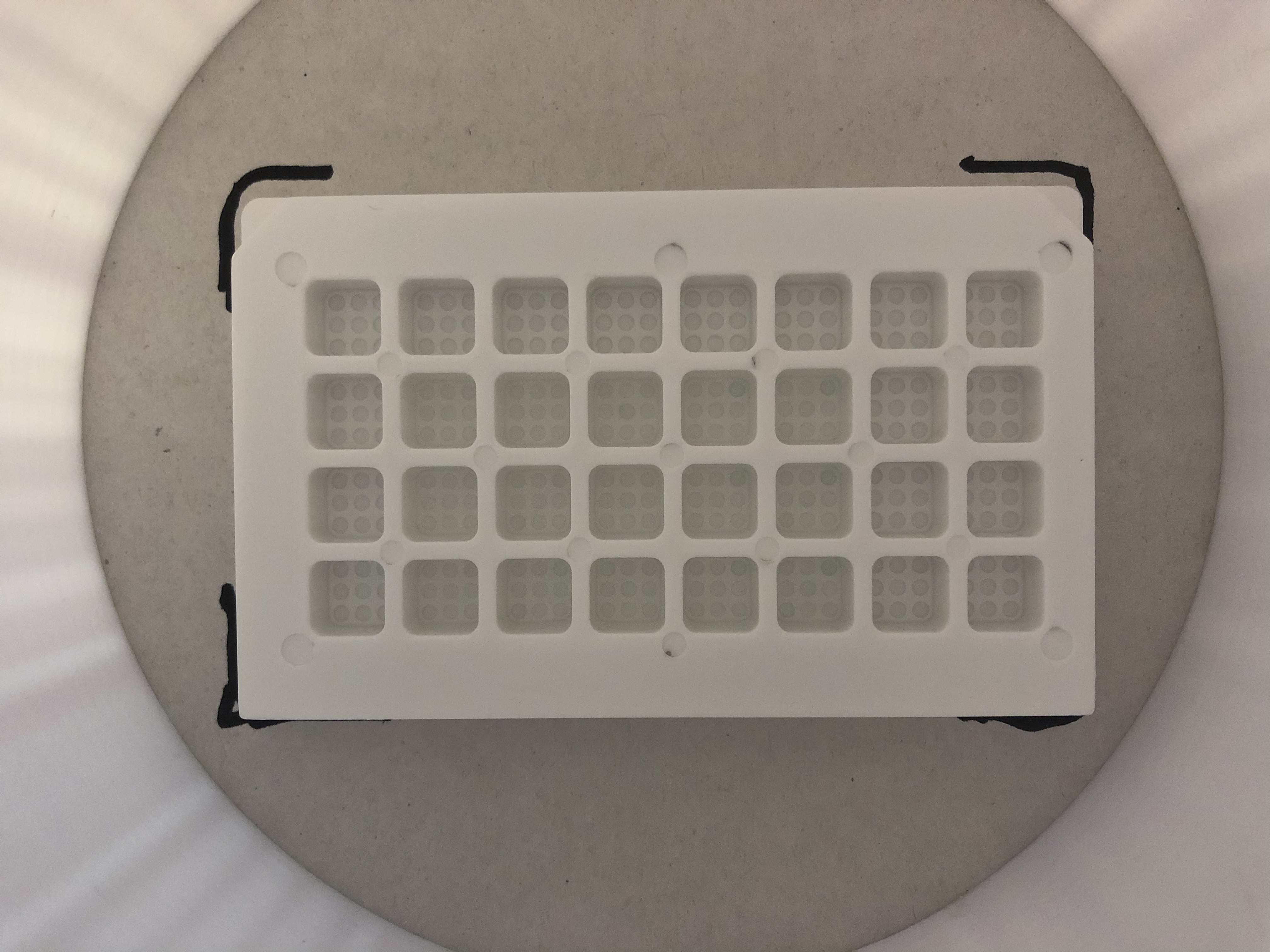

Supplement: Supplementary file 4 — Source Data [file 41467_2021_25989_MOESM4_ESM.zip › Image Files/Fig 6C & Supp Fig 17/FreezeDried_Run1/Run1_FD_1hr.jpeg]

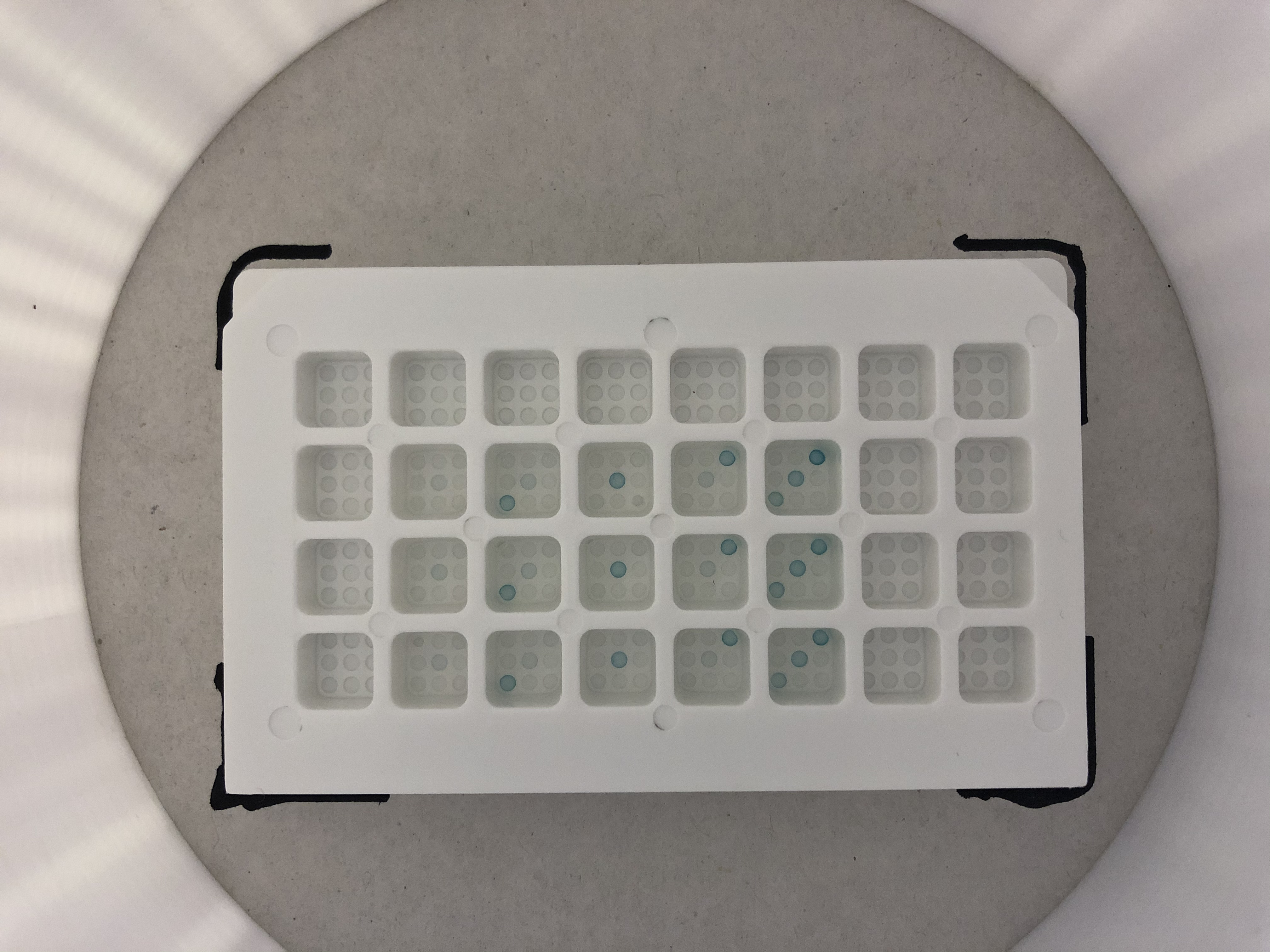

Supplement: Supplementary file 4 — Source Data [file 41467_2021_25989_MOESM4_ESM.zip › Image Files/Fig 6C & Supp Fig 17/FreezeDried_Run3/Run3_FD_3hr.jpeg]

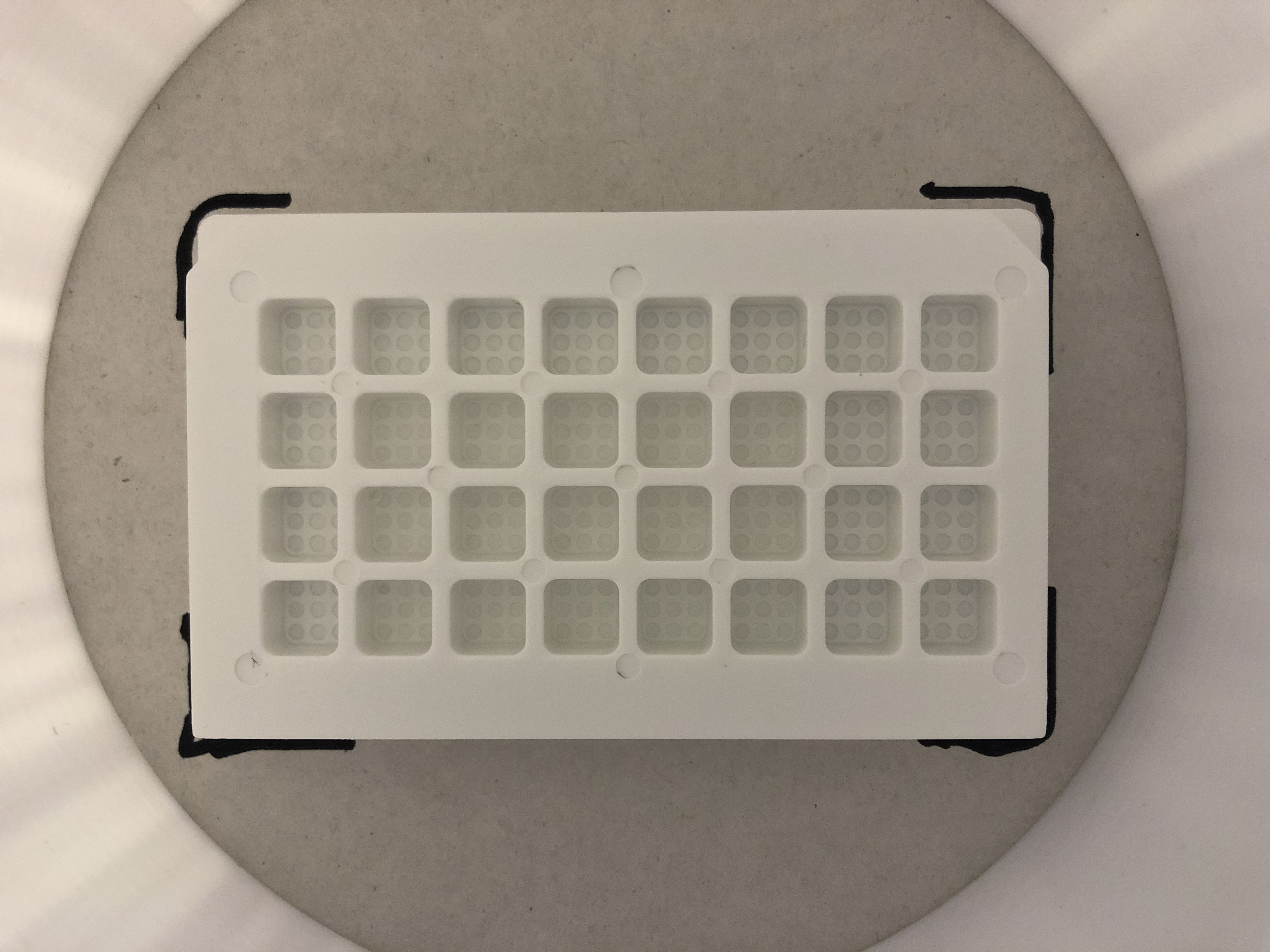

Supplement: Supplementary file 4 — Source Data [file 41467_2021_25989_MOESM4_ESM.zip › Image Files/Fig 6C & Supp Fig 17/FreezeDried_Run2/Run2_FD_0hr.jpeg]

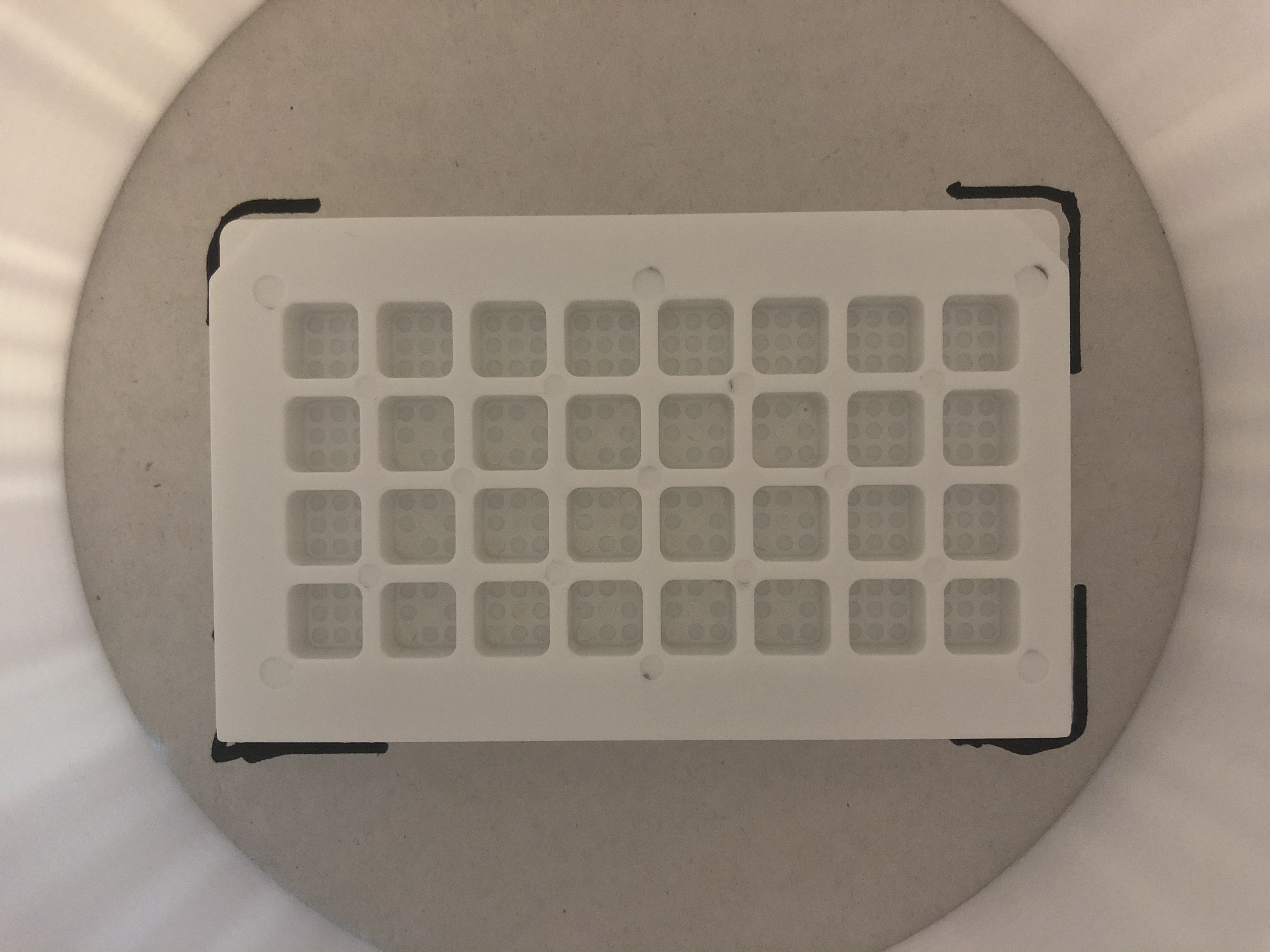

Supplement: Supplementary file 4 — Source Data [file 41467_2021_25989_MOESM4_ESM.zip › Image Files/Fig 6C & Supp Fig 17/FreezeDried_Run1/Run1_FD_T0_plate.jpeg]

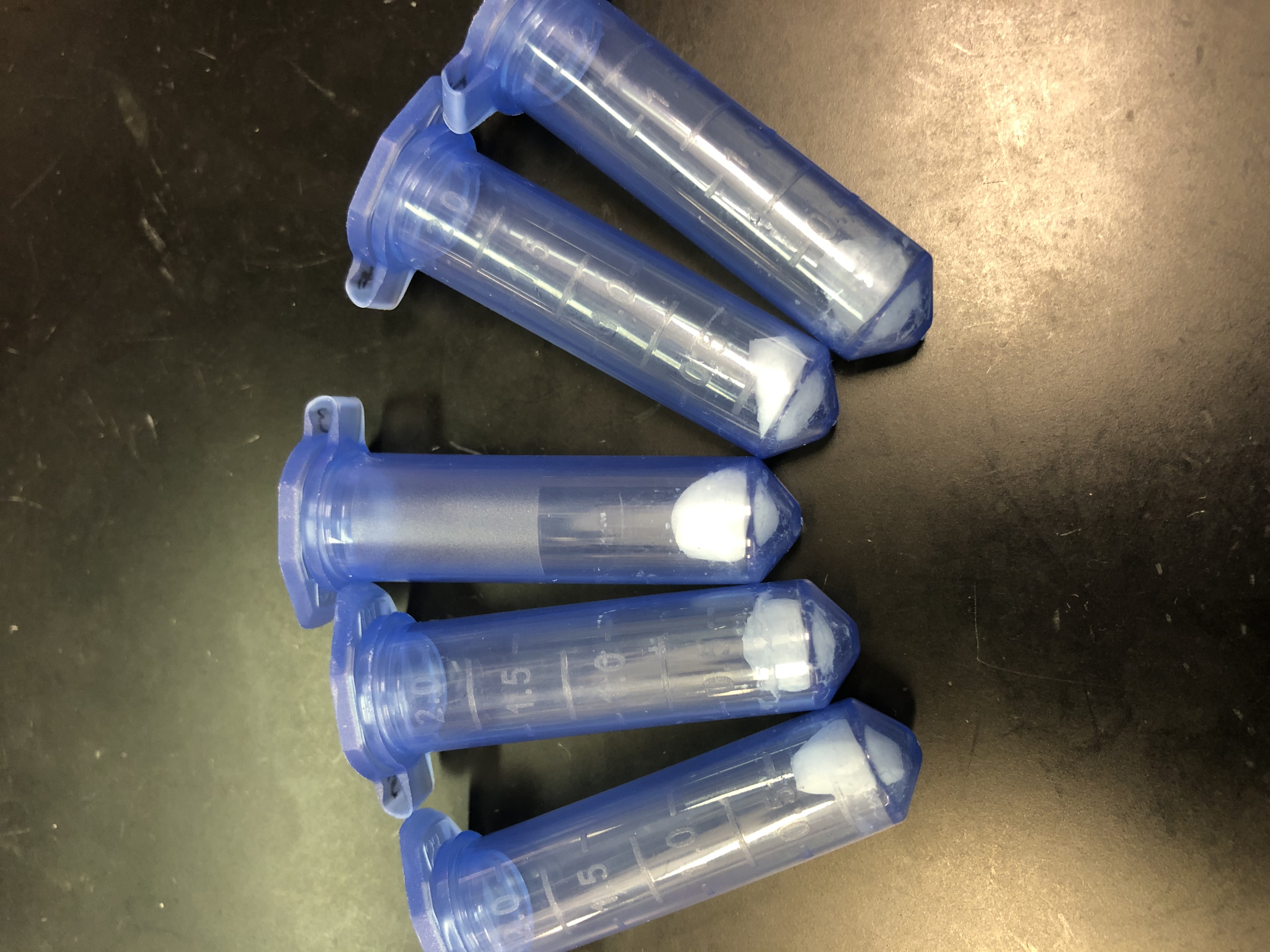

Supplement: Supplementary file 4 — Source Data [file 41467_2021_25989_MOESM4_ESM.zip › Image Files/Fig 6C & Supp Fig 17/FreezeDried_Run2/Run2_FD_T0_tubes.jpeg]

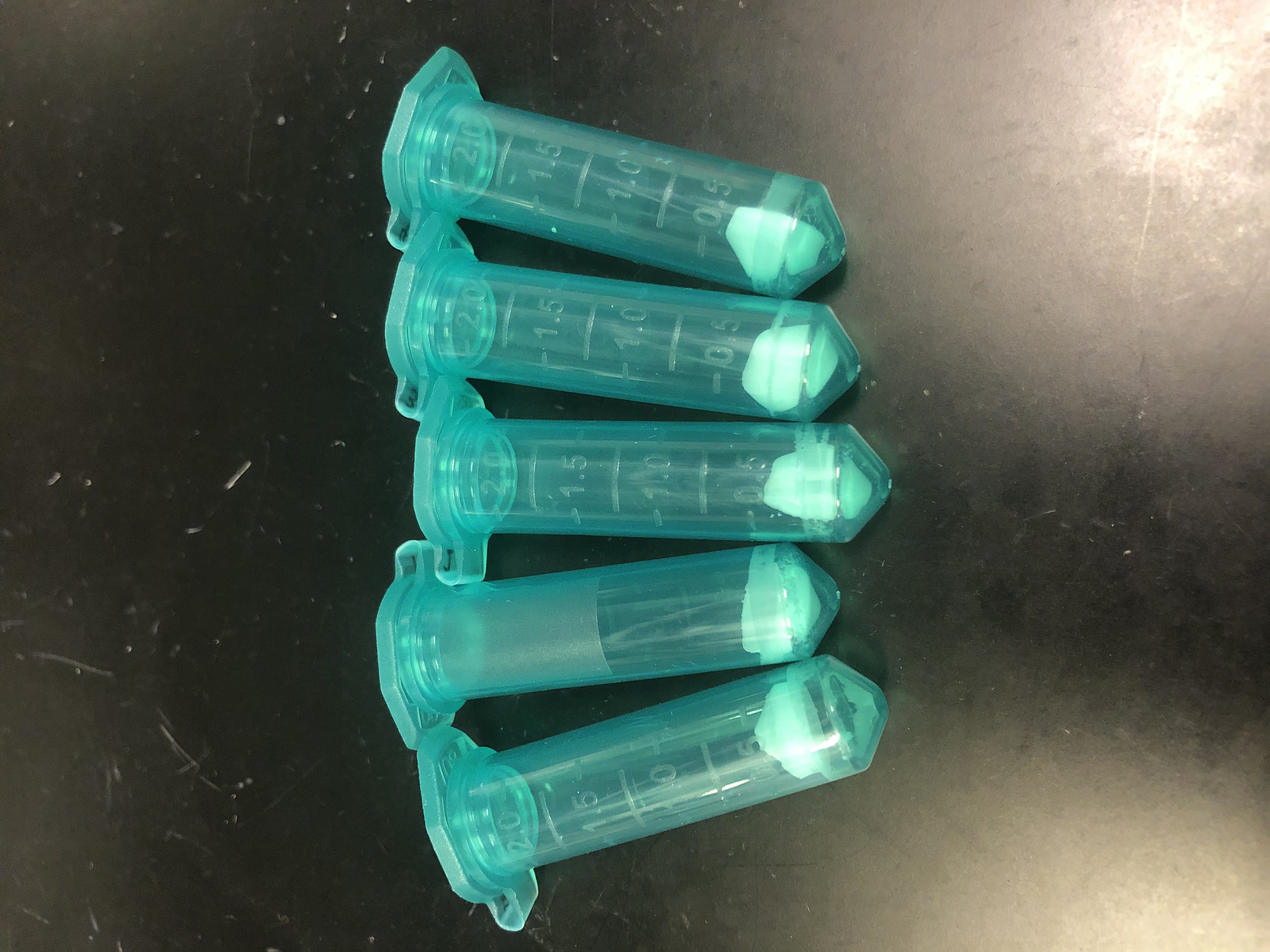

Supplement: Supplementary file 4 — Source Data [file 41467_2021_25989_MOESM4_ESM.zip › Image Files/Fig 6C & Supp Fig 17/FreezeDried_Run1/Run1_FD_T0_tubes.jpeg]

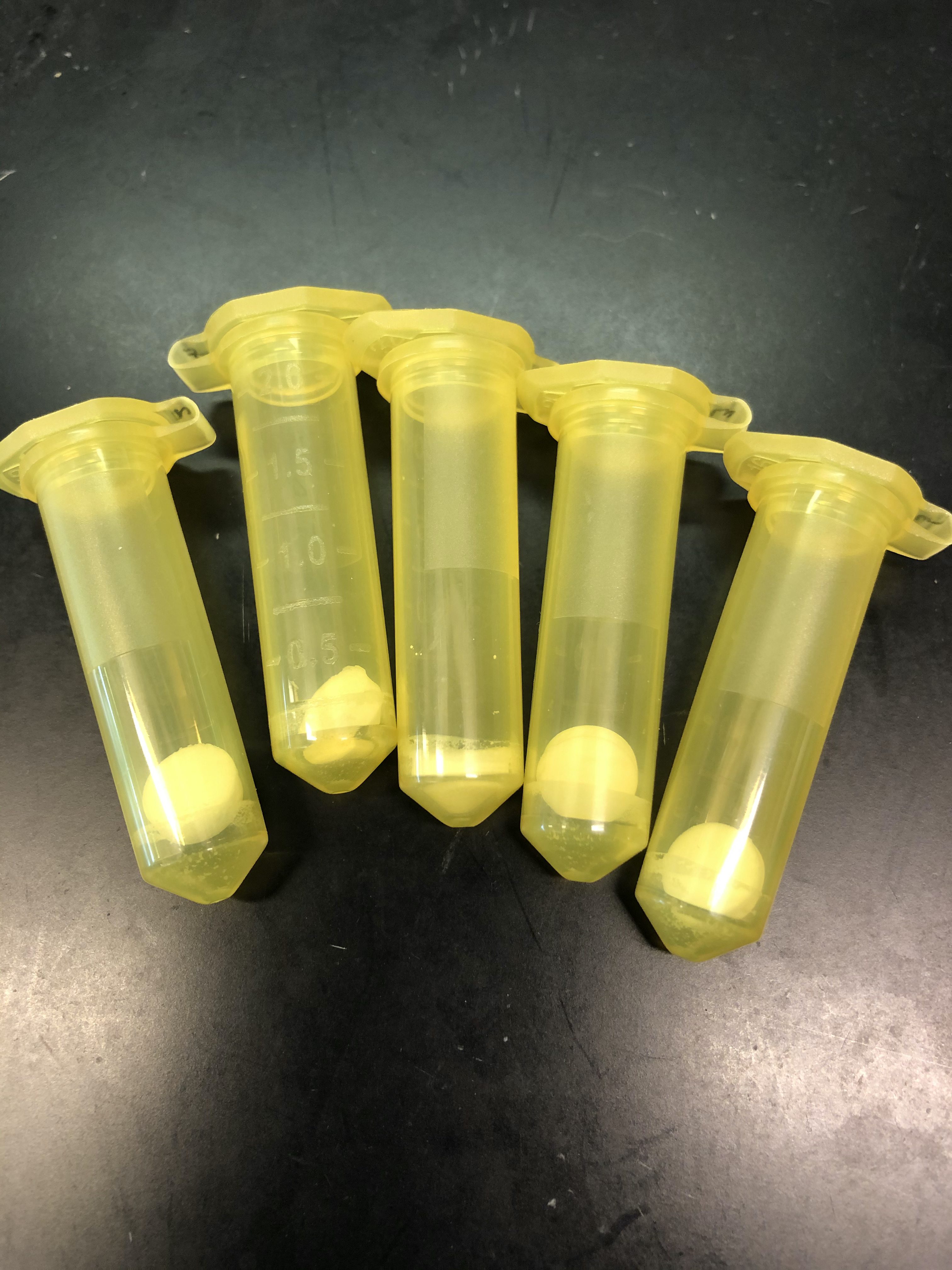

Supplement: Supplementary file 4 — Source Data [file 41467_2021_25989_MOESM4_ESM.zip › Image Files/Fig 6C & Supp Fig 17/FreezeDried_Run3/Run3_FD_T0_tubes.jpeg]

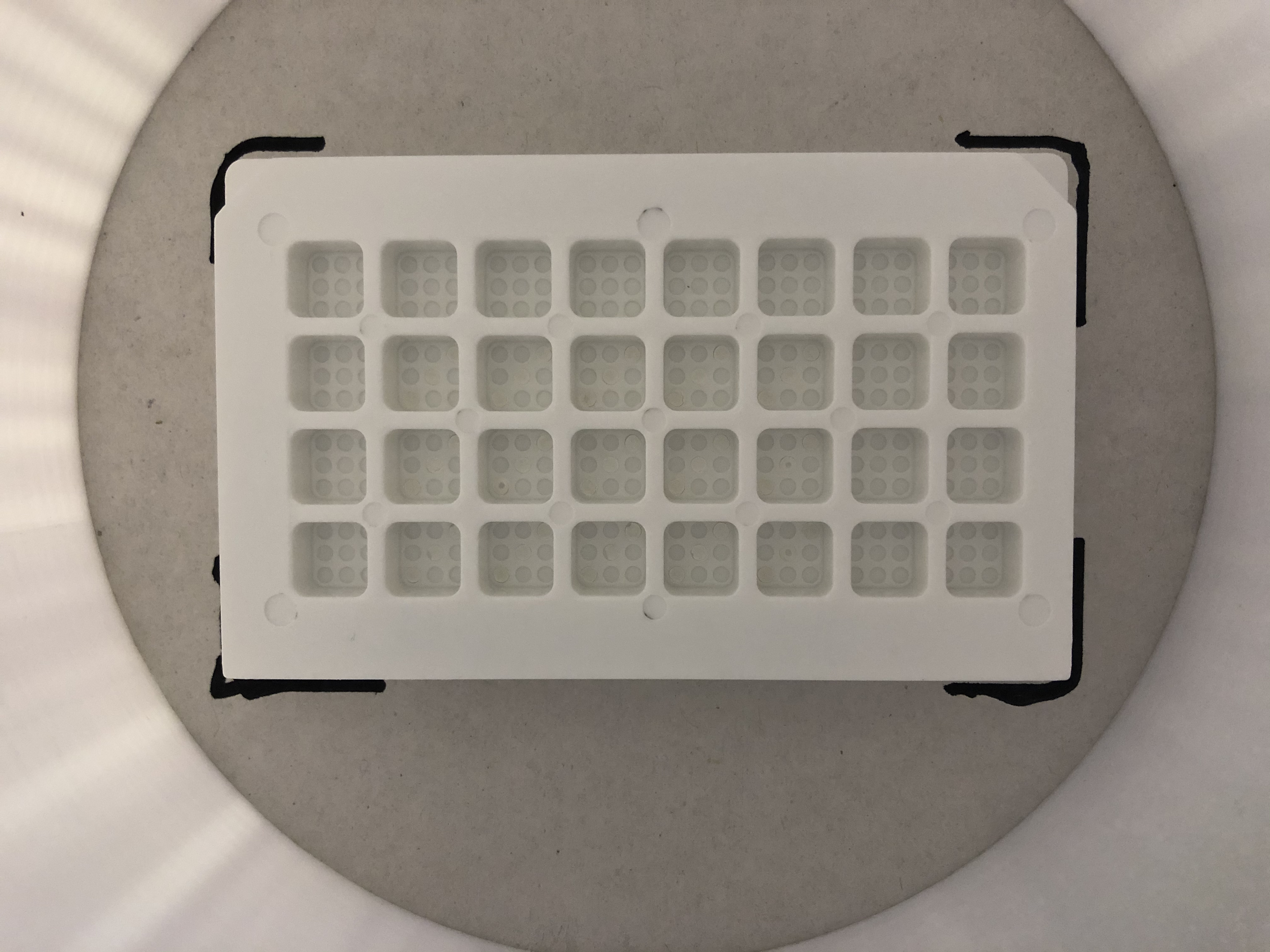

Supplement: Supplementary file 4 — Source Data [file 41467_2021_25989_MOESM4_ESM.zip › Image Files/Fig 6C & Supp Fig 17/FreezeDried_Run3/Run3_FD_T0_plate.jpeg]

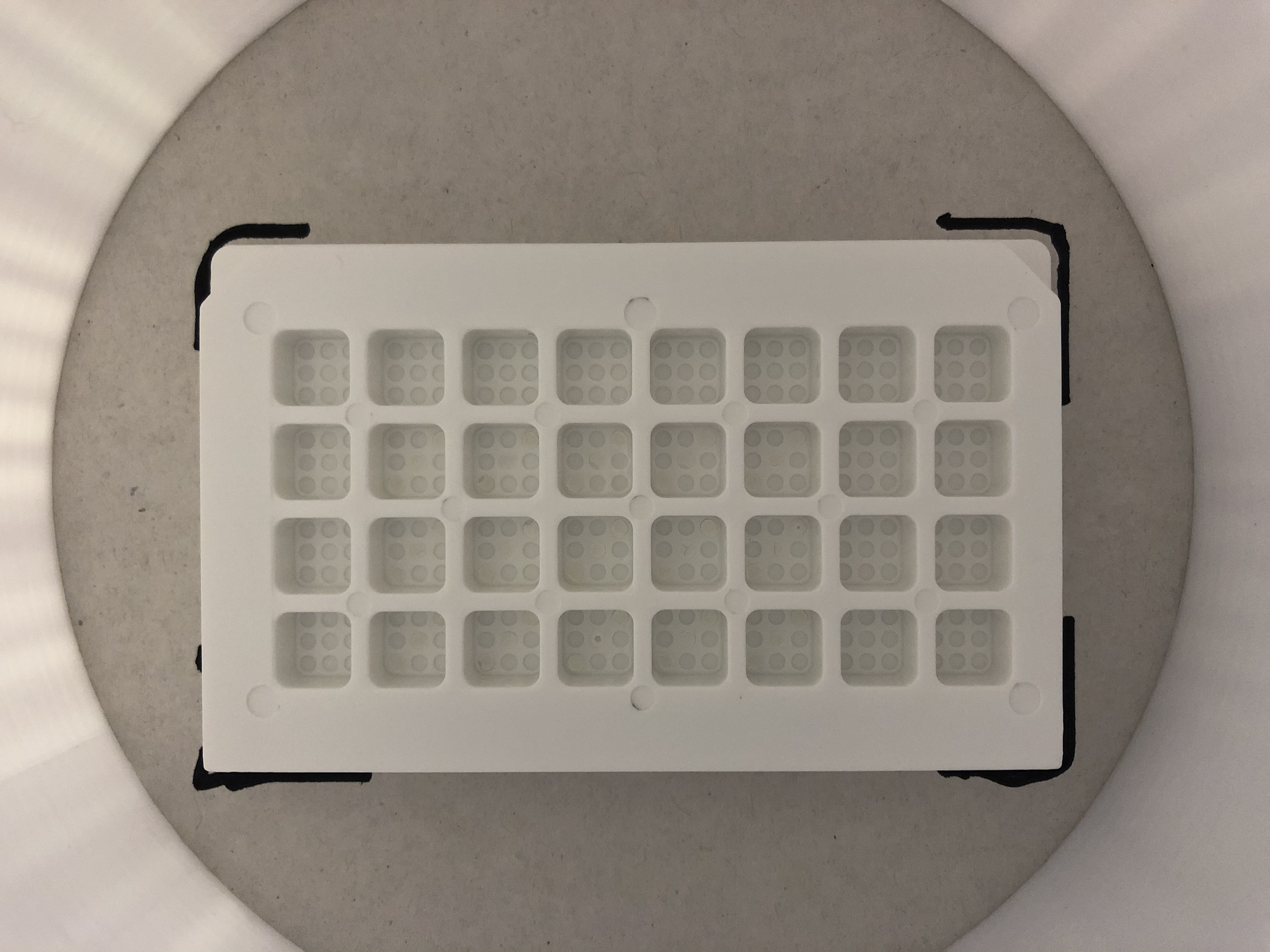

Supplement: Supplementary file 4 — Source Data [file 41467_2021_25989_MOESM4_ESM.zip › Image Files/Fig 6C & Supp Fig 17/FreezeDried_Run2/Run2_FD_T0_plates.jpeg]
